# Enhancement of disease resistance, growth potential, and photosynthesis in tomato (*Solanum lycopersicum*) by inoculation with an endophytic actinobacterium, *Streptomyces thermocarboxydus *strain BPSAC147

**DOI:** 10.1371/journal.pone.0219014

**Published:** 2019-07-03

**Authors:** Ajit Kumar Passari, Kalidas Upadhyaya, Garima Singh, Ahmed M. Abdel-Azeem, Sugitha Thankappan, Sivakumar Uthandi, Abeer Hashem, Elsayed Fathi Abd_Allah, Jahangir Ahmed Malik, Alqarawi AS, Vijai Kumar Gupta, Sanjay Ranjan, Bhim Pratap Singh

**Affiliations:** 1 Department of Biotechnology, Mizoram University, Aizawl, Mizoram, India; 2 Departamento de Biologia Molecular y Biotecnologia, Instituto de Investigaciones Biomedicas, Universidad Nacional Autonoma de Mexico (UNAM), Mexico, Mexico; 3 Department of Forestry, Mizoram University, Aizawl, Mizoram, India; 4 Department of Botany, Suez Canal University, Ismailia, Egypt; 5 Biocatalysts Lab, Department of Agricultural Microbiology, Tamil Nadu Agricultural University, Coimbatore, India; 6 Botany and Microbiology Department, College of Science, King Saud University, Riyadh, Saudi Arabia; 7 Mycology and Plant Disease Survey Department, Plant Pathology Research Institute, ARC, Giza, Egypt; 8 Department of Chemistry and Biotechnology, ERA Chair of Green Chemistry, Tallinn University of Technology, Tallinn, Estonia; 9 Plant Production Department, College of Food and Agricultural Sciences, King Saud University, Riyadh, Saudi Arabia; 10 Application Scientist, Spectraritec, Ranjit Nagar Commercial Complex, Saadipur, Delhi; Universite Paris-Sud, FRANCE

## Abstract

Biotic stresses in plants have a significant impact on agricultural productivity. In the present study, *in vivo* experiments were conducted to determine the physiological responses of tomato (*Solanum lycopersicum* L.) seedlings by inoculation with an endophytic actinobacterium, *Streptomyces thermocarboxydus* isolate BPSAC147 under greenhouse conditions. Further, photochemical quantum yield of photosystem II (PSII) (F_v_/F_m_), photochemical quenching (qP) and non-photochemical (NPQ) were calculated in seedlings inoculated with *S*. *thermocarboxydus* (T1) and were compared with control (T0) plants. Furthermore, the electron transport rate (ETR) of PSII exhibited a significant increase in **T1** plants, relative to **T0** plants. These results indicate that inoculation of tomato seedlings with *S*. *thermocarboxydus* had a positive effect on the process of photosynthesis, resulting in enhanced chlorophyll fluorescence parameters due to increased ETR in the thylakoid membrane. GC-MS analysis showed significant differences in the volatile compounds in the different treatments performed under greenhouse conditions. The present study suggests that *S*. *thermocarboxydus* can be used as new biocontrol agent to control *Fusarium* wilt in tomato crops and enhance productivity by enhancing photosynthesis.

## Introduction

Biotic stresses in plants, such as infections by pathogenic bacteria and fungi, significantly impact agricultural productivity. Fungi, in particular, are considered a major disease causative agent in plants and are known to alter photosynthetic-related metabolisms [1]. Therefore, it is critical to identify sustainable approaches to manage plant diseases and other plant biotic stresses. Plant growth promoting microorganisms (PGPM) are considered to have advantageous effects on plant health and nutrient uptake [1, 2]. PGPM influence plant growth and fitness by both direct and indirect mechanisms. Direct mechanisms include N_2_ fixation, Fe sequestration, phytohormone production, and phosphate solubilisation, while major indirect mechanisms are antibiosis and production of lytic enzymes [3]. The beneficial effects on plant growth mediated by rhizospheric bacteria and fungi have been well documented [4, 5]. The effect of endophytic actinobacteria on photosynthetic efficiency and plant growth, however, has not been widely studied [6, 7]. Endophytes are characterized by their ability to colonize the intracellular spaces and xylem conducting elements in plants without causing any disease symptoms [8, 9].

Studies have reported on the beneficial effect of PGPM, including endophytic bacteria, on plant physiology by their ability to suppress biotic stresses such as pathogenic bacteria and fungi, insects, and native plants [7]. Several questions regarding the association of endophytic microorganisms with host plants, however, are still unanswered; including how long do they reside in the host and do they have any impact on photosynthesis. Abd_Allah et al. [9] and Hashem et al. [3] reported that the endophytic bacterium, *Bacillus subtilis* (BERA 71), enhances the photosynthesis and growth of chickpea (*Beta vulgaris* L.) and mung bean via the production of phytohormones. Zhang et al. [10] suggested that plant growth promoting rhizobacteria may function as a plant growth stimulator through an auxin-dependent mechanism. The ability of endophytic actinobacteria to modulate biotic and abiotic stresses has been documented [11,12], however, studies relating to their effect on photosynthetic parameters have not been conducted. The effects of beneficial bacteria on plant photosynthesis have been examined for the first time as per Zhang et al. [13]. The parameters that have a significant effect on the photosynthetic apparatus are minimum fluorescence (F_o_), maximum photochemical quantum yield of PSII (Fv/Fm), NPQ and ETR [1, 14]. Relative ETR values determines the stress measurements among the treated and control plants under similar light absorption, similarly NPQ is measured by the quenching of chlorophyll fluorescence and is considered as an important photoprotective mechanism and higher qP indicate that more fluorescence is being quenched by the photochemical process [15]. Endophytic actinobacteria as a significant, potential source of secondary metabolites has been reported in the literature in recent years [16,17,18]. Among bacteria, *Streptomyces* alone account for more than 70% of the microbial natural products that have been documented and demonstrated to represent a potential source for the development of novel approaches for managing both biotic and abiotic stresses [19, 20, 21]. Among the bacteria, *Streptomyces* species are well known to produce a plethora of volatile organic compounds (VOCs) which may directly or indirectly influence the growth and gene expression of microorganism or plants [22, 23]. They are considered and recognized as ideal “infochemicals” as they can easily diffuse [24]. Researchers have documented them as potential agents in management of several phytopathogenic fungal diseases as compared to conventional fungicides [25, 26]. The present study was designed to understand the VPCs produced by the endophytic strain BPSAC147 having plant growth promoting potential.

We have identified several endophytic actinobacteria associated with traditional medicinal plants and documented their ability to produce phytohormones [27]. Endophytic actinobacteria have the potential to inhibit pathogens [7, 28], produce antimicrobial compounds [16] and synthesize metabolites of industrial importance [18, 29]. The current knowledge of endophytic actinobacteria clearly indicates their potential value for ameliorating biotic and abiotic stress in plants and improving plant productivity. The present study had a dual purpose, first to determine the potential of a PGP isolate of *Streptomyces* spp. to ameliorate the biotic stress in tomato seedlings caused by the fungal pathogen, *Fusarium*, under greenhouse conditions. In addition, the photosynthetic metabolism of inoculated and non-inoculated tomato seedlings was also assessed. We hypothesized that the inoculation of tomato seedlings with an endophytic actinobacterium, *Streptomyces thermocarboxydus* isolate BPSAC147, would have a positive effect on plant growth promotion by controlling *Fusarium* wilt disease and enhancing photosynthesis-related metabolism as evidenced by its impact on enhancing photosystem II (PSII) (F_v_/F_m_), ETR, and NPQ; as well as qP, compared to non-inoculated control plants.

## Materials and methods

### Sample collection and isolation of endophytic *Streptomyces* isolates

Healthy *Rhynchotoechum ellipticum* L. was collected based on traditional and ethnobotanical properties from Murlen National Park (23°36′N 93°16′E) in Mizoram, India. Permission for the collection of medicinal plants was obtained from Mr. Liandawla, Chief wildlife warden, Environment and forest department, Government of Mizoram, India. Sterilization of leaf surfaces was performed after plants were brought back to the laboratory as described by Passari et al. [30]. The disinfected leaf tissues were placed on actinomycetes isolation agar (AIA) media amended with cycloheximide (60 μg/ml) and nalidixic acid (80 μg/ml) to inhibit fungal growth, and to suppress the growth of fast-growing bacteria. Actinobacteria colonies were recultured several times until pure cultures were obtained. All four isolates of were deposited in Suez Canal University Fungarium (SCUF- (http://www.wfcc.info/ccinfo/index.php/collection/by_id/1180/) under the accession numbers SCUF 1520 to 1523.

### Tomato seed germination assay

Four endophytic *Streptomyces* isolates identified in a previous PGPR study [16] were evaluated in the seed germination assay. Tomato seeds were surface sterilized with 2.0% NaOCl for 2 min and subsequently rinsed three times with sterile distilled water. The tomato seeds were mixed with a suspension culture (10^−3^ cells/ml) of each *Streptomyces* isolate. After 2 h of soaking, 10 seeds were transferred into sterile petri dishes containing sterile moistened filter papers (Whatman filter paper). The petri dishes were kept at room temperature and assayed for germination. Controls seeds were surface sterilized and used as control without inoculation of bacterial suspension. All of the petri dishes were sprayed daily with sterilized distilled water. Germination rate, as measured by seedling emergence, was determined as previously described by Ranganathan and Thavaranjit [31] using the following formula:

% germination = number of seeds that germinated/total number of seeds ×100

### Identification and phylogenetic analysis of isolate BPSAC147

Sequencing of a fragment of the 16S-rRNA gene was performed as described by Passari et al. [30]. The evolutionary model was selected based on the lowest BIC (4464.838) and AIC (4276.621) using the MEGA 6.0 phylogenetic tree construction software. A neighbor-joining tree [32] was constructed using Kimura 2-parameter model (K2) with MEGA 6.0 [33] taking *Bacillus amyloliquefaciens* strain DSM7 as an outlier.

### *In vivo* plant growth promotion assay

Pot experiments were conducted to determine the effect of isolate BPSAC147 on plant growth and its biocontrol of *Fusarium oxysporum* on tomato (*Solanum lycopersicum*) seedlings under greenhouse conditions. Tomato (*Solanum lycopersicum*) seeds (PUSA-120) were acquired from Indian Agricultural Research Institute (IARI), New Delhi, India and surface sterilized in 70% ethanol for 5 min followed by 2% NaOCl for 3 minutes and then rinsed with sterile distilled water three times. Surface sterilized seeds were placed in plastic bags for germination prior to growing plants in the greenhouse. After 20 days, the germinated tomato seedlings were planted separately in individual plastic bags (30×15×20 cm) containing sterilized soil. A suspension of isolate BPSAC147 (10^−6^ CFUmL^-1^) was applied to the tomato seedlings growing in the greenhouse. The experiment consisted of four treatments. **T0:** control, 12 tomato seedlings without addition of the BPSAC147 isolate; **T1:** tomato seedlings inoculated with isolate BPSAC147; **T2:** tomato seedlings + isolate BPSAC147 + *Fusarium oxysporum* (10^−3^ CFUmL^-1^), and; **T3:** Tomato seedlings + *Fusarium oxysporum* (10^−3^ CFUmL^-1^). The first two treatments were used to evaluate the PGP properties of BPSAC147 and the other two treatments were conducted to evaluate the biocontrol capacity of BPSAC147 against *F*. *oxysporum*. Plants were grown at 25–32 ^0^C, re-inoculated each week, and watered on a daily basis until plants were harvested. After a period of 60 days, six plants from each treatment were uprooted and measurements were taken of shoot and root, length, fresh weight, and dry weight. Dry weights were measured as described by Barnawal et al. [4]. All plants were maintained in the greenhouse for 60 days. The percent disease index was calculated according to Shanmugam and Kanoujia [34] as follows: Disease index = [R (rating × number of plants rated)/Total number of plants × highest rating] × 100. Data were statistically analysed using a one-way ANOVA and LSD tests at p < 0.05. All of the experiments were conducted four times with twelve replicates for each treatment in each experiment.

### Estimation of chlorophyll

For chlorophyll extraction, 0.5 g of fresh leaves were obtained from plants in each treatment and ground in 10–20 ml of 80% acetone. The obtained solution from each sample was transferred in a 15 ml centrifuge tube and centrifuged at 6000 rpm for 10 min. The supernatant was transferred to another centrifuge tube and the chlorophyll extraction procedure was repeated until the residue was colourless. Absorbance of the final supernatant was measured at 645 nm and 663 nm using a Multiscan GO (ThermoScientific) spectrophotometer. The acetone solvent was used as a blank. The concentrations of chlorophyll a, b and total chlorophyll were calculated using the following formula as per Ni et al. [35].:

Total Chlorophyll: 20.2(A645) + 8.02(A663)

Chlorophyll a: 12.7(A663)– 2.69(A645)

Chlorophyll b: 22.9(A645)– 4.68(A663)

### Photosynthesis measurements

Leaves from replicate plants in each of the treatments were taken and placed in the dark for 30 min. Subsequently, chlorophyll (Chl) fluorescence parameters were estimated with the use of a portable pulse amplitude modulation dual pam fluorometer (Model No: Dual Pam100.Walz; Company: Germany). The maximum photosynthetic quantum efficiency of photosystem II (*F*v/*F*m) was determined using chlorophyll fluorescence where *F*v is maximum variable fluorescence (*F*m–*F*o), *F*o is minimum Chl fluorescence yield in the dark-adapted state and *F*m is maximum Chl fluorescence yield in the dark-adapted state. The leaves were kept in the holder of the fluorometer and then minimal fluorescence (*F*o) was calculated. *F*m was evaluated after exposing leaves to a 0.6 s saturating pulse of light, while a saturating pulse of white light was supplied at 10 min intervals for 800 ms at about 6000 μmol photons m^-2^s^-1^ to calculate the greatest (*F*m') and lowest (*F*o') fluorescence intensity; as well as the steady state fluorescence intensity in the light-adapted state (*F*s) as per Calatayud et al. [36]. Estimates of ETR = *Φ*_PSII_ ×PAR×0.5× leaf absorptivity coefficient) and PSII efficiency (*Φ*_PSII_ = (*F*m'-*F*s')/*F*m’) were determined by increasing PAR up to eleven saturation pulses from 0 to 849 μmol photons m^-2^s^-1^. Moreover, the efficiency of qP = [(*F*m'- *F*s)/ (*F*m'- *F*o')], NPQ = [(*F*m- *F*m')/ *F*m']), and the coefficient of photochemical [qL = qP × (*F*o/*F*s′)] fluorescence quenching was determined according to Ribeiro et al. [37] and Samaniego-Gamez et al. [1].

### Determination of volatile compounds by GC-MS analysis

Isolate BPSAC147 was inoculated into ISP1 broth and cultured at 28°C for seven days to detect volatile compounds. Headspace samples of ISP1 broth were used as negative controls. Volatile compounds present in the headspace samples were determined using GC/MS and a Thermal Desorber Turbomatrix 150 (Perkin Elmer, USA). The GC-MS conditions are as follows: 10:1 split, 20 psi carrier as Helium gas, 50 to 250°C at 10°C per min for oven temperature, electron impact spectra at 70 eV and positive ion mode whereas, the analysis was performed using 30 m × 250 μm capillary column with 5% phenyl-methyl siloxane. The VOC were identified by comparing the obtained mass spectra with NIST (National Institute of Standards and Technology) 14 Mass Spectral Library (NIST/EPA/NIH). VOC detection and analysis were carried out as described by Lee et al. [38]. Volatile compounds that exhibited more than >90% similarity with mass spectra in the NIST library were placed on a “positive list” of tentative compounds.

Volatiles from tomato leaves collected from each treatment were determined by grinding them in a mixer/grinder. The resulting mixture was placed in methanol for 24 h and the extract was subsequently filtered through Whatman no. 1 filter paper. The filtered solvent was dried at 45°C using a rotary evaporator system (BUCHI, Switzerland) to acquire a crude extract. The crude extract from each treatment was used to identify volatile compounds using GC-MS analysis. The spectra of the obtained volatile compounds were matched against spectra in the GC-MS NIST (2014).

### Statistical analysis

All the treatment plants consisted of four replicates with 12 plants per replicate and the results are presented as mean ± standard error (SE). Data were statically analysed using a one way ANOVA and least significant difference (LSD) tests at p≤0.05 and p ≤0.01. Similarly, the chlorophyll data were calculated using three biological replicates and the results are presented as a mean ± standard error (SE) with least significant difference (LSD) tests conducted at p≤0.05 and p ≤0.01. Statistical analysis was performed to check difference in metabolites of tomato plant extract using METABOANALYST 4.0.

## Results

### Seed germination assay

**Fig 1:**
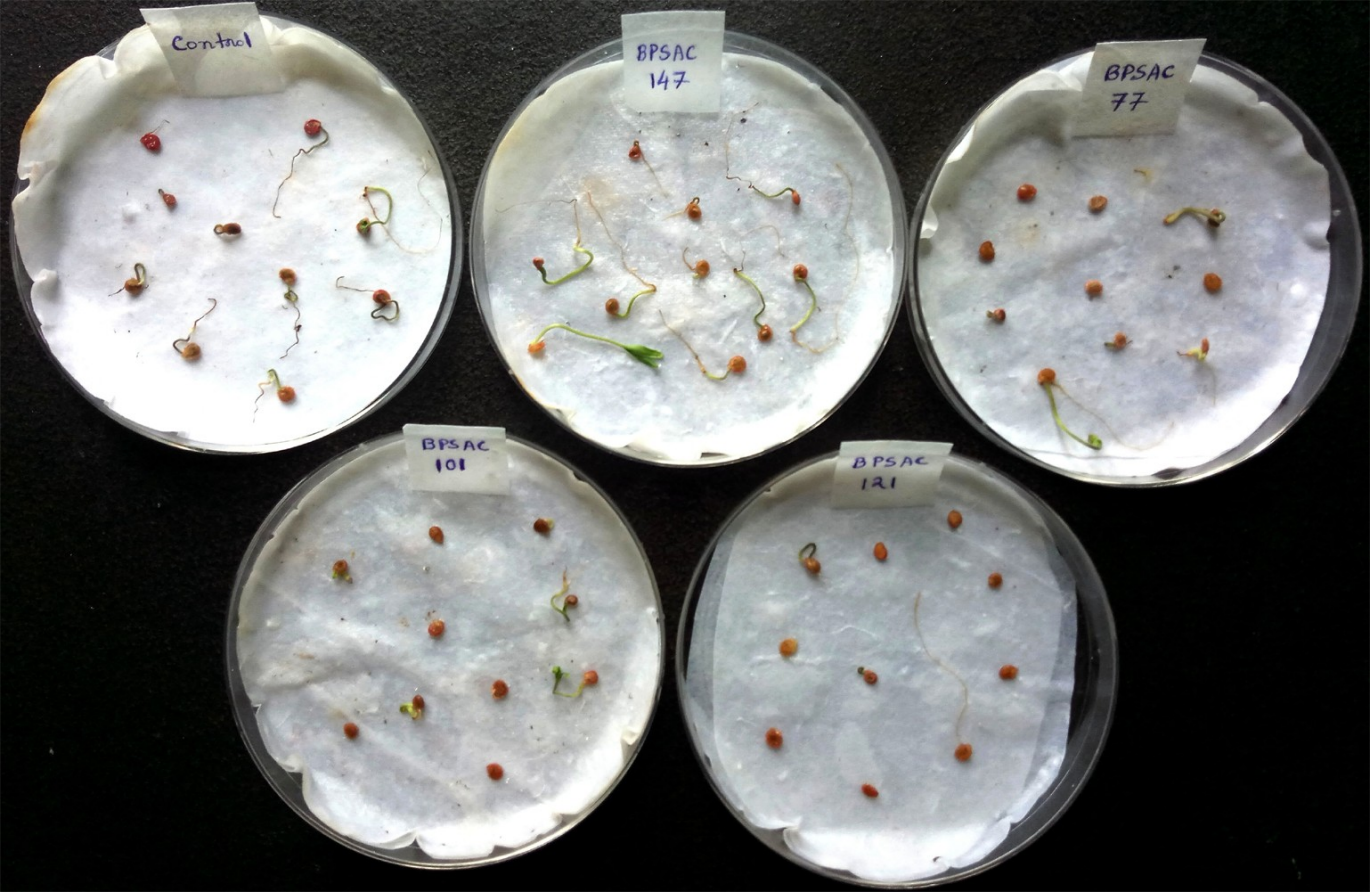
Seed germination rate in tomato inoculated with four different isolates (BPSAC77; BPSAC101; BPSAC121 and BPSAC147) compared with the control.

Four isolates (BPSAC77, BPSAC101, BPSAC121, and BPSAC147) were selected for further evaluation in the seed germination assay based on a previous PGPR study [16]. Ten tomato seeds inoculated with the four individual *Streptomyces* isolates exhibited increased seedling growth compared to non-inoculated control plants. Relative to control seedlings (n = 9; 90%), the most significant effect on tomato seedling growth was observed in seedlings inoculated with BPSAC147 (n = 10; 100%), followed by BPSAC77 (n = 5; 50%), BPSAC101 (n = 4; 40%) and BPSAC121 (n = 3; 30%) (**Fig 1**). There was also a significant effect of the bacterial isolates on the germination rate of tomato seeds, relative to non-inoculated seeds. Seeds treated with BPSAC147 exhibited maximum germination relative to the controls and other isolates. Thus, isolate BPSAC147 was selected for further study.

### Amplification of 16S-rRNA gene and phylogenetic analysis

**Fig 2:**
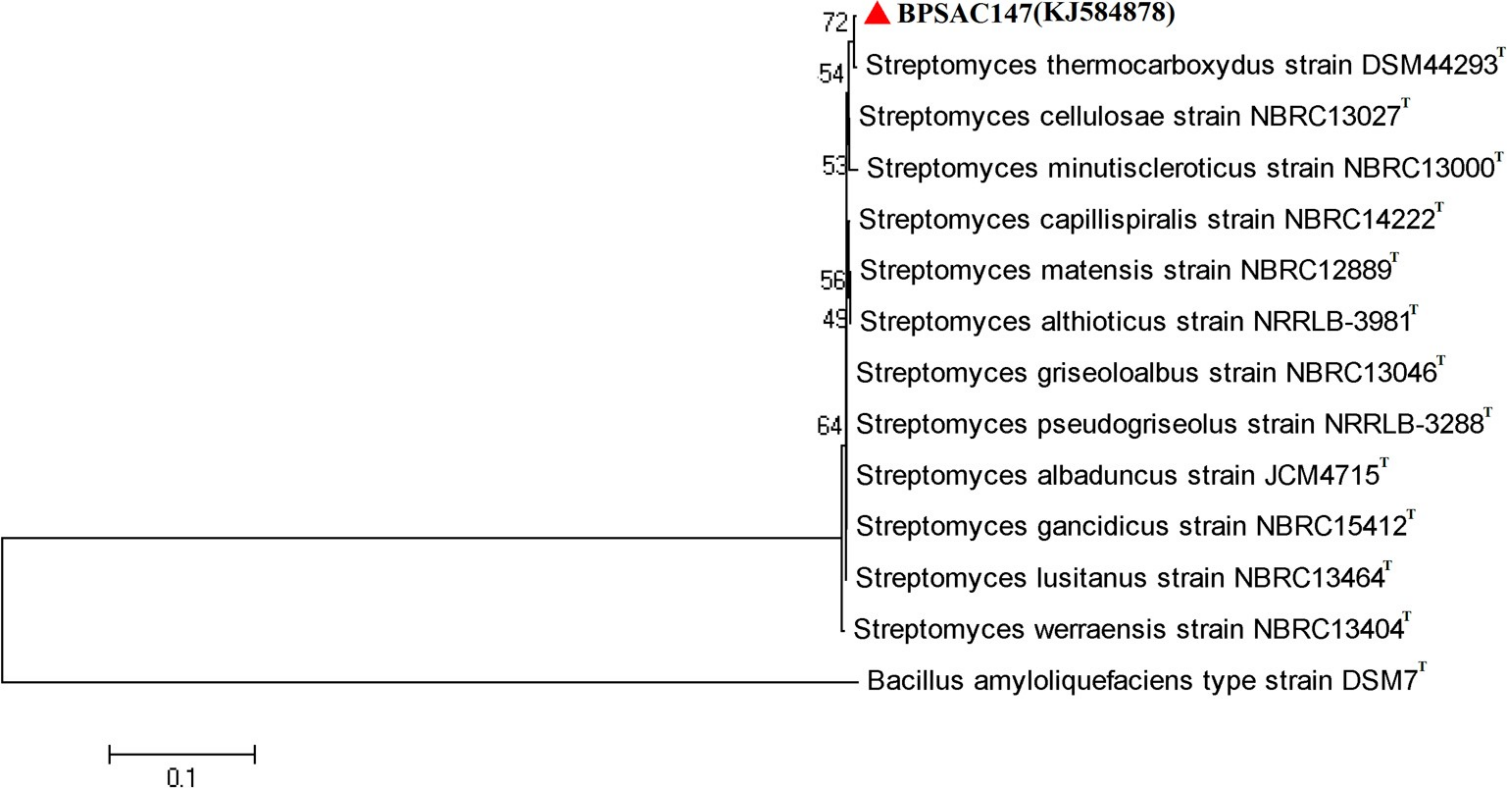
Neighbour joining phylogenetic tree using Tamura 3-parameter model based on 16S rRNA gene sequence of *Streptomyces thermocarboxydus* isolate BPSAC147 was constructed to compare with closest type strains retrieved from EzTaxon database. Numbers at branches indicate bootstrap values in 1,000 replicates.

A fragment of the 16S-rRNA gene of BPSAC147, which exhibited significant PGPR activity, was sequenced. Based on the obtained sequence, BPSAC147 was identified as *Streptomyces thermocarboxydus* and the sequences were deposited in NCBI GenBank (Accession number KJ584878). The 16S sequence of BPSAC147 exhibited 99.75% similarity to *Streptomyces thermocarboxydus* isolate DSM 44293 in the EZtaxon database; whereas, BPSAC147 exhibited 99.5% similarity with *Streptomyces cellulosae* strain NBRC 13027, 98.88% similarity to *Streptomyces minutiscleroticus* strain NBRC 13000, 99.13% similarity to *Streptomyces capillispiralis* strain NBRC14222 and 99.12% similarity to *Streptomyces matensis* strain NBRC12889. The sequence of BPSAC147 and 12 reference isolates recovered from the EzTaxon database were used to construct a phylogenetic tree. The tree was made based on a Kimura-2 parameter model using the neighbour joining method. The resulting phylogenetic tree indicated that isolate BPSAC147 was closely similar to *Streptomyces thermocarboxydus* isolate DSM 44293 with a 72% bootstrap supported value (**Fig 2**).

### *In-vivo* plant growth promotion assay

The effect of *Streptomyces thermocarboxydus* isolate BPSAC147 on growth promotion and its biocontrol activity against *F*. *oxysporum* was evaluated in tomato plants under greenhouse conditions. At 60 days after treatment, shoot length was significantly longer in tomato plants that were inoculated with isolate BPSAC147 (T1 = 117.6 cm), compared to non-inoculated control plants (T0 = 110.85 cm). Similarly, greater root length was observed in tomato plants inoculated with isolate BPSAC147 (T1 = 37.16 cm) than in control plants (T0 = 25.9 cm). Additionally, higher shoot and root dry weights were attained in T1 (4.44 gm and 1.5 gm, respectively) plants than in control, T0 (3.49 gm and 1.03 gm) plants (**S1 Table and S1 Fig**).

**Fig 3:**
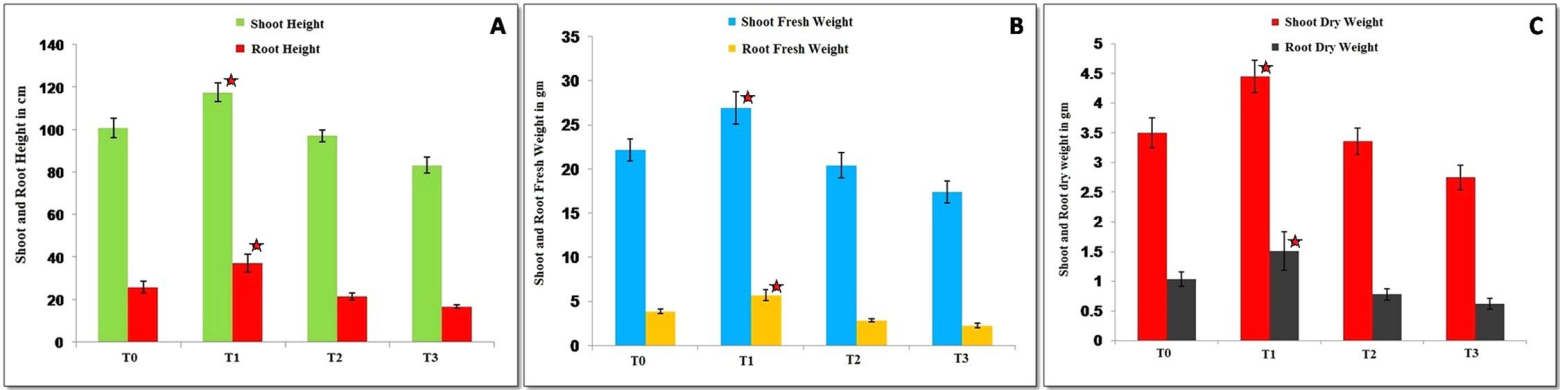
Effect of potential endophytic isolate *Streptomyces thermocarboxydus* isolate BPSAC147 on shoot length, root length, plant weight and biocontrol effect of tomato seedlings after 60 days. (**A**) Shoot and Root height in cm after 60 days; (**B**) Here, Shoot and Root Fresh weight in gm after 60 days and (**C**) Shoot and Root dry height in gm after 60 days. Here **T0**. Tomato plants grown in greenhouse as control; **T1**. Tomato plants grown in greenhouse by isolate BPSAC147 (PGPR effect); **T2.** Tomato plants grown in greenhouse by isolate BPSAC147 with fungal pathogen *Fusarium oxysporum* (Biocontrol effect); **T3.** Tomato plants grown in greenhouse fungal pathogen *Fusarium oxysporum*. The graphs were presented as a mean ± standard error (SE). Star (*) indicates statistically significant differences in treatment T1 as compare to other treatments.

The biocontrol activity of isolate BPSAC147 against *F*. *oxysporum* was very high. Observations of wilting were made 60 days after inoculation of BPSAC147 and the pathogen. The T2 treatment (PGP isolate BPSAC147 + *F*. *oxysporum*) exhibited greater shoot and root length (97.1 cm and 21.45 cm, respectively) than the T3 treatment (*F*. *oxysporum* alone). Treatment with the pathogen alone (T3) decreased shoot length, root length, and fresh weight of the plant while the T2 treatment inhibited infection by *F*. *oxysporum* and tomato plants appeared healthy. There were no significant difference in tomato shoot length, root length, shoot and root weight between the T2 with control T0 treatments after 60 days (**Fig 3 and Table 1**).

After 60 days, decrease in disease incidence was indicated in the tested T2 treatment as compare to treatment T3. We have found that treatment T2 (strain BPSAC147+ *Fusarium oxysporum*) showed highest decrease in diseases index (42.3%) as compared to treatment T3 (only *Fusarium oxysporum*) (54.9%).

**Table 1:** *In-Vivo* plant growth promotion assay in tomato plant after 60 days transplantation.

Treatments Label	Shoot length (cm)	Root length (cm)	Shoot: Root (length ratio)	Shoot Weight		Root Weight		Total dry weight (gm)	Shoot: Root (dry ratio)
				Fresh (gm)	Dry (gm)	Fresh (gm)	Dry (gm)		
60 days after transplantation									
**T0 (PGPR -)**	100.85±4.61	25.90±2.63	3.89:1	22.17±1.22	3.49±0.25	3.91±0.27	1.03±0.12	2.26±0.31	3.38:1
**T1 (PGPR +)**	117.6±4.47	37.16±4.09	3.16:1	26.91±1.83	4.44±0.27	5.73±0.62	1.5±0.32	2.97±0.39	2.96:1
**T2 (PGPR/Disease +)**	97.10±2.87	21.45±1.63	4.52:1	20.41±1.42	3.35±0.22	2.86±0.21	0.77±0.09	2.06±0.31	4.35:1
**T3 (Disease -)**	83.3±3.69	16.70±0.83	4.98:1	17.42±1.24	2.74±0.20	2.27±0.27	0.62±0.09	1.68±0.26	4.41:1
**LSD @ 5%**	9.44	6.17	-	3.45	0.572	0.912	0.441	0.505	-
**LSD @ 1%**	13.56	8.87	-	4.95	0.823	1.31	0.633	0.724	-

### Chlorophyll content

An estimation of chlorophyll content was determined in plants from the four different treatments after 60 days. The analysis was conducted on fresh tomato leaf samples extracted with acetone solvent. Absorbance of the resulting extracts was recorded at 645 nm and 663 nm. Results indicated that the concentration of chlorophyll a & b after 60 days of growth was highest in T1 (18.545 μg/ml & 16.104 μg/ml, respectively) plants followed by T0 (8.455 μg/ml & 7.246 μg/ml, respectively) plants, T2 (6.607 μg/ml & 6.022 μg/ml, respectively) plants, and T3 (4.206 μg/ml & 5.044 μg/ml, respectively) plants. Similarly, maximum total chlorophyll content (a + b) was found in T1 (34.649 μg/ml) plants, T0 (15.701 μg/ml), T2 (12.629 μg/ml), and T3 (9.25 μg/ml) plants (**Tables 2 and S2)**.

**Table 2:** Chlorophyll content in treatment tomato plant.

Treatment Label	Chlorophyll a (μg/ml)	Chlorophyll b (μg/ml)	Total Chlorophyll (a+b) (μg/ml)
**T0 (PGPR -)**	8.455	7.246	15.701
**T1 (PGPR +)**	18.545	16.104	34.649
**T2 (PGPR/Disease +)**	6.607	6.022	12.629
**T3 (Disease -)**	4.206	5.044	9.25
**5% LSD level**	11.818		

### Fluorescence parameters and photosynthesis

**Fig 4:**
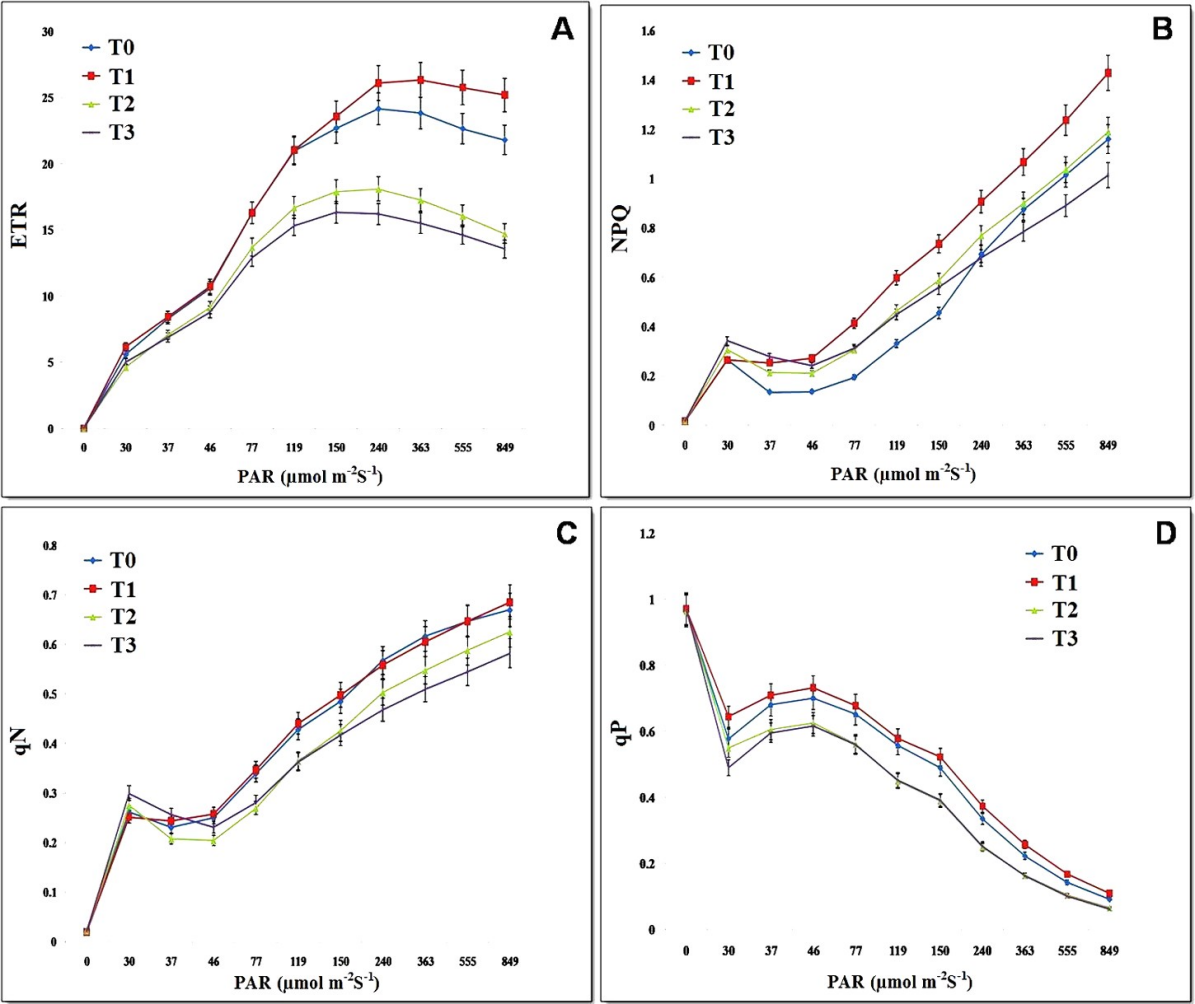
Effect of PGPR treatments on photosynthesis parameters; **A)** Electron transport rate (ETR); **B)** Non-photochemical fluorescence quenching (NPQ); **C)** Non-photochemical quenching (qN); **D)** Photochemical quenching (qP) of healthy and infected tomato leaf.

Measurements of photosynthesis and fluorescence parameters were taken on leaves of plants from each of the previously described treatments. Plants in the greenhouse were grown under a regime of 10 h light and 25–28°C temperature. Results indicated that the ETR of PSII at 849 μmol photons m^-2^s^-1^was highest in leaves obtained from T1 (25.2) plants, followed by leaves from T0 (21.8), T2 (14.73), and T3 (13.56) plants. Leaves of plants inoculated with BPSAC147 (T1 treatment) reached maximum ETR (26.33) at 363 μmol photons m^-2^s^-1^ whereas leaves from T0 (control) and T2 (inoculated with BPSAC147 and *F*. *oxysporum*) plants reached a maximum ETR (T0 = 24.16 & T2 = 18.1) at 240 μmol photons m^-2^s^-1^. Moreover, at 150 and 240 μmol photons m^-2^s^-1^, leaves obtained from T3 plants exhibited the highest ETR value (16.33). The maximum yield of PSII at 849 μmol photons m^-2^s^-1^was observed in leaves of T1 (0.0707) plants followed by leaves from T0 (0.061), T2 (0.041), and T3 (0.038) plants. **Fig 4**illustrates changes in qP in response to increasing irradiance. The presence of isolate BPSC147 (T1) in tomato plants resulted in changes in the quenching coefficients. Leaves of plants from the T1 treatment exhibited higher qP (0.7307) at 46 μmol photons m^-2^s^-1^ compared to leaves from T0 (0.7) plants. Notably, leaves of plants inoculated with BPSAC147 and the pathogen (T2 treatment) exhibited a higher qP (0.6043) at 37 μmol photons m^-2^s^-1^ than leaves of plants inoculated with just the pathogen (0.595) (T3 treatment). Similarly, leaves of T1 plants exhibited a higher NPQ (1.43) at 849 μmol photons m^-2^s^-1^ than leaves from T0 (1.37), T2 (1.19), and T3 (1.01) plants. Interestingly, NPQ began to gradually decrease in leaves of T3 plants (0.4503) at (119 μmol photons m^-2^s^-1^) compared to leaves of T2 plants (0.466). The qN at 849 μmol photons m^-2^s^-1^ was highest (0.6853) in leaves of T1 plants, followed by leaves from T0 (0.6697), T2 (0.6253) and T3 (0.582) plants. Results indicated a rapid increase in qN in T1 plants, while a slow increase in qN values was observed in T3 plants inoculated with just the pathogen. A significantly (5% of LSD level) higher Fv/Fm (0.812) value was observed in T1 plants inoculated with BPSAC147 than in T0 (0.790) non-inoculated, control plants. The value of ETR and chlorophyll content was also higher in T1 plants. Notably, T2 plants, inoculated with BPSAC147 and the pathogen, exhibited a significantly (5% LSD level) higher an Fv/Fm (0.781) value than T3 (0.706) plants which were inoculated with just the pathogen (**S3 Table**).

### GC-MS analysis

#### Detection of volatile compounds produced by isolates BPSAC147

A total of 35 volatile compounds were identified to be emitted by BPSAC147 using GC-MS analysis (**S4 Table**). The highest percentage of peak area (17.959) was observed for 2, 6, 10-Dodecatrien-1-ol, 3, 7, 11-trimethyl with a retention time 16.895, whereas the lowest percentage of peak area (0.894) was absorbed for credence with a retention-time of 15.244. Most of the detected compounds have been reported to possess antimicrobial and antioxidant activity.

**Fig 5:**
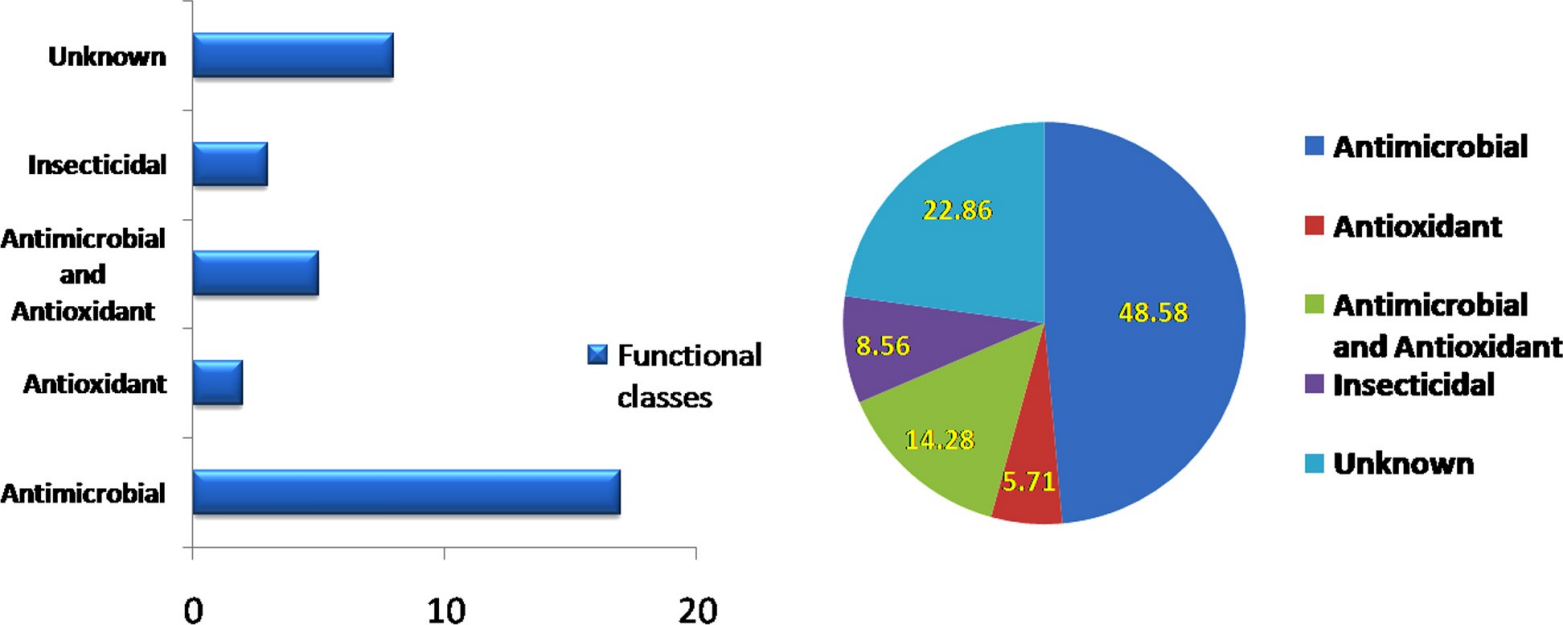
Functional classes of mVOCs produced by BPSAC 147, as analyzed in GC-MS-TD.

Among the volatile organic compounds detected, 17 possess (48.5%) antimicrobial properties, 2 were potent antioxidants (5.71%), whereas 14.5% exhibited both properties. In addition, 3 compounds (8.56%) pronounced insecticidal properties and acts as a serum protease inhibitor (**Fig 5**). The signature VOCs of *Streptomyces thermocarboxydus* BPSAC147 are 1H-pyrazole, 1,3 benzothiazole, 7-epi-transsesquisabinene, cedrene, azulene derivatives and piperoidene. The compounds identified in the TD spectrum didn’t show any match with pubchem and KEGG compounds in metaboanalyst analysis.

#### Detection of volatile compounds in tomato leaf extracts obtained from the different treatment groups

Extracts from leaves obtained from the four different treatment groups were analyzed. A total of 28, 30, 29 and 28 volatile organic compounds (VOCs) were identified in leaf extracts obtained from T0, T1, T2, and T3 plants, respectively. The greatest percentage of peak area (5.473) in T0 extracts was observed for dodecyl acrylate with a retention time of 15.804, while the lowest percentage of peak area (0.314) was observed for 3-Desoxo-3, 16-dihydroxy-12-desoxyphorbol 3, 13, 16, 20-tetraacetate with a retention time of 33.886. Similarly, the greatest percentage of peak area (6.905) in T1 extracts was observes for dodecyl acrylate with a retention time of 15.828, while the lowest percentage of peak area (0.433) was observed for 17-(1, 5-Dimethylhexyl)-10, 13-dimethyl-3-styryl hexadecahydro cyclopenta [a] phenanthren-2-one with a retention time of 31.009. The greatest percentage of peak area (3.161%) in extracts of T2 plants was observed for [2, 4, 6-Decatrienoic acid, 1a, 2, 5, 5a, 6, 9, 10, 10aoctahydro-5, 5a-dihydroxy-4-(hydroxyl methyl) 1, 7, 9-trimethyl-1-[(2-methyl-1-oxo-2-butenyl)oxy]methyl]-11-oxo-1H 2,8 amethanocyclopenta[a] cyclopropa [e]cyclodecen-6-yl ester] with a retention time of 30.034, while the smallest percentage of peak area (0.598%) was observed for strychane, 1-acetyl-20à-hydroxy-16-methylene- with a retention time of 29.639. Lastly, the greatest percentage peak area (8.013%) in extracts of T3 plants was observed for dodecyl acrylate with a retention time of 15.828, while the lowest percentage peak area (0.239%) was observed for 2-Nonadecanone 2, 4-dinitrophenylhydrazine with a retention time of 21.601.

The GC-MS analysis indicated that two volatile compounds (Benzene, 1, 3-bis (1, 1-dimethylethyl) and Dodecyl acrylate) were found in extracts obtained from the T0, T1, and T3 plants. Interestingly, only two compounds (1-Dodecanol and 9, 19-Cyclolanostan-3-ol, 24, 24-epoxymethano-, acetate) that could potentially promote plant growth were detected in extracts of both T0 and T1 plants (https://www3.epa.gov/pesticides/chem_search/reg_actions/reregistration/fs_G-5_1-Jun-07.pdf). Four compounds (2, 4-Di-tert-butylphenol; Hexadecanoic acid, methyl ester; Heptadecanoic acid, 16-methyl-, methyl ester and 3, 8, 12-Tri-O-acetoxy-7-desoxyingol-7-one) were detected in extracts of both T0 and T3 plants that may be defense-related compounds protecting the plants against the fungal pathogen (**S5 Table and S2–S5 Figs**).

#### GC-MS analysis of metabolites in tomato leaf extracts obtained from the different treatment groups

**Fig 6:**
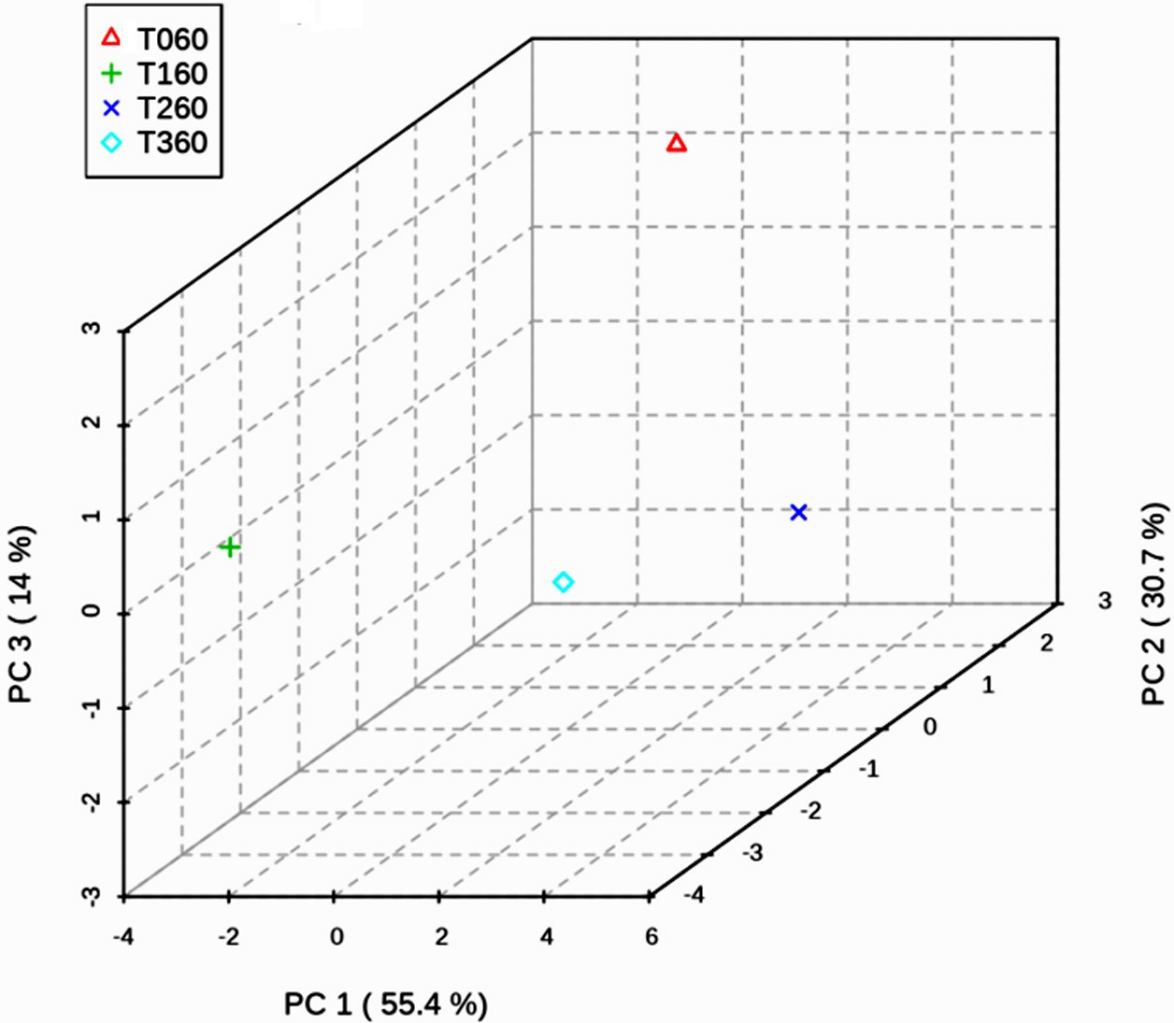
3D score plot between the selected PCs. The explained variances are shown in brackets.

**Fig 7:**
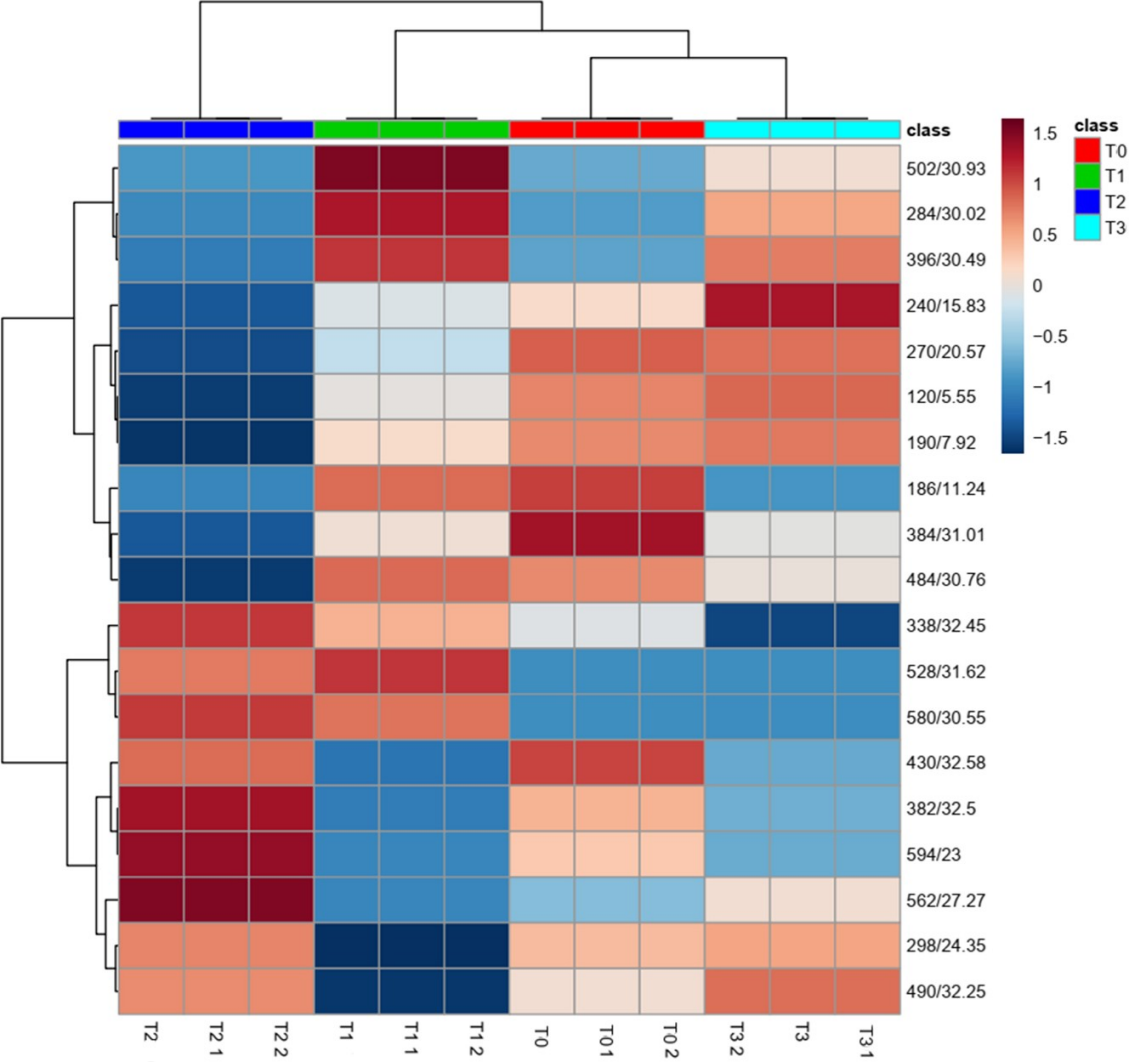
Hierarchical cluster analysis for the metabolites of tomato plant leaf extracts on 60 days result shown as heat map.

To further reveal the difference between the metabolite footprint of 60 d among the various treatments, statistical analysis was performed in METABOANALYST 4.0 and observed that the relative abundance of metabolites exhibited no statistical differences between the control and treatments. The metabolite identified in tomato leaves treated with *Streptomyces thermocarboxydus* BPSAC147 registered unique compounds when compared with its interaction with *F*. *oxysporum* and *F*. *oxysporum* alone. The metabolite detected in the interaction of *Streptomyces thermocarboxydus* BPSAC147 and *F*. *oxysporum* is a tetracycline compound that exhibits antimicrobial property whereas betulin has anticancer property. The hierarchical clustering of metabolites on 60 d showed three major clusters with T1, T3 and Control (T0) vs T2 (**Fig 6**). The PCA analysis also revealed thee clusters with PC1, PC2 and PC3contributing 55.4%, 30.7% and 14% variance respectively as depicted in 3-D score plot (**Fig 7 and S6 Table**).

## Discussion

The use of chemical fertilizers has become common place which has had a harmful impact on the environment. Hence, it is essential to identify endophytic microorganisms that can be used as a method for increasing plant host resistance to disease and the improvement of soil health [4]. In this regard, a number of actinobacteria have been reported to stimulate plant growth, function as biocontrol agents against diverse pathogenic fungi; and in general, increase abiotic and biotic stress tolerance in plants. Takahashi et al. [39] reported that of the underlying mechanism of the beneficial microbe-plant interaction involves complex cross-talk between numerous molecular pathways. Therefore, it has become evident that plant-microbe interactions can be used to support plant health [7, 40].

In the present study, four bacterial isolates (BPSAC77, BPSAC101, BPSAC121, and BPSAC147) were demonstrated to enhance the germination of tomato seeds. Inoculation of BPSAC147 increased the germination percentage to 100% compared to 90% in the untreated, control seeds. This finding is consistent with the result obtained by Rae-Hyun and Song [41], who reported that *Rhodopseudomonas* KL9 and *Rhodopseudomonas* BL6 increased the germination percentage of tomato seeds by 31.8% and 7.6%, relative to untreated, control seeds. Differences in the improvement of germination percentage may depend on the level of bacterial colonization in the seed, seed coat properties, and the amount of bacterial substances that can penetrate into the seed [42]. This finding is consistent with Lasudee et al. [21] who state that *Streptomyces thermocarboxydus* isolate S3 increased the germination percentage of mung bean seeds (95–98%) which was statistically higher than the control. Other environmental factors may also influence the growth-stimulating properties of some bacteria. For example, BPSAC147 may produce some phosphorus, enzymes, or phytohormone that stimulates tomato seeds to germinate.

In the present study, endophytic *Streptomyces thermocarboxydus* isolate BPSAC147 was obtained from root tissues of *Alstonia scholaris* L. and identified based on its 16S rRNA gene sequence. A phylogenetic tree indicated that isolate BPSAC147 was closely related to *Streptomyces thermocarboxydus* isolate DSM 44293 with a 72% bootstrap supported value, which is similar to a report by Passari et al. [30].

*Fusarium oxysporum* is a soil-borne pathogen that infects several different crop plants. The fungus enters the vascular system by infecting plant roots. Microorganisms can protect the infection site used by pathogens by producing specific nutrients (N, P, and S) and other compounds (antibiotics, lytic enzymes and phytohormone). Actinobacteria are known to be present in the rhizosphere of plants [28], and *Streptomyces* sp. are known to be endophytic in root tissues [43], where they can function as potential biocontrol agents and also modulate plant development. In the present study, *Streptomyces thermocarboxydus* isolate BPSAC147 was evaluated for its ability to promote plant growth and act as a biocontrol agent against *F*. *oxysporum* in tomato plants grown under greenhouse conditions. Similar results were reported by Goudjal et al. [44] who indicated that endophytic *Streptomyces* spp. isolate CA-2 and AA-2 significantly increased the shoot length, root length, and dry weight of tomato plants, relative to non-inoculated control plants. Isolate BPSAC147 also exhibited biocontrol activity against *F*. *oxysporum* in tomato plants which may be due to its potential to have significant plant growth promoting potential and secondary metabolites production as documented in our earlier study by Passari et al. [16]. Similar findings were reported by Goudjal et al. [45] who indicated that *Streptomyces* sp. isolate SNL2 could inhibit *Fusarium oxysporum* f. sp. *radicis lycopersici* infection of tomato plants. Moreover, Dias et al. [28] also reported that *Streptomyces* sp. isolate PM4 and PM5 significantly increased the shoot length (50.1 cm and 49.0 cm, respectively) and root length (31.0 cm and 29.2 cm, respectively) of tomato plants, relative to control plants (49.5 cm and 24.7 cm, respectively). Additionally, *Streptomyces caeruleatus* isolate ZL2 [46] and *Pseudomonas* sp. isolate S85 [47] were reported to decrease the level of root rot disease in tomato seedlings caused by *Fusarium* sp. Our study, however, is the first report on the ability of endophytic *Streptomyces thermocarboxydus* isolate BPSAC147 to exhibit PGP and biocontrol activity in tomato plants.

Our results indicated that *S*. *thermocarboxydus* isolate BPSAC147 induced plant growth promotion and exhibited biocontrol activity in tomato plants for up to 60 days. Results also indicated that chlorophyll a & b levels were highest in T1 plants at 60 days after the different treatments were initiated. This finding is somewhat similar to results reported by Dias et al. [28] indicating that *Streptomyces* sp. isolate PM5 produced a greater amount of chlorophyll a & b (0.14 μg/ml and 0.13 μg/ml, respectively) than control (0.11 μg/ml and 0.14 μg/ml, respectively) plants. Moreover, a higher level of total chlorophyll content (a + b) was found in T1 (34.649 μg/ml) plants than in T0 (15.701 μg/ml) plants. This finding is consistent with Dias et al. [28] who found that total chlorophyll content (1.66 μg/ml) in tomato plants inoculated with *Streptomyces* sp. isolate PM5 was significantly higher than the level in control (1.36 μg/ml) plants. Babu et al. [48] also reported that the rhizobacteria *B*. *subtilis* and *Azotobacter chroococcum* induced higher levels of chlorophyll content in tomato plants, relative to control plants. The inoculation of mung beans (*Vigna radiata*) with *S*. *thermocarboxydus* isolate S3 showed the significant increase in fresh weight, root length and total length in the presence of IAA production as state by Lasudee et al. [21].

Maxwell and Johnson [49] suggested that the photosynthetic efficiency of photosystem II (Fv/Fm) is a good indicator of stress levels in plants. In our study, photosynthetic parameters, including Fv/Fm, ETR, NPQ, qN, qP and yield rate were calculated in leaves obtained from plants subjected to the different treatments (T0, non-inoculated control plants; T1, plants inoculated with BPSAC147; T2, plants inoculated with BPSAC 147 + *F*. *oxysporum*, and T3, plants inoculated with *F*. *oxysporum*). T1 plants exhibited a significant increase in photosynthetic relative to T0 plants and in T2 plants relative to T3 plants. The Fv/Fm value represents the greatest quantum efficiency of PSII. We observed the highest photochemical quantum yield of PSII (Fv/Fm) in T1 plants, with a value of 0.812. This result is consistent with results reported by Samaniego-Gamez et al. [1] who indicated that Fv/Fm values were highest (3.6) in pepper plants inoculated with *Bacillus* sp. isolate M9. Fv/Fm represents the greatest efficiency of photosystem (PSII) and can be used to decrease QA, and as also serve as an indicator of plant photosynthetic performance [50]. T1 plants reached a higher maximum ETR (25.2) at 849 μmol photons m^-2^s^-1^, relative to T3 (13.56) plants. This finding is consistent with results reported by Samaniego-Gamez et al. [1] who indicated that the ETR of PSII exhibited a maximum level at 1150 μmol photons m^-2^s^-1^ in pepper plants inoculated with *Bacillus* sp. isolate M9, with a value of 45.4. Our results are also in agreement with the premise that the ETR of PSII is reduced by stress [51]. A significant increase in the ETR value is induced by PGPR. Melis [52] suggested that the quinone acceptor (Qa) is extremely oxidized and that its excitation energy is used in electron transport due to the increasing electron transport rate of PSII to reduce photodamage. Therefore, microbial inoculation of plants has the potential to enhance the rate of photosynthesis, as well as plant growth. In our study, T1 plants had a higher qP value (0.7307) than T0 plants (0.7) at 46 μmol photons m^-2^s^-1^. This finding is in complete agreement with Samaniego-Gamez et al. [1], who reported that pepper plants inoculated with *Bacillus* sp. isolate M9 and *Bacillus cereus* isolate K46 exhibited a higher qP than control plants (27% vs. 24%, respectively). qP represents the fraction of excitation captured by open traps and transferred to the chemical energy in the PSII system [53]. Higher qP indicate that more fluorescence is being quenched by the photochemical process [53]. Ranjan et al. [54] reported that immature fruits of *Jatropha curcas* (L.) trees exhibit a higher qP (0.46) as compared to mature and ripe fruits at 1200 μmol photons m^-2^s^-1^. In our study, T1 tomato plants exhibited higher NPQ (1.43) than in T0 control plants (1.37). Kumar et al. [55] indicated that PGPR can function in inducing growth in crop plants and impact the overall physiology of the entire plant resulting in higher yields in a variety of crops. It appears evident that microbes can augment abiotic and biotic stress tolerance in plants through the PGPR process [56].

GC-MS is useful method to detect and identify volatile organic compounds (VOCs) [57, 58, 59]. VOCs comprising of alcohols, ketones, esters, acetic acid, aldehydes, benzene groups, carboxylic groups, amide groups, and their derivatives have been reported to be produced by Actinobacteria [60], VOCs can also function as a signaling molecule. In the present study, 35 volatile compounds were detected by GC-MS analysis to be emitted from *Streptomyces thermocarboxydus* isolate BPSAC147. The relative abundance of 2, 6, 10-Dodecatrien-1-ol, 3, 7, 11-trimethyl was 17.959%, methylene diamine, N, N'-diacetyl- 6.043%), 1-Buten-3-yne, 2-methyl- 4.93%, and 4-(4'-hydroxyphenoxy) biphenyl 4.65%. Dodecane, 5,8-diethyl contains exhibited the largest percentage of peak area (3.102%). This finding is consistent with report by Guo et al. [61] who indicated that ether extracts of *Scapania verrucosa* Heeg and its endophytic fungus *Chaetomium fusiforme* contained dodecane, 5, 8-diethyl volatile compounds which have antifungal and antitumor activity. In addition, compounds such as methoxyacetic acid, 4-tetradecyl ester, and benzene also have antimicrobial properties against bacteria [62]. Oxirane, a hexyl compound identified in the present study, has also been reported to possess antimicrobial activity [63]. In their study, *Paracoccus pantotrophus* isolate FMR19 exhibited antimicrobial and antioxidant activity against bacterial pathogens and [MDROs] via the production of oxirane, hexyl compound.

In the present study, methanolic extracts of tomato leaves taken from plants subjected to each of the four treatments (T0, T1, T2, and T3) were collected to identify VOCs using GC-MS analysis. A total of 115 VOCs was detected from the four treatments. Two compounds (2, 4-di-tert-butylphenol and diethyl phthalate), which have antioxidant, antifungal and cytotoxic activity [64, 65, 66], were detected in T0 plants. Clonazepam and rhodopin compounds, which exhibit antioxidant, anticonvulsant, and pesticide and heavy metal tolerance activity [67, 68], were identified in T1 plants. The presence of these compounds in T1 plants suggests that they may function in promoting the growth of tomato plants and also serve as defense-related compounds against fungal pathogens. Methyl stearate, eicosapentaenoic acid, a TBDMS derivative, and ethyl iso-allocholate, which has anti-inflammatory, anticancer and anti-inflammatory activity [69, 70, 71], were identified in T2 plants. Lastly, 2, 4-di-tert-butylphenol, which has antioxidant properties, and astaxanthin, which has inflammation properties, were identified in T3 plants [64, 72].

The metabolite ethyl iso–allochaolate is highly unique upon the interaction of *S*. *thermocarboxydus* BPSAC147 with *F*. *oxysporum*, which is detected in the extract. Glp 1- Apelinendogenous peptide capable of binding the apelin receptor (APJ), which was originally described as an orphan G-protein-coupled receptor widely expressed in the tissues of human organs [73]. The apelin is so far not reported in plant systems which need further investigation. Oncontrary, mannnitol a compatible solute to modulate stress response was identified in T3 with *F*. *oxysporum* which might be due to the activation of Induced systemic resistance in tomato. In general, carotenoids, benzenoids and flavanoids are more predominant in the extracts of tomato leaves treated with *S*. *thermocarboxydus* BPSAC147 alone and combination of *S*. *thermocarboxydus* BPSAC147 with *F*. *oxysporum*.

## Supporting information

S1 Table Effect of potential endophytic isolate *Streptomyces thermocarboxydus* isolate BPSAC147 on shoot length, root length, plant weight and biocontrol effect of tomato seedlings after 60 days in triplicate. (XLS)

S2 Table Estimation of chlorophyll content in treated tomato plant in triplicate. (XLS)

S3 Table Effect of PGPR treatments on photosynthesis parameters in tomato plant. (XLS)

S4 Table Detection of volatile compounds in *Streptomyces thermocarboxydus* isolate BPSAC147. (PDF)

S5 Table Detection of volatile compounds in tomato leaf methanolic extracts in different treatments. (PDF)

S6 Table Data analysis of metabolites in tomato leaf extracts obtained from the different treatment groups using Metaboanalyst 4.0. (PDF)

S1 Fig Effect of PGPR and biocontrol efficiency in tomato plant under green house condition after 60 days. (PNG)

S2 Fig Detection of volatile compounds in tomato leaf methanolic extracts in control (T0) treatments. (PDF)

S3 Fig Detection of volatile compounds in tomato leaf methanolic extracts in T1 treatments. (PDF)

S4 Fig Detection of volatile compounds in tomato leaf methanolic extracts in T2 treatments. (PDF)

S5 Fig Detection of volatile compounds in tomato leaf methanolic extracts in T3 treatments. (PDF)
