# Supplementary material for: Enhancement of disease resistance, growth potential, and photosynthesis in tomato (Solanum lycopersicum) by inoculation with an endophytic actinobacterium, Streptomyces thermocarboxydus strain BPSAC147
Source: PLoS One. 2019 Jul 3;14(7):e0219014. doi: 10.1371/journal.pone.0219014 (PMC6608948; doi:10.1371/journal.pone.0219014)

S4 Table. Detection of Volatile Compounds in *Streptomyces thermocarboxydus* isolate BPSAC147

Sl. No.	RT	Compound Name	MW	Molecular structure	Function	Area %	Reference
1	1.764	1-Buten-3-yne, 2-methyl-	66	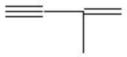	-	4.930	-
2	1.824	Methylene chloride	84	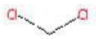	Insecticidal	3.023	-
3	2.004	Methylene diamine, N,N'-diacetyl-	130	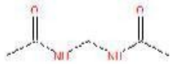	Antimicrobial	6.043	Zhao *et al.,* [1]
4	2.174	1H-Pyrazole, 3-methyl-	82	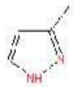	Antimicrobial	1.858	Arslan *et al.,* [2]
5	2.299	4-(2 Aminoethyl)benzenesulfonyl fluoride	200	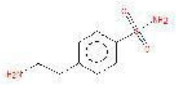	Serine protease inhibitor	1.537	Jiang *et al.,* [3]
6	2.334	1,5-Hexadiyne	78	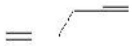	Antibacterial	1.579	Peng *et al.,* [4]
7	2.344	Benzene	78	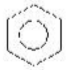	Antimicrobial	1.017	Sohrabi *et al.,* [5]
8	3.009	Pyridine, 1,2,3,6-tetrahydro-1,2-	83	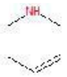	Antimicrobial	2.862	Mohamed *et al.,*

		dimethyl-					[6]
9	3.079	Pilocarpic acid	208	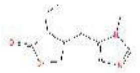	-	2.671	-
10	3.289	Resorcinol	110	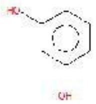	Antimicrobial	1.229	Rahangdale, [7]
11	3.549	2-n-Butylacrolein	112	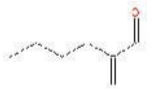	Antimicrobial	1.246	Sirdaarta *et al.,* [8]
12	3.97	Octane, 2-methyl-	128	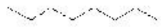	-	2.923	-
13	4.71	3,5-Octadiyne	106	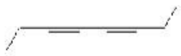	Antimicrobial & Antioxidant	2.498	Oloyede *et al.,* [9]
14	4.995	Oxirane, hexyl-	128	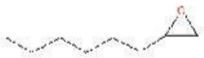	Antioxidant activity	0.920	Begum *et al.,* [10]
15	5.02	Benzeneethanamine, N-[(4-hydroxy) hydrocinnamoyl]	161	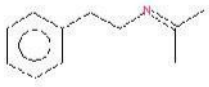	Antimicrobial	0.958	Harikrishnan *et al.,* [11]
16	5.05	Benzenepropanoyl bromide	212	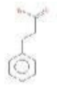	-	1.069	-

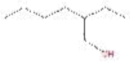

17	7.881	1-Hexanol, 2-ethyl-	130		Antimicrobial and Antioxidant	3.282	Zhao *et al.,* [1]
18	9.347	Nonane	128	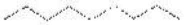	Antimicrobial	0.996	Morah *et al.,* [12]
19	12.148	Benzothiazole	135	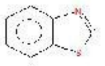	Antimicrobial, anticonvulsant, Anti inflammatory and antiviral	2.805	Ali and Siddiqui, [13]
20	15.244	Cedrene	204	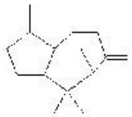	Antibacterial and repellent	0.894	Taran *et al.,* [14]
21	15.494	7-epi-trans-sesquisabinene hydrate	222	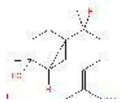	Antimicrobial	1.122	Hussein *et al.,* [15]
22	15.644	1H-3a,7-Methanoazulene, octahydro-3,8,8-trimethyl-6-methylene-, [3R-(3à,3aá,7á,8aà)] -	218	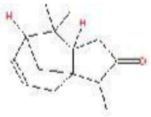	Antimicrobial	2.358	Saravanakumar and Gayathri, [16]

23	16.094	4,7-Methanoazulene, 1,2,3,4,5,6,7,8-octahydro-1,4,9,9-tetramethyl-, [1S-(1à,4à,7à)] -	204	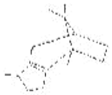	-	1.651	-
24	16.304	tert-Hexadecanethiol	258	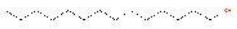	Antimicrobial	2.110	Parasuraman *et al.,* [17]
25	16.304	2-Piperidinone, N-[4-bromo-n-butyl]-	233	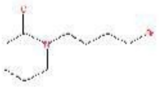	Antimicrobial and antioxidant	2.110	Arslan *et al.,* [2]
26	16.435	Decahydro-1,1,4a,5,6-pentamethylnaphthalene	208	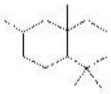	Antimicrobial	3.370	Peng *et al.,* [4]
27	16.78	(E)-á-Famesene	204	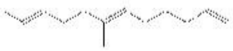	-	2.582	-
28	16.895	2,6,10-Dodecatrien-1-ol, 3,7,11-trimethyl-	280	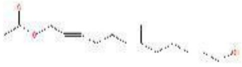	Antimicrobial	17.959	Liu *et al.,* [18]
29	18.395	Dodecane, 5,8-diethyl-	226	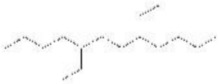	Antifungal and antibacterial	3.102	Guo *et al.,* [19]
30	19.156	N-Isobutylundeca-2(E)-en-8,10-diynamide	231	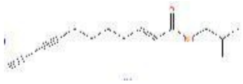	Antioxidants	1.452	Maimulyanti and Prihadi, [20]

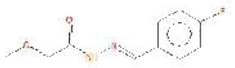

31	19.181	Methoxyacetic acid, 4-tetradecyl ester	210	Antimicrobial	0.905	Sohrabi *et al.,* [5]
32	20.041	Ethanol, 2-(tetradecyloxy)-	258	-	1.069	-
33	20.211	Copaene	204	Antioxidant and Antimicrobial	2.681	Phukan *et al.,* [21]
34	21.467	Pentadecane, 2,6,10,14-tetramethyl-	268	Antimicrobial and antioxidant	3.193	Zhao *et al.,* [1]
35	22.577	4-(4'-Hydroxyphenoxy)biphenyl	262	-	4.650	-

References

1. Zhao J, Jiang L, Tang X, Peng L, Li X, Zhao G, et al. Chemical Composition, Antimicrobial and Antioxidant Activities of the Flower Volatile Oils of *Fagopyrum esculentum*, *Fagopyrum tataricum* and *Fagopyrum Cymosum*. Molecules. 2018; 23(1): 182.
2. Arslan S, Loğoğlu E, Öktemer A. Antimicrobial activity studies on some piperidine and pyrrolidine substituted halogenobenzene derivatives. J Enzyme Inhib Med Chem. 2006; 21(2): 211-214.

3. Jiang YH, Shi Y, He YP, Du J, Li RS, Shi HJ, et al. Serine protease inhibitor 4-(2-aminoethyl) benzenesulfonyl fluoride hydrochloride (AEBSF) inhibits the rat embryo implantation in vivo and interferes with cell adhesion in vitro. Contraception. 2011;84(6):642-648.
4. Peng W, Lin Z, Wang L, Chang J, Gu F, Zhu X. Molecular characteristics of *Illicium verum* extractives to activate acquired immune response. Sau J Biol Sci. 2016; 23(3): 348-352.
5. Sohrabi M, Zhang L, Zhang K, Ahmetagic A, Wei MQ. Volatile organic compounds as novel markers for the detection of bacterial infections. Clin Microbiol: Open Acc. 2014; 3(3): 151.
6. Mohamed HM, EL-Wahab AHA, EL-Agrody AM, Bedair AH, Eid FA, Khafagy MM, et al. Synthesis and characterization of new diiodocoumarin derivatives with promising antimicrobial activities. Beilstein J Org Chem. 2011;7:1688.
7. Rahangdale SS. Synthesis, characterization and antimicrobial activity of resorcinol-melamine-formaldehyde resin. J Chem Pharm Res. 2012; 4(10):4451-4458.
8. Sirdaarta J, Matthews B, White A, Cock IE. GC-MS and LC-MS analysis of Kakadu plum fruit extracts displaying inhibitory activity against microbial triggers of multiple sclerosis. Pharmacog Commun. 2015; 5(2).
9. Oloyede GK. Toxicity, antimicrobial and antioxidant activities of methyl salicylate dominated essential oils of *Laportea aestuans* (Gaud). Arab J Chem. 2016; 9: S840-S845.
10. Begum I, Faridha R, Mohankumar M, Ramani K. "GC–MS Analysis of Bio-active Molecules Derived from *Paracoccus pantotrophus* FMR19 and the Antimicrobial Activity Against Bacterial Pathogens and MDROs." Ind J Microbiol. 2016; 56: 426-432.
11. Harikrishnan R, Kim MC, Kim JS, Balasundaram C, Jawahar S, Heo MS. Identification and antimicrobial activity of combined extract from *Azadirachta indica* and *Ocimum sanctum*. Isr J Aquacult = Bamid. 2010; 62(2):85-95.
12. Morah FN, Emehige EP, Mowang MM. Chemical composition and antimicrobial activity of *Nauclea latifolia* leaf essential oil. Int J Chem Biochem Sci. 2017; 11: 44-50.
13. Ali R, Siddiqui N. Biological Aspects of Emerging Benzothiazole: A Short Review. J Chem. 2013;2013.

14. Taran M, Ghasempour HR, Shirinpour E. Antimicrobial activity of essential oils of *Ferulago angulata* subsp. *carduchorum*. Jundishapur J Microbiol. 2010; 3(2): 10-14.
15. Hussein HM, Hameed IH, Ibraheem OA. Antimicrobial Activity and spectral chemical analysis of methanolic leaves extract of *Adiantum capillus-veneris* using GC-MS and FT-IR spectroscopy. Int J Pharmacog Phytochem Res. 2016; 8(3): 369-385.
16. Saravanakumar S, Gayathri K. Chemical characterization of *Vetiveria Zizanioideslinn* root. Int J Pharm Bio Sci. 2016; 7(4): 689-695.
17. Parasuraman S, Ren LY, Chuon BLC, Yee SWK, Qi TS, Ching JYS, et al. Phytochemical, antimicrobial and mast cell stabilizing activity of ethanolic extract of *Solanum trilobatum* Linn. leaves. Malays J Microbiol. 2016; 12(5):359-364.
18. Liu RH, Shen CJ, Wu HJ, Deng CJ, Liu SY. Characterisation of bio-oil from fast pyrolysis of rice husk in a fluidised bed reactor. J Energy Inst. 2011; 84: 73.

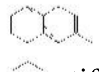

19. Guo L, Wu JZ, Han T, Cao T, Rahman K, Qin LP. Chemical composition, antifungal and antitumor properties of ether extracts of *Scapania verrucosa* Heeg. and its endophytic fungus *Chaetomium fusiforme*. Molecules. 2008; 13(9): 2114-2125.
20. Maimulyanti A, Prihadi AR. Chemical composition of essential oil and hexane extract and antioxidant activity of various extracts of *Acmella uliginosa* (Sw.) Cass flowers from Indonesia. Agric Nat Res. 2016; 50(4): 264-269.
21. Phukan H, Bora CR, Mitra PK. Phytochemical Screening and GC-MS Analysis of Methanolic leaf Extract of an Endemic Plant *Kayea assamica*. IOSR J Pharma Biol Sci. 2017; 12(5): 07-16.

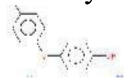

Supplement: S4 Table — (PDF) [file pone.0219014.s004.pdf]
