# Supplementary material for: Enhancement of disease resistance, growth potential, and photosynthesis in tomato (Solanum lycopersicum) by inoculation with an endophytic actinobacterium, Streptomyces thermocarboxydus strain BPSAC147
Source: PLoS One. 2019 Jul 3;14(7):e0219014. doi: 10.1371/journal.pone.0219014 (PMC6608948; doi:10.1371/journal.pone.0219014)
Supplement: S5 Table — (PDF) [file pone.0219014.s005.pdf]

S5 Table. Detection of Volatile Compounds in tomato leaf methanolic extracts in different treatments

Sl. No	RT	Compound Name	Molecular Structure	MW	Area%	Activity
			**Treatment T0**			
1	7.905	Benzene, 1,3-bis(1,1-dimethylethyl)-	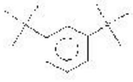	190	0.665	-
2	11.227	1-Dodecanol	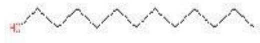	186	1.894	-
3	11.867	2,4-Di-tert-butylphenol	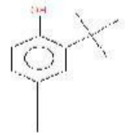	206	0.498	Antioxidant and Antifungal Properties [64, 65]
4	15.804	Dodecyl acrylate	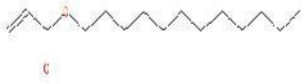	240	5.473	-
5	15.964	Hexadecane, 1,1-bis(dodecyloxy)-	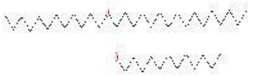	594	0.548	-
6	20.555	Hexadecanoic acid, methyl ester	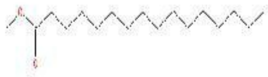	270	1.083	-

7	21.051	Diethyl phthalate	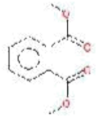	222	0.33	Cytotoxic activity [66]
8	21.196	Dasycarpidan-1-methanol, acetate (ester)	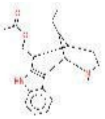	326	0.316	-
9	21.326	6-Hydroxy-powelline-N-nitroso-7-demethoxy-,aldehyde	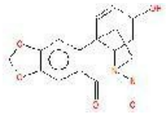	316	0.327	-
10	21.821	6-Amino-5-cyano-4-(5-cyano-2,4-dimethyl-1Hpyrrol-3-yl)-2-methyl-4H-pyran-3-carboxylic acid ethyl ester	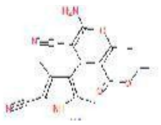	326	0.35	-
11	24.332	Heptadecanoic acid, 16-methyl-, methyl ester	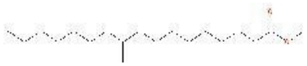	298	0.955	-
12	29.834	Benzoic acid, 4-methyl-2-trimethylsilyloxy-,trimethylsilyl ester	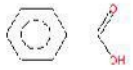	136	0.413	-

13	30.539	Hexa-t-butylselenatrisiletane	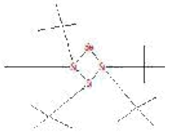	506	0.385	-
14	33.366	á-D-Glucopyranosiduronic acid, 3-(5-ethylhexahydro-2,4,6-trioxo-5-pyrimidinyl)-1,1-dimethylpropyl 2,3,4-tris-O-(trimethylsilyl)-, methyl ester	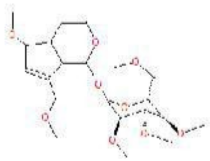	430	0.754	-
15	33.451	17-(1,5-Dimethylhexyl)-10,13-dimethyl-3-styryl hexa deca hydro cyclopenta [a] phenanthren-2-one	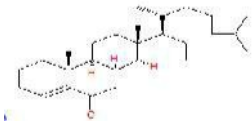	394	0.678	-
16	33.486	Bicyclo[5.3.0]decan-2-one, 9-(diphenyl methylene)-	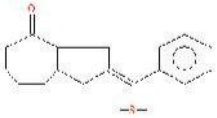	312	0.678	-
17	33.541	4-[4-(2-Methoxyphenyl)-1H-pyrazol-3-yl]benzene-1,3-diol	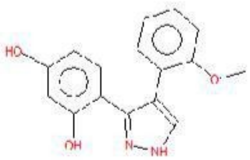	282	1.161	-

18	33.606	Methyl 9,11,13-octadecatrienoate, adduct with 1-methyl-1,3,4-triazolin-2,5-dione	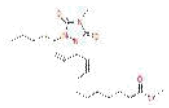	405	1.19	-
19	33.686	9,19-Cyclolanostan-3-ol, 24,24-epoxymethano-,acetate	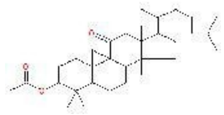	484	0.564	-
20	33.721	Acetic acid, 17-(4-hydroxy-5-methoxy-1,5-dimethylhexyl)-4,4,10,13,14-penta methyl-2,3,4,5,6,7, 10,11,12,13, 14, 15, 16,17-tetradeca hydro cyclopenta [a] phenanthryl ester	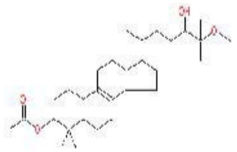	516	1.185	-
21	33.776	Glycidyl oleate	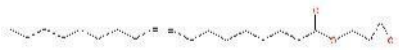	338	0.413	-
22	33.801	Phenanthrene, 9-dodecyltetradecahydro-	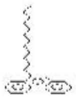	346	0.315	-

23	33.821	4-Acetyloxyimino-6,6-dimethyl-3-methylsulfanyl-4,5,6,7-tetrahydro-benzo[c]thiophene-1-carboxylic acid methyl ester	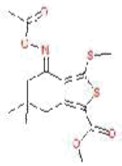	341	0.381	-
24	33.851	9,12-Octadecadienoic acid, 2-phenyl-1,3-dioxan-5-yl ester, cis-	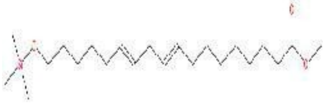	382	0.416	-
25	33.886	3-Desoxo-3,16-dihydroxy-12-desoxyphorbol 3,13,16,20-tetraacetate	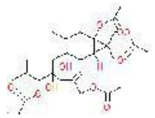	534	0.314	-
26	33.911	1H-Cyclopropa[3,4]benz[1,2-e]azulene-3-carboxaldehyde, 9a-(acetyloxy)-1a,1b, 4,4a,5,7a,7b,8,9,9a-decahydro-4a,7b-di hydroxy-1,1,6,8-tetramethyl-5,9-dioxo-, [1aR-(1aà,1bá, 4aá,7aà, 7bà,8à, 9aà)] -	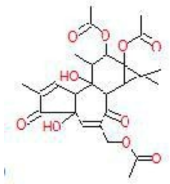	504	0.597	-

27	33.981	Spirost-8-en-11-one, 3-hydroxy-, (3á,5à,14á,20á,	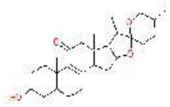	428	0.34	-
28	34.126	3,8,12-Tri-O-acetoxy-7-desoxyingol-7-one	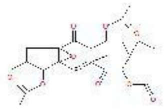	490	0.33	-
			**Treatment T1**			
1	5.549	Benzaldehyde, 4-methyl-	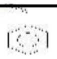	120	0.643	-
2	7.92	Benzene, 1,3-bis(1,1-dimethylethyl)-	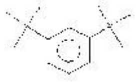	190	0.65	-
3	11.247	1-Dodecanol	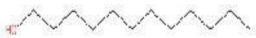	186	2.087	-
4	15.828	Dodecyl acrylate	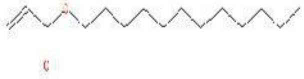	240	6.905	-
5	15.978	E-10-Dodecen-1-ol propionate	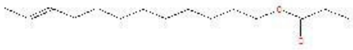	240	0.741	-
6	20.57	Pentadecanoic acid, 14-methyl-, methyl ester	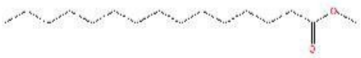	270	0.519	-
7	24.352	Methyl tetradecanoate	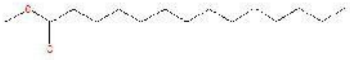	242	0.703	-

8	30.019	5,7,9(11)-Androstatriene, 3-hydroxy-17-oxo-	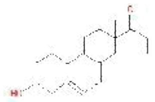	284	0.944	-
9	30.149	Benzo[h]quinoline, 2,4-dimethyl-	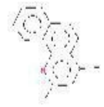	207	0.88	-
10	30.194	1,4,7-Androstatrien-3,17-dione	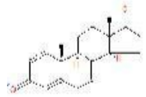	282	1.359	-
11	30.304	Docosanoic acid, 1,2,3-propanetriyl ester	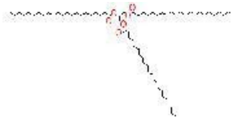	1058	0.738	-
12	30.364	.psi.,.psi.-Carotene, 1,1',2,2'-tetrahydro-1,1'-dimethoxy-	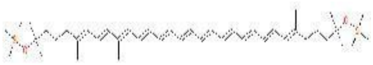	716	0.591	-
13	30.434	Estriol 16à-(á-D-glucuronide)	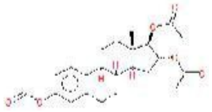	414	1.394	-
14	30.554	7aH-Cyclopenta[a] cyclopropa [f] cyclo undecene-2,4,7,7a,10,11-hexol, 1,1a, 2, 3, 4,4a,5,6,7,10,11,11adodecahydro-1,1,	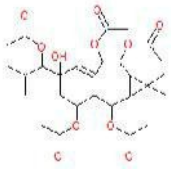	580	1.964	-

		3,6,9-pentamethyl-, 2,4,7,10,11-penta acetate				
15	30.579	7,8-Epoxylanostan-11-ol, 3-acetoxy-	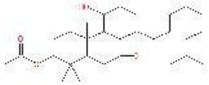	502	0.933	-
16	30.634	Clonazepam	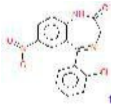	315	0.67	Antioxidant and Anticonvulsant [67]
17	30.699	17á-Acetoxy-1',1'-dicarboethoxy-1á,2á -dihydro-17à-methyl-3'H-cycloprop[1,2]-5à-androst-1-en-3-one	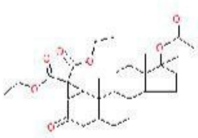	502	1.075	-
18	30.759	9,19-Cyclolanostan-3-ol, 24,24-epoxymethano-,acetate	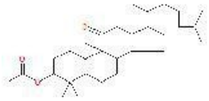	484	0.964	-
19	30.919	3-Isopropyl-6a,10b-dimethyl-8 - (2-oxo-2-phenyl ethyl)-dode cahydro-benzo [f]chromen-7-one	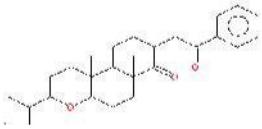	396	0.554	-
20	31.009	17-(1,5-Dimethylhexyl)-10,13-dimethyl-3-styryl hexadecahydro cyclopenta [a] phenanthren-2-one	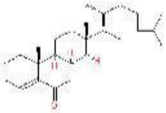	384	0.433	-

21	31.069	(+)-Prostaglandin F2à, 4TMS derivative	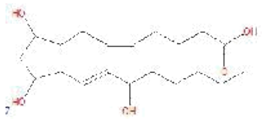	354	0.524	-
22	31.124	Strychane, 1-acetyl-20à-hydroxy-16-methylene-	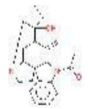	338	0.974	-
23	31.53	Pregn-5-ene-3,11-dione, 17, 20:20,21-bis [methylene bis (oxy)]-, cyclic 3-(1,2-ethane diyl acetal)	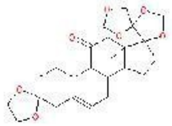	446	0.439	-
24	32.625	Phosphorothioic acid, O-(4,5-dichloro-2-methoxyphenyl) O,O-dimethyl ester	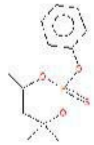	272	0.559	-
25	32.735	1,8-Dioxa-5-thiaoctane, 8-(9-bora bicycle [3.3.1]non-9-yl)-3-(9-bora bi cyclo[3.3.1]non-9-yloxy)-1-phenyl-	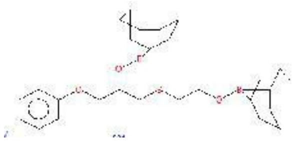	468	0.603	-
26	32.835	5,5,6-Exo-8,9,10-hexachlorocamphene	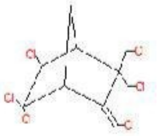	340	0.939	-
27	32.945	Rhodopin	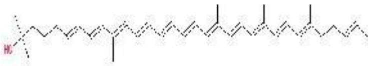	554	0.724	Pesticide and heavy

						metal tolerance [68]
28	33.035	10-Acetoxy-2-hydroxy-1,2,6a,6b, 9,9,12 ahepta methyl-1,3,4,5,6,6a,6b, 7,8,8a, 9, 10,11,12, 12a, 12b,13,14b-octadeca hydro -2H-picene-4acarboxylic acid, methyl ester	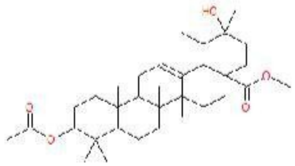	528	1.236	-
29	33.18	7,8-Epoxylanostan-11-ol, 3-acetoxy	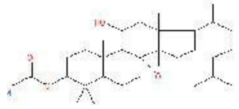	502	0.475	-
30	33.31	l-Methionine, N-(5-chlorovaleryl)-, methyl ester	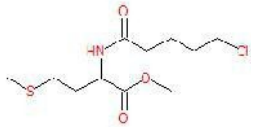	281	0.485	-
			**Treatment T2**			
1	11.247	1-Undecanol	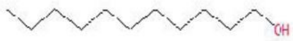	172	1.268	-
2	15.818	Dodecyl acrylate	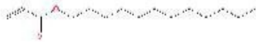	240	3.139	-
3	20.575	Hexadecanoic acid, methyl ester	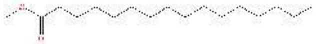	270	0.796	-

4	24.257	Ethyl iso-allocholate	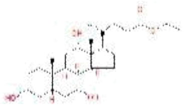	436	0.623	-
5	24.347	Methyl stearate	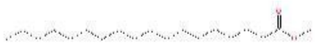	298	1.648	Anti-inflammatory activity [69]
6	29.419	Eicosapentaenoic Acid, TBDMS derivative	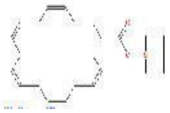	416	0.817	Anticancer activity [70]
7	29.639	Strychane, 1-acetyl-20à-hydroxy-16-methylene-	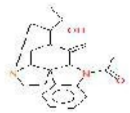	338	0.598	-
8	29.689	Oleic acid, 3-(octadecyloxy)propyl ester	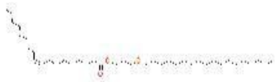	592	0.675	-
9	29.764	7aH-Cyclopenta[a]cyclopropa[f] cycloundecene-2,4,7,7a,10,11-hexol,1,1a,2,3,4,4a,5,6,7,10,11,11ado decahydro-1,1,3,6,9-pentamethyl-, 2,4,7,10,11-pentaacetate	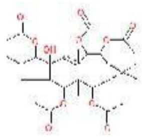	580	1.06	-

10	29.884	1-(2-Acetoxyethyl)-3,6-diazahomoadamantan-9-one oxime	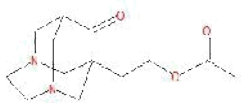	252	1.23	-
11	30.034	2,4,6-Decatrienoic acid, 1a,2,5,5a,6,9,10,10aoctahydro-5,5a-dihydroxy-4-(hydroxyl methyl)-1,7,9-trimethyl-1-[[(2-methyl-1-oxo-2-butenyl) oxy]methyl]-11-oxo-1H-2,8amethanocyclopenta[a]cyclopropa[e]cyclodecen-6-yl ester	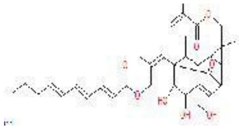	594	3.161	-
12	30.149	Tris(2-butoxyethyl) phosphate	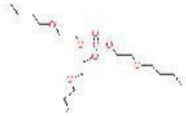	398	0.675	-
13	30.204	10-Acetoxy-2-hydroxy-1,2,6a, 6b, 9, 9,12 aheptamethyl-1,3, 4,5,6,6a,6b, 7,8,8a,9,10,11,12,12a,12b,13,14b-octadecahydro-2H-picene-4a carboxylic acid, methyl ester	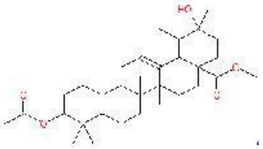	528	0.694	-

14	30.534	Oleic acid, eicosyl ester	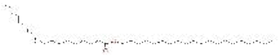	562	1.258	-
15	30.634	Docosahexaenoic acid, 1,2,3-propanetriyl ester	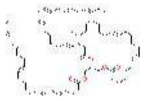	1022	0.672	-
16	30.749	3-Isopropyl-6a,7,10b-trimethyl-dodecahydrobenzo[f]chromene-7,8-dicarboxylic acid, dimethyl ester	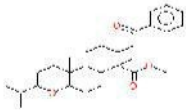	454	0.669	-
17	30.874	Octadecane, 1,1'-[1,3-propanediylbis(oxy)]bis-	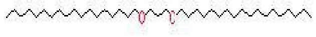	580	1.217	-
18	30.904	17-Pentatriacontene	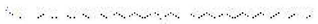	490	0.623	-
19	31.054	2-Nonadecanone 2,4-dinitrophenylhydrazine	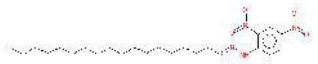	462	1.07	-
20	31.149	N,N'-Trimethylenebis[s-3-aminopropylthiosulfuric acid]	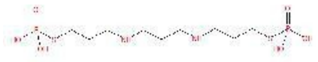	382	1.251	-
21	31.174	9,12,15-Octadecatrienoic acid, 2-phenyl-1,3-dioxan-5-yl ester	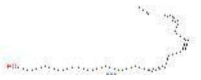	264	1.053	-

22	31.33	9-Desoxo-9-x-acetoxy-3,8,12-tri-O-acetylingol	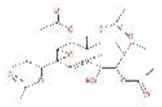	536	0.836	-
23	31.79	Propanoic acid, 2-(3-acetoxy-4,4,14-trimethylandrost-8-en-17-yl)-	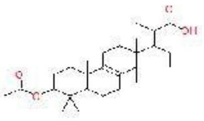	430	0.726	-
24	32.685	10,13-Octadecadiynoic acid, methyl ester	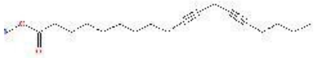	290	0.808	-
25	32.78	Cholestan-3-ol, 5-chloro-6-nitro-, (3á,5à,6á) -	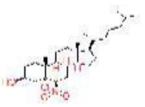	467	1.64	-
26	32.845	Testolactone	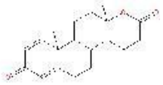	300	1.041	-
27	34.456	Ethyl iso-allocholate	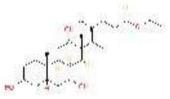	436	0.735	Anti-inflammatory activity [71]
28	34.526	Strychane, 1-acetyl-20à-hydroxy-16-methylene-	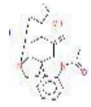	338	0.708	-
29	34.636	9à-Fluoro-17à-methyl-5à-Androstan-3á,11á,17á -triol	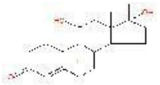	336	0.68	-

			**Treatment T3**			
1	3.063	2-Myristynoyl pantetheine	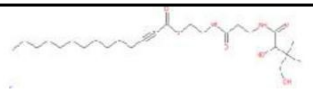	484	0.345	-
2	3.469	Furane-3,4(2H,5H)-dione, 2,2-dimethyl-5-spirocyclohexane-,dioxime	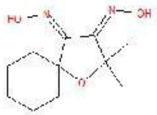	226	0.463	-
3	3.984	Glafenin	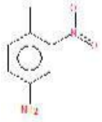	152	0.296	-
4	4.224	13,14-Epoxyursan-3-ol, acetate	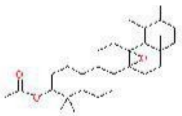	472	0.404	-
5	4.409	1,4-Pentanediamine	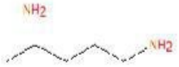	102	0.263	-
6	5.559	Benzaldehyde, 4-methyl-	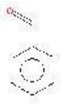	120	0.94	-
7	7.915	Benzene, 1,3-bis(1,1-dimethylethyl)-	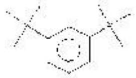	190	0.719	-

8	11.257	Cyclotetradecane	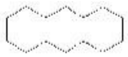	196	2.574	-
9	11.897	2,4-Di-tert-butylphenol	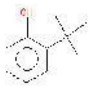	206	0.494	Antioxidant Properties [64]
10	13.698	D-Mannitol, 1,2:3,4:5,6-tris-O-(1-methylethylidene)-	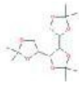	302	0.349	-
11	15.828	Dodecyl acrylate	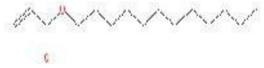	240	8.013	-
12	15.984	3-Propionyloxypentadecane	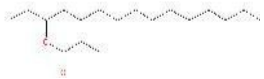	284	0.612	-
13	20.58	Hexadecanoic acid, methyl ester	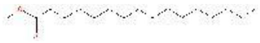	270	0.988	-
14	21.601	2-Nonadecanone 2,4-dinitrophenylhydrazine	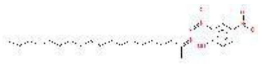	462	0.239	-
15	24.012	9-Octadecene, 1,1'-[1,2-ethanediylbis(oxy)]bis-, (Z,Z)-	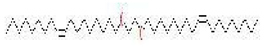	562	0.254	-
16	24.347	Heptadecanoic acid, 16-methyl-, methyl ester	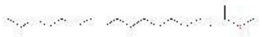	298	1.124	-

17	27.958	(4,4-Diphenyl-butyl)-(3-phenyl-piperidin-4-yl)-amine	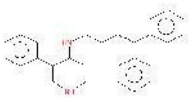	384	0.265	-
18	28.553	Octadecane, 1-chloro-	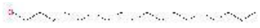	288	0.265	-
19	29.854	17-Pentatriacontene		490	0.351	-
20	30.064	3-Isopropyl-6a,10b-dimethyl-8-(2-oxo-2-phenylethyl)-dodecahydro-benzo[f] chromen-7-one	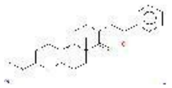	396	0.298	-
21	30.699	2-Chloroadenosine	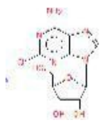	301	0.386	-
22	31.155	7,8-Epoxylanostan-11-ol, 3-acetoxy-	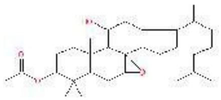	502	0.323	-
23	31.59	Glafenin	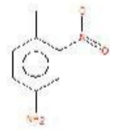	152	0.244	-
24	31.635	6-Azacholest-4-en-7-one, 6-benzyl-3à-hydroxy	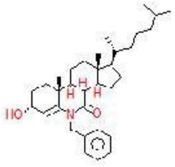	491	0.295	-

25	31.795	2,4-Di-tert-butylthiophenol	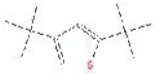	196	0.342	-
26	33.08	Astaxanthin	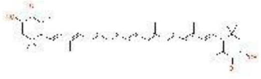	596	0.251	Inflammation [72]
27	33.596	3,8,12-Tri-O-acetoxy-7-desoxyingol-7-one	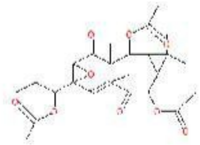	490	0.254	-
28	34.171	Distearin	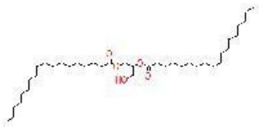	624	0.28	-
