# Supplementary material for: Enhancement of disease resistance, growth potential, and photosynthesis in tomato (Solanum lycopersicum) by inoculation with an endophytic actinobacterium, Streptomyces thermocarboxydus strain BPSAC147
Source: PLoS One. 2019 Jul 3;14(7):e0219014. doi: 10.1371/journal.pone.0219014 (PMC6608948; doi:10.1371/journal.pone.0219014)
Supplement: S6 Table — (PDF) [file pone.0219014.s006.pdf]

Metabolomic Data Analysis with MetaboAnalyst 4.0

Name: guest8911470831900528833

April 16, 2019

1 Data Processing and Normalization

1.1 Reading and Processing the Raw Data

MetaboAnalyst accepts a variety of data types generated in metabolomic studies, including compound concentration data, binned NMR/MS spectra data, NMR/MS peak list data, as well as MS spectra (NetCDF, mzXML, mzDATA). Users need to specify the data types when uploading their data in order for MetaboAnalyst to select the correct algorithm to process them. Table 1 summarizes the result of the data processing steps.

1.1.1 Reading MS Peak List and Intensities Data

MS peak list and intensities data should be uploaded as one zip file. It contains subfoulders with one for each group. Each folder contains peak list files, one per spectrum. The MS peak list format is either a two-column (mass and intensities) or three-column (mass, retention time, and intensities) comma separated values. The first line is assumed to be column labels. The files should be saved in .csv format. For paired analysis, users need to upload separately a text file specifying the paired information. Each pair is indicated by their sample names seperated by a colon ":" with one pair per line.

The uploaded files are peak lists and intensities data. A total of 12 samples were found. These samples contain a total of 357 peaks, with an average of 29.8 peaks per sample

1.1.2 Peak Matching and Alignment

Peaks need to be matched across samples in order to be compared. For two-column data, the program matches peaks by their m/z values. For three-column data, the program will further group peaks based on their retention time. During the process, mz and rt of each peak will be changed to their group median values. If a sample has more than one peak in a group, they will be replaced by their sum. Some peaks are excluded if they appear in less than half of both classes. The aligned peaks are reorganized into a single data matrix for further analysis. The name of the parent folder is used as class label for each sample.

A total of 76 peak groups were formed. Peaks of the same group were summed if they are from one sample. Peaks appearing in less than half of all samples in each group were ignored.

1.1.3 Data Integrity Check

Before data analysis, a data integrity check is performed to make sure that all the necessary information has been collected. The class labels must be present and contain only two classes. If samples are paired, the class label must be from -n/2 to -1 for one group, and 1 to n/2 for the other group (n is the sample number and must be an even number). Class labels with same absolute value are assumed to be pairs.

Compound concentration or peak intensity values should all be non-negative numbers. By default, all missing values, zeros and negative values will be replaced by the half of the minimum positive value found within the data (see next section)

1.1.4 Missing value imputations

Too many zeroes or missing values will cause difficulties for downstream analysis. MetaboAnalyst offers several different methods for this purpose. The default method replaces all the missing and zero values with a small values (the half of the minimum positive values in the original data) assuming to be the detection limit. The assumption of this approach is that most missing values are caused by low abundance metabolites (i.e.below the detection limit). In addition, since zero values may cause problem for data normalization (i.e. log), they are also replaced with this small value. User can also specify other methods, such as replace by mean/median, or use K-Nearest Neighbours (KNN), Probabilistic PCA (PPCA), Bayesian PCA (BPCA) method, Singular Value Decomposition (SVD) method to impute the missing values [1]. Please choose the one that is the most appropriate for your data.

Zero or missing variables were replaced with a small value: 0.127

1.1.5 Data Filtering

The purpose of the data filtering is to identify and remove variables that are unlikely to be of use when modeling the data. No phenotype information are used in the filtering process, so the result can be used with any downstream analysis. This step can usually improves the results. Data filter is strongly recommended for datasets with large number of variables (> 250) datasets contain much noise (i.e.chemometrics data). Filtering can usually improve your results[2].

For data with number of variables < 250, this step will reduce 5% of variables; For variable number between 250 and 500, 10% of variables will be removed; For variable number bwteen 500 and 1000, 25% of variables will be removed; And 40% of variabled will be removed for data with over 1000 variables. The None option is only for less than 5000 features. Over that, if you choose None, the IQR filter will still be applied. In addition, the maximum allowed number of variables is ***10000***

No data filtering was performed.

Table 1: Summary of data processing results

	Peaks (raw)	Missing/Zero	Peaks (processed)
C60 1	29	48	19
C60 2	29	48	19
C60	29	48	19
T160 1	30	50	19
T160 2	30	50	19
T160	30	50	19
T260 1	30	51	19
T260 2	30	51	19
T260	30	51	19
T360	30	50	19
T3601	30	50	19
T3602	30	50	19

[1]Stacklies W, Redestig H, Scholz M, Walther D, Selbig J. *pcaMethods: a bioconductor package, providing PCA methods for incomplete data.*, Bioinformatics 2007 23(9):1164-1167

[2]Hackstadt AJ, Hess AM.*Filtering for increased power for microarray data analysis*, BMC Bioinformatics. 2009; 10: 11.

1.2 Data Normalization

The data is stored as a table with one sample per row and one variable (bin/peak/metabolite) per column. The normalization procedures implemented below are grouped into four categories. Sample specific normalization allows users to manually adjust concentrations based on biological inputs (i.e. volume, mass); row-wise normalization allows general-purpose adjustment for differences among samples; data transformation and scaling are two different approaches to make features more comparable. You can use one or combine both to achieve better results.

The normalization consists of the following options:

1. Row-wise procedures:
   - Sample specific normalization (i.e. normalize by dry weight, volume)
   - Normalization by the sum
   - Normalization by the sample median
   - Normalization by a reference sample (probabilistic quotient normalization)[3]
   - Normalization by a pooled or average sample from a particular group
   - Normalization by a reference feature (i.e. creatinine, internal control)
   - Quantile normalization
2. Data transformation :
   - Generalized log transformation (glog 2)
   - Cube root transformation
3. Data scaling:
   - Mean centering (mean-centered only)
   - Auto scaling (mean-centered and divided by standard deviation of each variable)
   - Pareto scaling (mean-centered and divided by the square root of standard deviation of each variable)
   - Range scaling (mean-centered and divided by the value range of each variable)

Figure 1 shows the effects before and after normalization.

[3]Dieterle F, Ross A, Schlotterbeck G, Senn H. *Probabilistic quotient normalization as robust method to account for dilution of complex biological mixtures. Application in 1H NMR metabonomics*, 2006, Anal Chem 78 (13);4281 - 4290

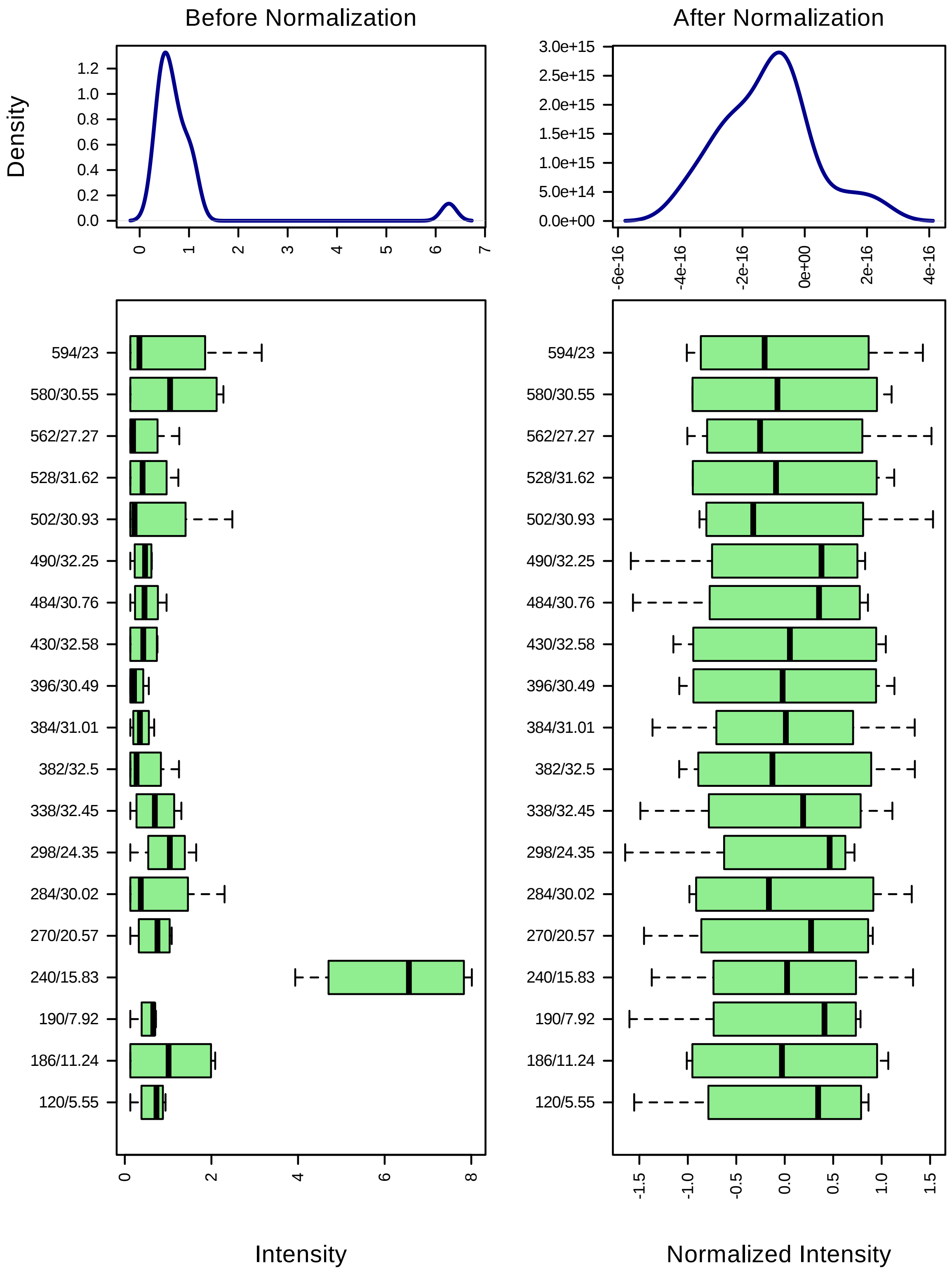

Figure 1: Box plots and kernel density plots before and after normalization. The boxplots show at most 50 features due to space limit. The density plots are based on all samples. Selected methods : Row-wise normalization: Normalization to constant sum; Data transformation: Log Normalization; Data scaling: Autoscaling.

2 Statistical and Machine Learning Data Analysis

MetaboAnalyst offers a variety of methods commonly used in metabolomic data analyses. They include:

1. Univariate analysis methods:
    - Fold Change Analysis
    - T-tests
    - Volcano Plot
    - One-way ANOVA and post-hoc analysis
    - Correlation analysis
2. Multivariate analysis methods:
    - Principal Component Analysis (PCA)
    - Partial Least Squares - Discriminant Analysis (PLS-DA)
3. Robust Feature Selection Methods in microarray studies
    - Significance Analysis of Microarray (SAM)
    - Empirical Bayesian Analysis of Microarray (EBAM)
4. Clustering Analysis
    - Hierarchical Clustering
        - Dendrogram
        - Heatmap
    - Partitional Clustering
        - K-means Clustering
        - Self-Organizing Map (SOM)
5. Supervised Classification and Feature Selection methods
    - Random Forest
    - Support Vector Machine (SVM)

`Please note: some advanced methods are available only for two-group sample analyais.`

2.1 Principal Component Analysis (PCA)

PCA is an unsupervised method aiming to find the directions that best explain the variance in a data set (X) without referring to class labels (Y). The data are summarized into much fewer variables called *scores* which are weighted average of the original variables. The weighting profiles are called *loadings*. The PCA analysis is performed using the `prcomp` package. The calculation is based on singular value decomposition.

The Rscript `chemometrics.R` is required. Figure 2 is pairwise score plots providing an overview of the various seperation patterns among the most significant PCs; Figure 3 is the scree plot showing the variances explained by the selected PCs; Figure 4 shows the 2-D scores plot between selected PCs; Figure 5 shows the 3-D scores plot between selected PCs; Figure 6 shows the loadings plot between the selected PCs; Figure 7 shows the biplot between the selected PCs.

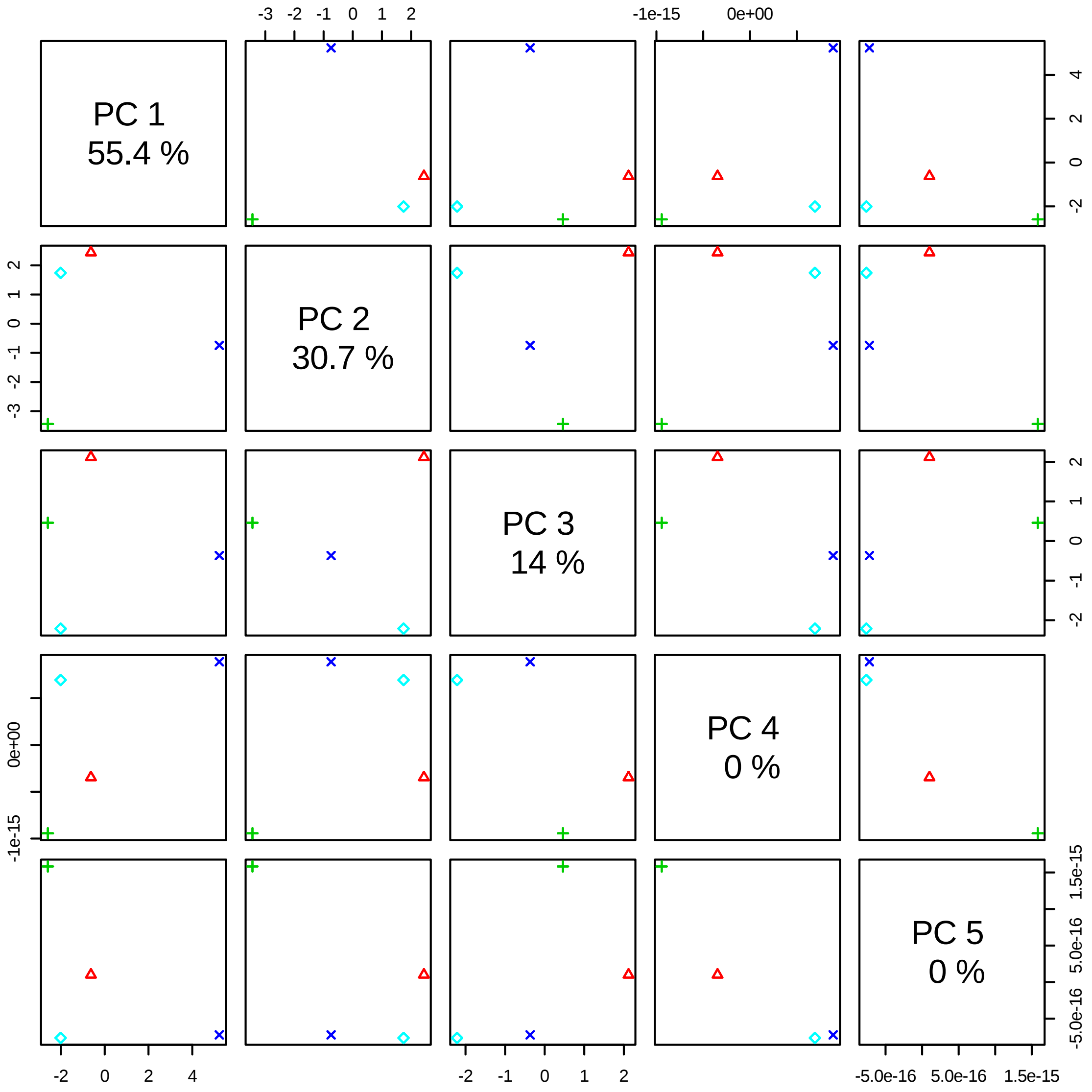

Figure 2: Pairwise score plots between the selected PCs. The explained variance of each PC is shown in the corresponding diagonal cell.

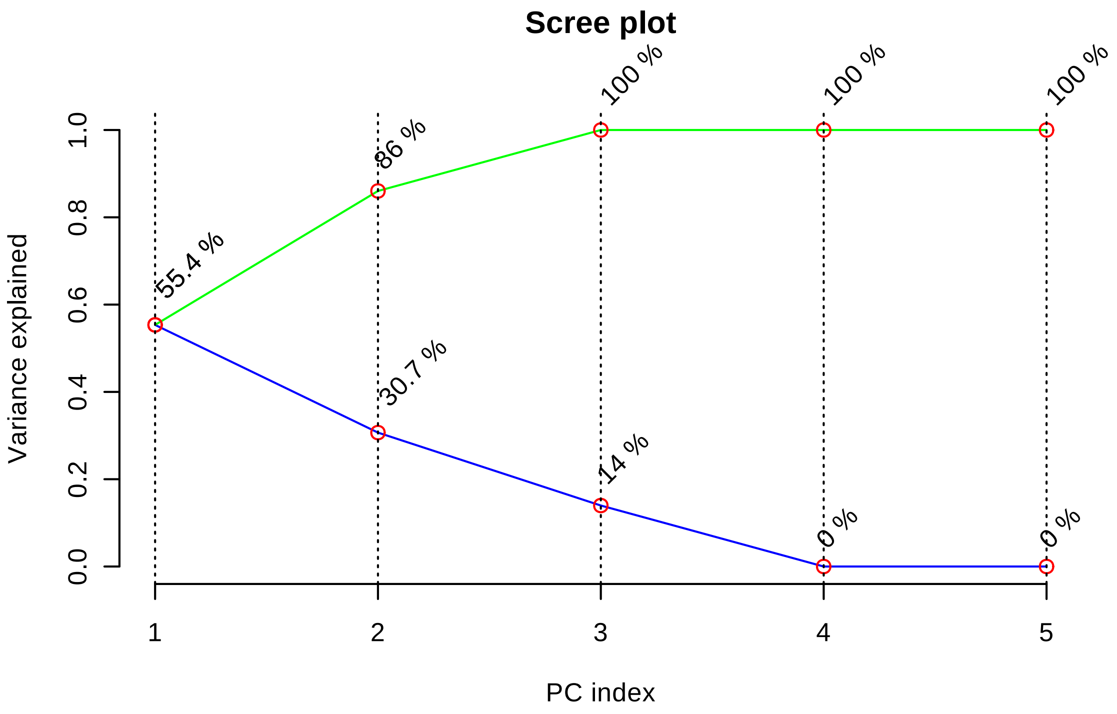

Figure 3: Scree plot shows the variance explained by PCs. The green line on top shows the accumulated variance explained; the blue line underneath shows the variance explained by individual PC.

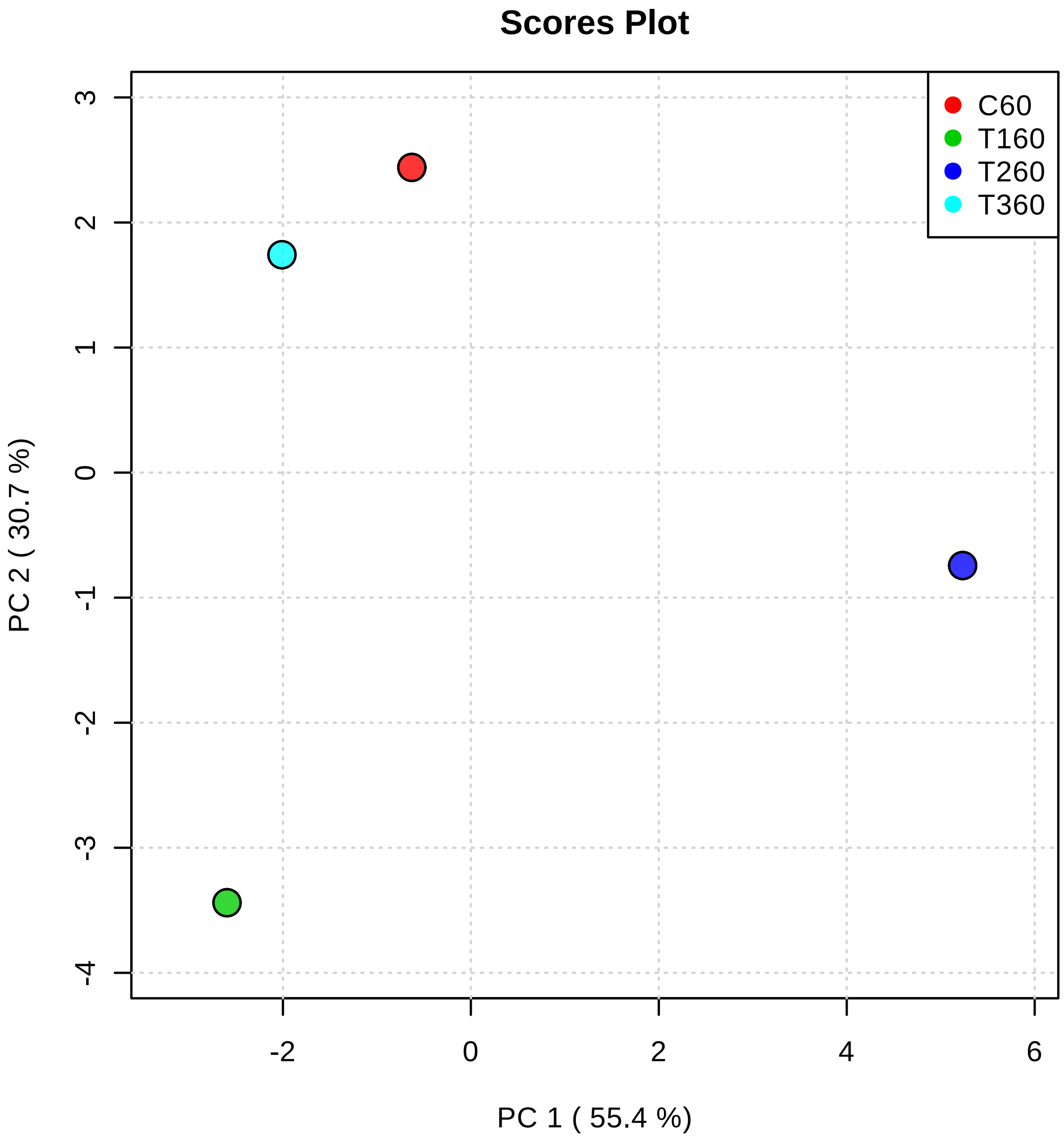

Figure 4: Scores plot between the selected PCs. The explained variances are shown in brackets.

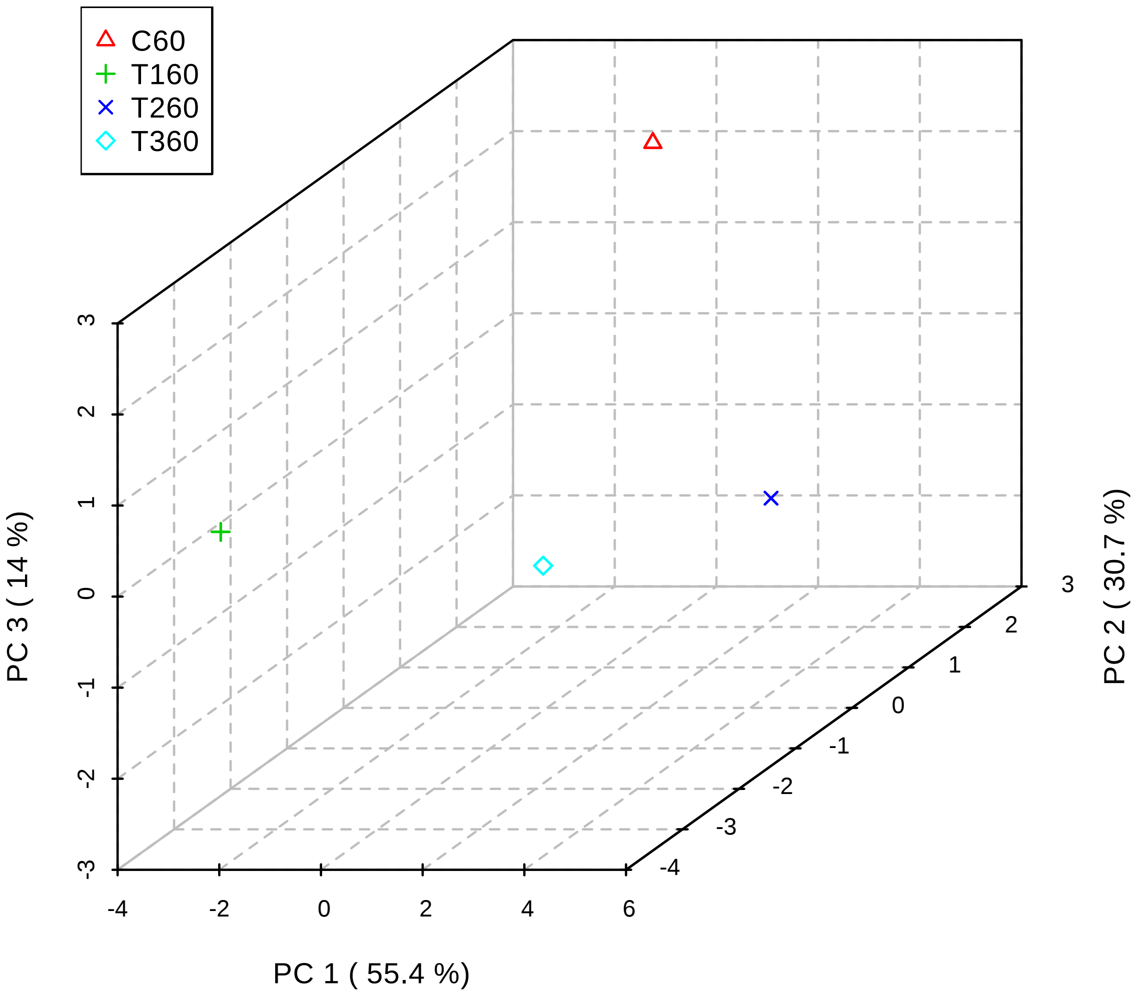

Figure 5: 3D score plot between the selected PCs. The explained variances are shown in brackets.

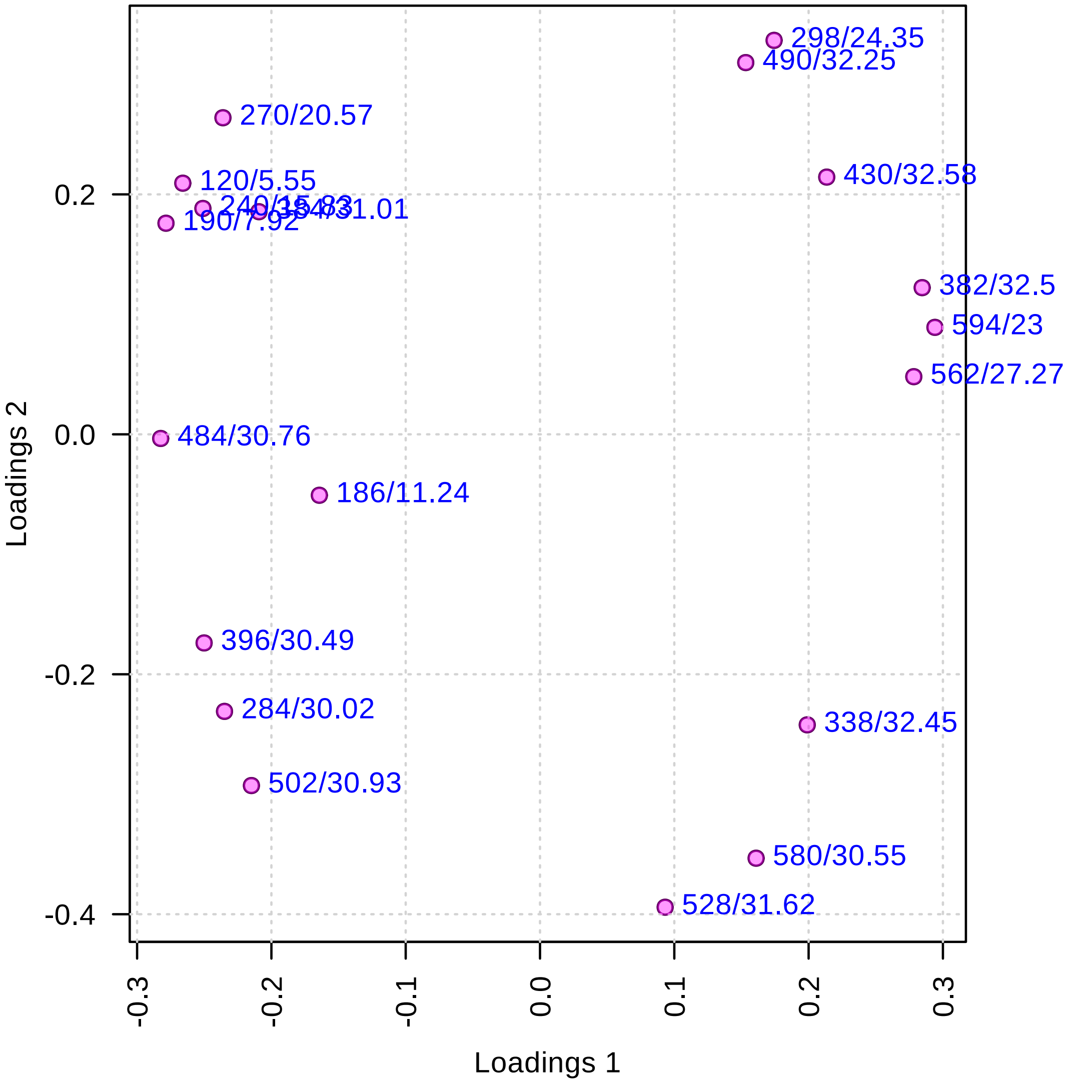

Figure 6: Loadings plot for the selected PCs.

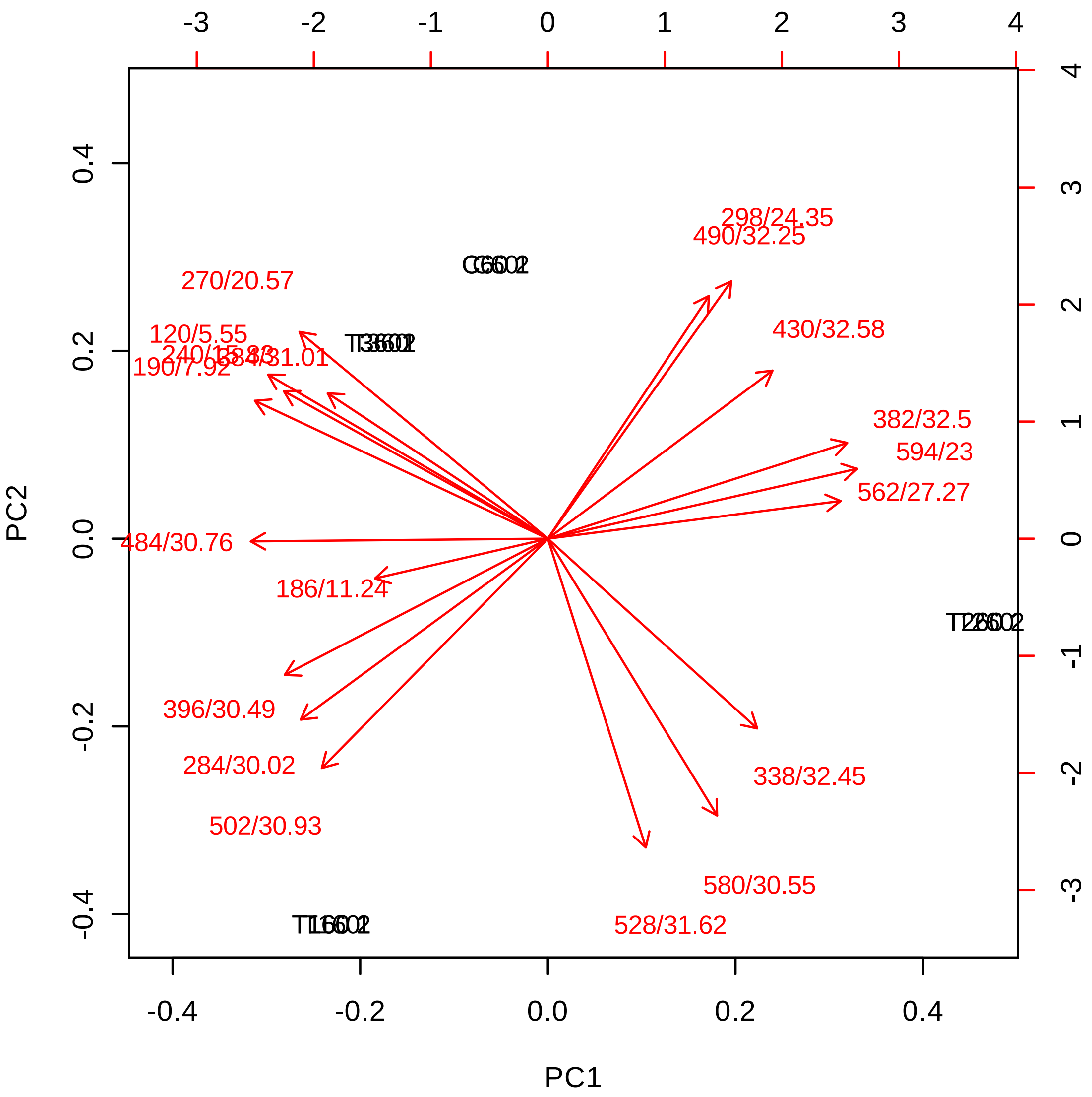

Figure 7: PCA biplot between the selected PCs. Note, you may want to test different centering and scaling normalization methods for the biplot to be displayed properly.

2.2 Hierarchical Clustering

In (agglomerative) hierarchical cluster analysis, each sample begins as a separate cluster and the algorithm proceeds to combine them until all samples belong to one cluster. Two parameters need to be considered when performing hierarchical clustering. The first one is similarity measure - Euclidean distance, Pearson's correlation, Spearman's rank correlation. The other parameter is clustering algorithms, including average linkage (clustering uses the centroids of the observations), complete linkage (clustering uses the farthest pair of observations between the two groups), single linkage (clustering uses the closest pair of observations) and Ward's linkage (clustering to minimize the sum of squares of any two clusters). Heatmap is often presented as a visual aid in addition to the dendrogram.

Hierachical clustering is performed with the `hclust` function in package `stat`. Figure 8 shows the clustering result in the form of a dendrogram. Figure 9 shows the clustering result in the form of a heatmap.

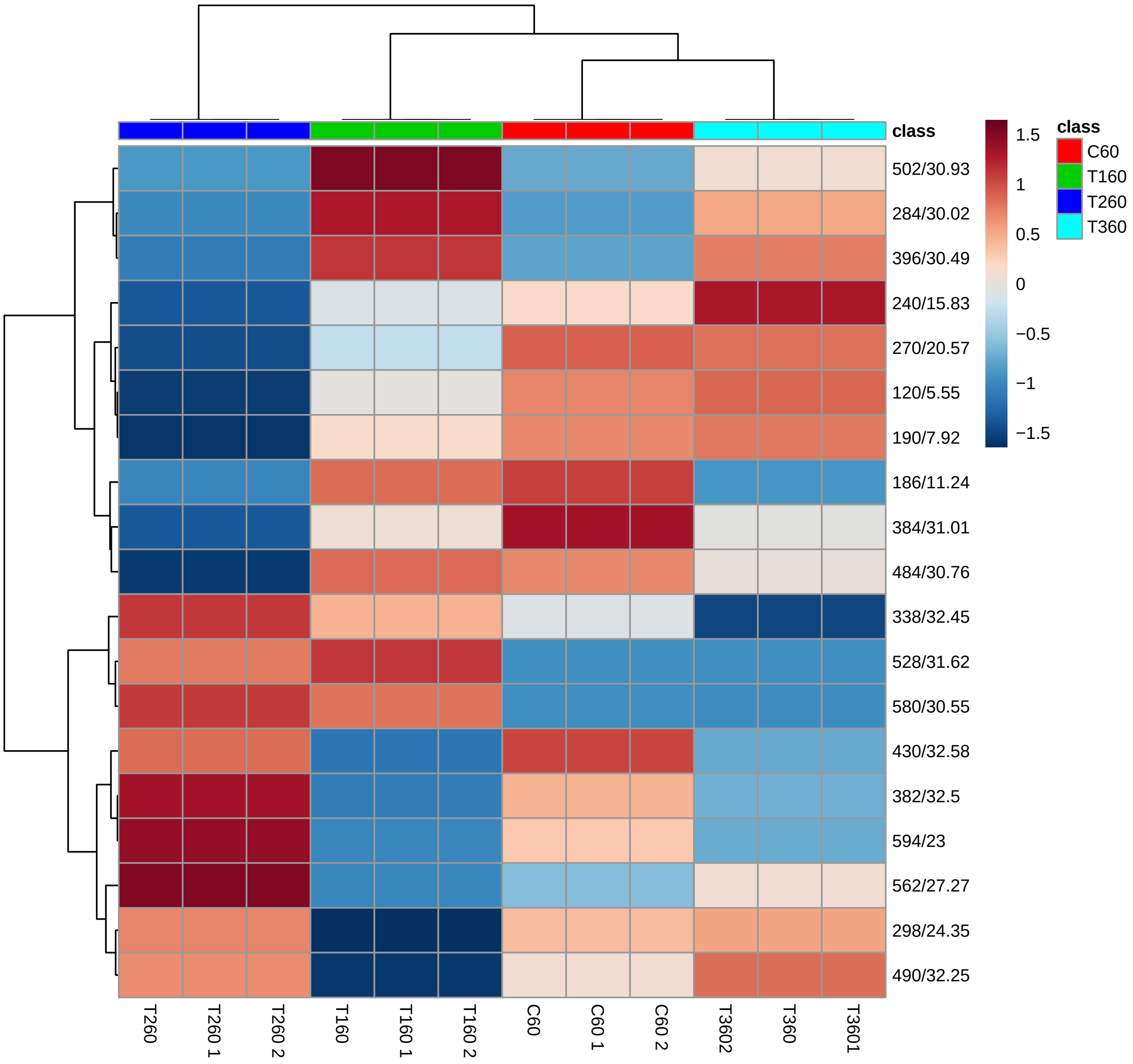

Figure 8: Clustering result shown as heatmap (distance measure using `euclidean`, and clustering algorithm using `ward.D`).

3 Appendix: R Command History

```
 [1] "mSet<-InitDataObjects(\"mspeak\", \"stat\", FALSE)"
 [2] "UnzipUploadedFile(\"Replacing_with_your_file_path\", \"upload\", T);"
 [3] "mSet<-Read.PeakList(mSet, \"upload\");"
 [4] "mSet<-GroupPeakList(mSet, 0.025,30.0);\nSetPeakList.GroupValues(mSet)"
 [5] "mSet<-SanityCheckData(mSet)"
 [6] "mSet<-ReplaceMin(mSet);"
 [7] "mSet<-PreparePrenormData(mSet)"
 [8] "mSet<-Normalization(mSet, \"SumNorm\", \"LogNorm\", \"AutoNorm\", ratio=FALSE, ratioNum=20)"
 [9] "mSet<-PlotNormSummary(mSet, \"norm_0_\", \"png\", 72, width=NA)"
[10] "mSet<-PlotSampleNormSummary(mSet, \"snorm_0_\", \"png\", 72, width=NA)"
[11] "mSet<-SaveTransformedData(mSet)"
[12] "mSet<-PreparePDFReport(mSet, \"guest8911470831900528833\")\n"
[13] "mSet<-PlotHeatMap(mSet, \"heatmap_0_\", \"png\", 72, width=NA, \"norm\", \"row\", \"euclidean\
[14] "mSet<-PCA.Anal(mSet)"
[15] "mSet<-PlotPCAPairSummary(mSet, \"pca_pair_0_\", \"png\", 72, width=NA, 5)"
[16] "mSet<-PlotPCAScree(mSet, \"pca_scree_0_\", \"png\", 72, width=NA, 5)"
[17] "mSet<-PlotPCA2DScore(mSet, \"pca_score2d_0_\", \"png\", 72, width=NA, 1,2,0.95,0,0)"
[18] "mSet<-PlotPCALoading(mSet, \"pca_loading_0_\", \"png\", 72, width=NA, 1,2);"
[19] "mSet<-PlotPCABiplot(mSet, \"pca_biplot_0_\", \"png\", 72, width=NA, 1,2)"
[20] "mSet<-PlotPCA3DScoreImg(mSet, \"pca_score3d_0_\", \"png\", 72, width=NA, 1,2,3, 40)"
[21] "mSet<-PlotPCA3DLoading(mSet, \"pca_scoreloading_0_\", \"json\", 1,2,3)"
[22] "mSet<-PreparePDFReport(mSet, \"guest8911470831900528833\")\n"
```

The report was generated on Tue Apr 16 10:37:32 2019 with R version 3.5.1 (2018-07-02).
