# Supplementary figures and images for: Enhancement of disease resistance, growth potential, and photosynthesis in tomato (Solanum lycopersicum) by inoculation with an endophytic actinobacterium, Streptomyces thermocarboxydus strain BPSAC147
Source: PLoS One. 2019 Jul 3;14(7):e0219014. doi: 10.1371/journal.pone.0219014 (PMC6608948; doi:10.1371/journal.pone.0219014)

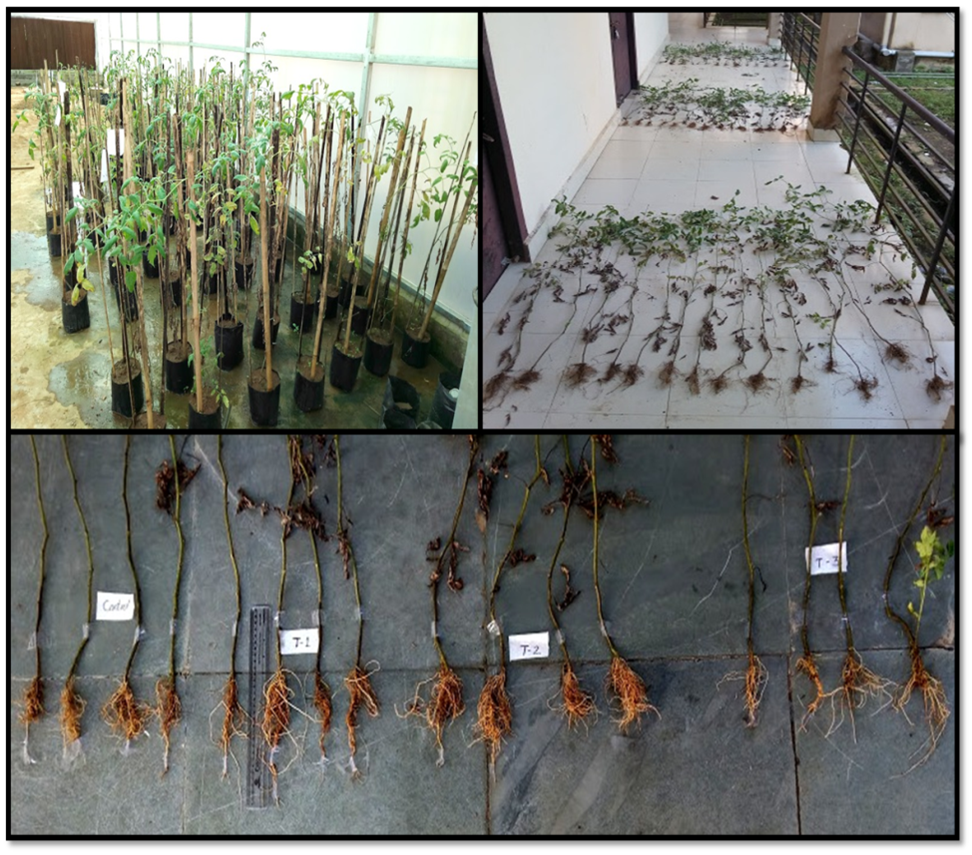

Supplement: S1 Fig — (PNG) [file pone.0219014.s007.png]
