# Supplementary material for: Enhancement of disease resistance, growth potential, and photosynthesis in tomato (Solanum lycopersicum) by inoculation with an endophytic actinobacterium, Streptomyces thermocarboxydus strain BPSAC147
Source: PLoS One. 2019 Jul 3;14(7):e0219014. doi: 10.1371/journal.pone.0219014 (PMC6608948; doi:10.1371/journal.pone.0219014)

TAMILNADU AGRICULTURAL UNIVERSITY - AGRICULTURAL MICROBIOLOGY

INSTRUMENT: PERKIN ELMER CLARUS SQ8C COLOUMN: DB-5 MS CAPILARY STANDARD NON - POLARCOLOUMN
INJECTION VOL: 1 MICRO LITER DIMENSION: 30Mts, ID: 0.25 mm, FILM: 0.25 IM CARRIER GAS: He
SAMPLE ID : C160

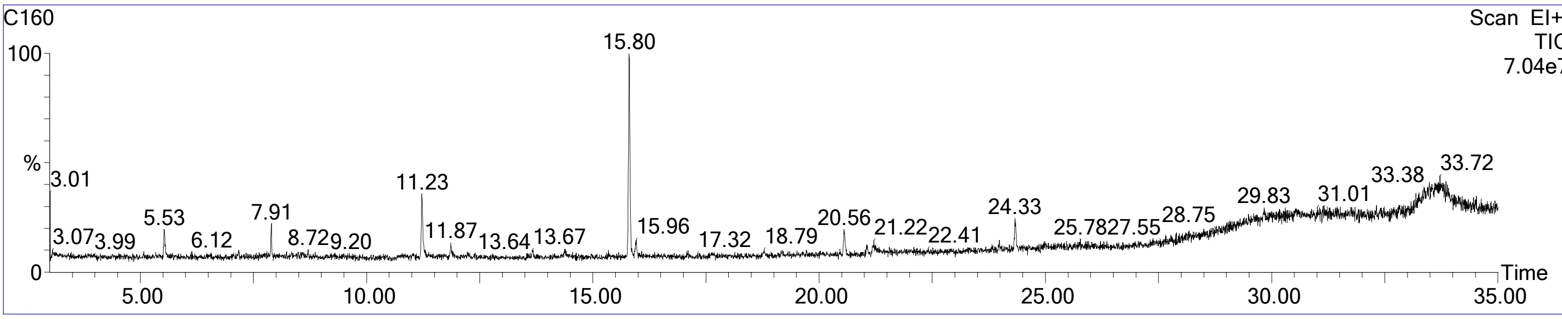

#	RT	Scan	Height	Area	Area %	Norm %
1	5.534	507	9,493,158	391,403.0	0.819	14.96

Pk #	RT	Hit	Compound Name	Match	R.Match	Prob.	CAS	Library
1	5.534	1	Benzaldehyde, 2-methyl-	724	861	17.8	529-20-4	mainlib
		2	Benzaldehyde, 3-methyl-	717	851	13.6	620-23-5	replib
		3	Benzaldehyde, 4-methyl-	712	846	11.0	104-87-0	mainlib
		4	Benzaldehyde, 3-methyl-	711	844	13.6	620-23-5	mainlib
		5	Benzaldehyde, 4-methyl-	710	848	11.0	104-87-0	replib
		6	Bicyclo[4.2.0]octa-1,3,5-trien-7-ol	708	847	9.3	35447-99-5	mainlib
		7	Benzaldehyde, 2-methyl-	706	835	17.8	529-20-4	replib
		8	Benzaldehyde, 4-methyl-	703	841	11.0	104-87-0	replib
		9	Benzaldehyde, 4-methyl-	702	838	11.0	104-87-0	replib
		10	Benzaldehyde, 4-methyl-	700	849	11.0	104-87-0	replib

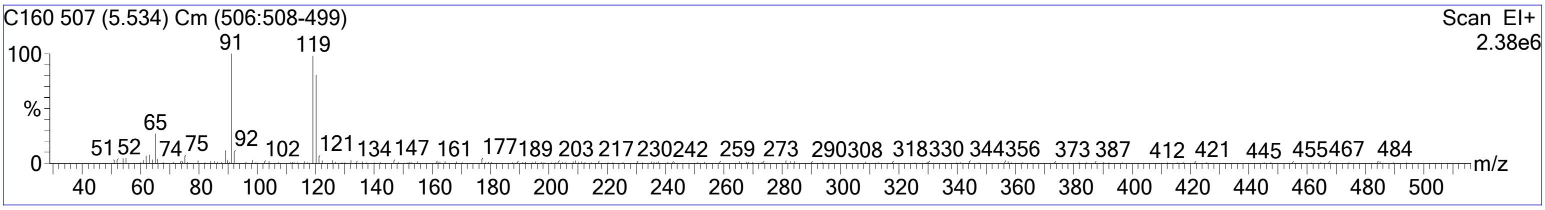

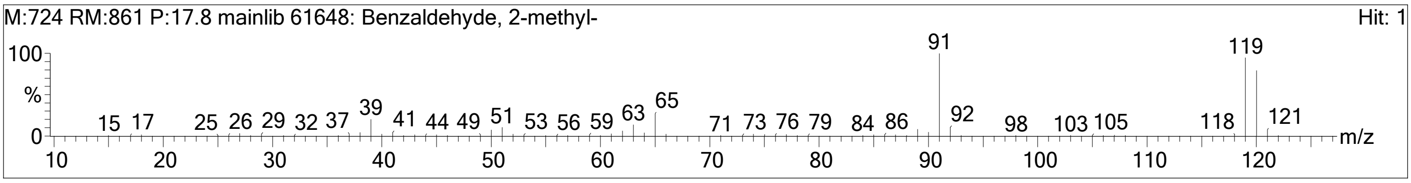

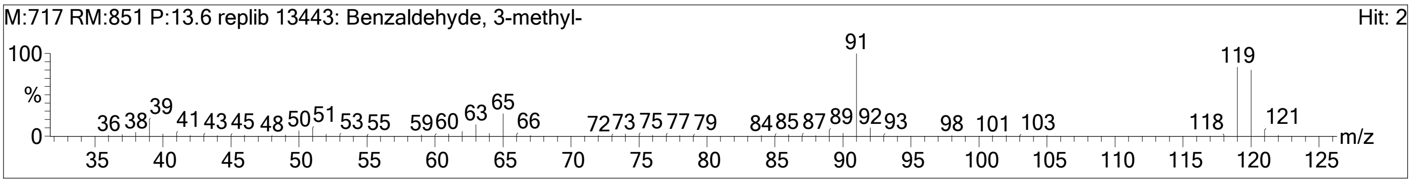

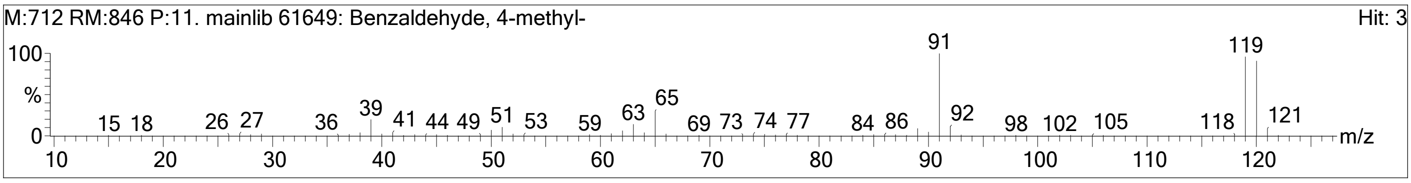

TAMILNADU AGRICULTURAL UNIVERSITY - AGRICULTURAL MICROBIOLOGY

INSTRUMENT: PERKIN ELMER CLARUS SQ8C COLOUMN: DB-5 MS CAPILARY STANDARD NON - POLARCOLOUMN
INJECTION VOL: 1 MICRO LITER DIMENSION: 30Mts, ID: 0.25 mm, FILM: 0.25 IM CARRIER GAS: He
SAMPLE ID : C160

#	RT	Scan	Height	Area	Area %	Norm %
2	7.905	981	11,602,610	317,636.8	0.665	12.14

Pk #	RT	Hit	Compound Name	Match	R.Match	Prob.	CAS	Library
2	7.905	1	Benzene, 1,3-bis(1,1-dimethylethyl)-	680	814	37.5	1014-60-4	replib
		2	Benzene, 1,3-bis(1,1-dimethylethyl)-	663	796	37.5	1014-60-4	replib
		3	Benzene, 1,3-bis(1,1-dimethylethyl)-	657	796	37.5	1014-60-4	mainlib
		4	Benzene, 1,4-bis(1,1-dimethylethyl)-	657	784	13.7	1012-72-2	replib
		5	Benzene, 1,4-bis(1,1-dimethylethyl)-	652	772	13.7	1012-72-2	mainlib
		6	Benzo[c]furanone, 3,3,4,7-tetramethyl-	648	759	9.9	37740-08-2	mainlib
		7	m-Cymene, 5-tert-butyl-	637	748	6.8	29577-19-3	mainlib
		8	Benzene, 1,4-dimethyl-2,5-bis(1-methylethyl)-	632	731	5.5	10375-96-9	replib
		9	Benzene, 1,5-dimethyl-2,4-bis(1-methylethyl)-	629	760	4.9	5186-68-5	mainlib
		10	Benzene, 1,4-dimethyl-2,5-bis(1-methylethyl)-	625	734	5.5	10375-96-9	replib

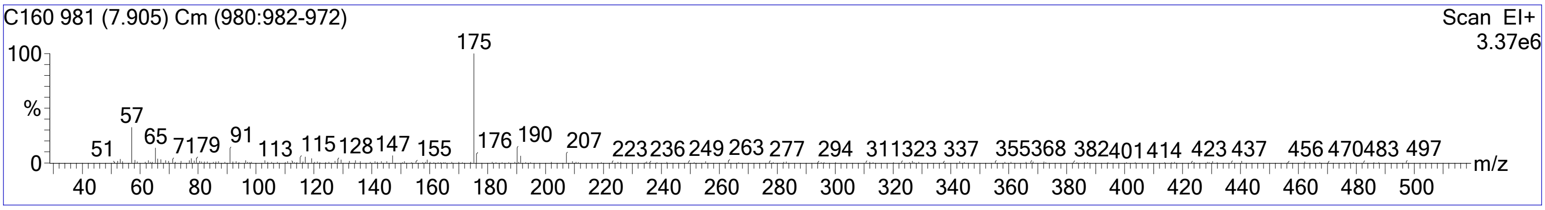

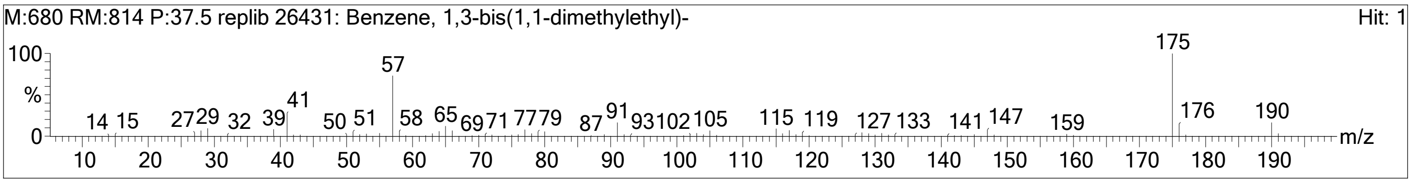

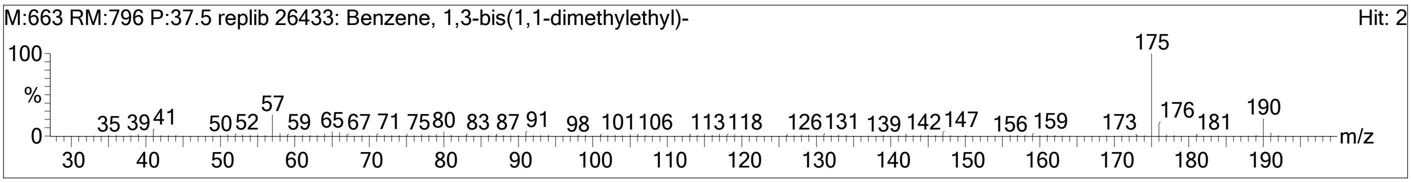

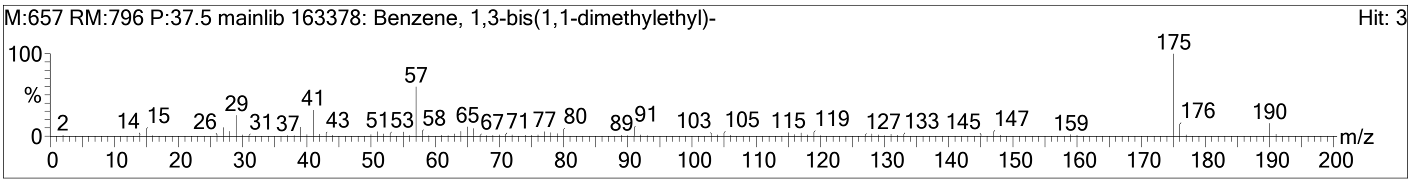

TAMILNADU AGRICULTURAL UNIVERSITY - AGRICULTURAL MICROBIOLOGY

INSTRUMENT: PERKIN ELMER CLARUS SQ8C COLOUMN: DB-5 MS CAPILARY STANDARD NON - POLARCOLOUMN
INJECTION VOL: 1 MICRO LITER DIMENSION: 30Mts, ID: 0.25 mm, FILM: 0.25 IM CARRIER GAS: He
SAMPLE ID : C160

#	RT	Scan	Height	Area	Area %	Norm %
3	11.227	1645	20,994,868	905,327.2	1.894	34.61

Pk #	RT	Hit	Compound Name	Match	R.Match	Prob.	CAS	Library
3	11.227	1	1-Dodecanol	740	862	7.0	112-53-8	replib
		2	1-Undecanol	732	875	5.2	112-42-5	mainlib
		3	1-Hexadecanol	730	844	4.8	36653-82-4	mainlib
		4	1-Pentadecene	727	841	4.2	13360-61-7	replib
		5	n-Tridecan-1-ol	722	832	3.4	112-70-9	replib
		6	Cyclotetradecane	721	835	3.3	295-17-0	mainlib
		7	Hexadecen-1-ol, trans-9-	721	835	3.3	64437-47-4	mainlib
		8	Cyclopentadecane	721	833	3.3	295-48-7	replib
		9	n-Tridecan-1-ol	719	814	3.4	112-70-9	replib
		10	Cyclotridecane	718	844	2.9	295-02-3	mainlib

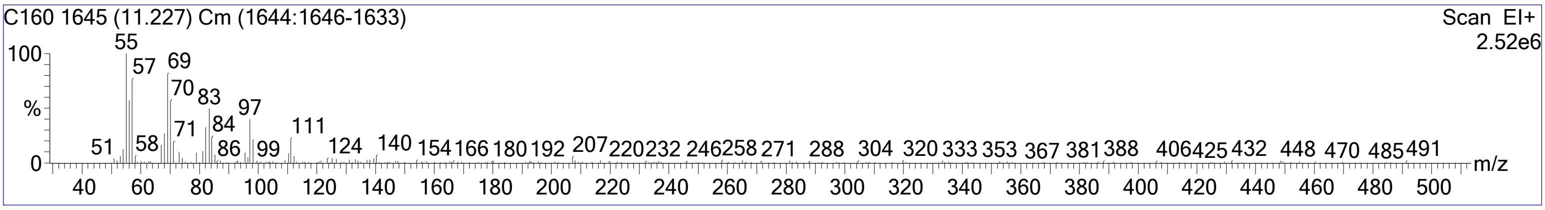

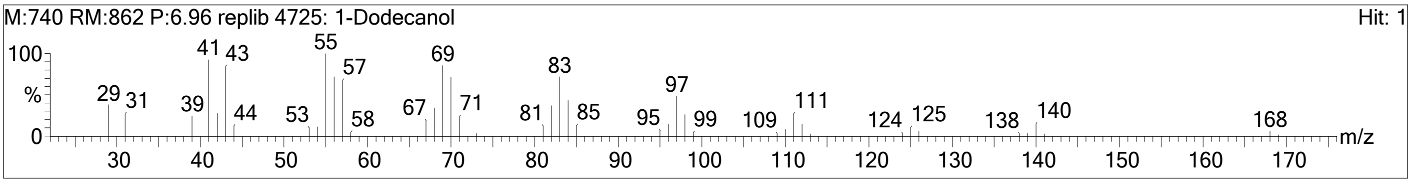

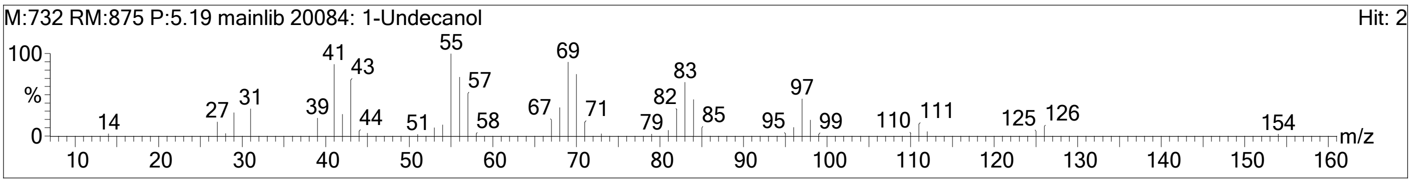

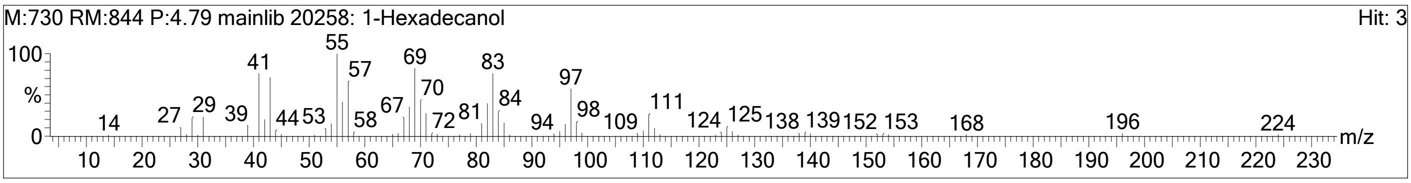

TAMILNADU AGRICULTURAL UNIVERSITY - AGRICULTURAL MICROBIOLOGY

INSTRUMENT: PERKIN ELMER CLARUS SQ8C COLOUMN: DB-5 MS CAPILARY STANDARD NON - POLARCOLOUMN
INJECTION VOL: 1 MICRO LITER DIMENSION: 30Mts, ID: 0.25 mm, FILM: 0.25 IM CARRIER GAS: He
SAMPLE ID : C160

#	RT	Scan	Height	Area	Area %	Norm %
4	11.867	1773	5,417,990	237,810.4	0.498	9.09

Pk #	RT	Hit	Compound Name	Match	R.Match	Prob.	CAS	Library
4	11.867	1	2,4-Di-tert-butylphenol	569	718	28.7	96-76-4	replib
		2	Phenol, 3,5-bis(1,1-dimethylethyl)-	561	719	21.4	1138-52-9	replib
		3	2,4-Di-tert-butylphenol	555	728	28.7	96-76-4	mainlib
		4	2,4-Di-tert-butylphenol	550	756	28.7	96-76-4	replib
		5	Phenol, 2,6-bis(1,1-dimethylethyl)-	548	692	13.8	128-39-2	replib
		6	Phenol, 3,5-bis(1,1-dimethylethyl)-	545	740	21.4	1138-52-9	replib
		7	Phenol, 3,5-bis(1,1-dimethylethyl)-	543	723	21.4	1138-52-9	replib
		8	Phenol, 2,5-bis(1,1-dimethylethyl)-	541	710	10.6	5875-45-6	mainlib
		9	Phenol, 2,5-bis(1,1-dimethylethyl)-	541	694	10.6	5875-45-6	replib
		10	Phenol, 2,6-bis(1,1-dimethylethyl)-	528	685	13.8	128-39-2	replib

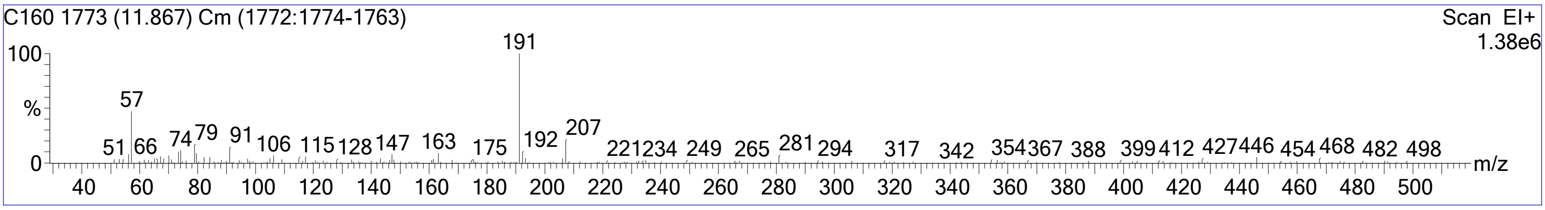

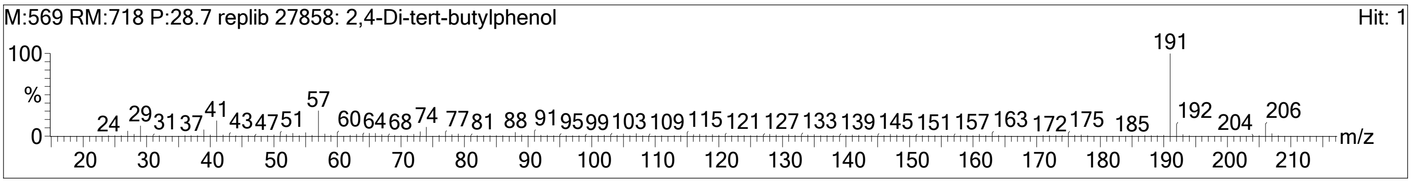

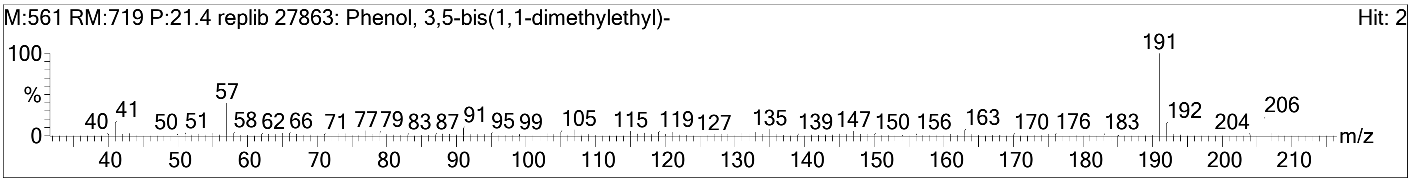

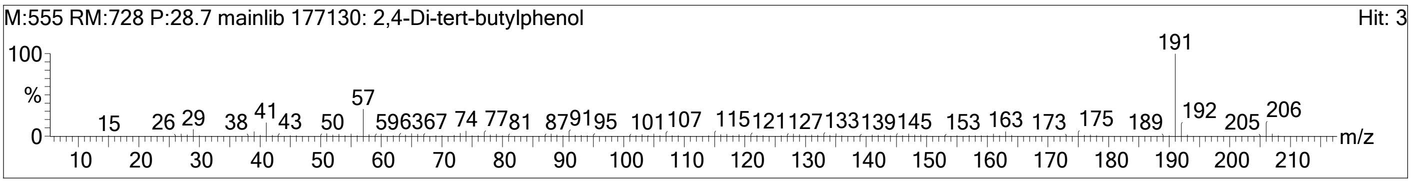

TAMILNADU AGRICULTURAL UNIVERSITY - AGRICULTURAL MICROBIOLOGY

INSTRUMENT: PERKIN ELMER CLARUS SQ8C COLOUMN: DB-5 MS CAPILARY STANDARD NON - POLARCOLOUMN
INJECTION VOL: 1 MICRO LITER DIMENSION: 30Mts, ID: 0.25 mm, FILM: 0.25 IM CARRIER GAS: He
SAMPLE ID : C160

#	RT	Scan	Height	Area	Area %	Norm %
5	15.804	2560	65,737,824	2,616,165.8	5.473	100.00

Pk #	RT	Hit	Compound Name	Match	R.Match	Prob.	CAS	Library
5	15.804	1	Dodecyl acrylate	873	902	48.5	2156-97-0	replib
		2	Dodecyl acrylate	870	904	48.5	2156-97-0	replib
		3	Dodecyl acrylate	792	820	48.5	2156-97-0	mainlib
		4	2-Propenoic acid, tridecyl ester	791	821	4.9	3076-04-8	mainlib
		5	1-Dodecanol	783	836	3.7	112-53-8	replib
		6	2-Propenoic acid, pentadecyl ester	775	802	2.8	43080-23-5	mainlib
		7	Ethylene diacrylate	762	795	1.8	2274-11-5	mainlib
		8	1-Tetradecanol	761	777	1.7	112-72-1	replib
		9	1-Undecanol	758	852	1.5	112-42-5	mainlib
		10	1-Dodecanol	757	805	3.7	112-53-8	replib

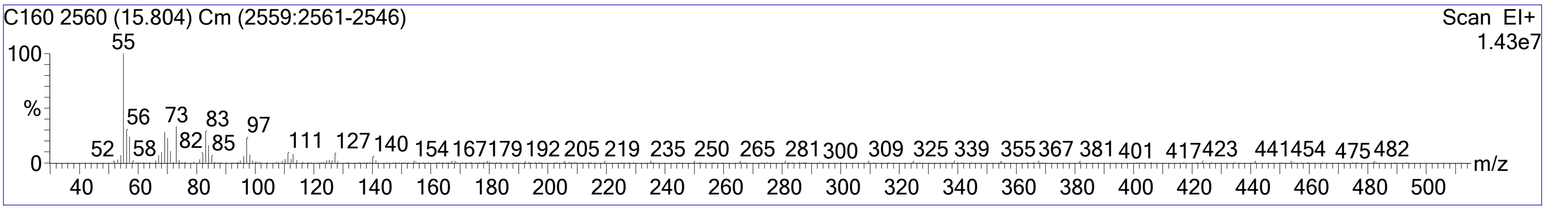

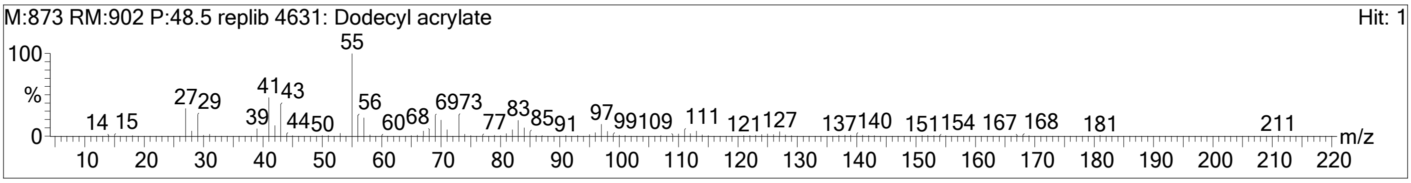

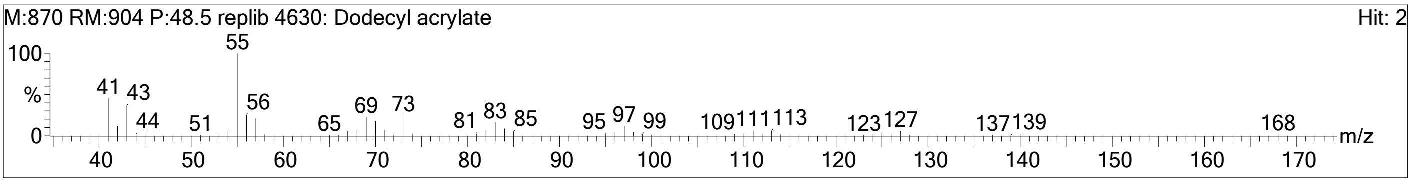

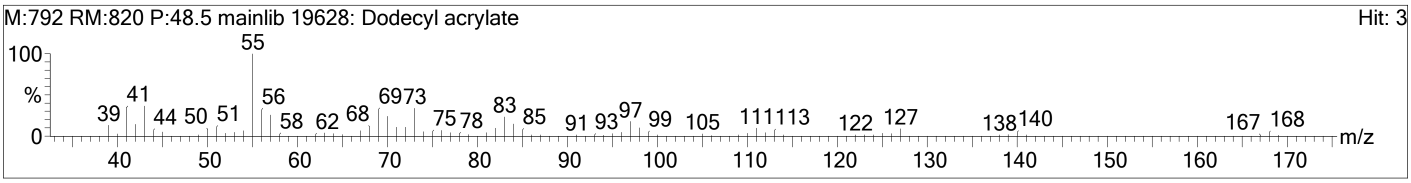

TAMILNADU AGRICULTURAL UNIVERSITY - AGRICULTURAL MICROBIOLOGY

INSTRUMENT: PERKIN ELMER CLARUS SQ8C COLOUMN: DB-5 MS CAPILARY STANDARD NON - POLARCOLOUMN
INJECTION VOL: 1 MICRO LITER DIMENSION: 30Mts, ID: 0.25 mm, FILM: 0.25 IM CARRIER GAS: He
SAMPLE ID : C160

#	RT	Scan	Height	Area	Area %	Norm %
6	15.964	2592	5,955,808	261,784.0	0.548	10.01

Pk #	RT	Hit	Compound Name	Match	R.Match	Prob.	CAS	Library
6	15.964	1	Hexadecane, 1,1-bis(dodecyloxy)-	486	557	5.3	56554-64-4	mainlib
		2	5-Octadecenal	479	556	4.1	56554-88-2	mainlib
		3	17-Pentatriacontene	476	575	3.6	6971-40-0	replib
		4	17-Pentatriacontene	472	501	3.6	6971-40-0	mainlib
		5	Vinyl lauryl ether	470	737	2.8	765-14-0	mainlib
		6	Tetrapentacontane, 1,54-dibromo-	469	538	2.7		mainlib
		7	Z-10-Methyl-11-tetradecen-1-ol propionate	468	549	2.6		mainlib
		8	Oleic Acid	466	535	2.4	112-80-1	replib
		9	cis-11-Eicosenoic acid	465	524	2.3	5561-99-9	mainlib
		10	1-Tricosanol	463	520	2.1	3133-01-5	replib

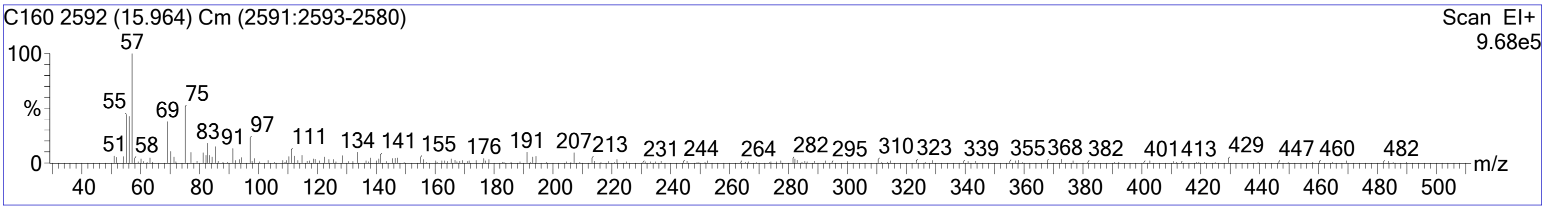

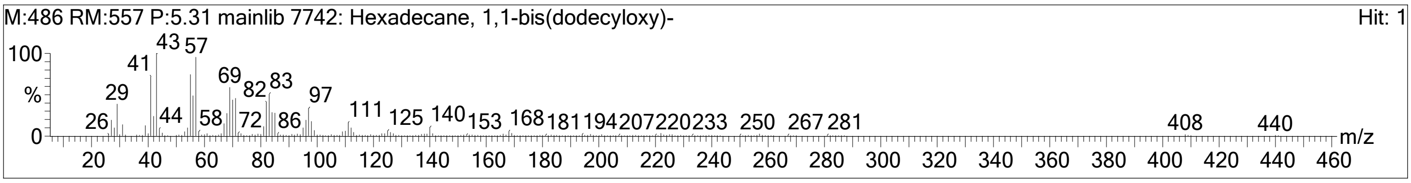

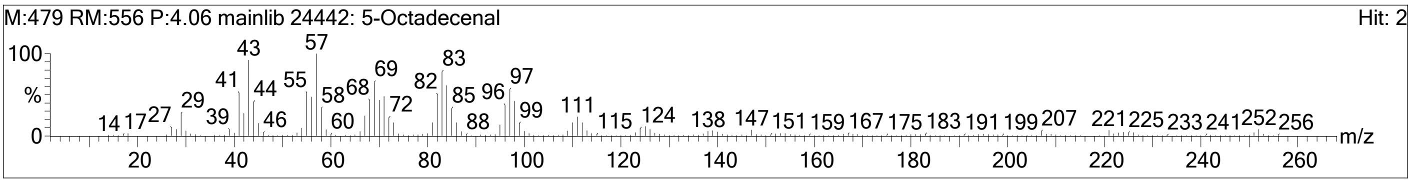

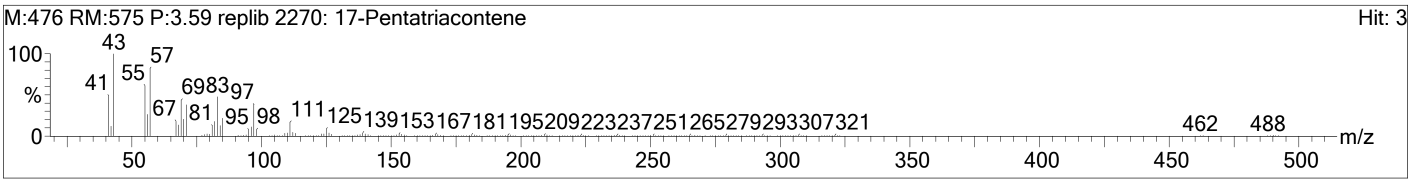

TAMILNADU AGRICULTURAL UNIVERSITY - AGRICULTURAL MICROBIOLOGY

INSTRUMENT: PERKIN ELMER CLARUS SQ8C   COLOUMN: DB-5 MS CAPILARY STANDARD NON - POLARCOLOUMN
INJECTION VOL: 1 MICRO LITER   DIMENSION: 30Mts, ID: 0.25 mm, FILM: 0.25 IM   CARRIER GAS: He
SAMPLE ID : C160

#	RT	Scan	Height	Area	Area %	Norm %
7	17.634	2926	2,179,672	187,130.9	0.392	7.15

Pk #	RT	Hit	Compound Name	Match	R.Match	Prob.	CAS	Library
7	17.634	1	Octasiloxane, 1,1,3,3,5,5,7,7,9,9,11,11,13,13,15,15-hexadecamethyl-	364	522	11.3	19095-24-0	mainlib
		2	2,4-Imidazolidinedione, 5-[3,4-bis[(trimethylsilyl)oxy]phenyl]-3-methyl-5-phenyl-1-(trimethylsilyl)-	362	384	10.4	55517-85-6	mainlib
		3	Heptasiloxane, 1,1,3,3,5,5,7,7,9,9,11,11,13,13-tetradecamethyl-	358	520	8.8	19095-23-9	mainlib
		4	17-(1,5-Dimethylhexyl)-10,13-dimethyl-3-styrylhexadecahydrocyclopenta[a]phenanthren-2-one	352	372	6.9		mainlib
		5	Morphine, 2TMS derivative	340	396	4.6	55449-66-6	replib
		6	(+)-Prostaglandin F2à, 4TMS derivative	338	374	4.2	50669-95-9	mainlib
		7	Morphine, 2TMS derivative	337	358	4.6	55449-66-6	replib
		8	Hexasiloxane, 1,1,3,3,5,5,7,7,9,9,11,11-dodecamethyl-	329	451	3.1	995-82-4	mainlib
		9	Morphine, bis(trimethylsilyl) ether	326	345	2.7		mainlib
		10	Pregn-4-en-3-one, 11,17,20,21-tetrakis[(trimethylsilyl)oxy]-, O-methyloxime, (11á, 20S)-	323	366	2.4	57325-93-6	mainlib

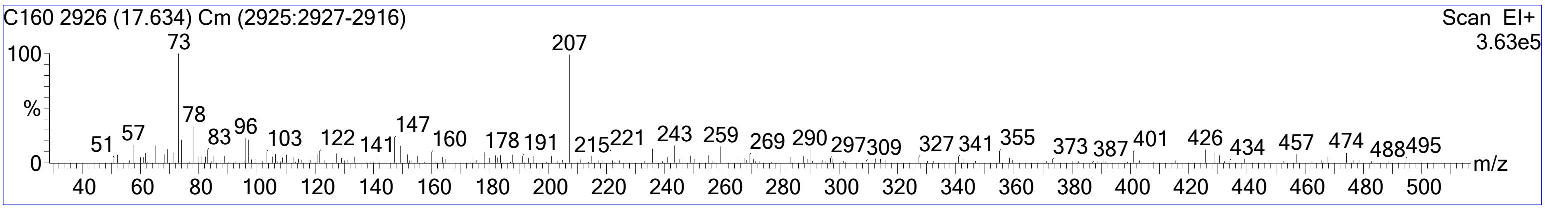

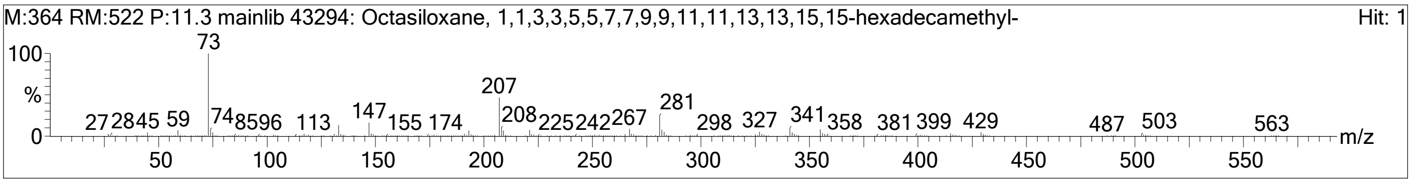

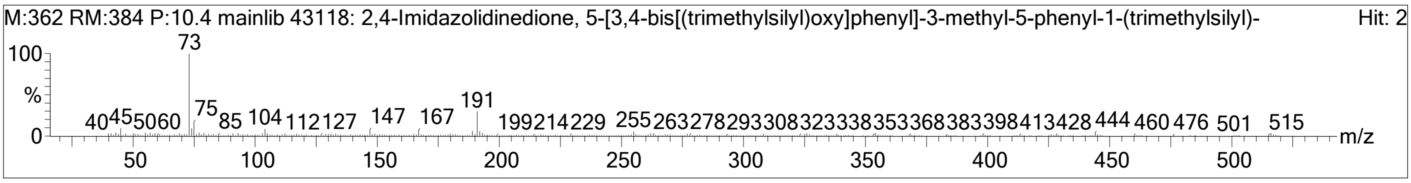

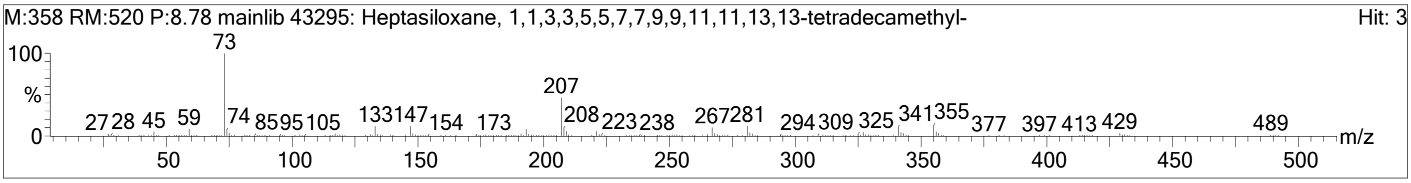

TAMILNADU AGRICULTURAL UNIVERSITY - AGRICULTURAL MICROBIOLOGY

INSTRUMENT: PERKIN ELMER CLARUS SQ8C COLOUMN: DB-5 MS CAPILARY STANDARD NON - POLARCOLOUMN
INJECTION VOL: 1 MICRO LITER DIMENSION: 30Mts, ID: 0.25 mm, FILM: 0.25 IM CARRIER GAS: He
SAMPLE ID : C160

#	RT	Scan	Height	Area	Area %	Norm %
8	20.555	3510	9,525,572	517,484.4	1.083	19.78

Pk #	RT	Hit	Compound Name	Match	R.Match	Prob.	CAS	Library
8	20.555	1	Hexadecanoic acid, methyl ester	544	801	10.2	112-39-0	replib
		2	Undecanoic acid, 2-methyl-	536	838	7.6	24323-25-9	mainlib
		3	Tetracosanoic acid, methyl ester	518	619	3.9	2442-49-1	replib
		4	Heptacosanoic acid, methyl ester	517	676	3.8	55682-91-2	mainlib
		5	Hexadecanoic acid, methyl ester	517	673	10.2	112-39-0	mainlib
		6	Decanoic acid, 2-methyl-	516	859	3.6	24323-23-7	mainlib
		7	Hexadecanoic acid, 15-methyl-, methyl ester	515	745	3.5	6929-04-0	mainlib
		8	Pentadecanoic acid, 14-methyl-, methyl ester	514	714	3.3	5129-60-2	mainlib
		9	Hexadecanoic acid, 14-methyl-, methyl ester	514	600	3.3	2490-49-5	mainlib
		10	Methyl tetradecanoate	510	707	2.8	124-10-7	replib

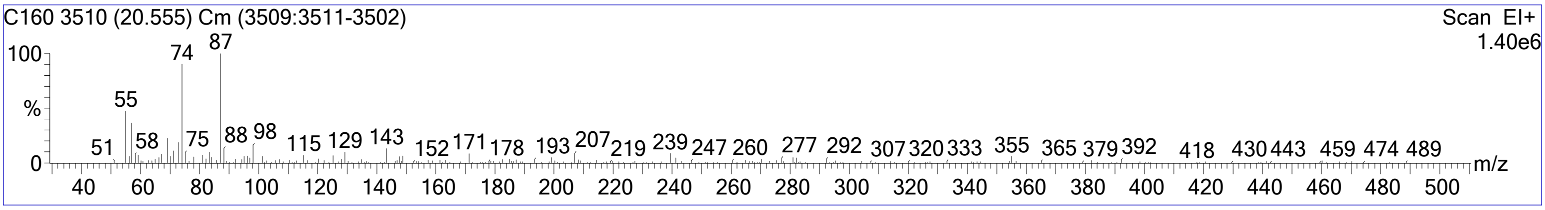

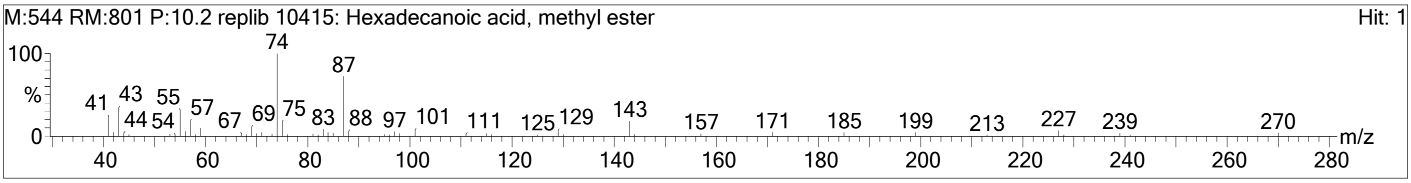

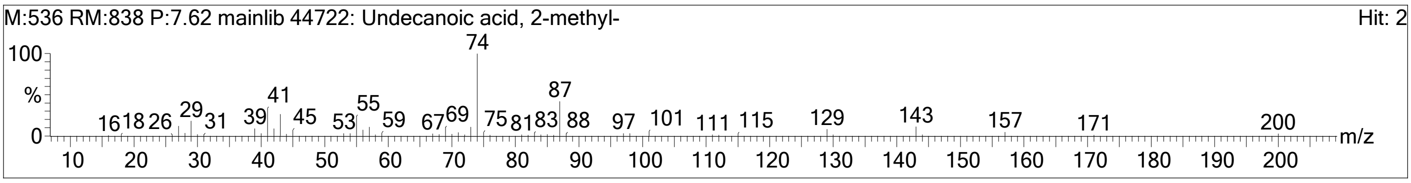

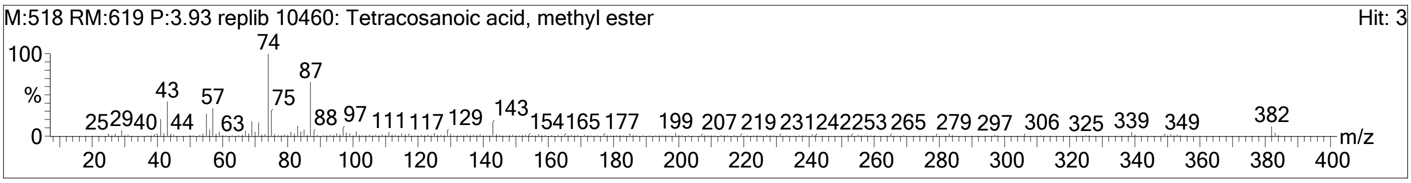

TAMILNADU AGRICULTURAL UNIVERSITY - AGRICULTURAL MICROBIOLOGY

INSTRUMENT: PERKIN ELMER CLARUS SQ8C
COLOUMN: DB-5 MS CAPILARY STANDARD NON - POLARCOLOUMN
INJECTION VOL: 1 MICRO LITER
DIMENSION: 30Mts, ID: 0.25 mm, FILM: 0.25 IM
CARRIER GAS: He
SAMPLE ID : C160

#	RT	Scan	Height	Area	Area %	Norm %
9	21.051	3609	4,116,618	157,959.8	0.330	6.04

Pk #	RT	Hit	Compound Name	Match	R.Match	Prob.	CAS	Library
9	21.051	1	Diethyl phthalate	583	900	52.1	84-66-2	nist_msms
		2	Metaproterenol	548	902	13.1	586-06-1	nist_msms
		3	Methyldopa	491	929	2.4	555-30-6	nist_msms
		4	Ethaneperoxoic acid, 1-cyano-1-[2-(2-phenyl-1,3-dioxolan-2-yl)ethyl]pentyl ester	481	920	1.7	58422-92-7	mainlib
		5	1,2-Benzenedicarboxylic acid	481	918	1.7	88-99-3	nist_msms
		6	Benzoylmetronidazole	477	875	1.5	13182-89-3	nist_msms
		7	Dibutyl phthalate	474	686	1.3	84-74-2	replib
		8	1,2-Benzenedicarboxylic acid, butyl octyl ester	473	652	1.2	84-78-6	replib
		9	Benzyl butyl phthalate	469	627	1.0	85-68-7	replib
		10	1,2-Benzenedicarboxylic acid, butyl 2-ethylhexyl ester	463	676	0.8	85-69-8	replib

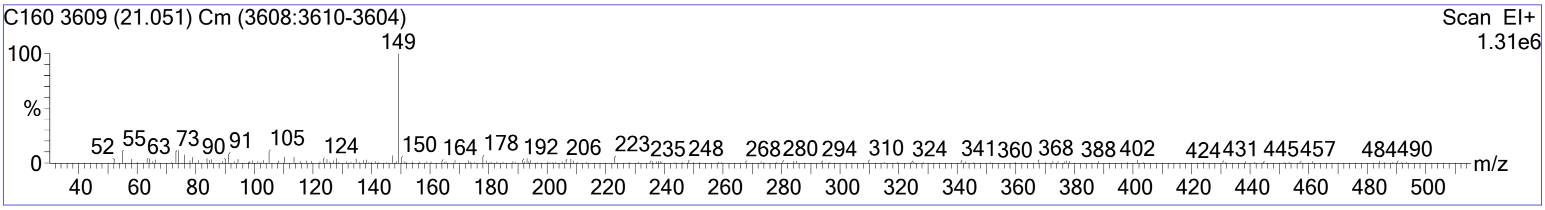

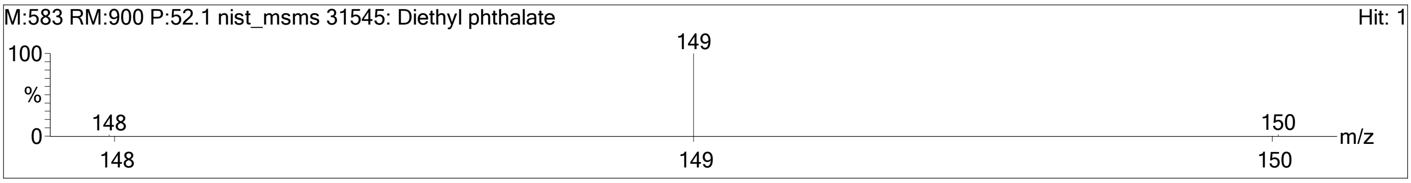

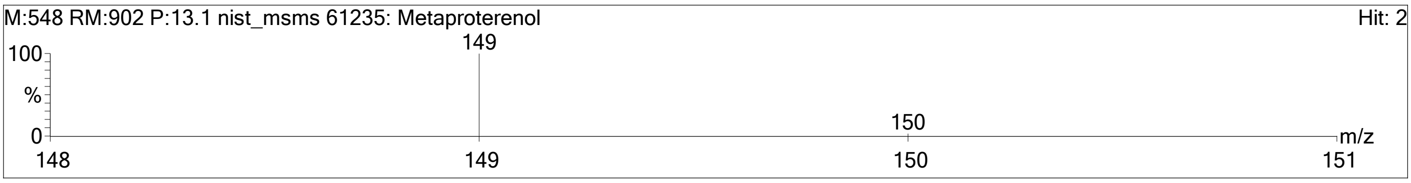

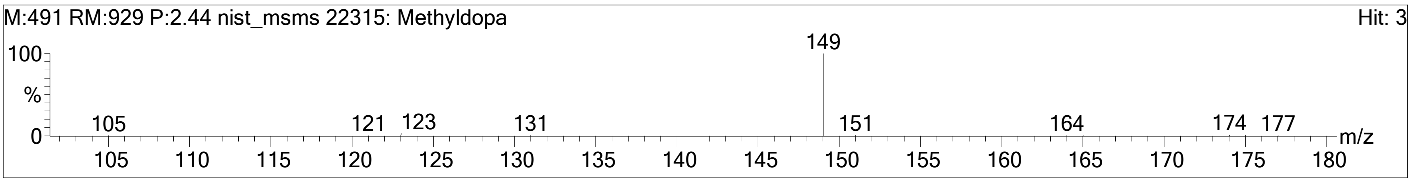

TAMILNADU AGRICULTURAL UNIVERSITY - AGRICULTURAL MICROBIOLOGY

INSTRUMENT: PERKIN ELMER CLARUS SQ8C COLOUMN: DB-5 MS CAPILARY STANDARD NON - POLARCOLOUMN
INJECTION VOL: 1 MICRO LITER DIMENSION: 30Mts, ID: 0.25 mm, FILM: 0.25 IM CARRIER GAS: He
SAMPLE ID : C160

#	RT	Scan	Height	Area	Area %	Norm %
10	21.196	3638	3,936,683	150,992.9	0.316	5.77

Pk #	RT	Hit	Compound Name	Match	R.Match	Prob.	CAS	Library
10	21.196	1	Dasycarpidan-1-methanol, acetate (ester)	442	509	5.3	55724-48-6	mainlib
		2	Octadecanoic acid, 2-hydroxy-1,3-propanediyl ester	442	498	5.3	504-40-5	replib
		3	Octadecanoic acid, 4-hydroxybutyl ester	440	516	4.9	15337-64-1	mainlib
		4	Hexadecanoic acid, 1-(hydroxymethyl)-1,2-ethanediyl ester	437	476	4.3	761-35-3	mainlib
		5	Pentadecanoic acid, 14-bromo-	434	523	3.8	74685-41-9	mainlib
		6	n-Decanoic acid	432	618	3.5	334-48-5	replib
		7	Octadecanoic acid	430	542	3.2	57-11-4	replib
		8	Octadecanoic acid	427	555	3.2	57-11-4	mainlib
		9	Pentadecanoic acid, 15-bromo-	426	513	2.8	56523-59-2	mainlib
		10	Octadecanoic acid	425	592	3.2	57-11-4	replib

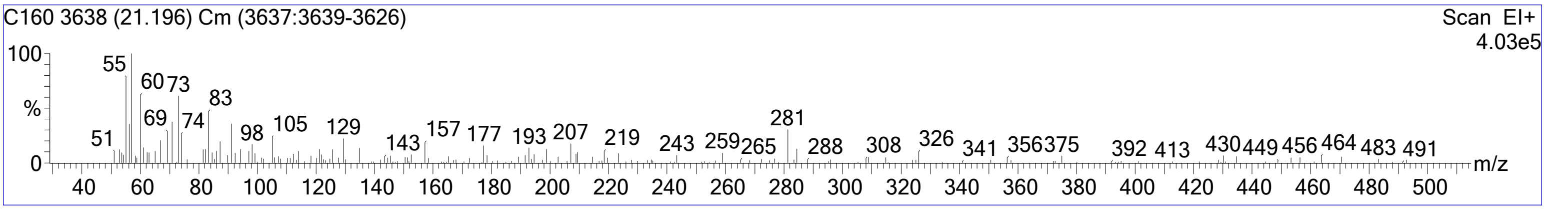

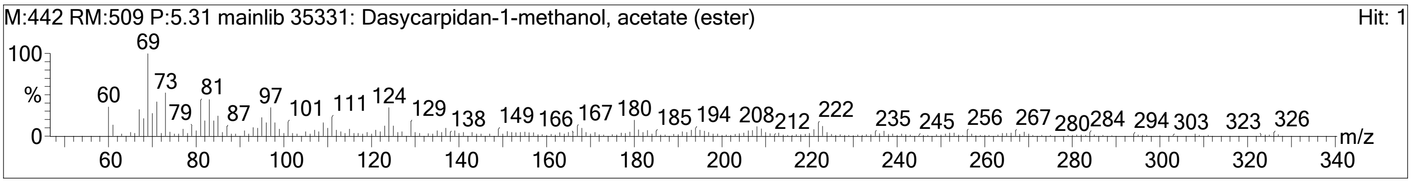

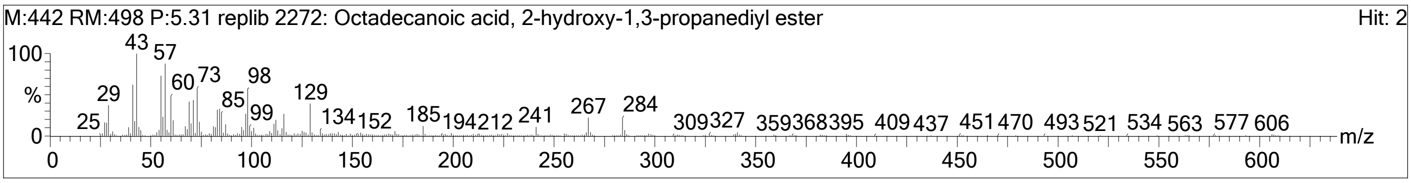

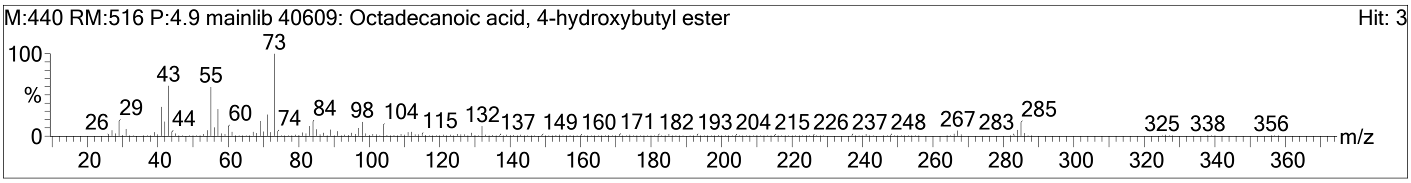

TAMILNADU AGRICULTURAL UNIVERSITY - AGRICULTURAL MICROBIOLOGY

INSTRUMENT: PERKIN ELMER CLARUS SQ8C COLOUMN: DB-5 MS CAPILARY STANDARD NON - POLARCOLOUMN
INJECTION VOL: 1 MICRO LITER DIMENSION: 30Mts, ID: 0.25 mm, FILM: 0.25 IM CARRIER GAS: He
SAMPLE ID : C160

#	RT	Scan	Height	Area	Area %	Norm %
11	21.326	3664	3,183,206	156,253.2	0.327	5.97

Pk #	RT	Hit	Compound Name	Match	R.Match	Prob.	CAS	Library
11	21.326	1	6-Hydroxy-powelline-N-nitroso-7-demethoxy-, aldehyde	373	442	12.0		mainlib
		2	Bufa-20,22-dienolide, 14,15-epoxy-3,16-dihydroxy-, (3á,5á,15á,16á)-	371	405	11.1	4026-95-3	mainlib
		3	4H-Cyclopropa[5',6']benz[1',2':7,8]azuleno[5,6-b]oxiren-4-one, 8,8a-bis(acetyloxy)-2a-[(acetyloxy)methyl]-1,1a,1b,1c,2a,3,3a,6a,6b,7,8,8a-dodecahydro-6b-hydroxy-3a-methoxy-1,1,5,7-tetramethyl-, [1aR-(1aà,1bá,1cà,2aà,3aá,6aà,6bà,7à,8á,8aà)]-	363	374	8.2	64807-01-8	mainlib
		4	Bufa-20,22-dienolide, 14,15-epoxy-3,11-dihydroxy-, (3á,5á,11à,15á)-	361	399	7.6	39005-15-7	mainlib
		5	Powelline, 6-hydroxy-	346	425	4.6	31128-98-0	mainlib
		6	Bufa-20,22-dienolide, 16-(acetyloxy)-14,15-epoxy-3-hydroxy-, (3á,5á,15á,16á)-	342	367	3.9	470-37-1	mainlib
		7	7,7-Dibromobicyclo[2.2.1]heptane-1-carboxylic acid, methyl ester	336	431	3.1	59093-00-4	mainlib
		8	Chlortetracycline	334	349	2.8	57-62-5	replib
		9	Pregn-16-en-20-one, 11,18-bis(acetyloxy)-3,9-epoxy-3-methoxy-, (3à,5á,11à)-	332	354	2.6	30384-39-5	mainlib
		10	Bufa-20,22-dienolide, 3-(acetyloxy)-14,15-epoxy-16-hydroxy-, (3á,5á,15á,16á)-	329	349	2.3	4026-96-4	replib

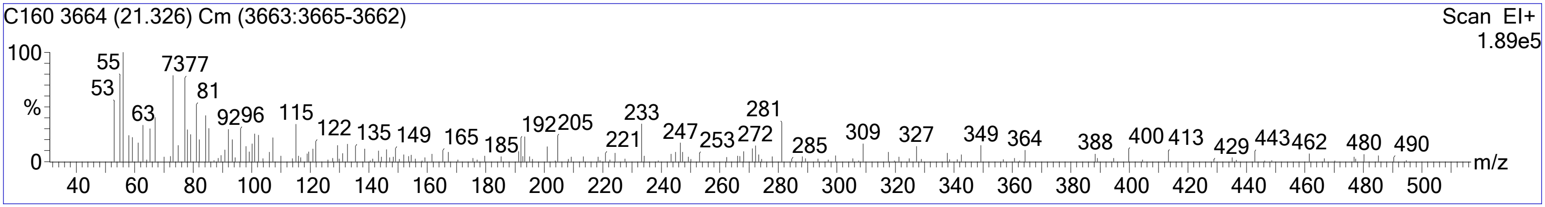

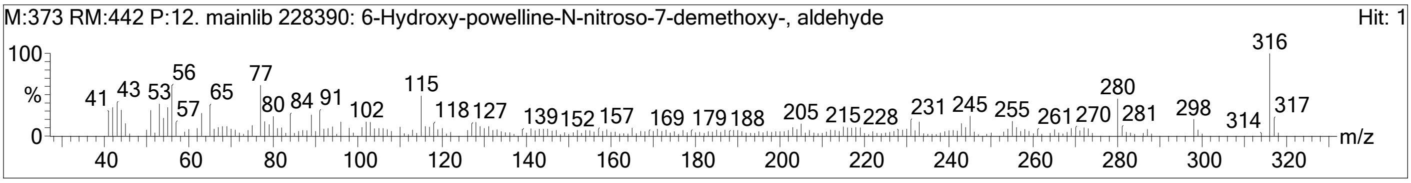

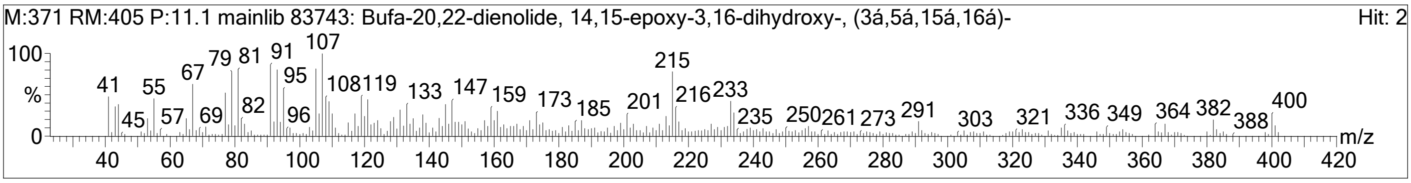

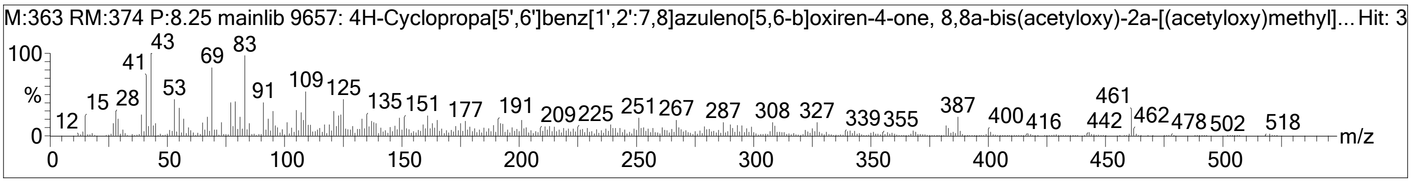

TAMILNADU AGRICULTURAL UNIVERSITY - AGRICULTURAL MICROBIOLOGY

INSTRUMENT: PERKIN ELMER CLARUS SQ8C COLOUMN: DB-5 MS CAPILARY STANDARD NON - POLARCOLOUMN
INJECTION VOL: 1 MICRO LITER DIMENSION: 30Mts, ID: 0.25 mm, FILM: 0.25 IM CARRIER GAS: He
SAMPLE ID : C160

#	RT	Scan	Height	Area	Area %	Norm %
12	21.821	3763	2,408,792	167,188.4	0.350	6.39

Pk #	RT	Hit	Compound Name	Match	R.Match	Prob.	CAS	Library
12	21.821	1	6-Amino-5-cyano-4-(5-cyano-2,4-dimethyl-1H-pyrrol-3-yl)-2-methyl-4H-pyran-3-carboxylic acid ethyl ester	396	473	13.6		mainlib
		2	1-Benzazirene-1-carboxylic acid, 2,2,5a-trimethyl-1a-[3-oxo-1-butenyl] perhydro-, methyl ester	380	469	7.8		mainlib
		3	2H-3,9a-Methano-1-benzoxepin, octahydro-2,2,5a,9-tetramethyl-, [3R-(3à,5aà,9à,9aà)]-	367	502	5.0	5956-09-2	replib
		4	Malonic acid, mononitrile, monothioamide, 2-(3-methyl-2-thienylmethylene)-	358	470	3.7	310454-37-6	mainlib
		5	2-(1-Cyclohexyl-1H-tetrazol-5-ylsulfanylmethyl)-6-nitro-1H-benzoimidazole	355	455	3.2		mainlib
		6	2,6-Di-t-butyloctahydroazulene-3a,8-diol	353	434	3.0		mainlib
		7	2-Myristynoyl pantetheine	351	425	2.8		mainlib
		8	Thieno[2,3-c]furan-3-carbonitrile, 2-amino-4,6-dihydro-4,4,6,6-tetramethyl-	348	459	2.4	447412-24-0	mainlib
		9	Carvacrol, TBDMS derivative	348	454	2.4		mainlib
		10	(2R,3R,4aR,5S,8aS)-2-Hydroxy-4a,5-dimethyl-3-(prop-1-en-2-yl)octahydronaphthalen-1(2H)-one	344	448	2.0	66884-74-0	mainlib

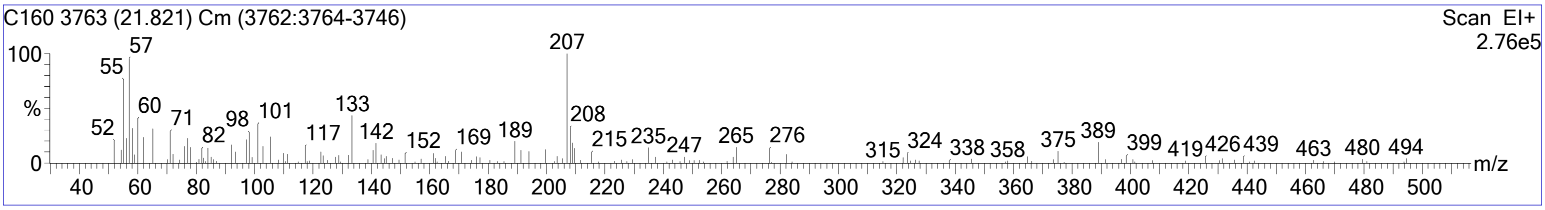

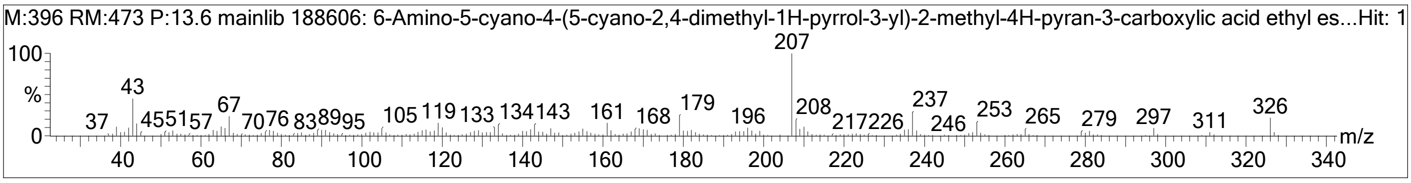

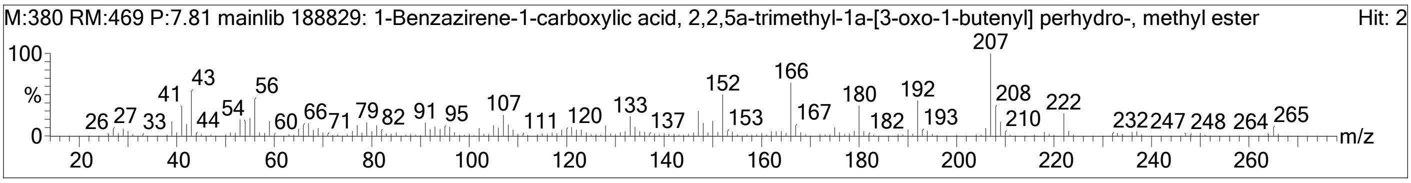

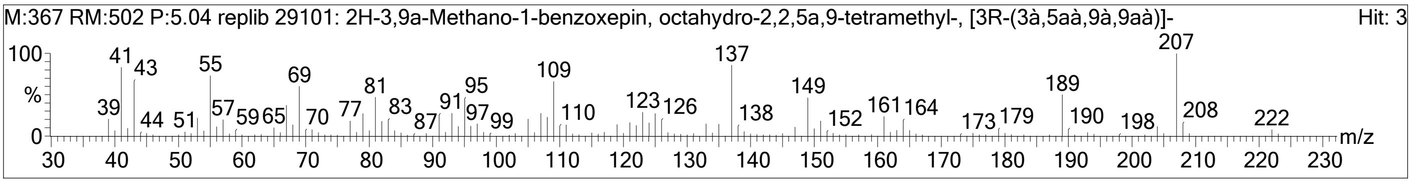

TAMILNADU AGRICULTURAL UNIVERSITY - AGRICULTURAL MICROBIOLOGY

INSTRUMENT: PERKIN ELMER CLARUS SQ8C COLOUMN: DB-5 MS CAPILARY STANDARD NON - POLARCOLOUMN
INJECTION VOL: 1 MICRO LITER DIMENSION: 30Mts, ID: 0.25 mm, FILM: 0.25 IM CARRIER GAS: He
SAMPLE ID : C160

#	RT	Scan	Height	Area	Area %	Norm %
13	24.332	4265	10,652,421	456,686.2	0.955	17.46

Pk #	RT	Hit	Compound Name	Match	R.Match	Prob.	CAS	Library
13	24.332	1	Heptadecanoic acid, 16-methyl-, methyl ester	597	681	19.1	5129-61-3	mainlib
		2	Methyl stearate	581	709	11.0	112-61-8	replib
		3	Heptadecanoic acid, 9-methyl-, methyl ester	574	651	8.4	54934-57-5	mainlib
		4	Methyl stearate	571	718	11.0	112-61-8	replib
		5	Methyl stearate	571	666	11.0	112-61-8	mainlib
		6	Heptadecanoic acid, 10-methyl-, methyl ester	570	643	7.1	2490-25-7	mainlib
		7	Heptadecanoic acid, 16-methyl-, methyl ester	568	640	19.1	5129-61-3	replib
		8	Methyl stearate	565	752	11.0	112-61-8	replib
		9	Hexadecanoic acid, 15-methyl-, methyl ester	563	638	5.4	6929-04-0	replib
		10	Hexadecanoic acid, 14-methyl-, methyl ester	558	629	4.4	2490-49-5	mainlib

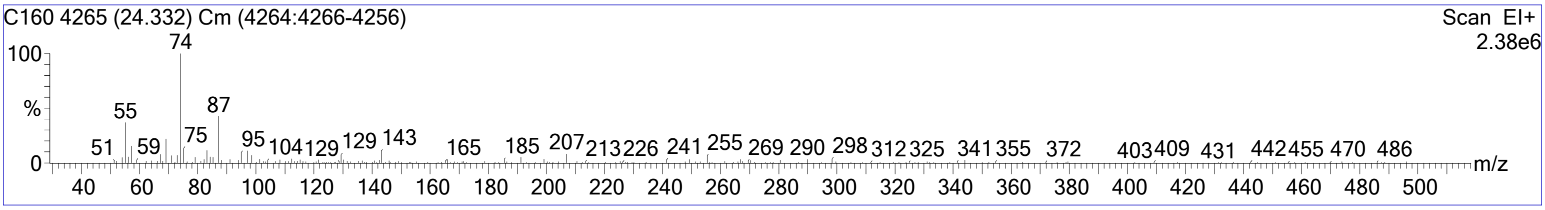

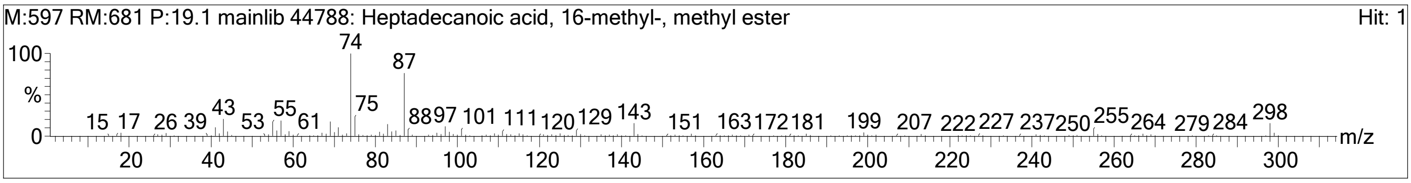

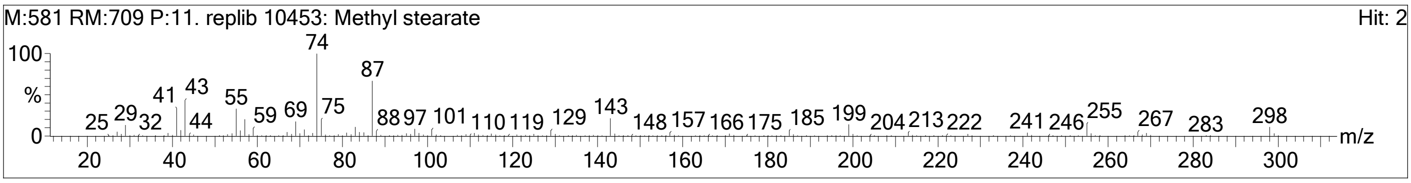

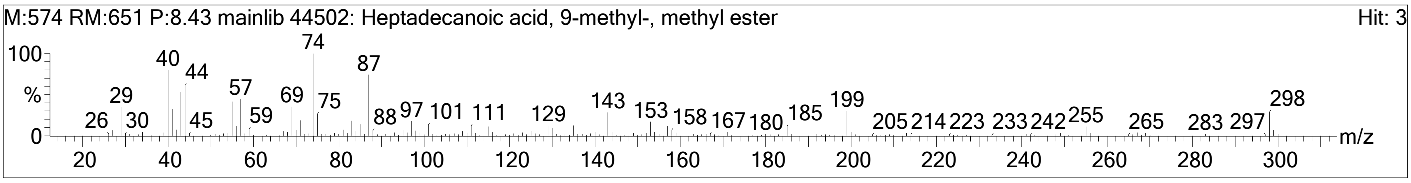

TAMILNADU AGRICULTURAL UNIVERSITY - AGRICULTURAL MICROBIOLOGY

INSTRUMENT: PERKIN ELMER CLARUS SQ8C COLOUMN: DB-5 MS CAPILARY STANDARD NON - POLARCOLOUMN
INJECTION VOL: 1 MICRO LITER DIMENSION: 30Mts, ID: 0.25 mm, FILM: 0.25 IM CARRIER GAS: He
SAMPLE ID : C160

#	RT	Scan	Height	Area	Area %	Norm %
14	29.834	5365	5,393,782	197,319.5	0.413	7.54

Pk #	RT	Hit	Compound Name	Match	R.Match	Prob.	CAS	Library
14	29.834	1	Benzoic acid, 4-methyl-2-trimethylsilyloxy-, trimethylsilyl ester	427	544	12.2	35-27-8	mainlib
		2	Spirost-8-en-11-one, 3-hydroxy-, (3á,5à,14á,20á, 22á,25R)-	413	463	7.6	58072-54-1	mainlib
		3	Octadecane, 1,1'-[1,3-propanediylbis(oxy)]bis-	410	437	6.8	17367-38-3	mainlib
		4	3-Methylsalicylic acid, 2TMS derivative	409	504	6.5		mainlib
		5	Stearic acid, 3-(octadecyloxy)propyl ester	408	441	6.2	17367-40-7	mainlib
		6	Methyl glycocholate, 3TMS derivative	396	431	4.2	57326-16-6	mainlib
		7	2-(tert.-Butyldimethylsilyl)oxybenzylidene acetophenone	395	505	4.0		mainlib
		8	1,2-Propanediol, 3-(octadecyloxy)-, diacetate	395	456	4.0	21994-81-0	mainlib
		9	Hexadecanoic acid, 1-(hydroxymethyl)-1,2-ethanediyl ester	393	419	3.7	761-35-3	mainlib
		10	5-Methylsalicylic acid, 2TMS derivative	388	503	3.0		mainlib

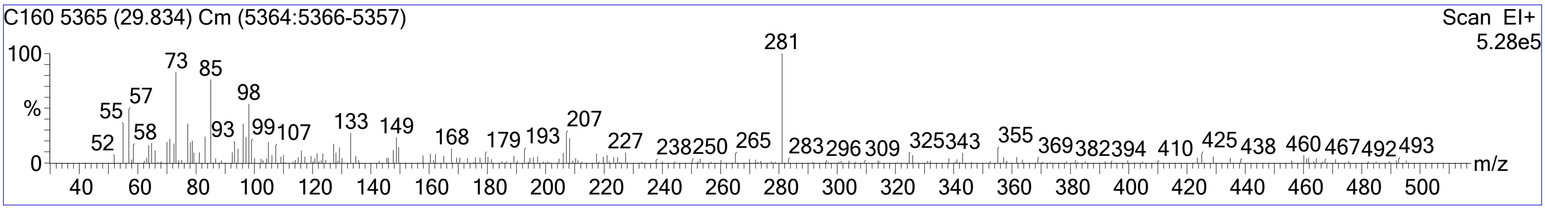

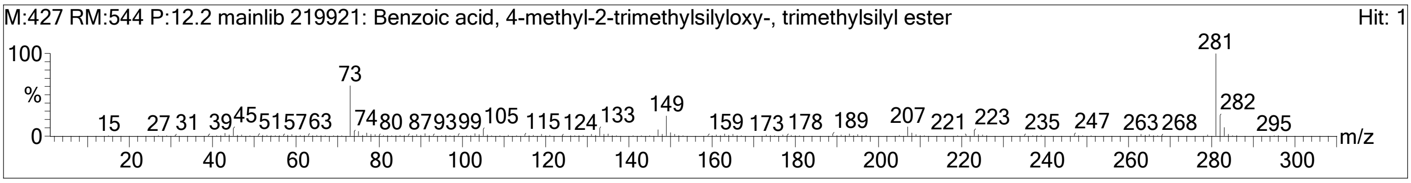

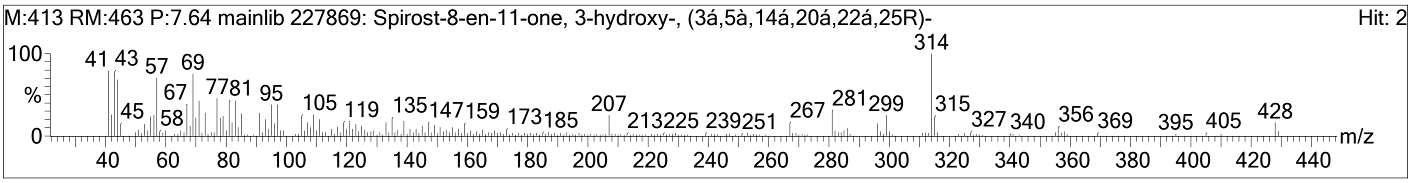

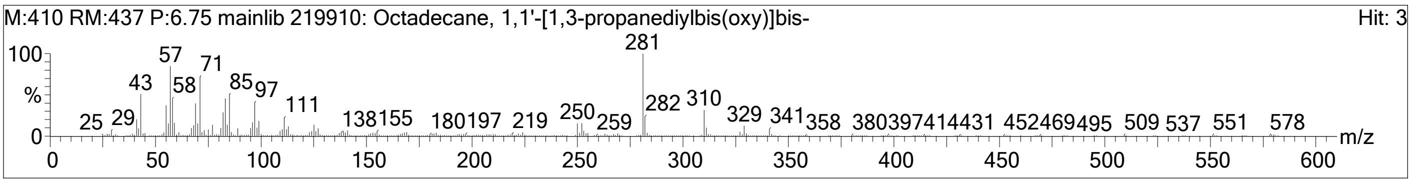

TAMILNADU AGRICULTURAL UNIVERSITY - AGRICULTURAL MICROBIOLOGY

INSTRUMENT: PERKIN ELMER CLARUS SQ8C COLOUMN: DB-5 MS CAPILARY STANDARD NON - POLARCOLOUMN
INJECTION VOL: 1 MICRO LITER DIMENSION: 30Mts, ID: 0.25 mm, FILM: 0.25 IM CARRIER GAS: He
SAMPLE ID : C160

#	RT	Scan	Height	Area	Area %	Norm %
15	30.539	5506	4,350,617	183,841.0	0.385	7.03

Pk #	RT	Hit	Compound Name	Match	R.Match	Prob.	CAS	Library
15	30.539	1	Hexa-t-butylselenatrisiletane	431	455	21.2	93194-15-1	mainlib
		2	3-Dimethylamino-2-(4-chlorphenyl)-thioacrylic acid, thiomorpholide	399	452	5.7		mainlib
		3	1,9-Dioxa-5-thianonane, 3,7-bis(9-borabicyclo[3.3.1]non-9-yloxy)-1,9-diphenyl-	398	426	5.4		mainlib
		4	(-)-Myrtenol, TBDMS derivative	392	517	4.3		mainlib
		5	Hexadecanoic acid, (2-phenyl-1,3-dioxolan-4-yl)methyl ester, cis-	392	427	4.3	42495-31-8	mainlib
		6	18,19-Secoyohimban-19-oic acid, 16,17,20,21-tetradehydro-16-(hydroxymethyl)-, methyl ester, (15á,16E)-	383	443	3.1	5523-49-9	mainlib
		7	1-Methoxy-4-nitro-2,3,5,6-tetramethylbenzene	381	522	2.9	52415-08-4	mainlib
		8	Isodemecolcine, N-desmethyl-	375	423	2.2	102419-92-1	replib
		9	1,5-Bis(4-methoxyphenyl)bicyclo[3.2.0]heptane	372	417	2.0	157367-56-1	mainlib
		10	1-(2,4-Dichloro-phenyl)-N'-hydroxy-cyclopropanecarboxamidine	370	465	1.8		mainlib

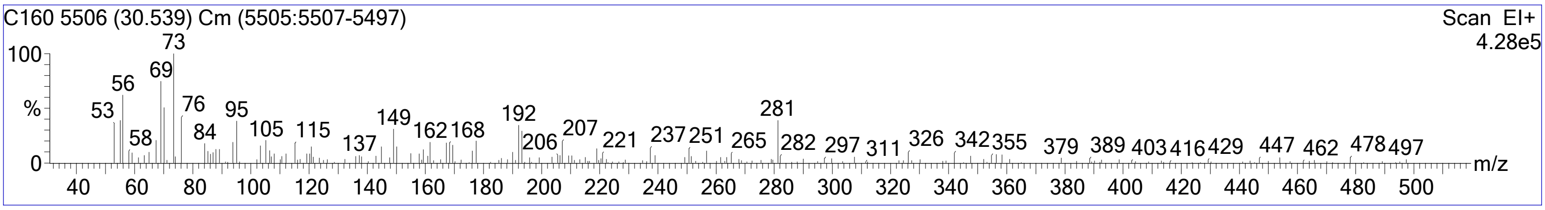

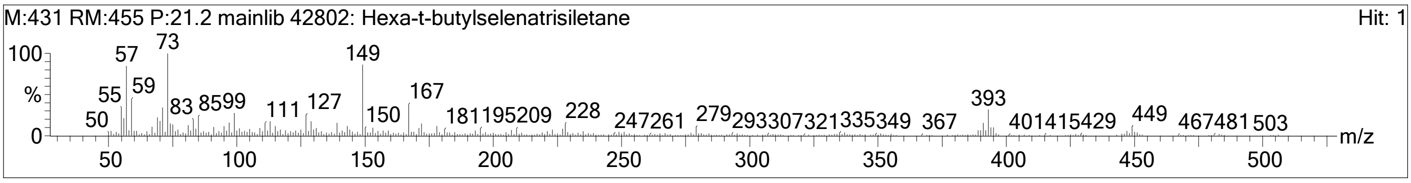

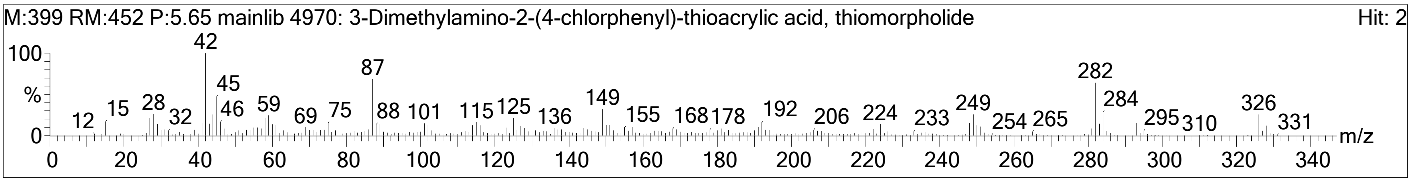

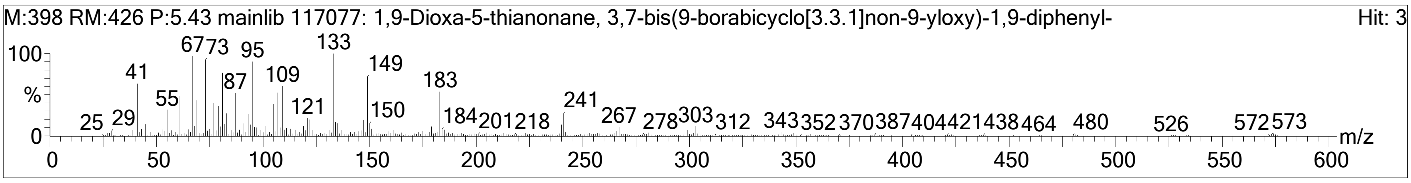

TAMILNADU AGRICULTURAL UNIVERSITY - AGRICULTURAL MICROBIOLOGY

INSTRUMENT: PERKIN ELMER CLARUS SQ8C   COLOUMN: DB-5 MS CAPILARY STANDARD NON - POLARCOLOUMN
INJECTION VOL: 1 MICRO LITER   DIMENSION: 30Mts, ID: 0.25 mm, FILM: 0.25 IM   CARRIER GAS: He
SAMPLE ID : C160

#	RT	Scan	Height	Area	Area %	Norm %
16	33.366	6071	7,920,426	360,421.4	0.754	13.78

Pk #	RT	Hit	Compound Name	Match	R.Match	Prob.	CAS	Library
16	33.366	1	á-D-Glucopyranosiduronic acid, 3-(5-ethylhexahydro-2,4,6-trioxo-5-pyrimidinyl)-1,1-dimethylpropyl 2,3,4-tris-O-(trimethylsilyl)-, methyl ester	396	434	8.9	55556-80-4	mainlib
		2	Hexadecanoic acid, 1a,2,5,5a,6,9,10,10a-octahydro-5,5a-dihydroxy-4-(hydroxymethyl)-1,1,7,9-tetramethyl-11-oxo-1H-2,8a-methanocyclopenta[a]cyclopropa[e]cyclodecen-6-yl ester, [1aR-(1aà,2à,5á,5aá,6á,8aà,9à,10aà)]-	389	389	6.8	52557-26-3	mainlib
		3	9-Octadecenoic acid (Z)-, tetradecyl ester	379	426	4.8	22393-85-7	mainlib
		4	Endrin ketone	369	435	3.4	53494-70-5	replib
		5	Dihydromorphine, 2TMS derivative	368	396	3.2		mainlib
		6	1,25-Dihydroxyvitamin D3, TMS derivative	367	502	3.1	55759-94-9	mainlib
		7	9-Octadecenoic acid, (2-phenyl-1,3-dioxolan-4-yl)methyl ester, cis-	366	425	3.0	56599-45-2	mainlib
		8	Pseduosarsasapogenin-5,20-dien	363	462	2.7		mainlib
		9	Decanoic acid, 1,1a,1b,4,4a,5,7a,7b,8,9-decahydro-4a,7b-dihydroxy-3-(hydroxymethyl)-1,1,6,8-tetramethyl-5-oxo-9aH-cyclopropa[3,4]benz[1,2-e]azulene-9,9a-diyl ester, [1aR-(1aà,1bá,4aá,7aà,7bà,8à,9á,9aà)]-	363	372	2.7	24928-17-4	replib
		10	Crinamine, 1,2-dihydro-6-hydroxy-11-oxo-	362	428	2.5		mainlib

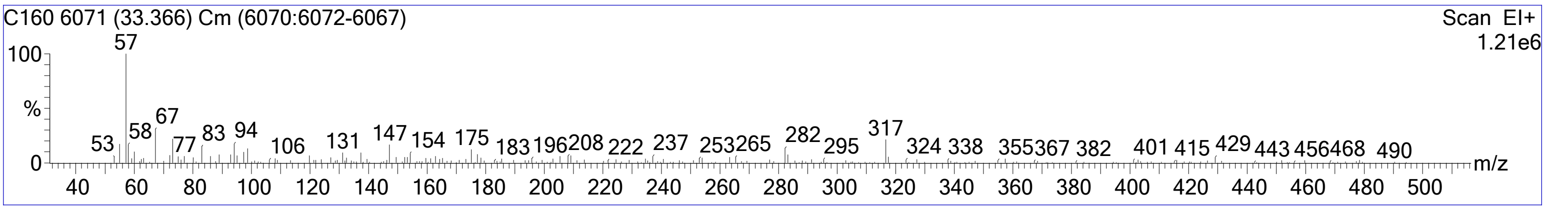

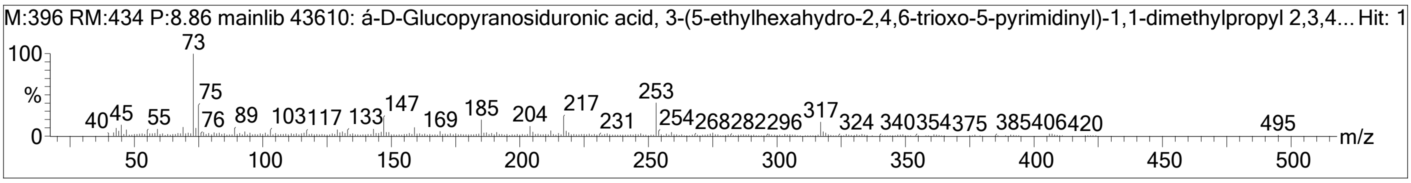

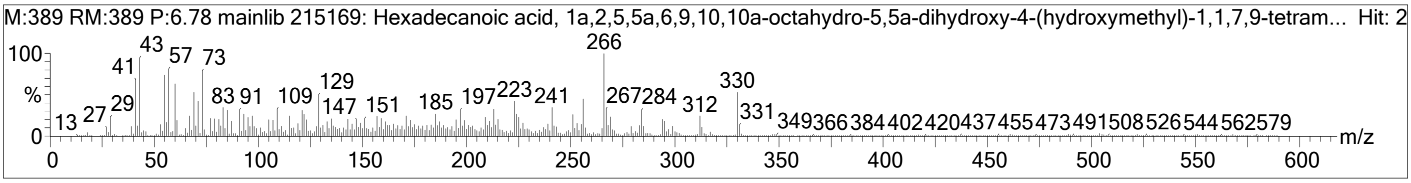

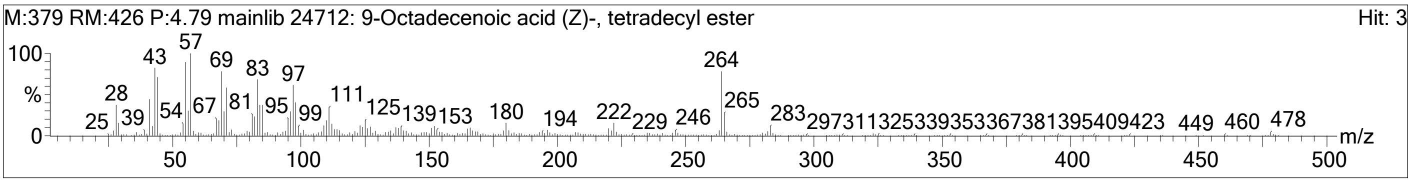

TAMILNADU AGRICULTURAL UNIVERSITY - AGRICULTURAL MICROBIOLOGY

INSTRUMENT: PERKIN ELMER CLARUS SQ8C COLOUMN: DB-5 MS CAPILARY STANDARD NON - POLARCOLOUMN
INJECTION VOL: 1 MICRO LITER DIMENSION: 30Mts, ID: 0.25 mm, FILM: 0.25 IM CARRIER GAS: He
SAMPLE ID : C160

#	RT	Scan	Height	Area	Area %	Norm %
17	33.451	6088	8,064,527	324,254.1	0.678	12.39

Pk #	RT	Hit	Compound Name	Match	R.Match	Prob.	CAS	Library
17	33.451	1	17-(1,5-Dimethylhexyl)-10,13-dimethyl-3-styrylhexadecahydrocyclopenta[a]phenanthren-2-one	400	417	11.4		mainlib
		2	Methyl glycocholate, 3TMS derivative	393	431	8.8	57326-16-6	mainlib
		3	17a-Methyl-3á-methoxy-17a-aza-D-homoandrost-5-ene-17-one	374	464	4.3	149942-10-9	mainlib
		4	2-Myristynoyl pantetheine	373	457	4.1		mainlib
		5	11à-Hydroxyprogesterone, trimethylsilyl ether, bis(O-methyloxime)	371	384	3.8		mainlib
		6	Chromone, 5-hydroxy-6,7,8-trimethoxy-2,3-dimethyl-	369	452	3.5		mainlib
		7	1,5,8-Trimethoxy-12a-methyl-1,2,3,3a,3b,4,5,6,7,8,9,10,10b,11,12,12a-hexadecahydro-benzo[3,4]cyclohepta[1,2-E]indene	367	426	3.2		mainlib
		8	9,10-Secocholesta-5,7,10(19)-triene-3,24,25-triol, (3á,5Z,7E)-	366	475	3.1	40013-87-4	mainlib
		9	Spirost-8-en-11-one, 3-hydroxy-, (3á,5à,14á,20á,22á,25R)-	364	428	2.8	58072-54-1	mainlib
		10	2-(2-Azepan-1-yl-2-oxoethyl)-1-hydroxy-1-phenyl-octahydro-pyrido[1,2-a]azepin-4-one	353	429	2.0		mainlib

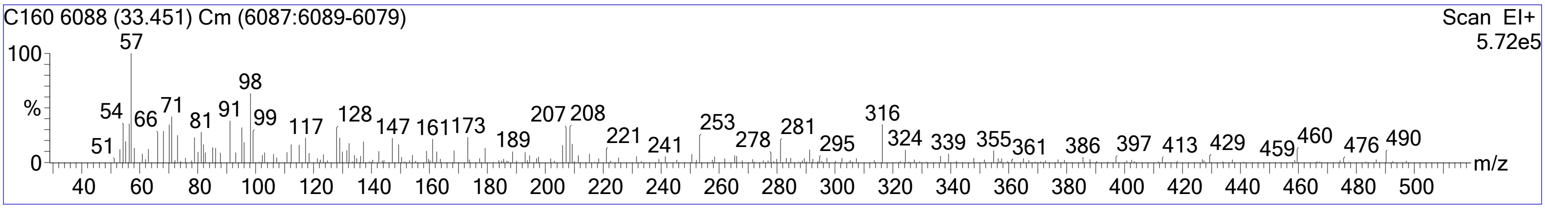

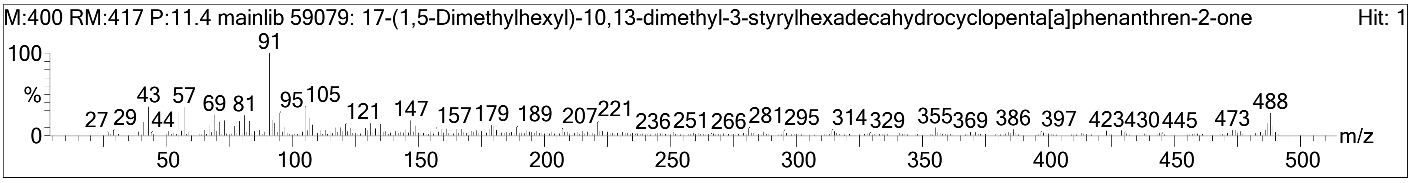

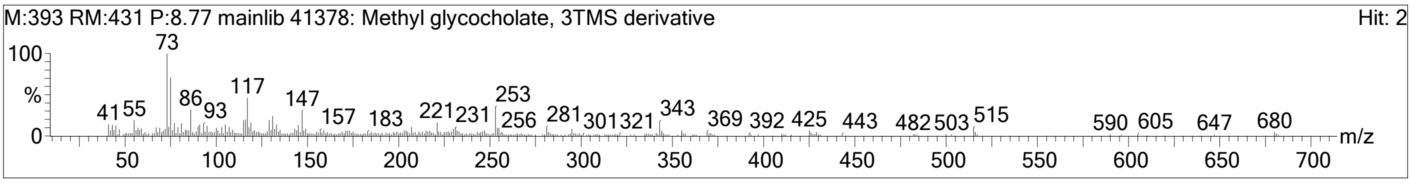

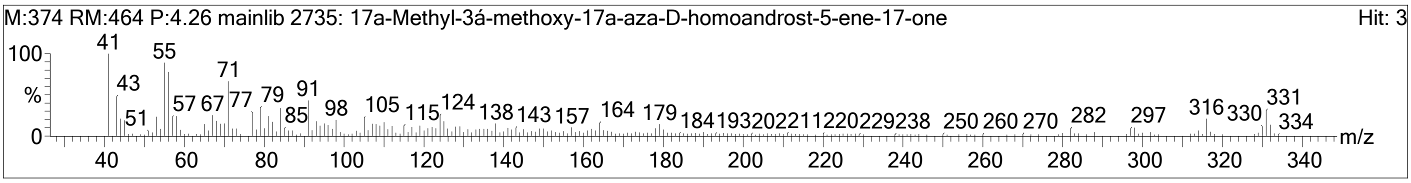

TAMILNADU AGRICULTURAL UNIVERSITY - AGRICULTURAL MICROBIOLOGY

INSTRUMENT: PERKIN ELMER CLARUS SQ8C COLOUMN: DB-5 MS CAPILARY STANDARD NON - POLARCOLOUMN
INJECTION VOL: 1 MICRO LITER DIMENSION: 30Mts, ID: 0.25 mm, FILM: 0.25 IM CARRIER GAS: He
SAMPLE ID : C160

#	RT	Scan	Height	Area	Area %	Norm %
18	33.486	6095	9,689,604	324,053.4	0.678	12.39

Pk #	RT	Hit	Compound Name	Match	R.Match	Prob.	CAS	Library
18	33.486	1	Bicyclo[5.3.0]decan-2-one, 9-(diphenylmethylene)-	370	450	7.7	345938-61-6	mainlib
		2	2-Thiophenecarboxaldehyde, 5-ethynyl-, (2,4-dinitrophenyl)hydrazone	359	460	5.2	56588-21-7	mainlib
		3	1,3-Cyclohexanedione, 2-[4-(4-methoxyphenylamino)-2-thiazolyl]-	347	448	3.5		mainlib
		4	1-(3-Bromo-2-fluoro-4-nitro-phenyl)-4-methyl-piperidine	346	430	3.4		mainlib
		5	16-Hydroxymethyleneandrost-5-en-3-ol-17-one	345	408	3.2		mainlib
		6	5-Oxo-3,4-dimethyl-2-pyrrolylideneacetothioic acid, à-(2H-3,4-dihydro-2-cyano-2,3,3-trimethylpyrrol-5-yl)-, S-t-butyl ester	340	413	2.6		mainlib
		7	Acetonitrile, 2-(1,2-dihydro-5-trifluoromethyl-1-methylpyridin-2-ylideno)-2-(2-benzimidazolyl)-	339	424	2.5	301313-00-8	mainlib
		8	8-Amino-5-[3-chlorophenylthio]-6-methoxyquinoline	339	419	2.5	64895-56-3	mainlib
		9	6-Hydroxy-powelline-N-nitroso-7-demethoxy-, aldehyde	339	416	2.5		mainlib
		10	1-Phenylpiperazine, 4-(5-amino-2-fluoro-4-nitrophenyl)-	336	393	2.2		mainlib

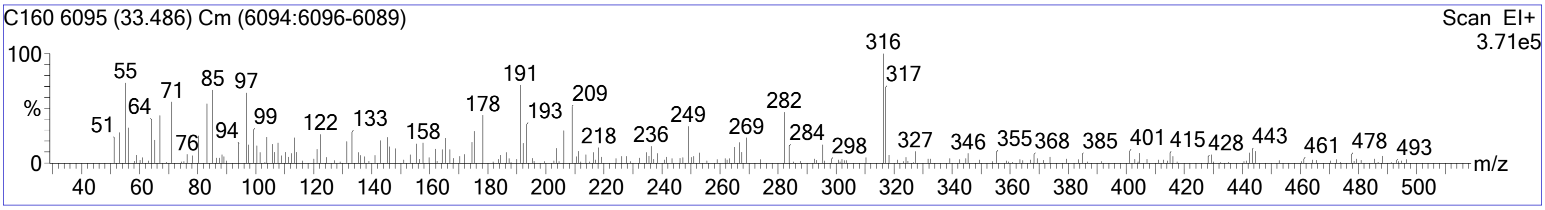

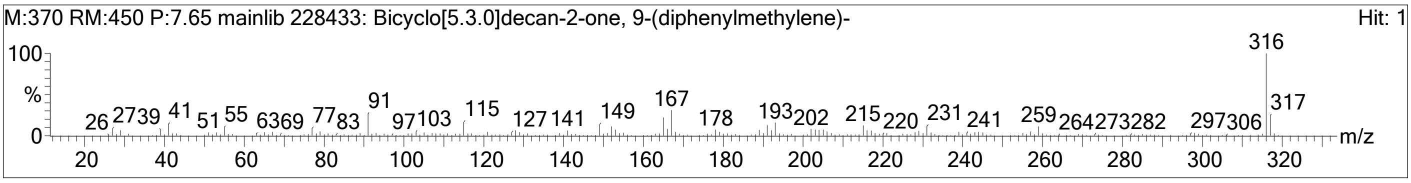

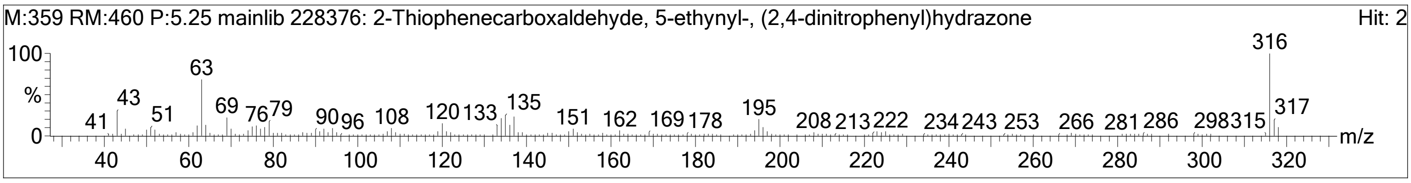

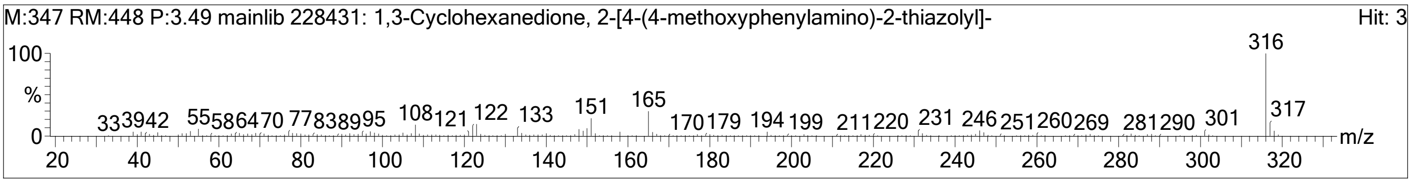

TAMILNADU AGRICULTURAL UNIVERSITY - AGRICULTURAL MICROBIOLOGY

INSTRUMENT: PERKIN ELMER CLARUS SQ8C COLOUMN: DB-5 MS CAPILARY STANDARD NON - POLARCOLOUMN
INJECTION VOL: 1 MICRO LITER DIMENSION: 30Mts, ID: 0.25 mm, FILM: 0.25 IM CARRIER GAS: He
SAMPLE ID : C160

#	RT	Scan	Height	Area	Area %	Norm %
19	33.541	6106	8,963,888	555,089.2	1.161	21.22

Pk #	RT	Hit	Compound Name	Match	R.Match	Prob.	CAS	Library
19	33.541	1	4-[4-(2-Methoxyphenyl)-1H-pyrazol-3-yl]benzene-1,3-diol	394	497	6.4		mainlib
		2	Corynan-17-ol, 18,19-didehydro-10-methoxy-, acetate (ester)	393	464	6.1	56053-13-5	mainlib
		3	Octadecane, 1,1'-[1,3-propanediylbis(oxy)]bis-	393	420	6.1	17367-38-3	mainlib
		4	5H-Cyclohepta[b]pyridine-3-carbonitrile, 6,7,8,9-tetrahydro-2-amino-4-(2-fluorophenyl)-	388	464	4.9		mainlib
		5	Corynan-17-ol, 18,19-didehydro-10-methoxy-	380	444	3.7	56053-12-4	mainlib
		6	1-(2'-Chloro-5'-sulfophenyl)-3-methyl-5-pyrazolone	371	436	2.7	88-76-6	mainlib
		7	2,3,4,5,7-Pentaaza-13-oxa-16-thiatetracyclo[7.7.0.0(2.6).0(10.15)]hexadeca-1(9),3,5,10(15)-tetraen-8-one, 7-benzyl-12,12-dimethyl-	368	451	2.4		mainlib
		8	2-(tert.-Butyldimethylsilyl)oxybenzylidene acetophenone	365	486	2.1		mainlib
		9	5-Ethoxy-2-[4-(2-methoxyphenyl)-1H-pyrazol-3-yl]phenol	365	453	2.1		mainlib
		10	1,2-Cyclobutanedicarboxamide, N,N'-diheptyl-N,N'-dimethyl-	364	433	2.0	82057-84-9	mainlib

C160 6106 (33.541) Cm (6105:6107-6099) Scan EI+

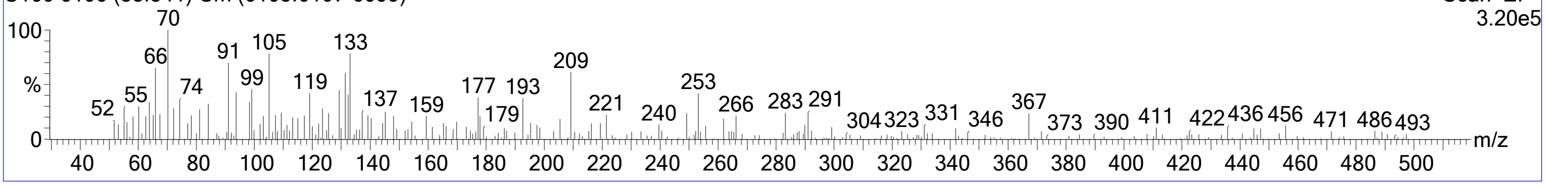

M:394 RM:497 P:6.38 mainlib 220177: 4-[4-(2-Methoxyphenyl)-1H-pyrazol-3-yl]benzene-1,3-diol Hit: 1

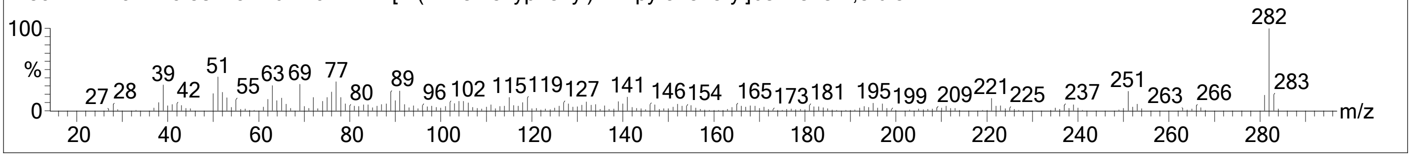

M:393 RM:464 P:6.13 mainlib 35310: Corynan-17-ol, 18,19-didehydro-10-methoxy-, acetate (ester) Hit: 2

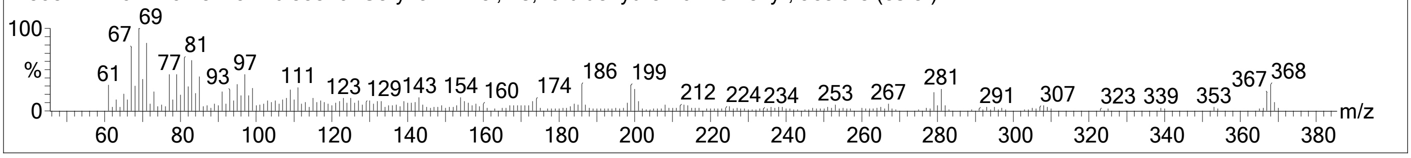

M:393 RM:420 P:6.13 mainlib 219910: Octadecane, 1,1'-[1,3-propanediylbis(oxy)]bis- Hit: 3

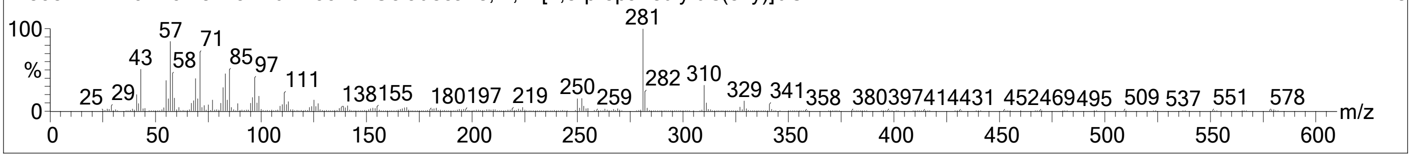

TAMILNADU AGRICULTURAL UNIVERSITY - AGRICULTURAL MICROBIOLOGY

INSTRUMENT: PERKIN ELMER CLARUS SQ8C COLOUMN: DB-5 MS CAPILARY STANDARD NON - POLARCOLOUMN
INJECTION VOL: 1 MICRO LITER DIMENSION: 30Mts, ID: 0.25 mm, FILM: 0.25 IM CARRIER GAS: He
SAMPLE ID : C160

#	RT	Scan	Height	Area	Area %	Norm %
20	33.606	6119	9,324,453	568,830.8	1.190	21.74

Pk #	RT	Hit	Compound Name	Match	R.Match	Prob.	CAS	Library
20	33.606	1	Methyl 9,11,13-octadecatrienoate, adduct with 1-methyl-1,3,4-triazolin-2,5-dione	386	460	8.7		mainlib
		2	1,8-Dioxa-5-thiaoctane, 8-(9-borabicyclo[3.3.1]non-9-yl)-3-(9-borabicyclo[3.3.1]non-9-yloxy)-1-phenyl-	370	437	5.0		mainlib
		3	Benz[e]azulene-3,8-dione, 3a,4,6a 7,9,10,10a,10b-octahydro-3a,10a-dihydroxy-5-(hydroxymethyl)-7-(1-hydroxy-1-methylethyl)-2,10-dimethyl-, [3aR-(3aà,6aà,7à,10á,10aá,10bá)]-	366	473	4.2	77590-91-1	mainlib
		4	Ethyl 5-(([3-cyano-4-(methoxymethyl)-6-methylpyridin-2-yl]sulfanyl)methyl)-1,2-oxazole-3-carboxylate	366	448	4.2		mainlib
		5	Cyclohexanecarboxamide, N-furfuryl-	362	541	3.5	6341-32-8	mainlib
		6	Benz[e]azulene-3,8-dione, 3a,4,6a,7,9,10,10a,10b-octahydro-3a,10a-dihydroxy-5-(hydroxymethyl)-2, 10-dimethyl-, (3aà,6aà,10á,10aá,10bá)-(+)-	358	459	3.0	25578-89-6	mainlib
		7	5-Cyano-4(4-methoxyphenyl)-2-(4-methylaminobutyl)-6-anilinopyrimidine	358	412	3.0		mainlib
		8	Deuteroporphyrin IX	356	411	2.8	448-65-7	nist_msms
		9	Pregn-5-en-20-one, 12-(acetyloxy)-3,8,14-trihydroxy-, (3á,12á,14á)-	353	409	2.5	55955-56-1	mainlib
		10	Deuteroporphyrin IX	353	407	2.8	448-65-7	nist_msms

C160 6119 (33.606) Cm (6118:6120-6115) Scan EI+ 2.86e5

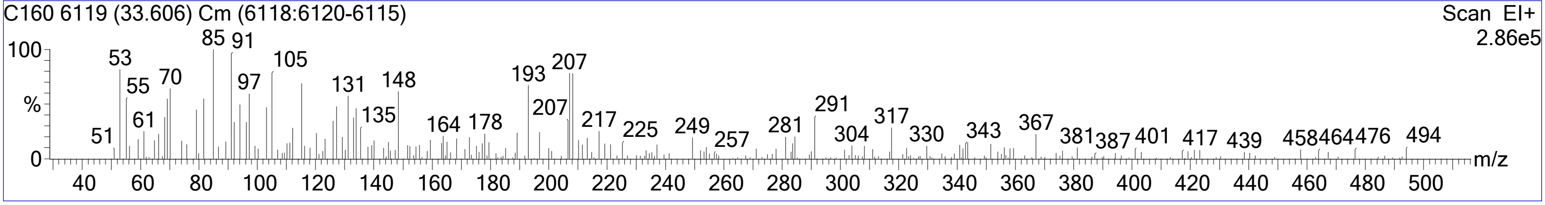

M:386 RM:460 P:8.66 mainlib 228553: Methyl 9,11,13-octadecatrienoate, adduct with 1-methyl-1,3,4-triazolin-2,5-dione Hit: 1

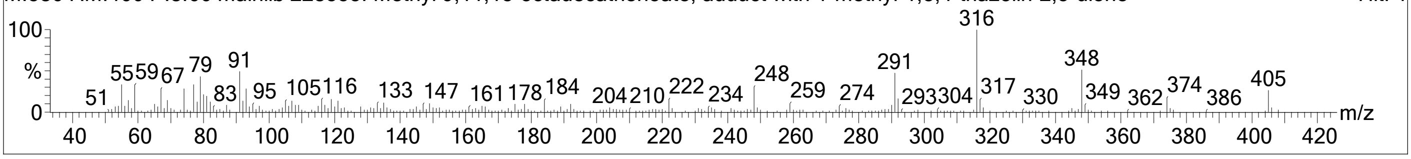

M:370 RM:437 P:4.98 mainlib 68432: 1,8-Dioxa-5-thiaoctane, 8-(9-borabicyclo[3.3.1]non-9-yl)-3-(9-borabicyclo[3.3.1]non-9-yloxy)-1-phenyl- Hit: 2

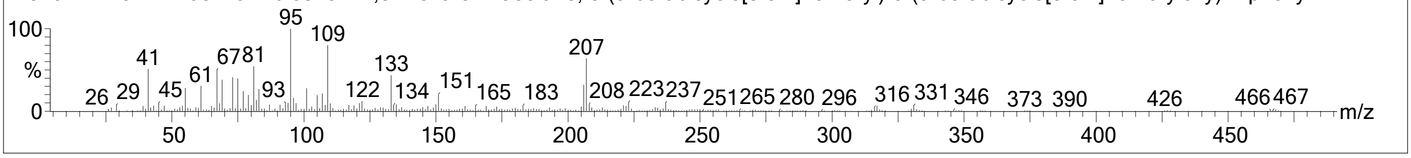

M:366 RM:473 P:4.21 mainlib 188584: Benz[e]azulene-3,8-dione, 3a,4,6a 7,9,10,10a,10b-octahydro-3a,10a-dihydroxy-5-(hydroxymethyl)-7-(... Hit: 3

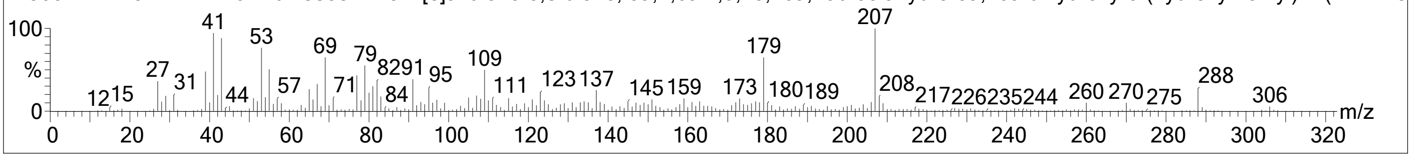

TAMILNADU AGRICULTURAL UNIVERSITY - AGRICULTURAL MICROBIOLOGY

INSTRUMENT: PERKIN ELMER CLARUS SQ8C   COLOUMN: DB-5 MS CAPILARY STANDARD NON - POLARCOLOUMN
INJECTION VOL: 1 MICRO LITER   DIMENSION: 30Mts, ID: 0.25 mm, FILM: 0.25 IM   CARRIER GAS: He
SAMPLE ID : C160

#	RT	Scan	Height	Area	Area %	Norm %
21	33.686	6135	9,527,371	269,478.8	0.564	10.30

Pk #	RT	Hit	Compound Name	Match	R.Match	Prob.	CAS	Library
21	33.686	1	9,19-Cyclolanostan-3-ol, 24,24-epoxymethano-, acetate	392	415	17.3		mainlib
		2	9,19-Cyclo-25,26-epoxyergostan-3-ol, 4,4,14-trimethyl-, acetate	366	394	5.2		mainlib
		3	Bicyclo[5.3.0]decan-2-one, 9-(diphenylmethylene)-	358	434	3.9	345938-61-6	mainlib
		4	Benzoic acid, 3,5-dicyclohexyl-4-hydroxy-, methyl ester	357	434	3.7	55125-23-0	mainlib
		5	Ethyl 5-(([3-cyano-4-(methoxymethyl)-6-methylpyridin-2-yl]sulfanyl)methyl)-1,2-oxazole-3-carboxylate	350	418	2.8		mainlib
		6	Spirostan-12-one, 3-hydroxy-, (3á,5à,25R)-	350	382	2.8	467-55-0	mainlib
		7	Sarreroside	346	421	2.4		mainlib
		8	Allo-cassaic acid methyl ester	346	411	2.4	2209-01-0	mainlib
		9	8-Amino-5-[3-chlorophenylthio]-6-methoxyquinoline	344	421	2.2	64895-56-3	mainlib
		10	Cholest-4-ene, 3á-(methoxymethoxy)-	344	367	2.2	4707-85-1	mainlib

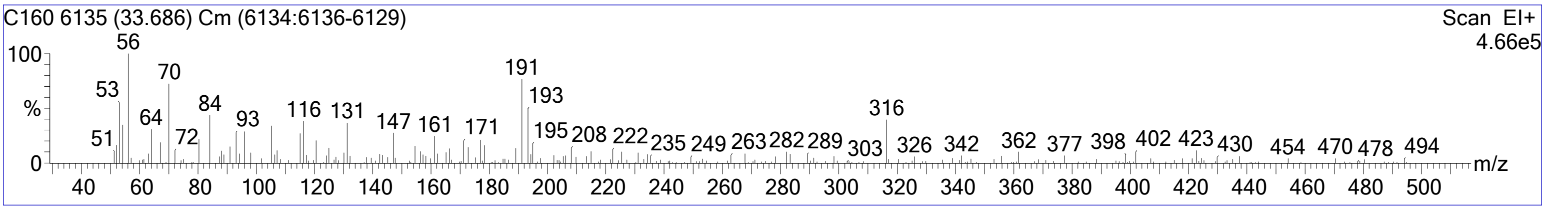

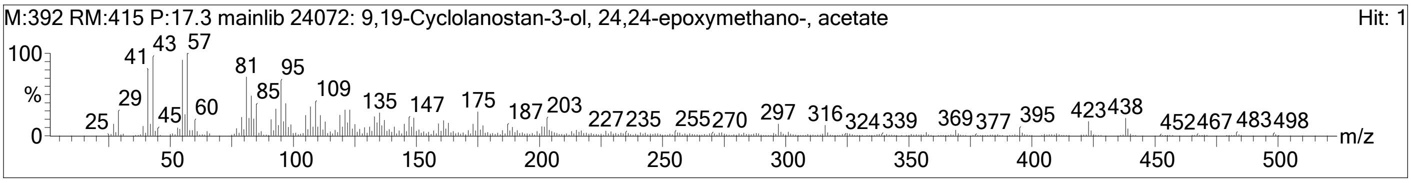

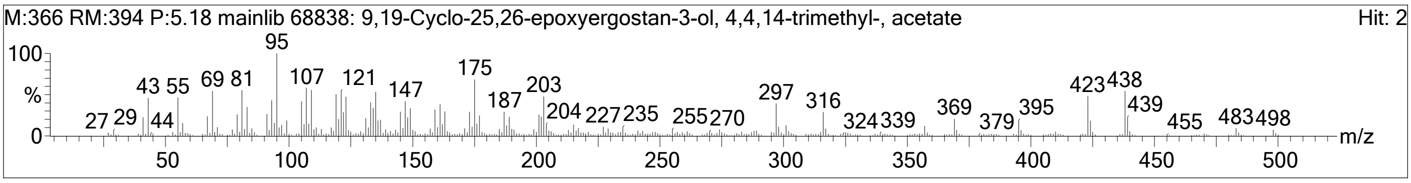

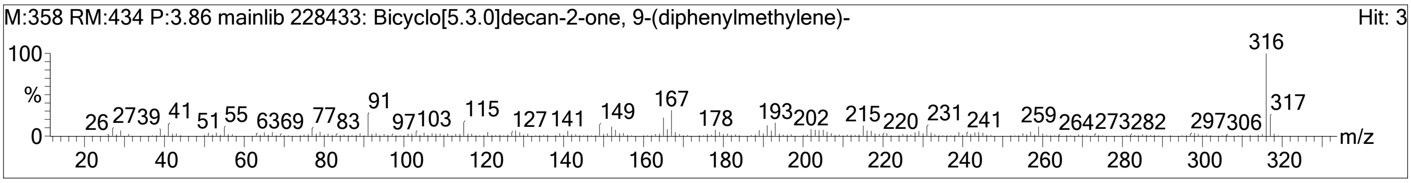

TAMILNADU AGRICULTURAL UNIVERSITY - AGRICULTURAL MICROBIOLOGY

INSTRUMENT: PERKIN ELMER CLARUS SQ8C COLOUMN: DB-5 MS CAPILARY STANDARD NON - POLARCOLOUMN
INJECTION VOL: 1 MICRO LITER DIMENSION: 30Mts, ID: 0.25 mm, FILM: 0.25 IM CARRIER GAS: He
SAMPLE ID : C160

#	RT	Scan	Height	Area	Area %	Norm %
22	33.721	6142	11,419,108	566,454.1	1.185	21.65

Pk #	RT	Hit	Compound Name	Match	R.Match	Prob.	CAS	Library
22	33.721	1	Acetic acid, 17-(4-hydroxy-5-methoxy-1,5-dimethylhexyl)-4,4,10,13,14-pentamethyl-2,3,4,5,6,7,10,11,12,13,14,15,16,17-tetradecahydrocyclopenta[a]phenanthryl ester	379	399	9.4		mainlib
		2	Dasycarpidan-1-methanol, acetate (ester)	361	435	4.8	55724-48-6	mainlib
		3	Decanoic acid, 1,1a,1b,4,4a,5,7a,7b,8,9-decahydro-4a,7b-dihydroxy-3-(hydroxymethyl)-1,1,6,8-tetramethyl-5-oxo-9aH-cyclopropa[3,4]benz[1,2-e]azulene-9,9a-diyl ester, [1aR-(1aà,1bá,4aá,7aà,7bà,8à,9á,9aà)]-	360	364	4.7	24928-17-4	replib
		4	Decanoic acid, 1,1a,1b,4,4a,5,7a,7b,8,9-decahydro-4a,7b-dihydroxy-3-(hydroxymethyl)-1,1,6,8-tetramethyl-5-oxo-9aH-cyclopropa[3,4]benz[1,2-e]azulene-9,9a-diyl ester, [1aR-(1aà,1bá,4aá,7aà,7bà,8à,9á,9aà)]-	357	374	4.7	24928-17-4	replib
		5	Docosanoic acid, 1,2,3-propanetriyl ester	356	382	3.9	18641-57-1	mainlib
		6	5H-Cyclopropa(3,4)benz(1,2-e)azulen-5-one, 1,1a-à,1b-á,4,4a,7a-à,7b,8,9,9a-decahydro-7b-à,9-á,9a-à-trihydroxy-3-hydroxymethyl-1,1,6,8-à-tetramethyl-4a-methoxy-, 9,9a-didecanoate	354	362	3.6	54870-24-5	mainlib
		7	Dihydromorphine, 2TMS derivative	350	386	3.1		mainlib
		8	4-Piperidineacetic acid, 1-acetyl-5-ethyl-2-[3-(2-hydroxyethyl)-1H-indol-2-yl]-à-methyl-, methyl ester	348	389	2.8	55724-47-5	mainlib
		9	Decanoic acid, 1,1a,1b,4,4a,5,7a,7b,8,9-decahydro-4a,7b-dihydroxy-1,1,6,8-tetramethyl-5-oxo-3-[[(1-oxodecyl)oxy]methyl]-9aH-cyclopropa[3,4]benz[1,2-e]azulene-9,9a-diyl ester, [1aR-(1aà,1bá,4aá,7aà,7bà,8à,9á,9aà)]-	348	348	2.8	20963-94-4	mainlib
		10	17-Pentatriacontene	347	368	2.7	6971-40-0	mainlib

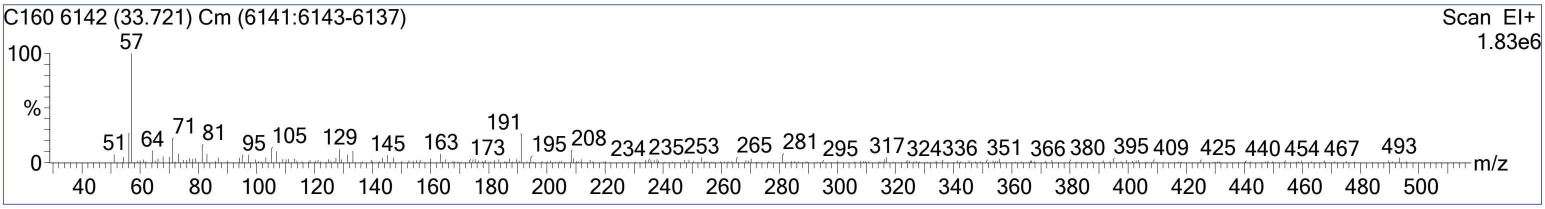

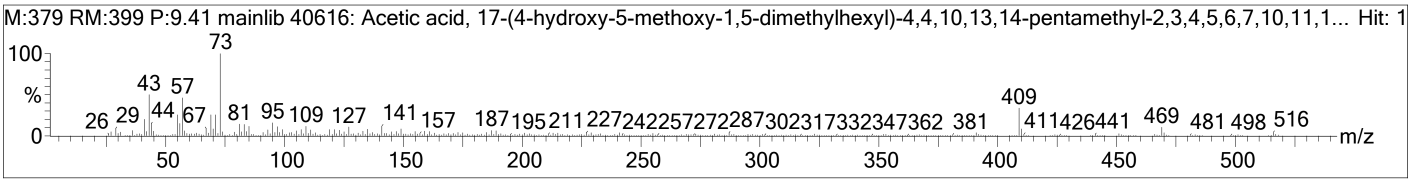

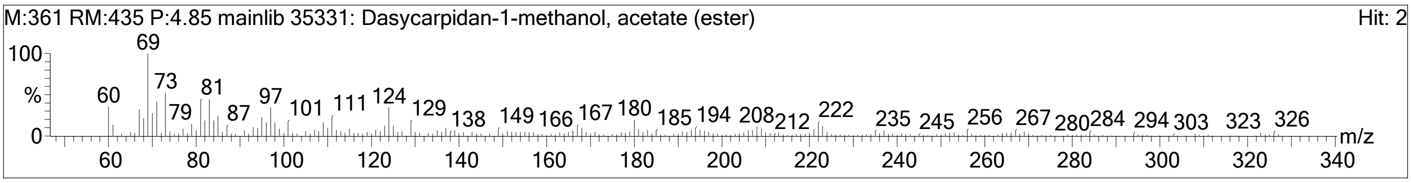

TAMILNADU AGRICULTURAL UNIVERSITY - AGRICULTURAL MICROBIOLOGY

INSTRUMENT: PERKIN ELMER CLARUS SQ8C COLOUMN: DB-5 MS CAPILARY STANDARD NON - POLARCOLOUMN
INJECTION VOL: 1 MICRO LITER DIMENSION: 30Mts, ID: 0.25 mm, FILM: 0.25 IM CARRIER GAS: He
SAMPLE ID : C160

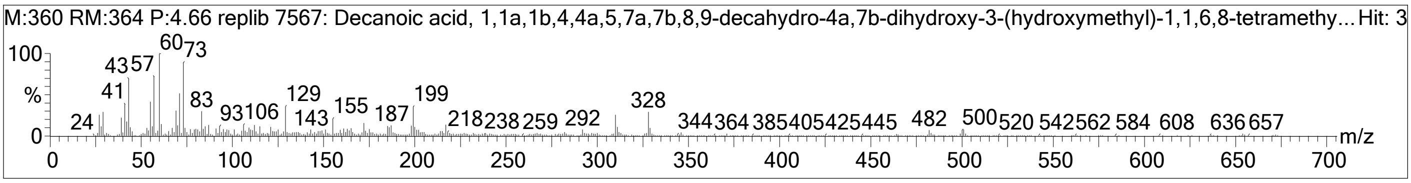

#	RT	Scan	Height	Area	Area %	Norm %
23	33.776	6153	9,068,867	197,433.5	0.413	7.55

Pk #	RT	Hit	Compound Name	Match	R.Match	Prob.	CAS	Library
23	33.776	1	Glycidyl oleate	398	520	6.0		mainlib
		2	22-Tricosenoic acid	393	449	4.8	65119-95-1	mainlib
		3	4á-Methylandrostane2,3-diol-1,17-dione	390	450	4.3		mainlib
		4	Oleic acid, eicosyl ester	385	403	3.4	22393-88-0	mainlib
		5	cis-11-Eicosenoic acid	384	478	3.3	5561-99-9	mainlib
		6	2,5-Furandione, dihydro-3-octadecyl-	384	424	3.3	47458-32-2	mainlib
		7	Oleic Acid	382	473	3.0	112-80-1	replib
		8	6-Octadecenoic acid, (Z)-	380	466	2.8	593-39-5	mainlib
		9	9-Octadecenoic acid (Z)-, octadecyl ester	377	394	2.5	17673-49-3	mainlib
		10	i-Propyl 11,12-methylene-octadecanoate	376	512	2.4		mainlib

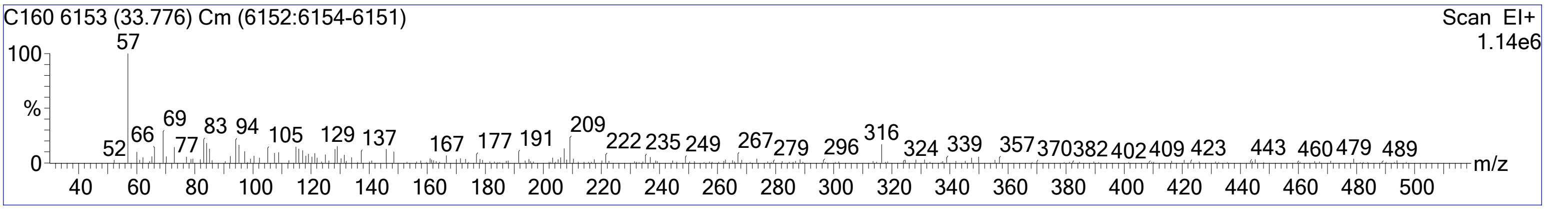

TAMILNADU AGRICULTURAL UNIVERSITY - AGRICULTURAL MICROBIOLOGY

INSTRUMENT: PERKIN ELMER CLARUS SQ8C COLOUMN: DB-5 MS CAPILARY STANDARD NON - POLARCOLOUMN
INJECTION VOL: 1 MICRO LITER DIMENSION: 30Mts, ID: 0.25 mm, FILM: 0.25 IM CARRIER GAS: He
SAMPLE ID : C160

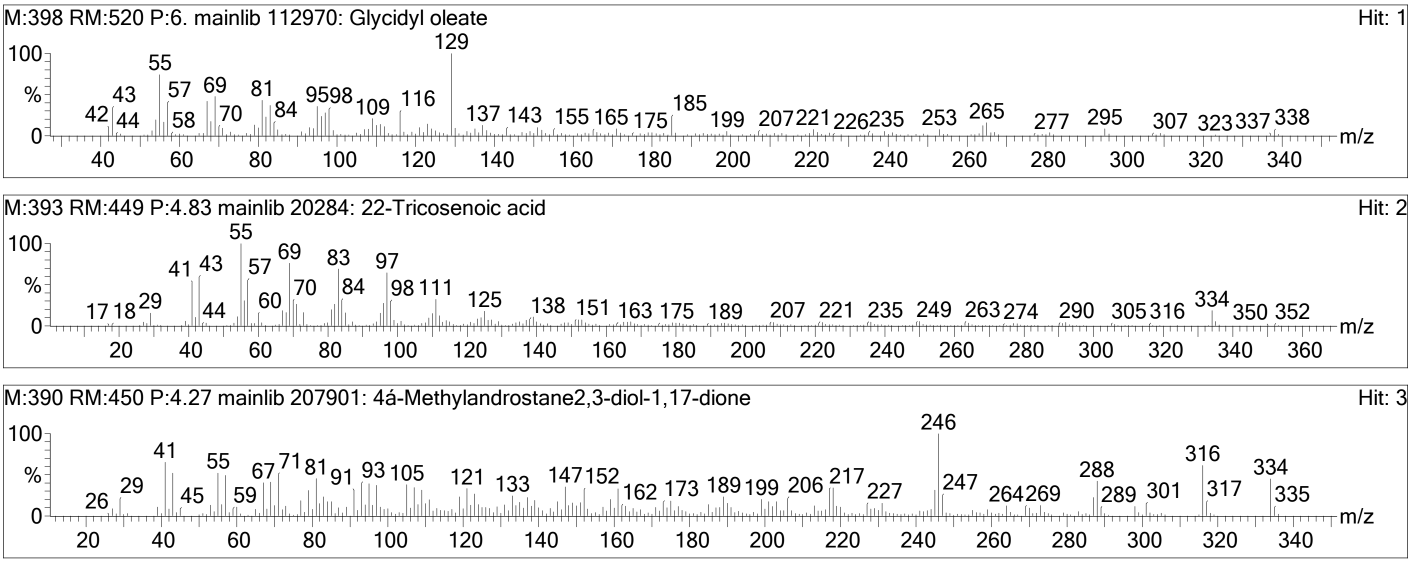

#	RT	Scan	Height	Area	Area %	Norm %
24	33.801	6158	8,937,539	150,710.5	0.315	5.76

Pk #	RT	Hit	Compound Name	Match	R.Match	Prob.	CAS	Library
24	33.801	1	Phenanthrene, 9-dodecyltetradecahydro-	421	451	6.2	55334-01-5	replib
		2	9,12,15-Octadecatrienoic acid, 2-phenyl-1,3-dioxan-5-yl ester	418	443	5.5	56700-76-6	mainlib
		3	9,9'-Biphenanthrene, octacosahydro-	414	443	4.7	55334-18-4	mainlib
		4	Milbemycin b, 13-chloro-5-demethoxy-28-deoxy-6, 28-epoxy-5-(hydroxyimino)-25-(1-methylethyl)-, (6R,13R,25R)-	411	412	4.1	107024-98-6	mainlib
		5	Trilinolein	404	422	3.2	537-40-6	mainlib
		6	Spirostan-6-ol, 3-amino-, (3á,5à,6à,25R)-	401	437	2.8	23656-00-0	mainlib

TAMILNADU AGRICULTURAL UNIVERSITY - AGRICULTURAL MICROBIOLOGY

INSTRUMENT: PERKIN ELMER CLARUS SQ8C COLOUMN: DB-5 MS CAPILARY STANDARD NON - POLARCOLOUMN
INJECTION VOL: 1 MICRO LITER DIMENSION: 30Mts, ID: 0.25 mm, FILM: 0.25 IM CARRIER GAS: He
SAMPLE ID : C160

Pk #	RT	Hit	Compound Name	Match	R.Match	Prob.	CAS	Library
		7	1-Hydroxy-2-(2,3,4,6-tetra-O-acetyl-beta-d-glucopyranosyl)-9H-xanthene-3,6,7-triyl triacetate	401	419	2.8		mainlib
		8	Cucurbitacin B, dihydro-	400	468	2.7	13201-14-4	mainlib
		9	Anthracene, 9-dodecyltetradecahydro-	399	446	2.6	55401-75-7	mainlib
		10	Anthracene, 9-dodecyltetradecahydro-	398	451	2.6	55401-75-7	replib

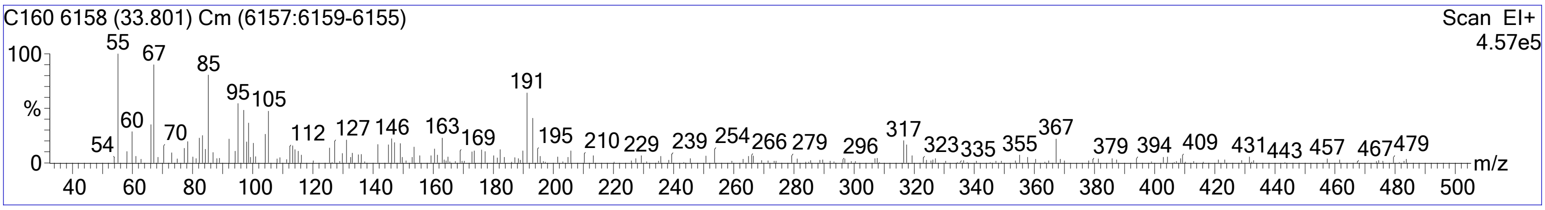

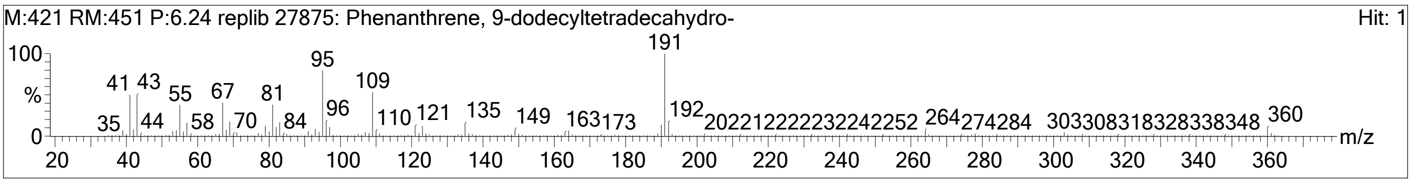

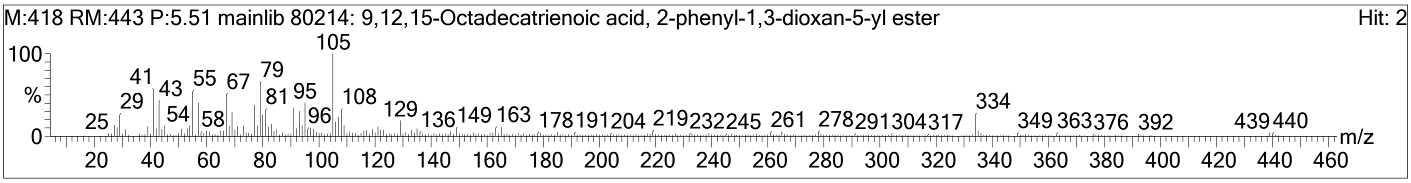

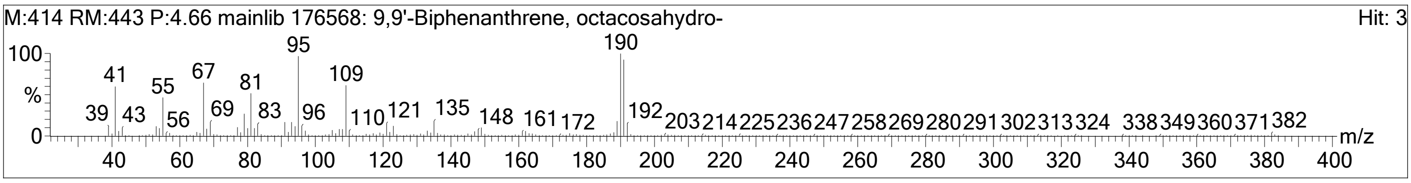

TAMILNADU AGRICULTURAL UNIVERSITY - AGRICULTURAL MICROBIOLOGY

INSTRUMENT: PERKIN ELMER CLARUS SQ8C COLOUMN: DB-5 MS CAPILARY STANDARD NON - POLARCOLOUMN
INJECTION VOL: 1 MICRO LITER DIMENSION: 30Mts, ID: 0.25 mm, FILM: 0.25 IM CARRIER GAS: He
SAMPLE ID : C160

#	RT	Scan	Height	Area	Area %	Norm %
25	33.821	6162	8,514,232	181,998.5	0.381	6.96

Pk #	RT	Hit	Compound Name	Match	R.Match	Prob.	CAS	Library
25	33.821	1	4-Acetyloxyimino-6,6-dimethyl-3-methylsulfanyl-4,5,6,7-tetrahydro-benzo[c]thiophene-1-carboxylic acid methyl ester	374	419	9.7		mainlib
		2	2,6-Di-tert-butyl-4-(3-mercaptopropyl)phenol	363	473	6.7	71728-85-3	mainlib
		3	Pseduosarsasapogenin-5,20-dien methyl ether	360	419	5.9	7604-99-1	mainlib
		4	Pseduosarsasapogenin-5-en methyl ether	360	418	5.9		mainlib
		5	5à-Pregn-16-en-20-one, 3á,12à-dihydroxy-, diacetate	357	379	5.2	5767-82-8	mainlib
		6	5,12-Naphthacenedione, 8-ethyl-7,8,9,10-tetrahydro-1,6,8,11-tetrahydroxy-	354	419	4.6	4877-81-0	mainlib
		7	Diosgenin	352	422	4.2	512-04-9	mainlib
		8	Hexahydropyridine, 1-acetyl-4-[4-hydroxy-3-methoxyphenyl]-	351	437	4.1	94427-40-4	mainlib
		9	Isoquinoline, 1,2,3,4-tetrahydro-5,6,7,8-tetramethoxy-	339	414	2.7	82261-00-5	mainlib
		10	(6,6-Dimethoxy-3a,6a-dimethyl-2-oxooctahydrocyclopenta[b]pyrrol-3-yl)acetic acid, methyl ester	337	422	2.5		mainlib

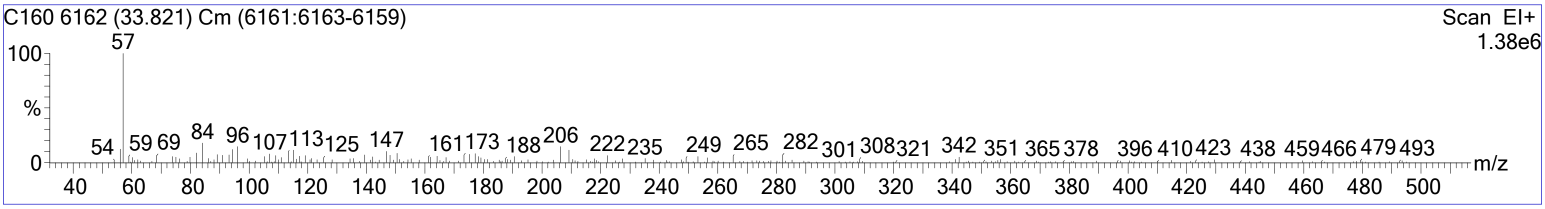

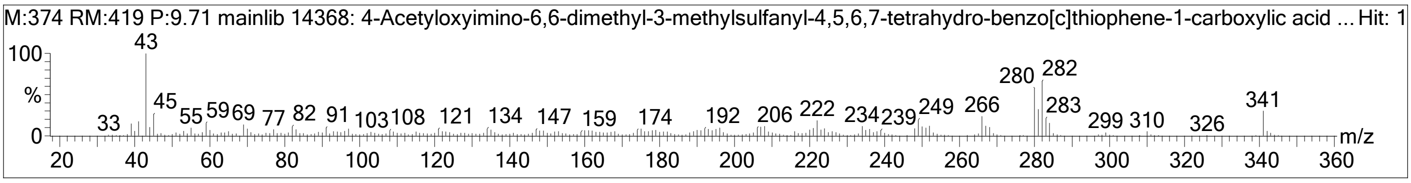

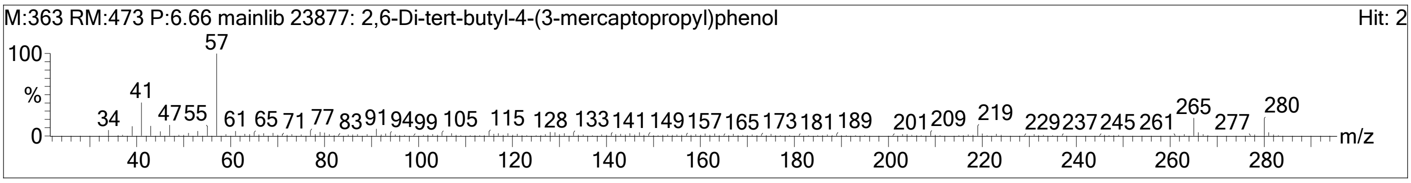

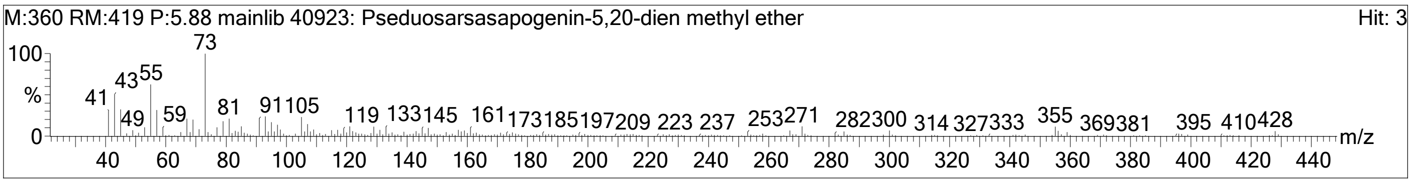

TAMILNADU AGRICULTURAL UNIVERSITY - AGRICULTURAL MICROBIOLOGY

INSTRUMENT: PERKIN ELMER CLARUS SQ8C   COLOUMN: DB-5 MS CAPILARY STANDARD NON - POLARCOLOUMN
INJECTION VOL: 1 MICRO LITER   DIMENSION: 30Mts, ID: 0.25 mm, FILM: 0.25 IM   CARRIER GAS: He
SAMPLE ID : C160

#	RT	Scan	Height	Area	Area %	Norm %
26	33.851	6168	9,400,841	199,050.9	0.416	7.61

Pk #	RT	Hit	Compound Name	Match	R.Match	Prob.	CAS	Library
26	33.851	1	9,12-Octadecadienoic acid, 2-phenyl-1,3-dioxan-5-yl ester, cis-	381	393	8.5	56687-50-4	mainlib
		2	N-(2-Phenylethyl)undeca-(2Z,4E)-diene-8,10-diynamide	368	459	5.5	99615-80-2	mainlib
		3	7,7-Dimethoxy-2,3,4,5,6,7-hexahydro-1H-cyclopenta[a]pentalene	364	489	4.6	58866-66-3	mainlib
		4	Leukotriene C4	351	514	3.0	72025-60-6	nist_msms
		5	Aspidospermidine-3-carboxylic acid, 6,7-didehydro-3,4-dihydroxy-16-methoxy-, methyl ester, (2á,3á,4á,5à,12á,1 9à)-	351	387	3.0	101043-53-2	mainlib
		6	Cholest-2-en-1-ol	350	402	2.9		mainlib
		7	Acetic acid, 8,9-dichloro-2-oxa-6-thia-adamantan-4-yl ester	349	429	2.8		mainlib
		8	Ethanone, 2-(5-methoxy-1-methyl-2-benzimidazolyl)thio-1-phenyl-	347	497	2.5	302929-97-1	mainlib
		9	9-Oxo-15S-hydroxy-5Z,8(12),13E,17Z-prostatetraenoic acid	345	510	2.3	36614-32-1	nist_msms
		10	Allocholesterol	341	385	2.0	517-10-2	mainlib

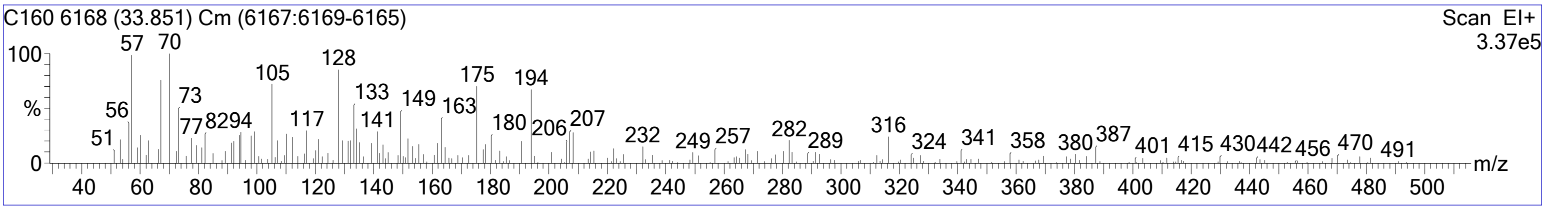

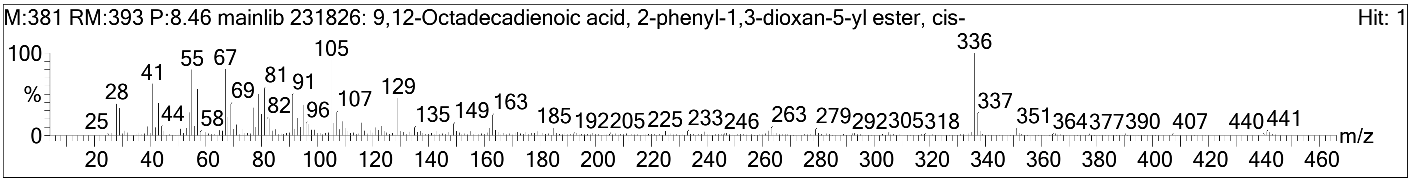

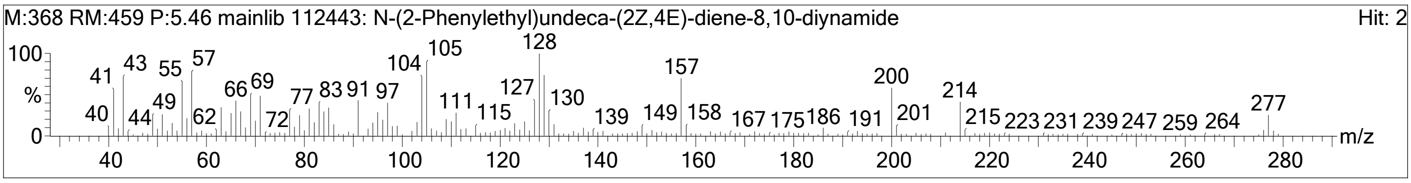

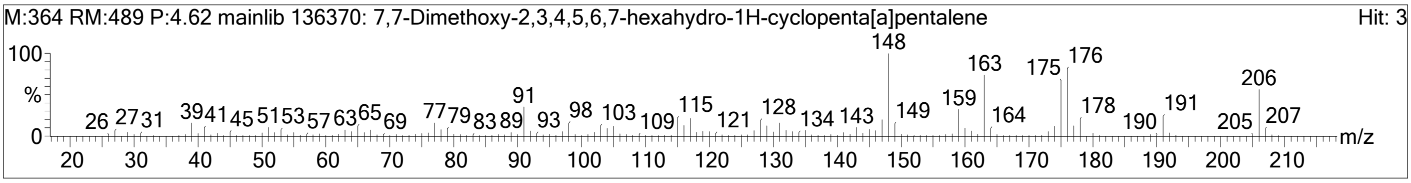

TAMILNADU AGRICULTURAL UNIVERSITY - AGRICULTURAL MICROBIOLOGY

INSTRUMENT: PERKIN ELMER CLARUS SQ8C COLOUMN: DB-5 MS CAPILARY STANDARD NON - POLARCOLOUMN
INJECTION VOL: 1 MICRO LITER DIMENSION: 30Mts, ID: 0.25 mm, FILM: 0.25 IM CARRIER GAS: He
SAMPLE ID : C160

#	RT	Scan	Height	Area	Area %	Norm %
27	33.886	6175	8,397,659	150,137.6	0.314	5.74

Pk #	RT	Hit	Compound Name	Match	R.Match	Prob.	CAS	Library
27	33.886	1	3-Desoxo-3,16-dihydroxy-12-desoxyphorbol 3,13, 16,20-tetraacetate	394	424	8.8		mainlib
		2	Glycyl-L-histidyl-L-lysine acetate	377	441	4.8	72957-37-0	mainlib
		3	Dexamethasone-21-acetate	374	388	4.3	1177-87-3	mainlib
		4	1-Oxo-forskolin	369	459	3.4		mainlib
		5	Benzoic acid, 3,5-dicyclohexyl-4-hydroxy-, methyl ester	369	435	3.4	55125-23-0	mainlib
		6	2,4-Imidazolidinedione, 5-[3,4-bis[(trimethylsilyl)oxy]phenyl]-3-methyl-5-phenyl-1-(trimethylsilyl)-	368	382	3.3	55517-85-6	mainlib
		7	Benz[e]azulen-3(3aH)-one, 4,6a,7,8,9,10,10a,10b-octahydro-3a,8,10a-trihydroxy-5-(hydroxymethyl)-2,10-dimethyl-, [3aR-(3aà,6aà,8á,10á,10aá,10bá)]-	367	443	3.2	77573-30-9	mainlib
		8	2,6,10,14,18,22-Tetracosahexaene, 2,6,10,15,19, 23-hexamethyl-, (all-E)-, didehydro deriv.	367	403	3.2	11051-27-7	mainlib
		9	5-á-Card-20(22)-enolide, 7-á,8-epoxy-3-á,11-à,14-trihydroxy-12-oxo-	362	454	2.5	22146-03-8	mainlib
		10	Benzaldehyde, 3-nitro-, (2-methyl-6-(1-piperidyl)-pyrimid-4-yl)hydrazone	361	443	2.5	330992-55-7	mainlib

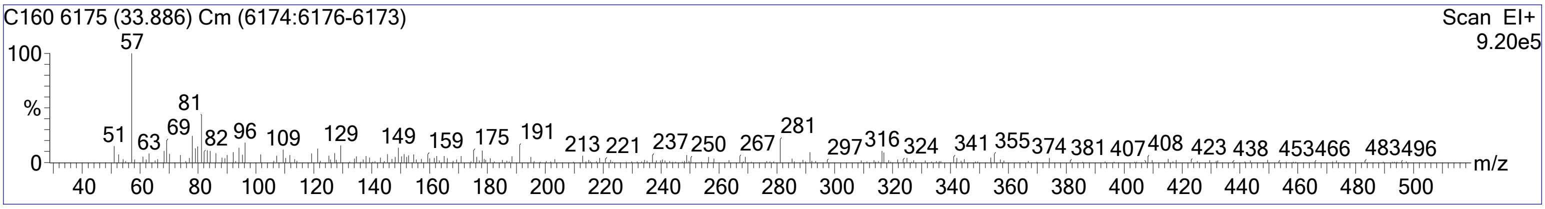

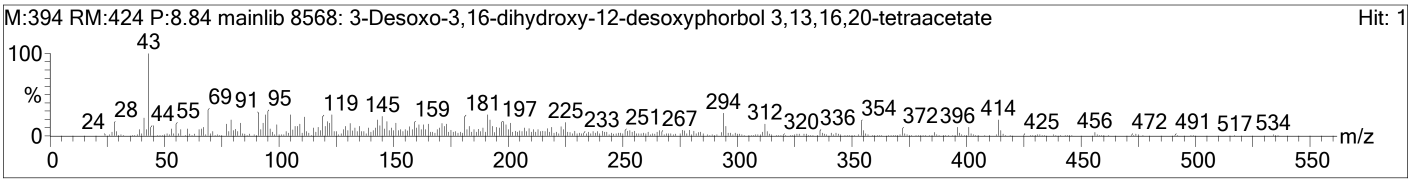

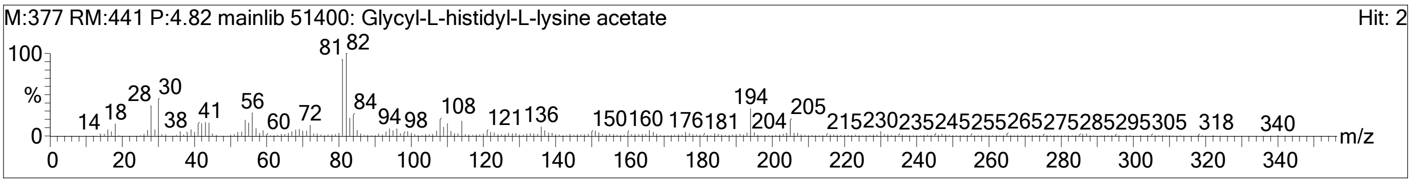

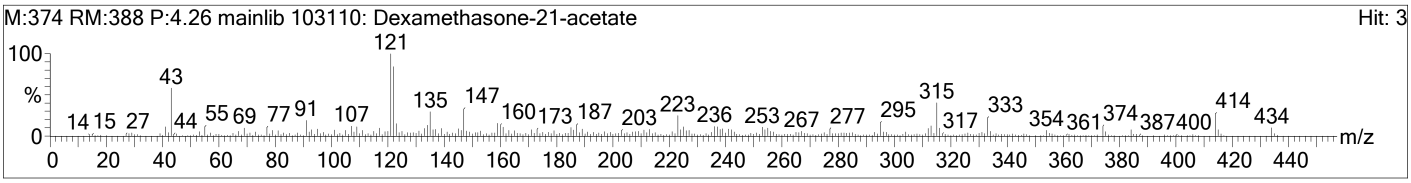

TAMILNADU AGRICULTURAL UNIVERSITY - AGRICULTURAL MICROBIOLOGY

INSTRUMENT: PERKIN ELMER CLARUS SQ8C COLOUMN: DB-5 MS CAPILARY STANDARD NON - POLARCOLOUMN
INJECTION VOL: 1 MICRO LITER DIMENSION: 30Mts, ID: 0.25 mm, FILM: 0.25 IM CARRIER GAS: He
SAMPLE ID : C160

#	RT	Scan	Height	Area	Area %	Norm %
28	33.911	6180	7,196,684	285,180.1	0.597	10.90

Pk #	RT	Hit	Compound Name	Match	R.Match	Prob.	CAS	Library
28	33.911	1	1H-Cyclopropa[3,4]benz[1,2-e]azulene-3-carboxaldehyde, 9a-(acetyloxy)-1a,1b,4,4a,5,7a,7b,8,9,9a-decahydro-4a,7b-dihydroxy-1,1,6,8-tetramethyl-5,9-dioxo-, [1aR-(1aà,1bá,4aá,7aà,7bà,8à,9aà)]-	381	409	9.9	77573-49-0	mainlib
		2	5-Methoxy-2-[4-(4-methoxyphenyl)-1H-pyrazol-3-yl]phenol	372	478	7.2		mainlib
		3	Octadecanoic acid, 2-(octadecyloxy)ethyl ester	365	401	5.5	28843-25-6	mainlib
		4	1H-Cyclopenta[a]phenanthrene-7-carboxylic acid, 2,3,6,7,8,9,10,11,12,13,14,15,16,17-tetradecahydro-10,13-dimethyl-3-oxo-17,2'-spiro(5-oxotetrahydrofuran)-, ethyl ester	361	383	4.7		mainlib
		5	Aldrin	360	391	4.5	309-00-2	replib
		6	Spirost-8-en-11-one, 3-hydroxy-, (3á,5à,14á,20á,22á,25R)-	354	407	3.5	58072-54-1	mainlib
		7	Aldrin	353	390	4.5	309-00-2	replib
		8	Aldrin	353	386	4.5	309-00-2	replib
		9	9-Ethyl-3-hydroxy-10,11b-dimethyl-1,2,3,4,6,6a,6b,7,8,9,11a,11b-dodecahydrobenzo[a]fluoren-11-one	352	437	3.2		mainlib
		10	Aspidofractinine-1-carboxaldehyde, 17-methoxy-3-oxo-, (2à,5à)-	351	398	3.1	55724-67-9	mainlib

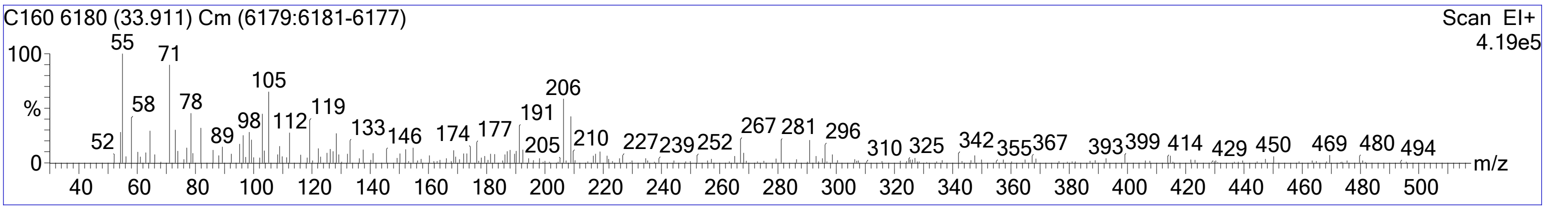

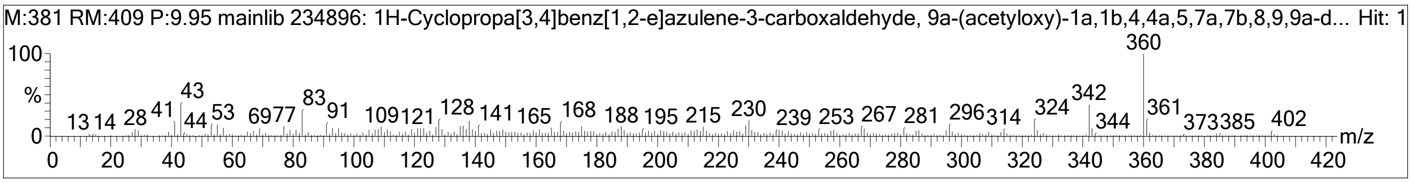

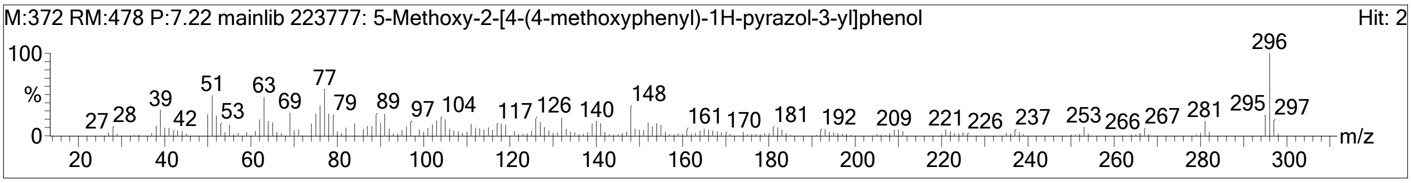

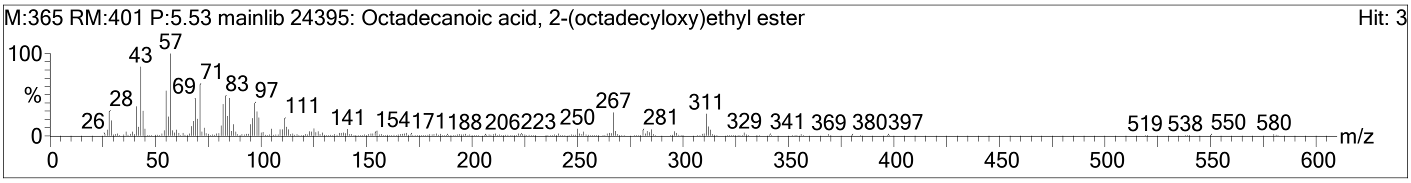

TAMILNADU AGRICULTURAL UNIVERSITY - AGRICULTURAL MICROBIOLOGY

INSTRUMENT: PERKIN ELMER CLARUS SQ8C COLOUMN: DB-5 MS CAPILARY STANDARD NON - POLARCOLOUMN
INJECTION VOL: 1 MICRO LITER DIMENSION: 30Mts, ID: 0.25 mm, FILM: 0.25 IM CARRIER GAS: He
SAMPLE ID : C160

#	RT	Scan	Height	Area	Area %	Norm %
29	33.981	6194	5,052,044	162,386.7	0.340	6.21

Pk #	RT	Hit	Compound Name	Match	R.Match	Prob.	CAS	Library
29	33.981	1	Spirost-8-en-11-one, 3-hydroxy-, (3á,5à,14á,20á, 22á,25R)-	415	474	9.3	58072-54-1	mainlib
		2	Acetic acid, 17-acetoxy-4,4,10,13-tetramethyl-7-oxo-2,3,4,7,8,9,10,11,12,13,14,15,16,17-tetradecahydro-1H-cyclopenta[a]phenanthren-3-yl (ester)	405	424	6.5		mainlib
		3	Octadecanoic acid, 2-(octadecyloxy)ethyl ester	396	433	4.8	28843-25-6	mainlib
		4	Octadecane, 1-[2-(hexadecyloxy)ethoxy]-	393	437	4.2	17367-10-1	mainlib
		5	Octadecanoic acid, 4-hydroxy-, methyl ester	391	456	3.9	2420-38-4	mainlib
		6	Stearic acid, 3-(octadecyloxy)propyl ester	390	414	3.7	17367-40-7	mainlib
		7	Thiocolchicine	389	444	3.6		mainlib
		8	1H-Cyclopropa[3,4]benz[1,2-e]azulene-3-carboxaldehyde, 9a-(acetyloxy)-1a,1b,4,4a,5,7a, 7b,8,9,9a-decahydro-4a,7b-dihydroxy-1,1,6,8-tetramethyl-5,9-dioxo-, [1aR-(1aà,1bá,4aá,7aà,7bà, 8à,9aà)]-	389	410	3.6	77573-49-0	mainlib
		9	Hexadecanoic acid, 2-(octadecyloxy)ethyl ester	385	431	3.0	29899-13-6	mainlib
		10	9-Ethyl-3-hydroxy-10,11b-dimethyl-1,2,3,4,6,6a,6b, 7,8,9,11a,11b-dodecahydrobenzo[a]fluoren-11-one	384	447	2.9		mainlib

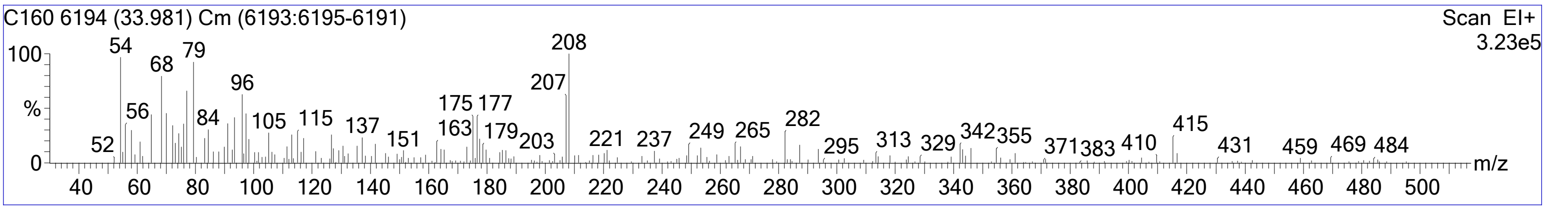

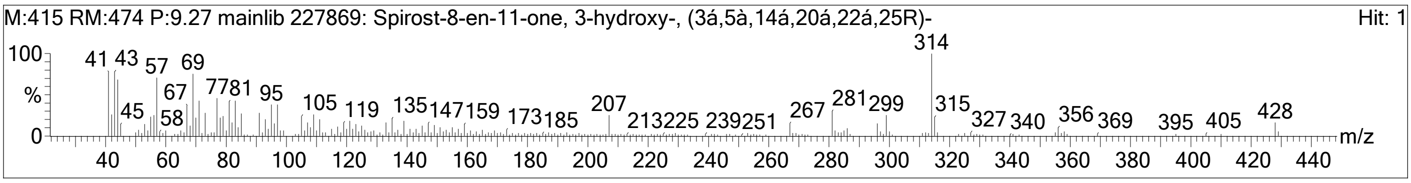

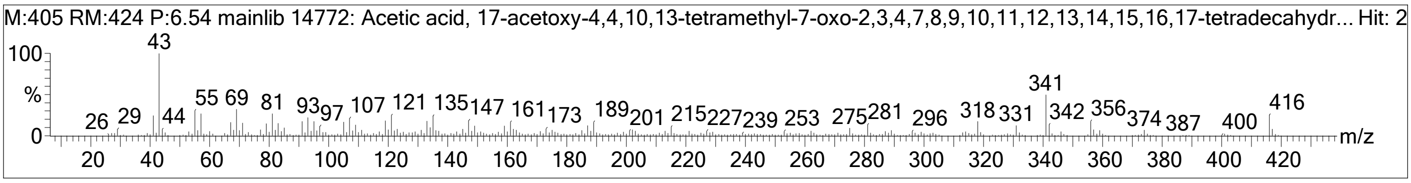

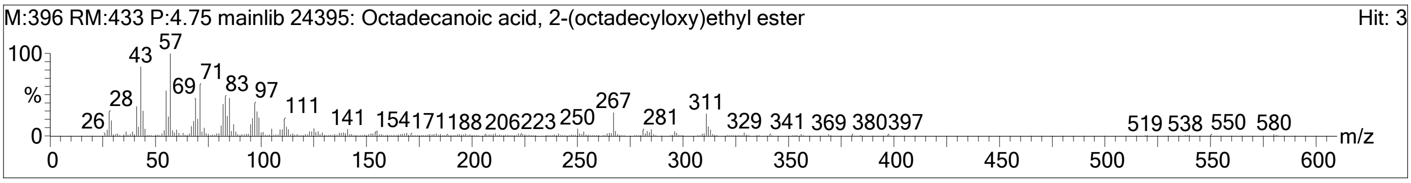

TAMILNADU AGRICULTURAL UNIVERSITY - AGRICULTURAL MICROBIOLOGY

INSTRUMENT: PERKIN ELMER CLARUS SQ8C COLOUMN: DB-5 MS CAPILARY STANDARD NON - POLARCOLOUMN
INJECTION VOL: 1 MICRO LITER DIMENSION: 30Mts, ID: 0.25 mm, FILM: 0.25 IM CARRIER GAS: He
SAMPLE ID : C160

#	RT	Scan	Height	Area	Area %	Norm %
30	34.126	6223	4,792,302	158,952.8	0.333	6.08

Pk #	RT	Hit	Compound Name	Match	R.Match	Prob.	CAS	Library
30	34.126	1	3,8,12-Tri-O-acetoxy-7-desoxyingol-7-one	406	442	9.5		mainlib
		2	2-Nonadecanone 2,4-dinitrophenylhydrazine	396	426	6.7	28813-61-8	mainlib
		3	1-(3-Cyano-4,5,6,7-tetrahydro-2-benzo[b]thienyl)-3-(3,4-dimethoxycinnamoyl)-2-thiourea	393	525	5.9		mainlib
		4	3,9-Epoxypregn-16-en-14-ol-20-one, 11,18-diacetoxy-3-methoxy-	393	407	5.9		mainlib
		5	3-Desoxo-3,16-dihydroxy-12-desoxyphorbol 3,13,16,20-tetraacetate	392	421	5.7		mainlib
		6	4a,7a-Epoxy-5H-cyclopenta[a]cyclopropa[f]cycloundecene-2,4,7,10,11-pentol, 1,1a,2,3,4,6,7,10,11,11a-decahydro-1,1,3,6,9-pentamethyl-, 2,7,10,11-tetraacetate	386	412	4.5	51950-35-7	mainlib
		7	Dihydromorphine, 2TMS derivative	383	411	4.0		mainlib
		8	9-Desoxo-9-x-acetoxy-3,8,12-tri-O-acetylingol	380	416	3.5		mainlib
		9	9-Desoxo-9-x-acetoxy-3-desoxy-7.8.12-tri-O-acetylingol-3-one	377	416	3.1		mainlib
		10	7,8,12-Tri-O-acetyl-3-desoxy-ingol-3-one	377	395	3.1		mainlib

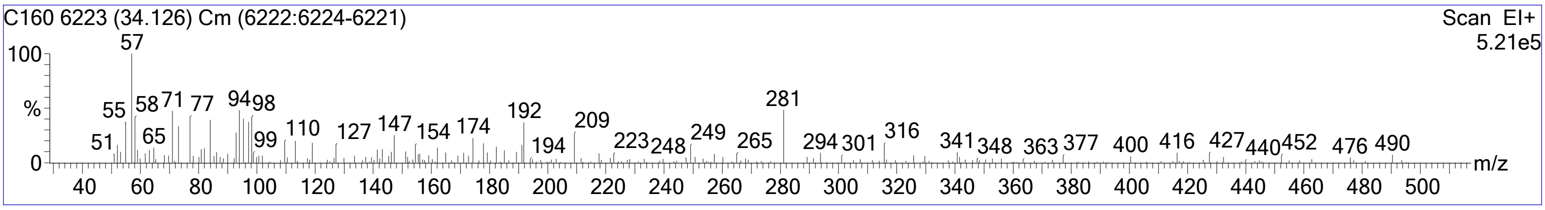

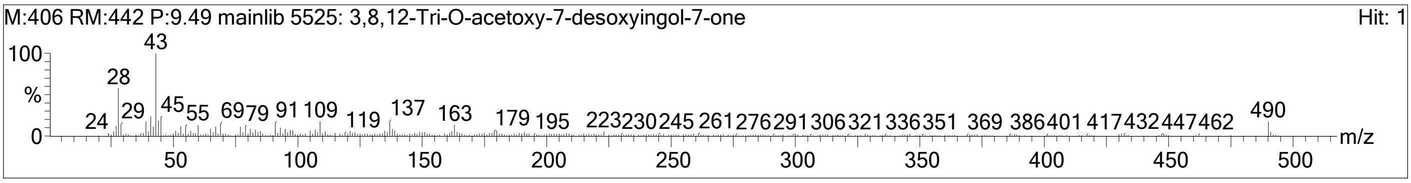

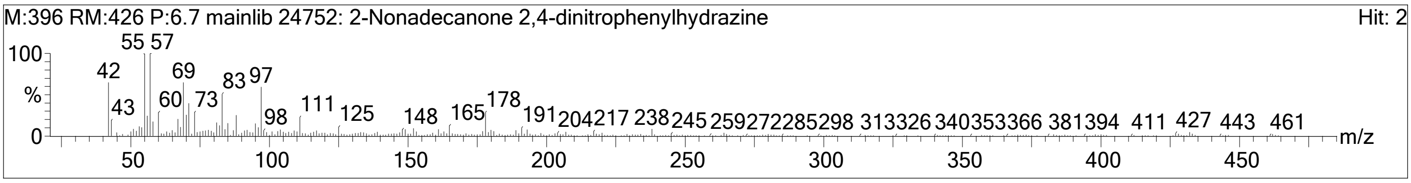

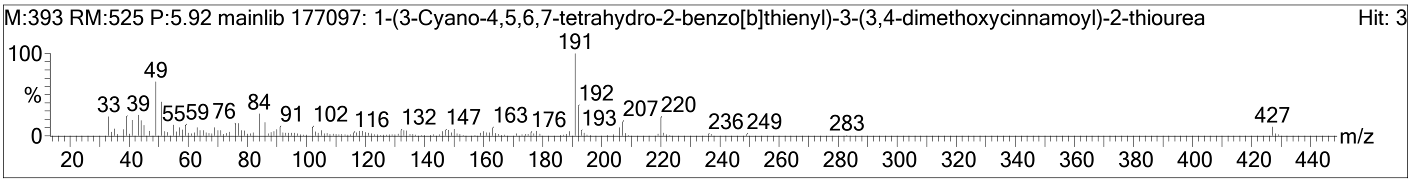

Supplement: S2 Fig — (PDF) [file pone.0219014.s008.pdf]
