# Supplementary material for: Enhancement of disease resistance, growth potential, and photosynthesis in tomato (Solanum lycopersicum) by inoculation with an endophytic actinobacterium, Streptomyces thermocarboxydus strain BPSAC147
Source: PLoS One. 2019 Jul 3;14(7):e0219014. doi: 10.1371/journal.pone.0219014 (PMC6608948; doi:10.1371/journal.pone.0219014)

TAMILNADU AGRICULTURAL UNIVERSITY - AGRICULTURAL MICROBIOLOGY

INSTRUMENT: PERKIN ELMER CLARUS SQ8C COLOUMN: DB-5 MS CAPILARY STANDARD NON - POLARCOLOUMN
INJECTION VOL: 1 MICRO LITER DIMENSION: 30Mts, ID: 0.25 mm, FILM: 0.25 IM CARRIER GAS: He
SAMPLE ID : TI60

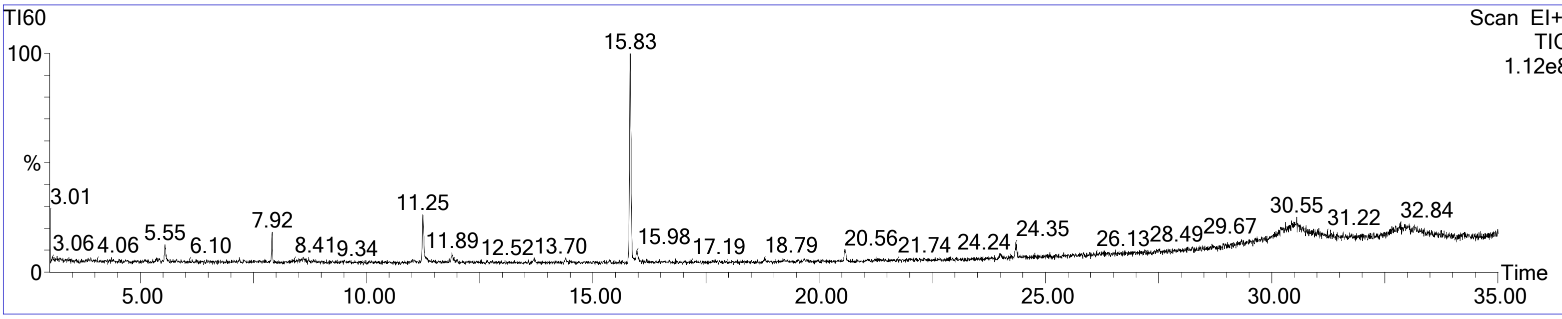

#	RT	Scan	Height	Area	Area %	Norm %
1	5.549	510	9,568,335	400,684.2	0.643	9.31

Pk #	RT	Hit	Compound Name	Match	R.Match	Prob.	CAS	Library
1	5.549	1	Benzaldehyde, 4-methyl-	659	909	24.9	104-87-0	replib
		2	Benzaldehyde, 4-methyl-	653	916	24.9	104-87-0	replib
		3	Benzaldehyde, 2-methyl-	649	909	17.5	529-20-4	mainlib
		4	Benzaldehyde, 4-methyl-	647	914	24.9	104-87-0	mainlib
		5	Benzaldehyde, 3-methyl-	643	897	13.8	620-23-5	replib
		6	Benzaldehyde, 2-methyl-	642	903	17.5	529-20-4	replib
		7	Benzaldehyde, 4-methyl-	641	917	24.9	104-87-0	replib
		8	Benzaldehyde, 3-methyl-	640	915	13.8	620-23-5	replib
		9	Benzaldehyde, 4-methyl-	637	901	24.9	104-87-0	replib
		10	Bicyclo[4.2.0]octa-1,3,5-trien-7-ol	635	878	10.3	35447-99-5	mainlib

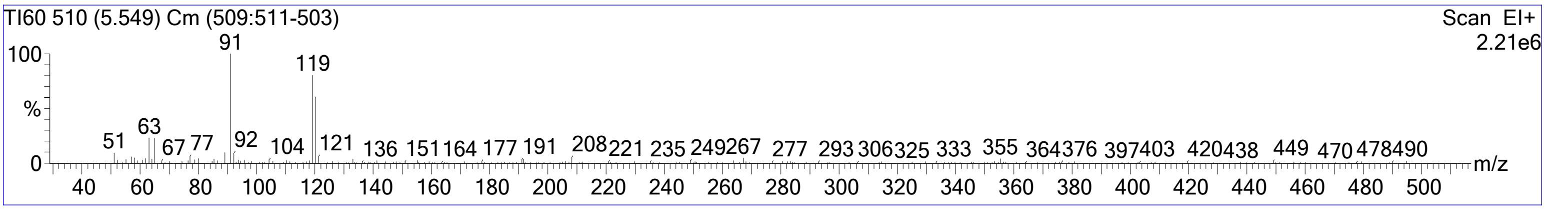

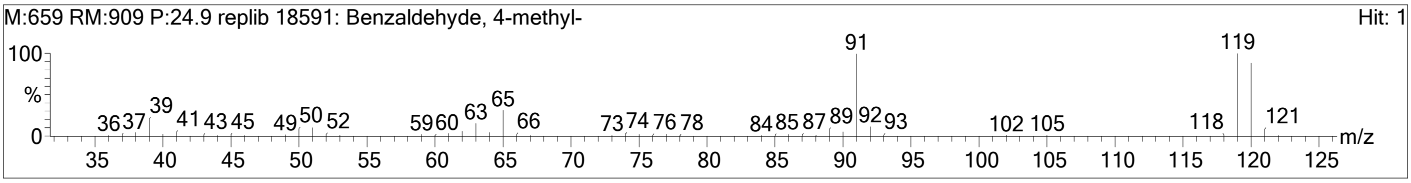

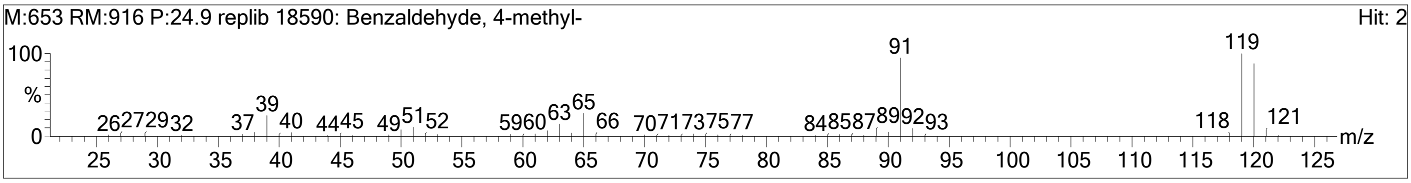

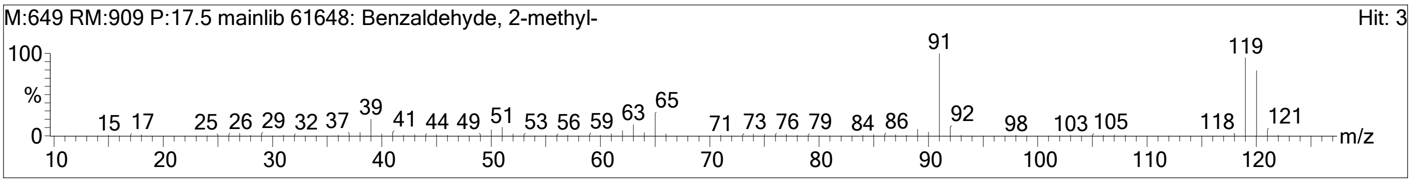

TAMILNADU AGRICULTURAL UNIVERSITY - AGRICULTURAL MICROBIOLOGY

INSTRUMENT: PERKIN ELMER CLARUS SQ8C COLOUMN: DB-5 MS CAPILARY STANDARD NON - POLARCOLOUMN
INJECTION VOL: 1 MICRO LITER DIMENSION: 30Mts, ID: 0.25 mm, FILM: 0.25 IM CARRIER GAS: He
SAMPLE ID : TI60

#	RT	Scan	Height	Area	Area %	Norm %
2	7.920	984	16,136,184	405,135.5	0.650	9.41

Pk #	RT	Hit	Compound Name	Match	R.Match	Prob.	CAS	Library
2	7.920	1	Benzene, 1,3-bis(1,1-dimethylethyl)-	768	863	53.4	1014-60-4	mainlib
		2	Benzene, 1,3-bis(1,1-dimethylethyl)-	764	854	53.4	1014-60-4	replib
		3	Benzene, 1,3-bis(1,1-dimethylethyl)-	764	849	53.4	1014-60-4	replib
		4	m-Cymene, 5-tert-butyl-	723	793	11.4	29577-19-3	mainlib
		5	Benzene, 1,4-bis(1,1-dimethylethyl)-	717	797	8.9	1012-72-2	replib
		6	Benzene, 1,4-bis(1,1-dimethylethyl)-	705	783	8.9	1012-72-2	mainlib
		7	Benzene, 1,5-dimethyl-2,4-bis(1-methylethyl)-	703	784	5.6	5186-68-5	mainlib
		8	Benzene, 1,4-dimethyl-2,5-bis(1-methylethyl)-	696	766	4.3	10375-96-9	replib
		9	Benzene, 1,4-dimethyl-2,5-bis(1-methylethyl)-	691	755	4.3	10375-96-9	replib
		10	2,3,3,4,7-Pentamethyl-2,3-dihydro-benzofuran	678	734	2.2		mainlib

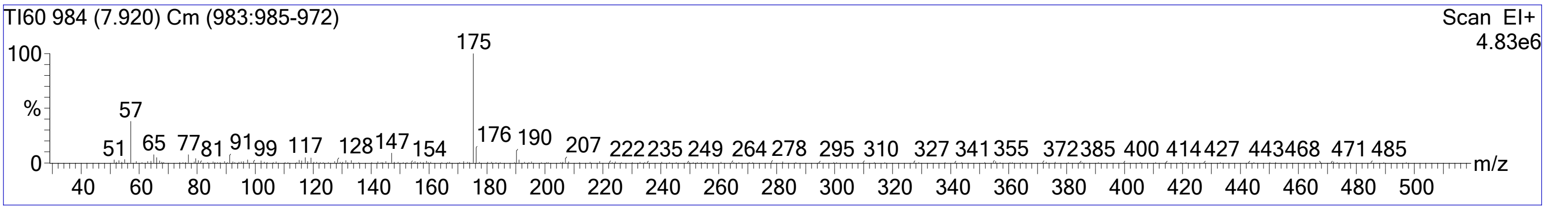

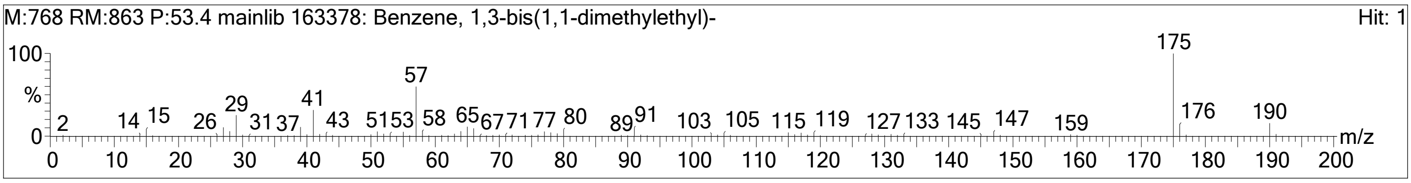

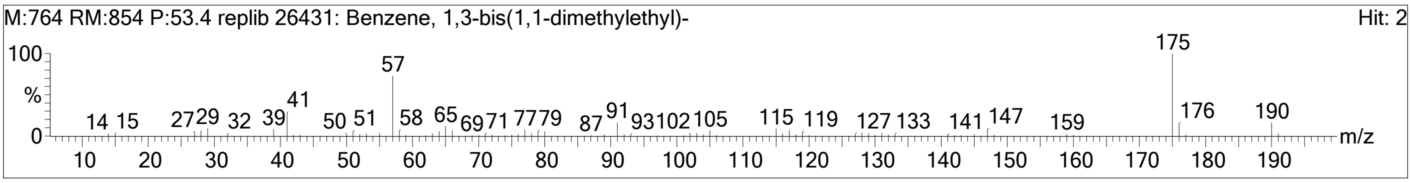

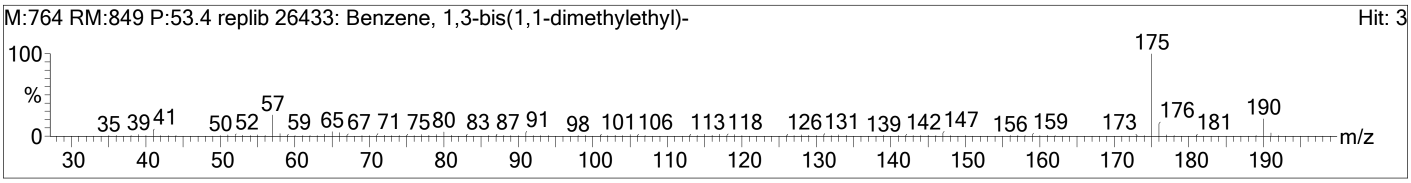

TAMILNADU AGRICULTURAL UNIVERSITY - AGRICULTURAL MICROBIOLOGY

INSTRUMENT: PERKIN ELMER CLARUS SQ8C
COLOUMN: DB-5 MS CAPILARY STANDARD NON - POLARCOLOUMN
INJECTION VOL: 1 MICRO LITER
DIMENSION: 30Mts, ID: 0.25 mm, FILM: 0.25 IM
CARRIER GAS: He
SAMPLE ID : TI60

#	RT	Scan	Height	Area	Area %	Norm %
3	11.247	1649	25,539,454	1,300,760.0	2.087	30.22

Pk #	RT	Hit	Compound Name	Match	R.Match	Prob.	CAS	Library
3	11.247	1	1-Dodecanol	782	881	8.5	112-53-8	replib
		2	1-Dodecanol	775	863	8.5	112-53-8	replib
		3	1-Dodecanol	771	855	8.5	112-53-8	replib
		4	1-Dodecanol	764	851	8.5	112-53-8	replib
		5	1-Dodecanol	763	843	8.5	112-53-8	replib
		6	1-Undecanol	762	879	3.9	112-42-5	mainlib
		7	Cyclopropane, nonyl-	758	844	3.3	74663-85-7	mainlib
		8	1-Tetradecanol	754	812	2.8	112-72-1	replib
		9	1-Dodecanol	753	832	8.5	112-53-8	replib
		10	1-Decanol	751	855	2.4	112-30-1	replib

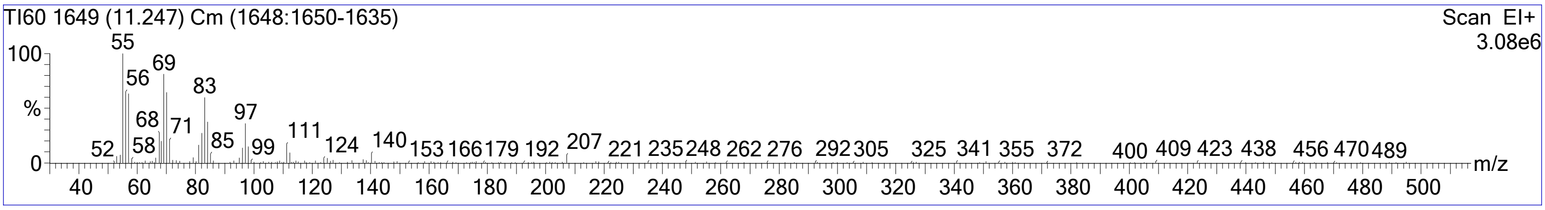

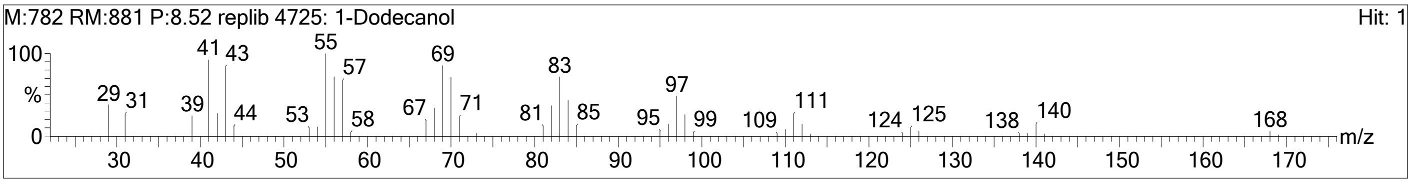

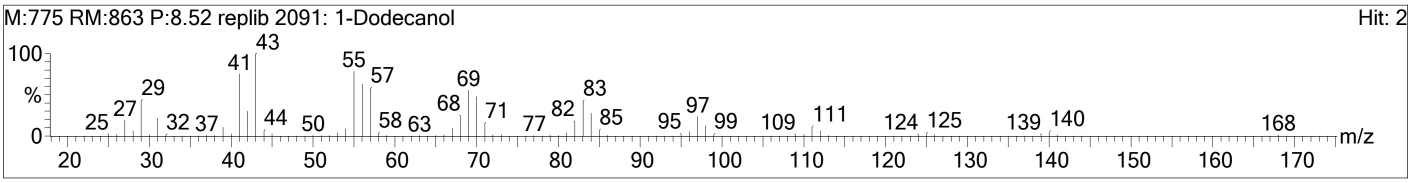

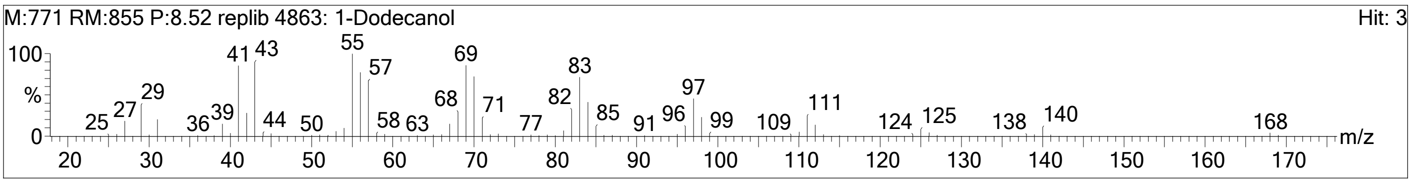

TAMILNADU AGRICULTURAL UNIVERSITY - AGRICULTURAL MICROBIOLOGY

INSTRUMENT: PERKIN ELMER CLARUS SQ8C COLOUMN: DB-5 MS CAPILARY STANDARD NON - POLARCOLOUMN
INJECTION VOL: 1 MICRO LITER DIMENSION: 30Mts, ID: 0.25 mm, FILM: 0.25 IM CARRIER GAS: He
SAMPLE ID : TI60

#	RT	Scan	Height	Area	Area %	Norm %
4	15.828	2565	108,177,888	4,303,653.0	6.905	100.00

Pk #	RT	Hit	Compound Name	Match	R.Match	Prob.	CAS	Library
4	15.828	1	Dodecyl acrylate	906	930	67.3	2156-97-0	replib
		2	Dodecyl acrylate	900	917	67.3	2156-97-0	replib
		3	Dodecyl acrylate	843	858	67.3	2156-97-0	mainlib
		4	2-Propenoic acid, pentadecyl ester	801	818	4.6	43080-23-5	mainlib
		5	2-Propenoic acid, tridecyl ester	798	821	4.0	3076-04-8	mainlib
		6	1-Dodecanol	796	843	3.7	112-53-8	replib
		7	2-Propenoic acid, tetradecyl ester	773	785	1.4	21643-42-5	mainlib
		8	Z-10-Tetradecen-1-ol acetate	762	769	0.9		mainlib
		9	Dichloroacetic acid, dodecyl ester	760	778	0.9	83005-01-0	mainlib
		10	3-(Prop-2-enoyloxy)tetradecane	757	789	0.8		mainlib

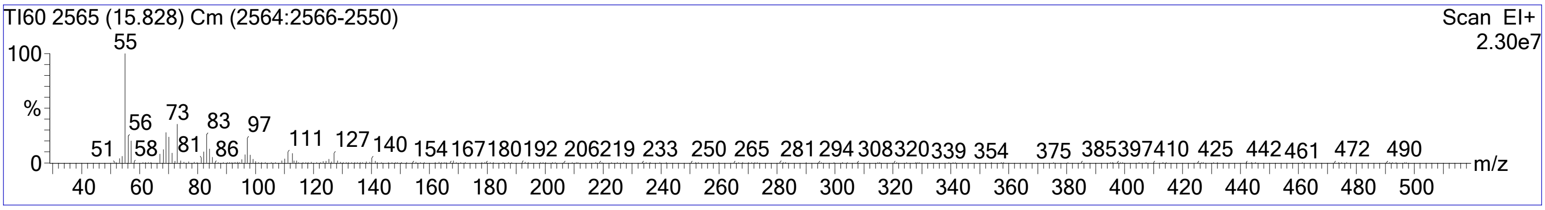

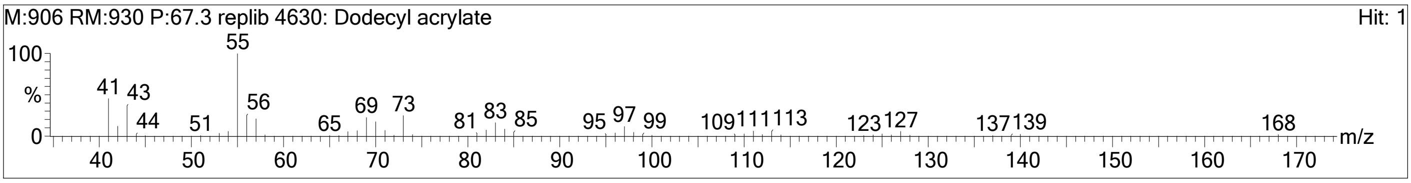

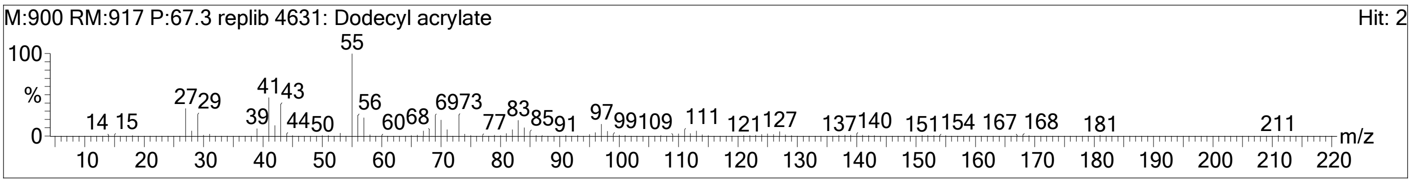

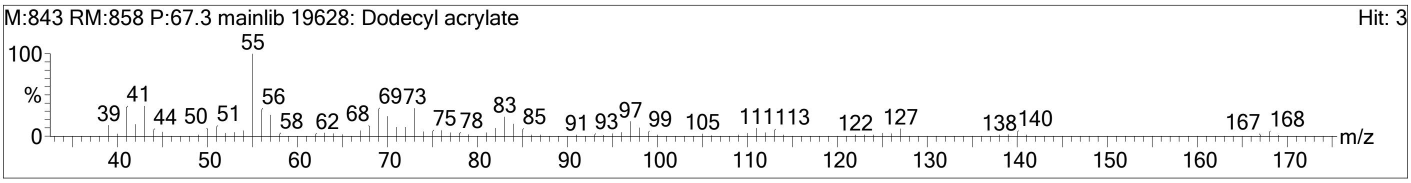

TAMILNADU AGRICULTURAL UNIVERSITY - AGRICULTURAL MICROBIOLOGY

INSTRUMENT: PERKIN ELMER CLARUS SQ8C COLOUMN: DB-5 MS CAPILARY STANDARD NON - POLARCOLOUMN
INJECTION VOL: 1 MICRO LITER DIMENSION: 30Mts, ID: 0.25 mm, FILM: 0.25 IM CARRIER GAS: He
SAMPLE ID : TI60

#	RT	Scan	Height	Area	Area %	Norm %
5	15.978	2595	7,190,967	461,969.0	0.741	10.73

Pk #	RT	Hit	Compound Name	Match	R.Match	Prob.	CAS	Library
5	15.978	1	E-10-Dodecen-1-ol propionate	502	652	6.2		mainlib
		2	1-Hexadecanol, 2-methyl-	493	568	4.5	2490-48-4	mainlib
		3	1,2-Octadecanediol	483	678	3.2	20294-76-2	mainlib
		4	1-Nonadecene	478	732	2.5	18435-45-5	replib
		5	Cyclododecane	475	623	2.3	294-62-2	replib
		6	1-Dodecanol, 3,7,11-trimethyl-	475	606	2.3	6750-34-1	replib
		7	Hexadecen-1-ol, trans-9-	472	728	2.0	64437-47-4	mainlib
		8	Propanoic acid, decyl ester	471	704	1.9	5454-19-3	mainlib
		9	Butyl hexadecyl ether	470	675	1.8		mainlib
		10	Dichloroacetic acid, 2-tetradecyl ester	469	695	1.8		mainlib

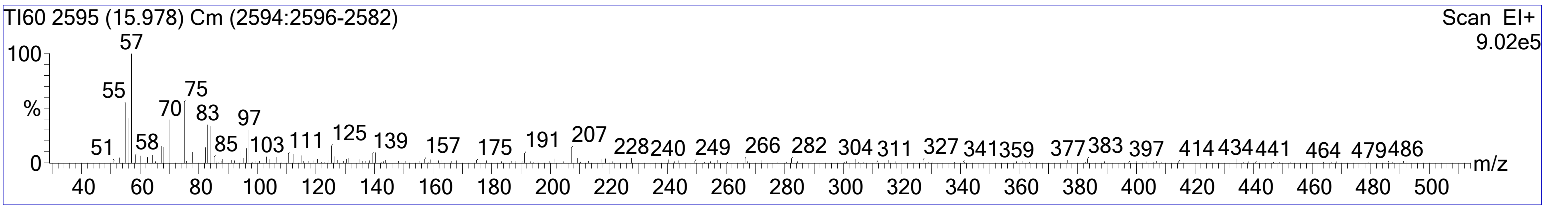

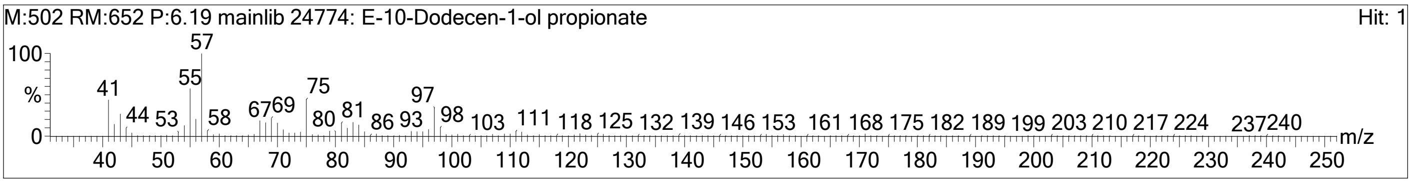

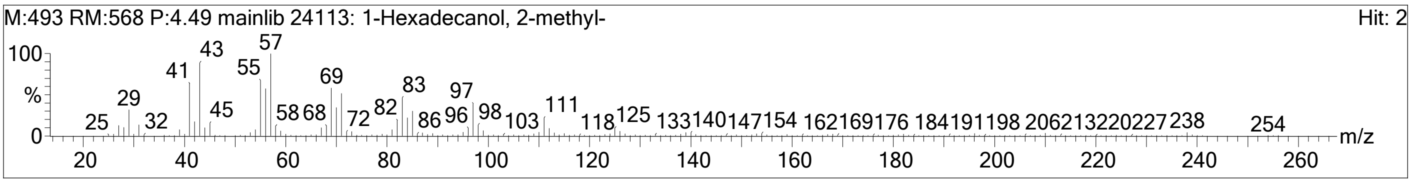

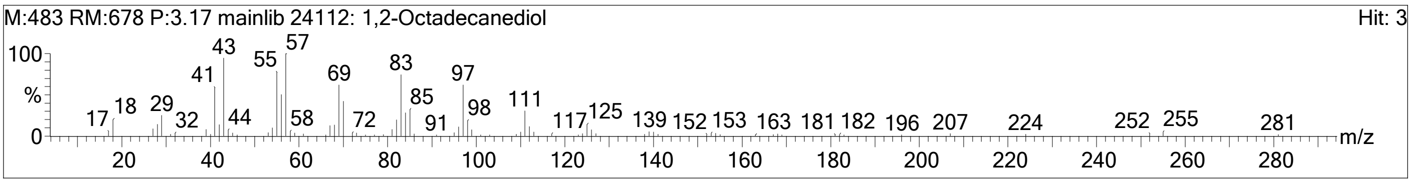

TAMILNADU AGRICULTURAL UNIVERSITY - AGRICULTURAL MICROBIOLOGY

INSTRUMENT: PERKIN ELMER CLARUS SQ8C COLOUMN: DB-5 MS CAPILARY STANDARD NON - POLARCOLOUMN
INJECTION VOL: 1 MICRO LITER DIMENSION: 30Mts, ID: 0.25 mm, FILM: 0.25 IM CARRIER GAS: He
SAMPLE ID : TI60

#	RT	Scan	Height	Area	Area %	Norm %
6	20.570	3513	6,694,133	323,641.2	0.519	7.52

Pk #	RT	Hit	Compound Name	Match	R.Match	Prob.	CAS	Library
6	20.570	1	Pentadecanoic acid, 14-methyl-, methyl ester	465	687	7.1	5129-60-2	mainlib
		2	Nonadecanoic acid, methyl ester	457	548	5.3	1731-94-8	replib
		3	Undecanoic acid, 11-bromo-, methyl ester	454	619	4.7	6287-90-7	replib
		4	Tridecanoic acid, methyl ester	450	669	4.0	1731-88-0	replib
		5	9-Bromononanoic acid, methyl(ester)	449	627	3.8	67878-15-3	mainlib
		6	Dodecanoic acid, 10-methyl-, methyl ester	446	681	3.4	5129-65-7	mainlib
		7	Hexadecanoic acid, methyl ester	446	653	3.4	112-39-0	replib
		8	Tridecanoic acid, methyl ester	445	825	4.0	1731-88-0	replib
		9	Tridecanoic acid, methyl ester	445	666	4.0	1731-88-0	mainlib
		10	Hexadecanoic acid, 15-methyl-, methyl ester	444	667	3.1	6929-04-0	mainlib

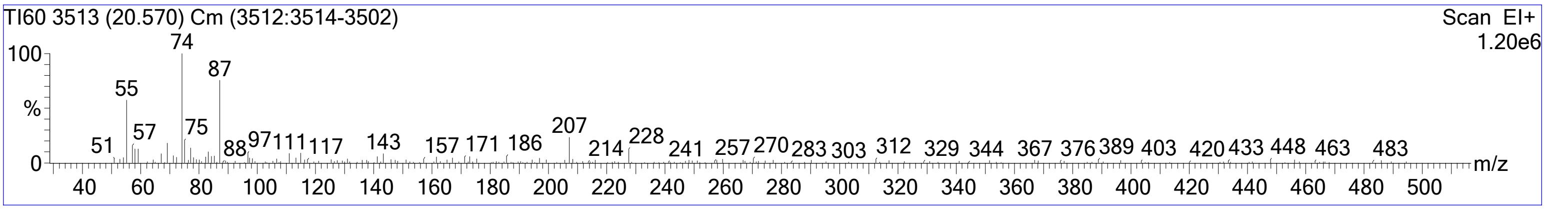

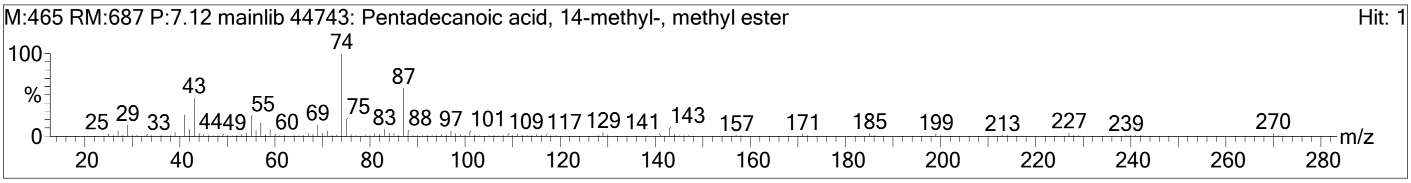

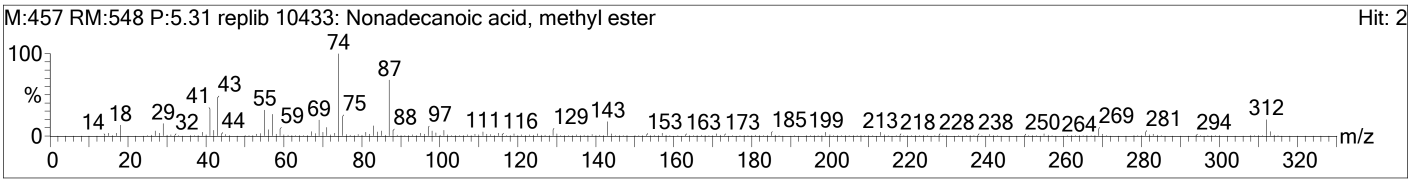

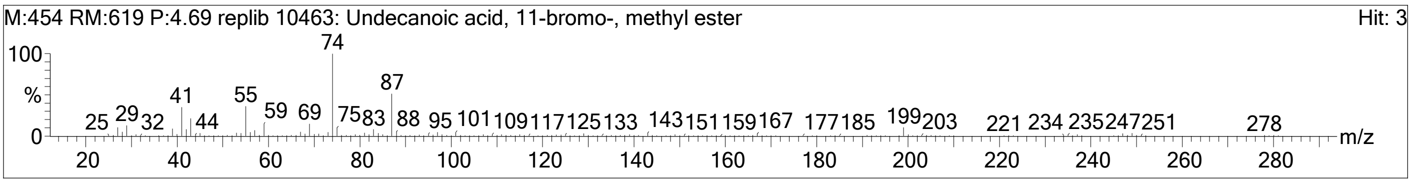

TAMILNADU AGRICULTURAL UNIVERSITY - AGRICULTURAL MICROBIOLOGY

INSTRUMENT: PERKIN ELMER CLARUS SQ8C COLOUMN: DB-5 MS CAPILARY STANDARD NON - POLARCOLOUMN
INJECTION VOL: 1 MICRO LITER DIMENSION: 30Mts, ID: 0.25 mm, FILM: 0.25 IM CARRIER GAS: He
SAMPLE ID : TI60

#	RT	Scan	Height	Area	Area %	Norm %
7	24.352	4269	9,463,171	437,936.9	0.703	10.18

Pk #	RT	Hit	Compound Name	Match	R.Match	Prob.	CAS	Library
7	24.352	1	Methyl tetradecanoate	560	721	9.2	124-10-7	replib
		2	Methyl tetradecanoate	549	742	9.2	124-10-7	replib
		3	Hexadecanoic acid, 14-methyl-, methyl ester	549	607	6.3	2490-49-5	mainlib
		4	Undecanoic acid, 11-bromo-, methyl ester	543	664	5.0	6287-90-7	replib
		5	Hexadecanoic acid, 15-methyl-, methyl ester	542	606	4.8	6929-04-0	replib
		6	Heptadecanoic acid, 16-methyl-, methyl ester	538	637	4.0	5129-61-3	mainlib
		7	Tetradecanoic acid, 12-methyl-, methyl ester	535	640	3.6	5129-66-8	mainlib
		8	Tridecanoic acid, 12-methyl-, methyl ester	531	759	3.0	5129-58-8	replib
		9	Dodecanoic acid, 2-methyl-	531	728	3.0	2874-74-0	replib
		10	Hexadecanoic acid, 15-methyl-, methyl ester	529	692	4.8	6929-04-0	mainlib

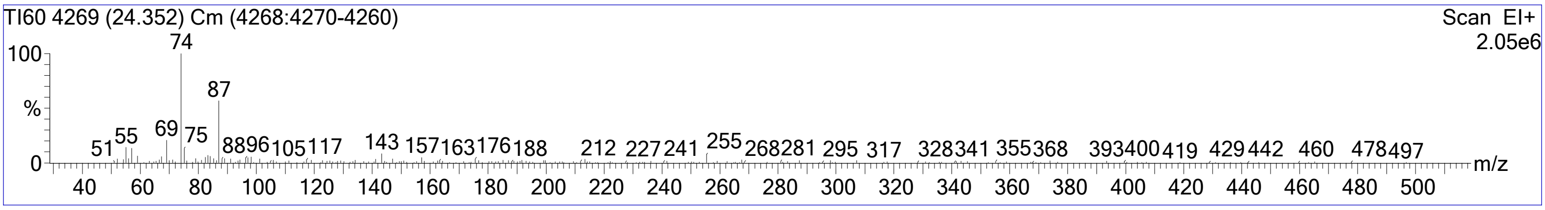

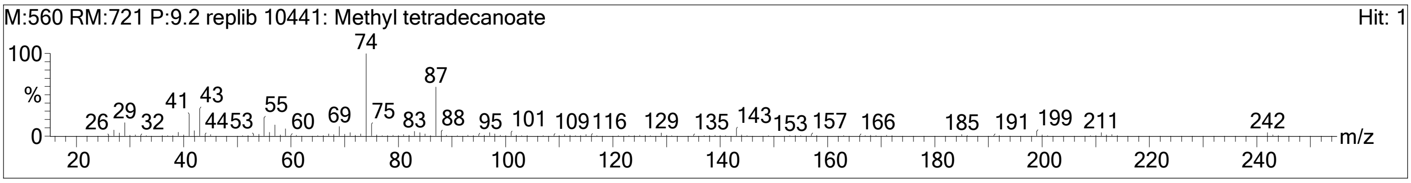

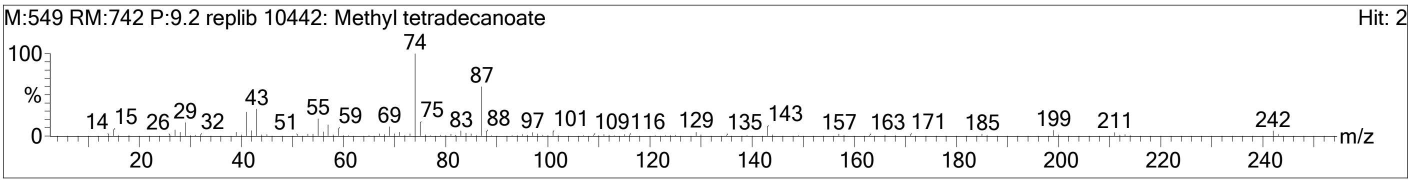

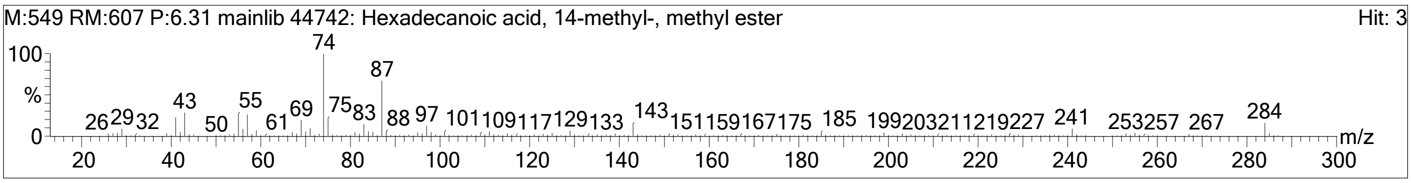

TAMILNADU AGRICULTURAL UNIVERSITY - AGRICULTURAL MICROBIOLOGY

INSTRUMENT: PERKIN ELMER CLARUS SQ8C   COLOUMN: DB-5 MS CAPILARY STANDARD NON - POLARCOLOUMN
INJECTION VOL: 1 MICRO LITER   DIMENSION: 30Mts, ID: 0.25 mm, FILM: 0.25 IM   CARRIER GAS: He
SAMPLE ID : TI60

#	RT	Scan	Height	Area	Area %	Norm %
8	30.019	5402	6,070,414	588,327.9	0.944	13.67

Pk #	RT	Hit	Compound Name	Match	R.Match	Prob.	CAS	Library
8	30.019	1	5,7,9(11)-Androstatriene, 3-hydroxy-17-oxo-	406	511	13.5		mainlib
		2	1,8-Dioxa-5-thiaoctane, 8-(9-borabicyclo[3.3.1]non-9-yl)-3-(9-borabicyclo[3.3.1]non-9-yloxy)-1-phenyl-	386	446	6.2		mainlib
		3	Acetic acid, 2-[[3-cyano-4-(methoxymethyl)-6-methyl-2-pyridinyl]thio]-	372	504	3.8		mainlib
		4	Triprolidine	372	503	3.8	486-12-4	replib
		5	N-(2,4-Dimethylphenyl)-7-nitro-2,1,3-benzoxadiazol-4-amine	369	451	3.4		mainlib
		6	Triprolidine	366	505	3.8	486-12-4	replib
		7	Benzothiazole-2,3-dicarboxylic acid, 2,3-dihydro-6-nitro-, dimethyl ester, 1,1-dioxide	366	458	3.0	335434-44-1	mainlib
		8	Thiocarbamic acid, N,N-dimethyl, S-1,3-diphenyl-2-butenyl ester	365	478	2.9		mainlib
		9	Triprolidine	364	490	3.8	486-12-4	replib
		10	Ethyl 4,4,6,6,8,8-hexamethyl-11-oxo-3,5,7,9,12-pentaoxa-4,6,8-trisilatetradecan-1-oate	363	490	2.7		mainlib

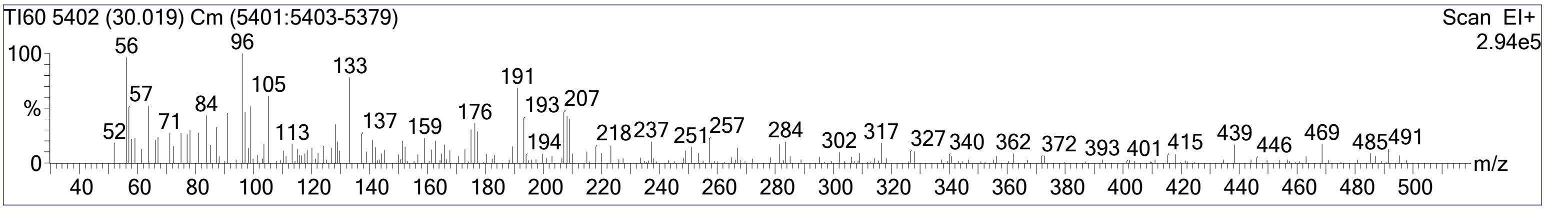

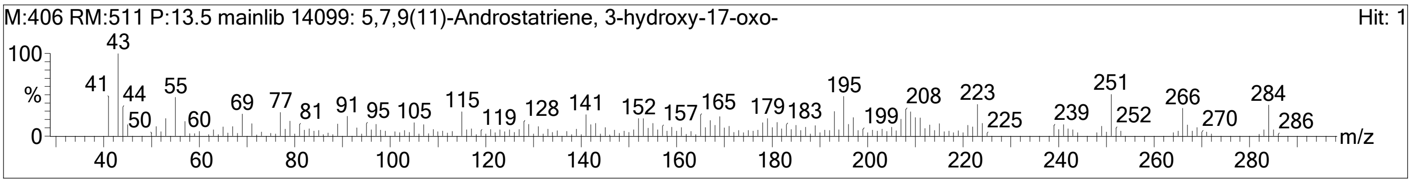

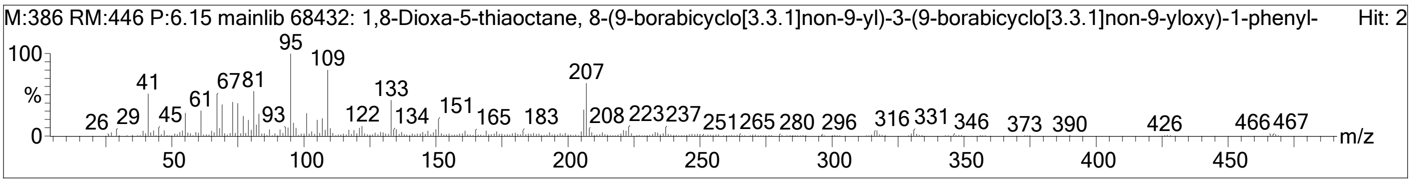

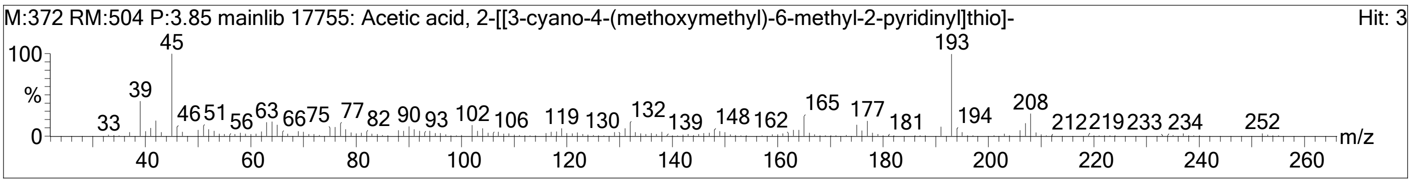

TAMILNADU AGRICULTURAL UNIVERSITY - AGRICULTURAL MICROBIOLOGY

INSTRUMENT: PERKIN ELMER CLARUS SQ8C COLOUMN: DB-5 MS CAPILARY STANDARD NON - POLARCOLOUMN
INJECTION VOL: 1 MICRO LITER DIMENSION: 30Mts, ID: 0.25 mm, FILM: 0.25 IM CARRIER GAS: He
SAMPLE ID : TI60

#	RT	Scan	Height	Area	Area %	Norm %
9	30.149	5428	8,710,438	548,205.6	0.880	12.74

Pk #	RT	Hit	Compound Name	Match	R.Match	Prob.	CAS	Library
9	30.149	1	Benzo[h]quinoline, 2,4-dimethyl-	430	654	8.2	605-67-4	mainlib
		2	1,2-Benzenediol, 3,5-bis(1,1-dimethylethyl)-	426	562	6.9	1020-31-1	replib
		3	1,2-Benzenediol, 3,5-bis(1,1-dimethylethyl)-	424	608	6.9	1020-31-1	mainlib
		4	2-Bromo-4,5-dimethoxycinnamic acid	421	584	5.6	51314-72-8	mainlib
		5	1,2-Dimethoxy-4-(1,3-dimethoxy-1-propenyl)benzene	421	568	5.6		mainlib
		6	Benzo[h]quinoline, 2,4-dimethyl-	418	734	8.2	605-67-4	replib
		7	2-Methyl-pentanoic acid [4-(2-methyl-pentanoylsulfamoyl)-phenyl]-amide	414	496	4.3		mainlib
		8	6-Amino-5-cyano-4-(5-cyano-2,4-dimethyl-1H-pyrrol-3-yl)-2-methyl-4H-pyran-3-carboxylic acid ethyl ester	411	480	3.8		mainlib
		9	5H-Benzo[b]pyran-8-ol, 2,3,5,5,8a-pentamethyl-6,7,8,8a-tetrahydro-	409	498	3.5	97306-66-6	replib
		10	4,6-di-tert-Butylresorcinol	405	568	2.9	5374-06-1	mainlib

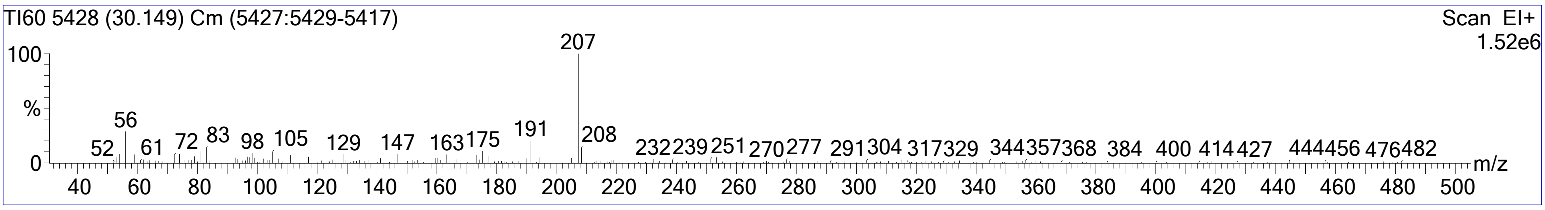

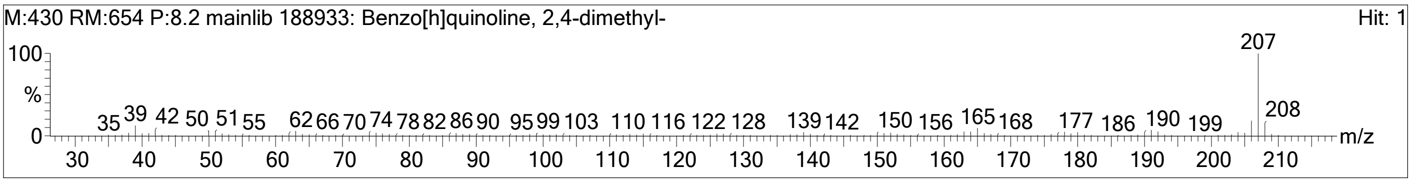

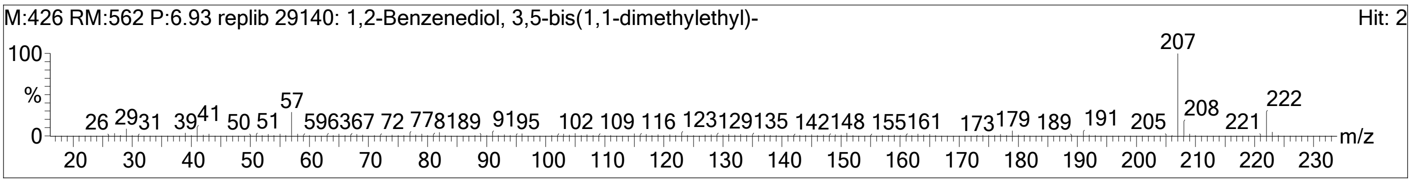

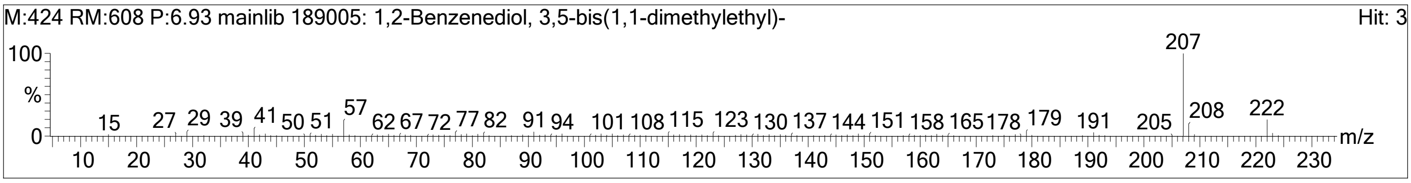

TAMILNADU AGRICULTURAL UNIVERSITY - AGRICULTURAL MICROBIOLOGY

INSTRUMENT: PERKIN ELMER CLARUS SQ8C COLOUMN: DB-5 MS CAPILARY STANDARD NON - POLARCOLOUMN
INJECTION VOL: 1 MICRO LITER DIMENSION: 30Mts, ID: 0.25 mm, FILM: 0.25 IM CARRIER GAS: He
SAMPLE ID : TI60

#	RT	Scan	Height	Area	Area %	Norm %
10	30.194	5437	10,091,696	847,115.9	1.359	19.68

Pk #	RT	Hit	Compound Name	Match	R.Match	Prob.	CAS	Library
10	30.194	1	1,4,7-Androstatrien-3,17-dione	398	477	10.4	14532-68-4	mainlib
		2	1,8-Dioxa-5-thiaoctane, 8-(9-borabicyclo[3.3.1]non-9-yl)-3-(9-borabicyclo[3.3.1]non-9-yloxy)-1-phenyl-	387	445	7.1		mainlib
		3	Acetic acid, 3-acetoxy-6-(2-cyanovinyl)-3a,6-dimethyl-2,3,3a,4,5,5a,6,9,9a,9b-decahydro-1H-cyclopenta[a]naphthalen-7-ylmethyl ester	379	430	5.3		mainlib
		4	4-[4-(2-Methoxyphenyl)-1H-pyrazol-3-yl]benzene-1,3-diol	375	446	4.5		mainlib
		5	D-Homo-24-nor-17-oxachola-20,22-dien-16-one, 1,3,7-tris(acetyloxy)-14,15:21,23-diepoxy-4,4,8-trimethyl-, (1à,3à,5à,7à,13à,14á,15á,17aà)-	374	397	4.3	2524-38-1	mainlib
		6	5àAndrost-16-ol, 17-ethylidene-3,5-dedihydro-6-methoxy-, pivalate	368	432	3.4		mainlib
		7	9-Octadecenoic acid (Z)-, tetradecyl ester	368	406	3.4	22393-85-7	mainlib
		8	Acridin-1(2H)-one, 3,4-dihydro-3,3-dimethyl-9-propylamino-	365	452	3.0	146352-02-5	mainlib
		9	1-(2-Acetoxyethyl)-3,6-diazahomoadamantan-9-one oxime	365	448	3.0		mainlib
		10	Ingol 3,8,12-triacetate	365	398	3.0		mainlib

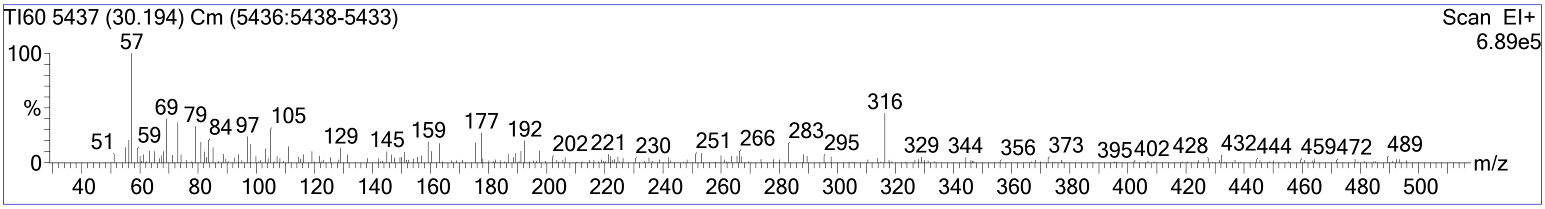

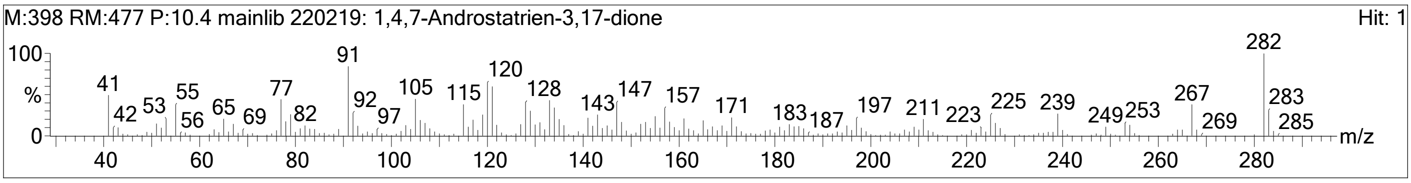

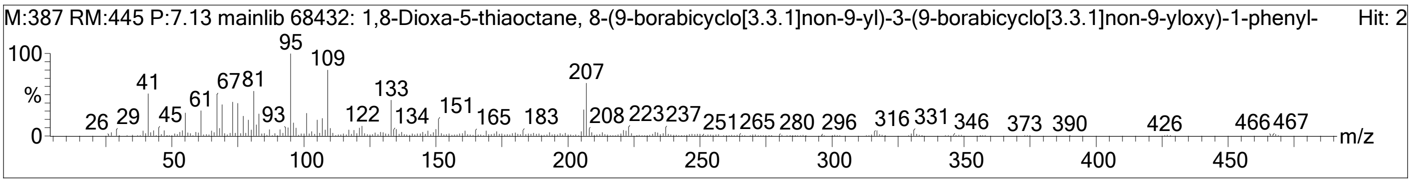

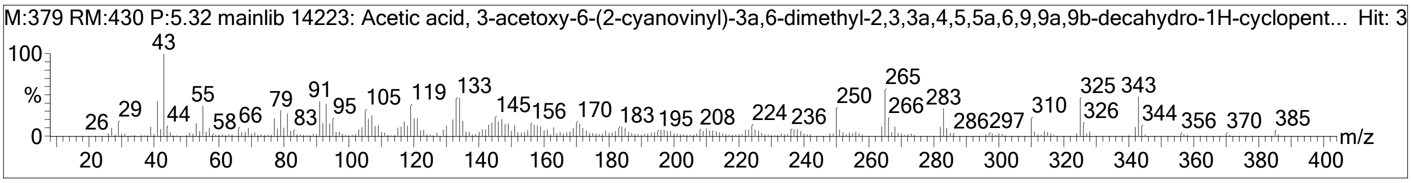

TAMILNADU AGRICULTURAL UNIVERSITY - AGRICULTURAL MICROBIOLOGY

INSTRUMENT: PERKIN ELMER CLARUS SQ8C
COLOUMN: DB-5 MS CAPILARY STANDARD NON - POLARCOLOUMN
INJECTION VOL: 1 MICRO LITER
DIMENSION: 30Mts, ID: 0.25 mm, FILM: 0.25 IM
CARRIER GAS: He
SAMPLE ID : TI60

#	RT	Scan	Height	Area	Area %	Norm %
11	30.304	5459	12,607,431	459,718.3	0.738	10.68

Pk #	RT	Hit	Compound Name	Match	R.Match	Prob.	CAS	Library
11	30.304	1	Docosanoic acid, 1,2,3-propanetriyl ester	402	425	5.8	18641-57-1	mainlib
		2	Dodecanoic acid, 1a,2,5,5a,6,9,10,10a-octahydro-5a-hydroxy-4-(hydroxymethyl)-1,1,7,9-tetramethyl-6,11-dioxo-1H-2,8a-methanocyclopenta[a]cyclopropa[e]cyclodecen-5-yl ester, [1aR-(1aà,2à,5á,5aá,8aà,9à,10aà)]-	396	396	4.5	77508-68-0	mainlib
		3	Octadecane, 3-ethyl-5-(2-ethylbutyl)-	389	422	3.5	55282-12-7	mainlib
		4	10-Acetoxy-2-hydroxy-1,2,6a,6b,9,9,12a-heptamethyl-1,3,4,5,6,6a,6b,7,8,8a,9,10,11,12,12a,12b,13,14b-octadecahydro-2H-picene-4a-carboxylic acid, methyl ester	387	405	3.2	14356-56-0	mainlib
		5	á-D-Galactopyranoside, methyl 2,3-bis-O-(trimethylsilyl)-, cyclic butylboronate	386	463	3.1	56211-10-0	mainlib
		6	Acetic acid, 17-(4-hydroxy-5-methoxy-1,5-dimethylhexyl)-4,4,10,13,14-pentamethyl-2,3,4,5,6,7,10,11,12,13,14,15,16,17-tetradecahydrocyclopenta[a]phenanthryl ester	386	404	3.1		mainlib
		7	Dihydromorphine, 2TMS derivative	385	412	3.0		mainlib
		8	3á,4á-Bis(trimethylsiloxy)cholest-5-ene	384	426	2.8	33287-25-1	mainlib
		9	Cholest-1-eno[2,1-a]naphthalene, 3',4'-dihydro-	380	403	2.4		mainlib
		10	17-Pentatriacontene	378	400	2.2	6971-40-0	mainlib

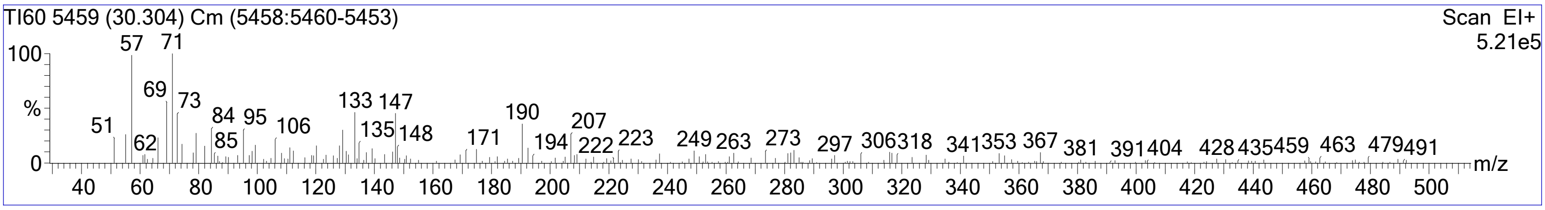

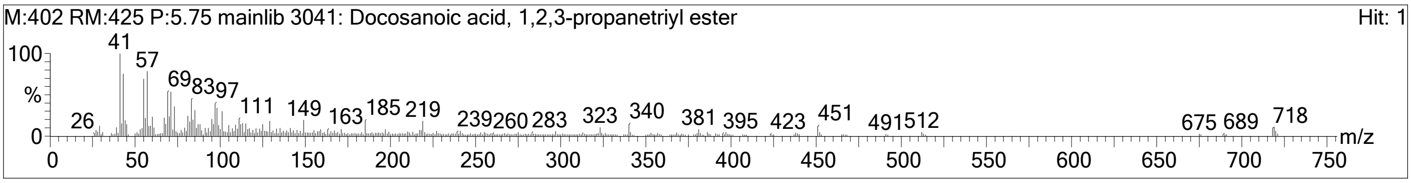

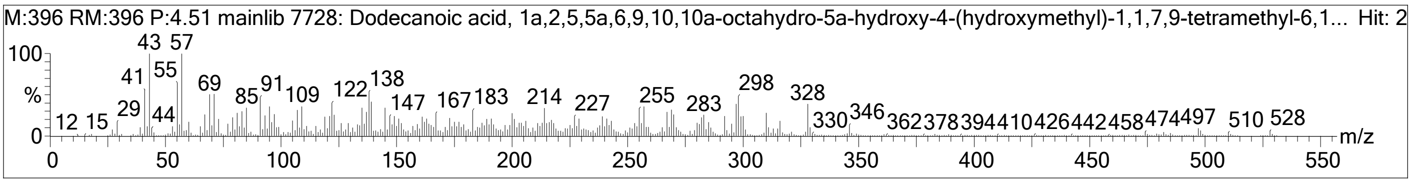

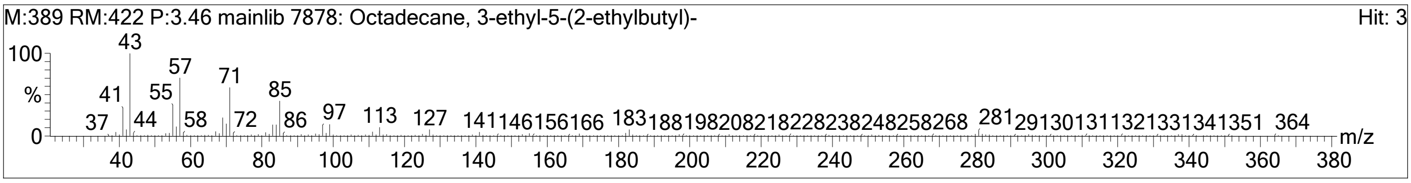

TAMILNADU AGRICULTURAL UNIVERSITY - AGRICULTURAL MICROBIOLOGY

INSTRUMENT: PERKIN ELMER CLARUS SQ8C COLOUMN: DB-5 MS CAPILARY STANDARD NON - POLARCOLOUMN
INJECTION VOL: 1 MICRO LITER DIMENSION: 30Mts, ID: 0.25 mm, FILM: 0.25 IM CARRIER GAS: He
SAMPLE ID : TI60

#	RT	Scan	Height	Area	Area %	Norm %
12	30.364	5471	10,596,189	368,394.3	0.591	8.56

Pk #	RT	Hit	Compound Name	Match	R.Match	Prob.	CAS	Library
12	30.364	1	.psi.,.psi.-Carotene, 1,1',2,2'-tetrahydro-1,1'-dimethoxy-	422	422	30.0	13833-01-7	mainlib
		2	1,8-Dioxa-5-thiaoctane, 8-(9-borabicyclo[3.3.1]non-9-yl)-3-(9-borabicyclo[3.3.1]non-9-yloxy)-1-phenyl-	385	445	7.3		mainlib
		3	D-Homo-24-nor-17-oxachola-20,22-diene-3,16-dione, 14,15:21,23-diepoxy-7-hydroxy-4,4,8-trimethyl-, (5à,7à,13à,14á,15á,17aà)-	385	406	7.3	10314-91-7	mainlib
		4	d-Homo-24-nor-17-oxachola-20,22-diene-3,16-dione, 7-(acetyloxy)-14,15:21,23-diepoxy-4,4,8-trimethyl-, (5à,7à,13à,14á,15á,17aà)-	374	388	5.0	2629-11-0	mainlib
		5	.psi.,.psi.-Carotene, 3,4-didehydro-1,2,7',8'-tetrahydro-1-methoxy-2-oxo-	373	376	4.8	13836-70-9	mainlib
		6	2-[(3H-Benzoimidazol-5-ylimino)-methyl]-4-nitro-phenol	358	464	2.9		mainlib
		7	3-Heptafluorobutyriloxy-3,5,10-pregnatrien-20-one	355	414	2.6		mainlib
		8	Butane-1,4-dioic acid, 2-[S-[3,6-dihydroxy-2,4,5-trimethylphenyl]thio]-	354	434	2.5		mainlib
		9	11-Dimethyl-1,2,3,4-tetrahydro-1,4-methanophenazine-1-carboxylic acid, methyl ester	353	428	2.4		mainlib
		10	Thiocoumarin-7-ol-2-aceic acid, 5,6,8-trimethyl-	348	423	1.9		mainlib

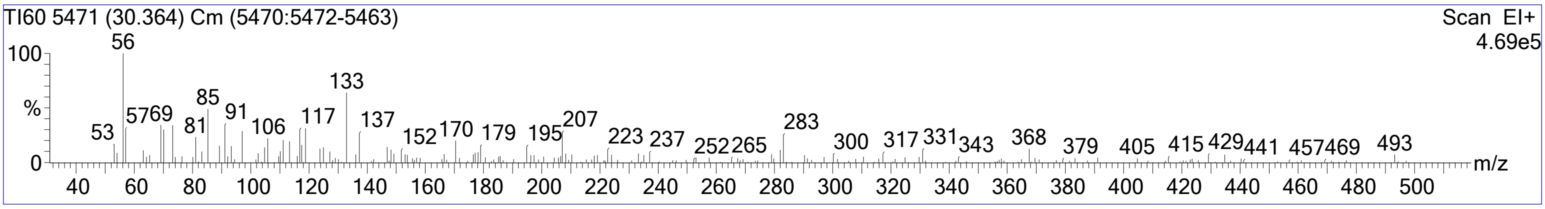

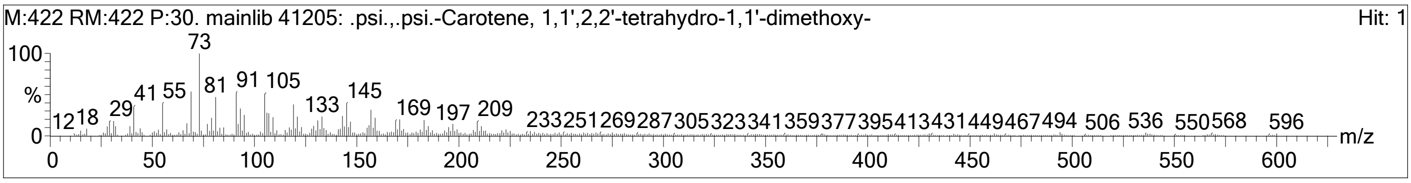

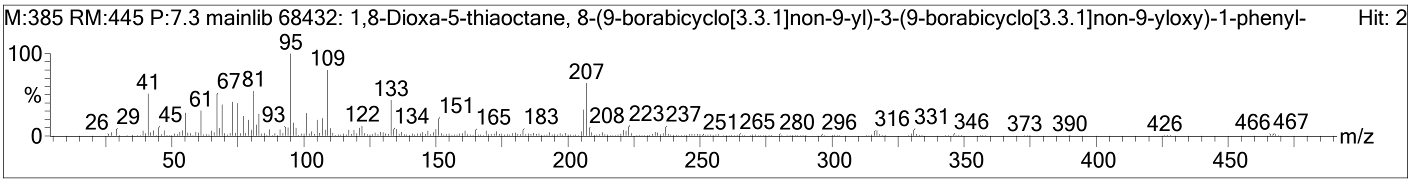

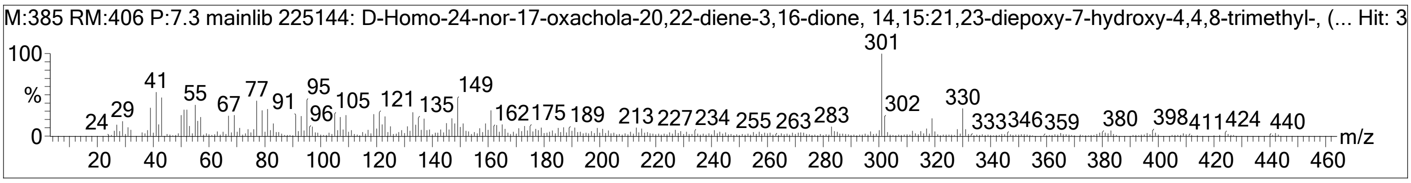

TAMILNADU AGRICULTURAL UNIVERSITY - AGRICULTURAL MICROBIOLOGY

INSTRUMENT: PERKIN ELMER CLARUS SQ8C COLOUMN: DB-5 MS CAPILARY STANDARD NON - POLARCOLOUMN
INJECTION VOL: 1 MICRO LITER DIMENSION: 30Mts, ID: 0.25 mm, FILM: 0.25 IM CARRIER GAS: He
SAMPLE ID : TI60

#	RT	Scan	Height	Area	Area %	Norm %
13	30.434	5485	13,337,660	868,989.6	1.394	20.19

Pk #	RT	Hit	Compound Name	Match	R.Match	Prob.	CAS	Library
13	30.434	1	Estriol 16à-(á-D-glucuronide)	697	999	55.1	1852-50-2	nist_msms
		2	Estriol 16à-(á-D-glucuronide)	697	999	55.1	1852-50-2	nist_msms
		3	Estriol 16à-(á-D-glucuronide)	697	999	55.1	1852-50-2	nist_msms
		4	Estriol 16à-(á-D-glucuronide)	697	999	55.1	1852-50-2	nist_msms
		5	Estriol 16à-(á-D-glucuronide)	697	999	55.1	1852-50-2	nist_msms
		6	Hyperoside	689	988	41.1	482-36-0	nist_msms
		7	4-Androsten-17á-ol-3-one glucosiduronate	592	786	3.2	1180-25-2	nist_msms
		8	Hyperoside	563	807	41.1	482-36-0	nist_msms
		9	4-Androsten-17á-ol-3-one glucosiduronate	561	805	3.2	1180-25-2	nist_msms
		10	4-Androsten-17á-ol-3-one glucosiduronate	559	802	3.2	1180-25-2	nist_msms

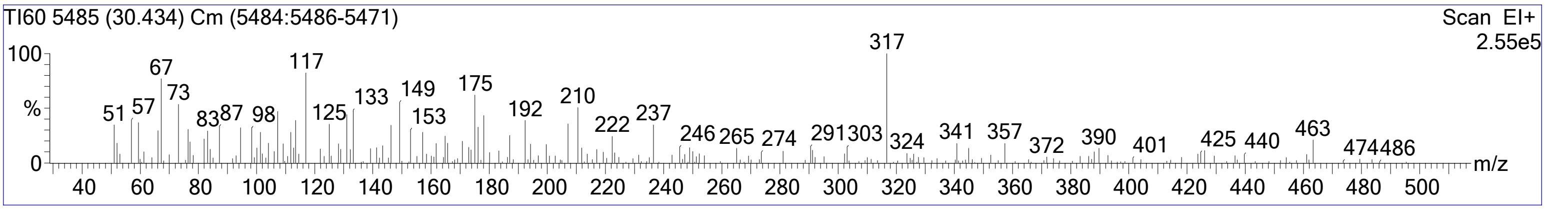

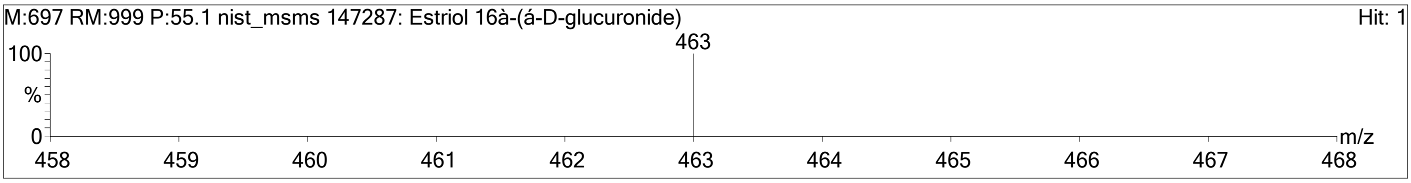

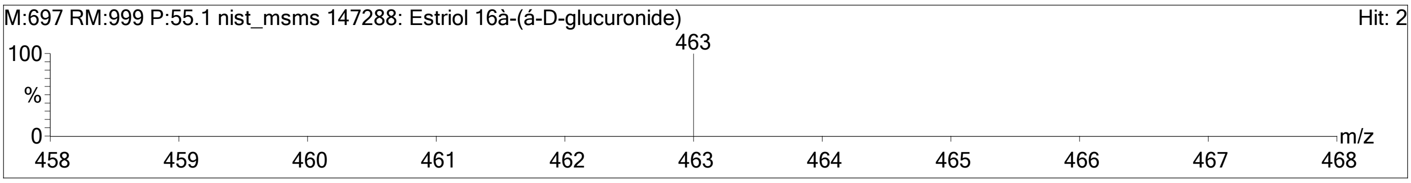

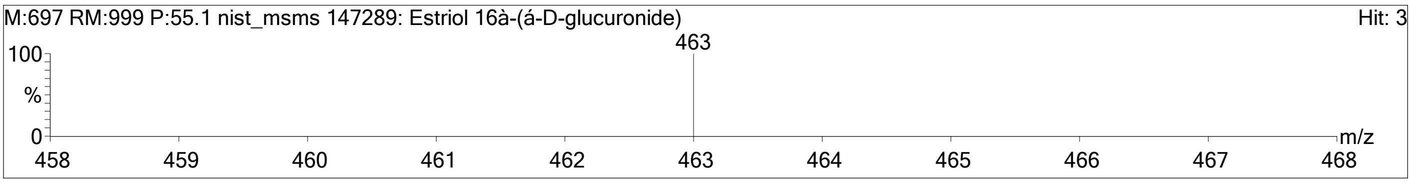

TAMILNADU AGRICULTURAL UNIVERSITY - AGRICULTURAL MICROBIOLOGY

INSTRUMENT: PERKIN ELMER CLARUS SQ8C COLOUMN: DB-5 MS CAPILARY STANDARD NON - POLARCOLOUMN
INJECTION VOL: 1 MICRO LITER DIMENSION: 30Mts, ID: 0.25 mm, FILM: 0.25 IM CARRIER GAS: He
SAMPLE ID : TI60

#	RT	Scan	Height	Area	Area %	Norm %
14	30.554	5509	15,085,814	1,223,963.0	1.964	28.44

Pk #	RT	Hit	Compound Name	Match	R.Match	Prob.	CAS	Library
14	30.554	1	7aH-Cyclopenta[a]cyclopropa[f]cycloundecene-2,4,7,7a,10,11-hexol, 1,1a,2,3,4,4a,5,6,7,10,11,11a-dodecahydro-1,1,3,6,9-pentamethyl-, 2,4,7,10,11-pentaacetate	422	436	11.3	51906-08-2	mainlib
		2	1-(2-Acetoxyethyl)-3,6-diazahomoadamantan-9-one oxime	415	493	8.6		mainlib
		3	Propanedioic acid, mononitrile, 2-[tetrahydro-4-(4-fluorophenyl)-2,2-dimethyl-4-pyranyl]-, ethyl ester	404	489	5.9	120729-52-4	mainlib
		4	Endrin ketone	404	452	5.9	53494-70-5	replib
		5	Trinexapac-ethyl, TMS derivative	398	443	4.7		mainlib
		6	Phen-1,4-diol, 2,3-dimethyl-5-trifluoromethyl-	397	496	4.5		mainlib
		7	Carnegine	387	461	3.2	490-53-9	mainlib
		8	Acetic acid, 17-acetoxy-4,4,10,13-tetramethyl-7-oxo-2,3,4,7,8,9,10,11,12,13,14,15,16,17-tetradecahydro-1H-cyclopenta[a]phenanthren-3-yl (ester)	386	403	3.0		mainlib
		9	Furan, 2,5-bis(3,4-dimethoxyphenyl)tetrahydro-3,4-dimethyl-, [2R-(2à,3á,4á,5à)]-	383	461	2.7	528-63-2	replib
		10	(3-Benzenesulfonyl-4-isobutyrylcyclopent-2-enyl)acetic acid, methyl ester	373	453	1.9		mainlib

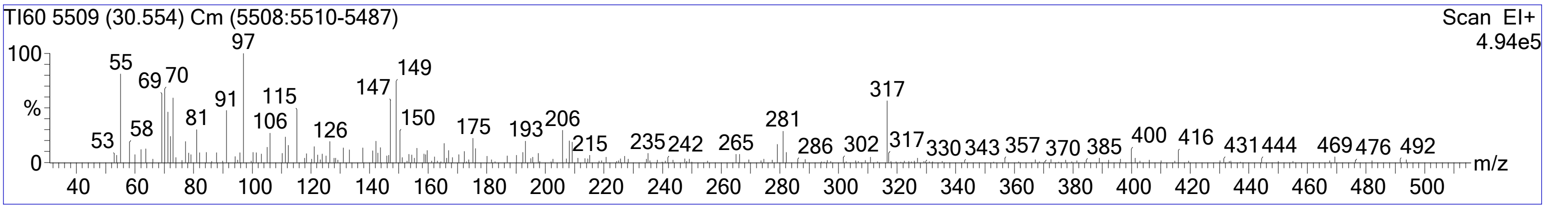

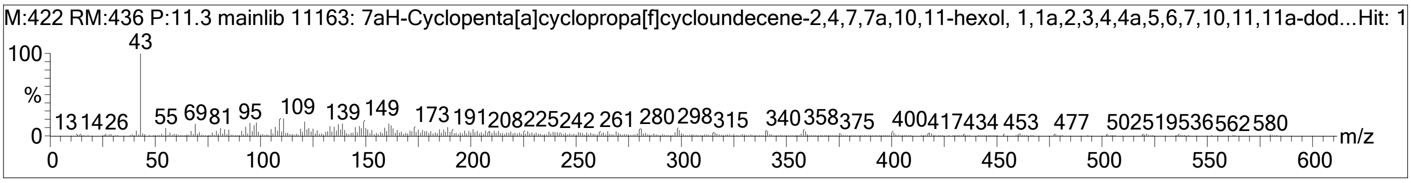

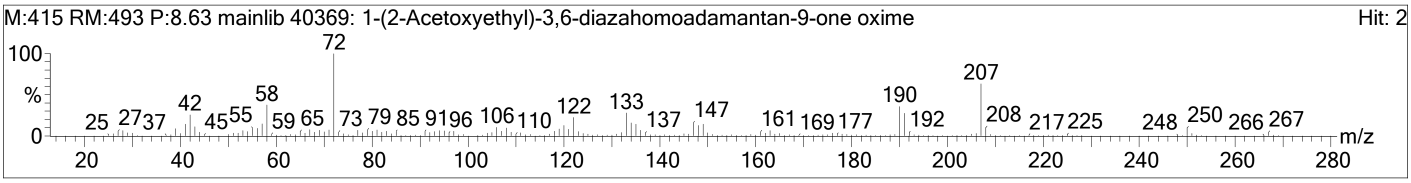

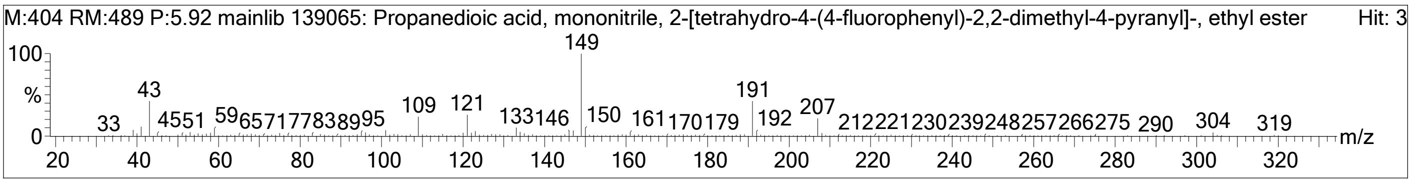

TAMILNADU AGRICULTURAL UNIVERSITY - AGRICULTURAL MICROBIOLOGY

INSTRUMENT: PERKIN ELMER CLARUS SQ8C COLOUMN: DB-5 MS CAPILARY STANDARD NON - POLARCOLOUMN
INJECTION VOL: 1 MICRO LITER DIMENSION: 30Mts, ID: 0.25 mm, FILM: 0.25 IM CARRIER GAS: He
SAMPLE ID : TI60

#	RT	Scan	Height	Area	Area %	Norm %
15	30.579	5514	11,865,328	581,444.2	0.933	13.51

Pk #	RT	Hit	Compound Name	Match	R.Match	Prob.	CAS	Library
15	30.579	1	7,8-Epoxylanostan-11-ol, 3-acetoxy-	411	423	4.8		mainlib
		2	Clocortolone pivalate	409	445	4.4	34097-16-0	mainlib
		3	17-Pentatriacontene	400	461	3.2	6971-40-0	replib
		4	Dodecanoic acid, 1a,2,5,5a,6,9,10,10a-octahydro-5a-hydroxy-4-(hydroxymethyl)-1,1,7,9-tetramethyl-6,11-dioxo-1H-2,8a-methanocyclopenta[a]cyclopropa[e]cyclodecen-5-yl ester, [1aR-(1aà,2à,5á,5aá,8aà,9à,10aà)]-	399	399	3.1	77508-68-0	mainlib
		5	Stearic acid, 2-(9-octadecenyloxy)ethyl ester, (Z)-	396	490	2.7	29027-97-2	mainlib
		6	Cyclohexane, 1,3,5-trimethyl-2-octadecyl-	394	413	2.5	55282-34-3	mainlib
		7	Heptadecane, 9-hexyl-	392	431	2.3	55124-79-3	mainlib
		8	2-Nonadecanone 2,4-dinitrophenylhydrazine	391	438	2.2	28813-61-8	mainlib
		9	17-Pentatriacontene	390	421	3.2	6971-40-0	mainlib
		10	Tetradecanoic acid, 9a-(acetyloxy)-1a,1b,4,4a,5,7a,7b,8,9,9a-decahydro-7b-hydroxy-3-(hydroxymethyl)-4a-methoxy-1,1,6,8-tetramethyl-5-oxo-1H-cyclopropa[3,4]benz[1,2-e]azulen-9-yl ester, [1aR-(1aà,1bá,4aá,7aà,7bà,8à,9á,9aà)]-	390	417	2.2	57716-89-9	mainlib

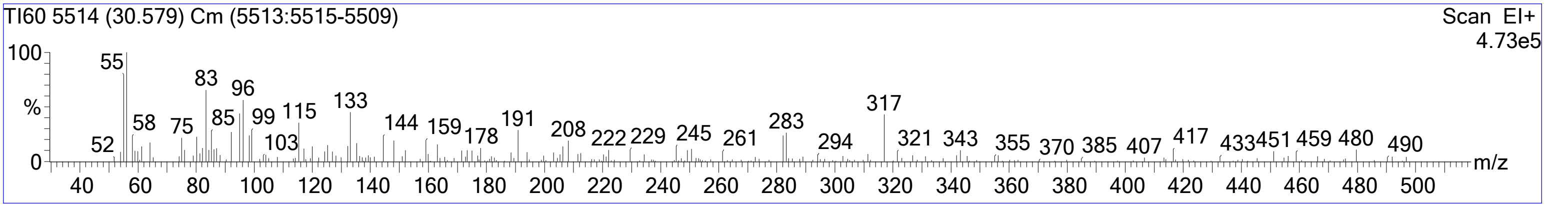

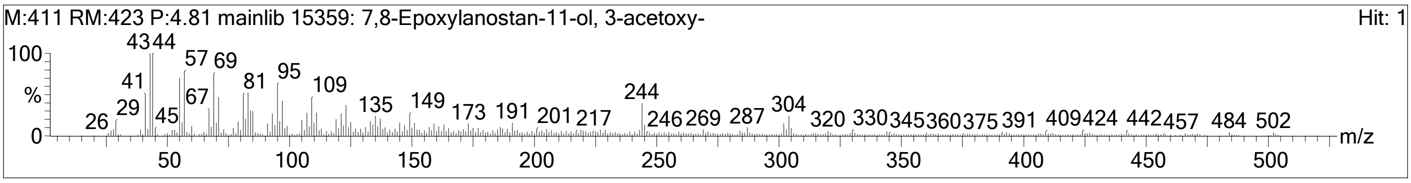

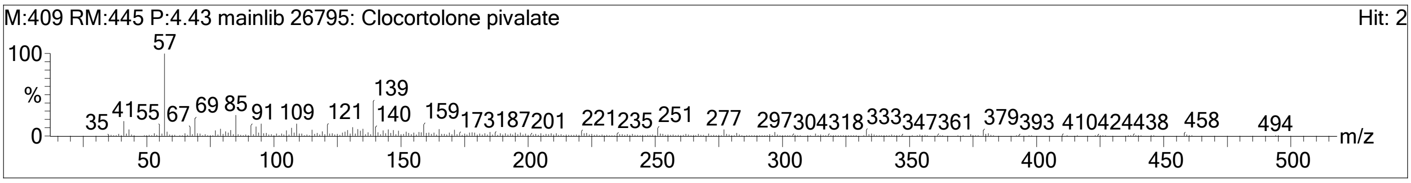

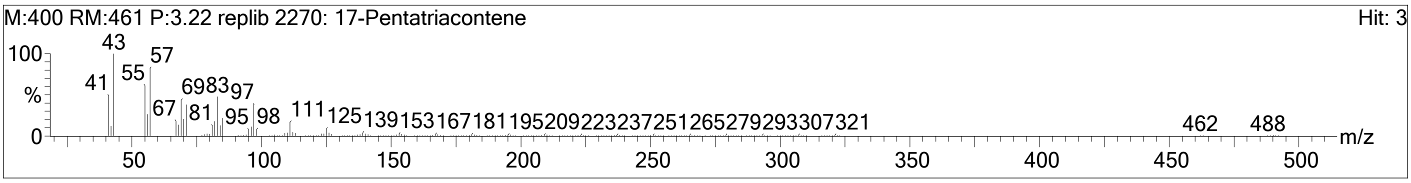

TAMILNADU AGRICULTURAL UNIVERSITY - AGRICULTURAL MICROBIOLOGY

INSTRUMENT: PERKIN ELMER CLARUS SQ8C COLOUMN: DB-5 MS CAPILARY STANDARD NON - POLARCOLOUMN
INJECTION VOL: 1 MICRO LITER DIMENSION: 30Mts, ID: 0.25 mm, FILM: 0.25 IM CARRIER GAS: He
SAMPLE ID : TI60

#	RT	Scan	Height	Area	Area %	Norm %
16	30.634	5525	11,048,982	417,876.8	0.670	9.71

Pk #	RT	Hit	Compound Name	Match	R.Match	Prob.	CAS	Library
16	30.634	1	Clonazepam	517	950	61.3	1622-61-3	nist_msms
		2	9-Borabicyclo[3.3.1]nonane, 9,9'-[1,4-phenylenebis(carbonothioylthio)]bis-	380	452	2.2	139759-17-4	mainlib
		3	Clonazepam	377	807	61.3	1622-61-3	nist_msms
		4	Pregnan-18-oic acid, 20-hydroxy-, ç-lactone, (5à)-	366	490	1.4	56143-34-1	mainlib
		5	Pregnan-18-oic acid, 20-hydroxy-, (5à)-	364	468	1.3	56143-33-0	mainlib
		6	2-Thiophenecarboxaldehyde, 5-ethynyl-, (2,4-dinitrophenyl)hydrazone	363	461	1.2	56588-21-7	mainlib
		7	16-Hydroxymethyleneandrost-5-en-3-ol-17-one	362	441	1.2		mainlib
		8	Dodecanoic acid, 1a,2,5,5a,6,9,10,10a-octahydro-5a-hydroxy-4-(hydroxymethyl)-1,1,7,9-tetramethyl-6,11-dioxo-1H-2,8a-methanocyclopenta[a]cyclopropa[e]cyclodecen-5-yl ester, [1aR-(1aà,2à,5á,5aá,8aà,9à,10aà)]-	359	359	1.0	77508-68-0	mainlib
		9	17a-Methyl-3á-methoxy-17a-aza-D-homoandrost-5-ene-17-one	356	440	0.9	149942-10-9	mainlib
		10	2-Phenanthrenecarboxylic acid, 1-(1,3-dithian-2-ylmethyl)-7-hydroxy-2,4b-dimethyl-1,2,3,4,4a,4b,5,6,7,8,10,10a-dodecahydro-, methyl ester	356	439	0.9		mainlib

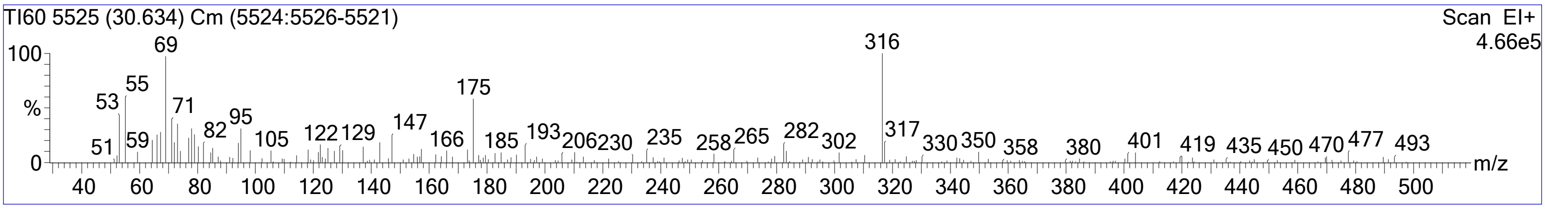

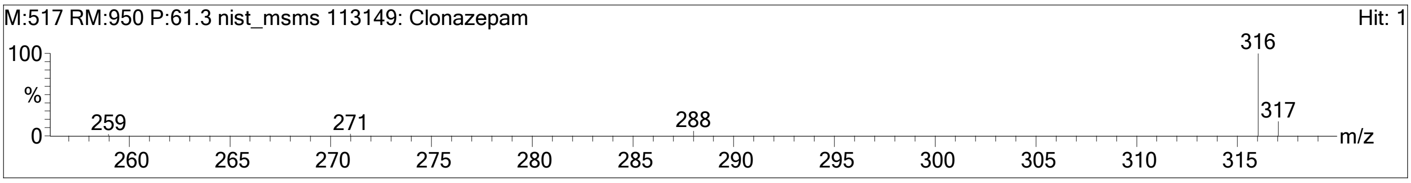

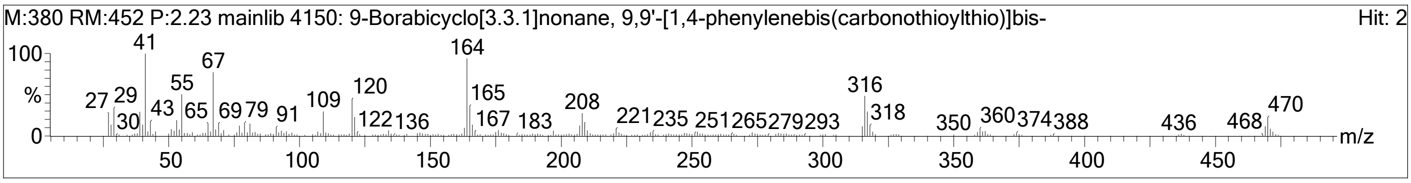

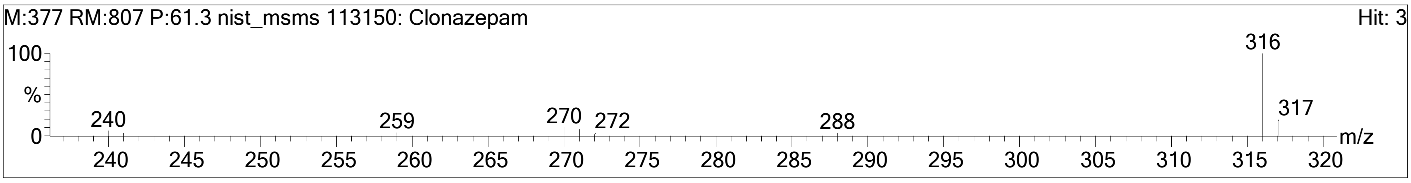

TAMILNADU AGRICULTURAL UNIVERSITY - AGRICULTURAL MICROBIOLOGY

INSTRUMENT: PERKIN ELMER CLARUS SQ8C COLOUMN: DB-5 MS CAPILARY STANDARD NON - POLARCOLOUMN
INJECTION VOL: 1 MICRO LITER DIMENSION: 30Mts, ID: 0.25 mm, FILM: 0.25 IM CARRIER GAS: He
SAMPLE ID : TI60

#	RT	Scan	Height	Area	Area %	Norm %
17	30.699	5538	10,361,085	670,193.8	1.075	15.57

Pk #	RT	Hit	Compound Name	Match	R.Match	Prob.	CAS	Library
17	30.699	1	17á-Acetoxy-1',1'-dicarboethoxy-1á,2á-dihydro-17à-methyl-3'H-cycloprop[1,2]-5à-androst-1-en-3-one	421	432	17.0	80097-22-9	mainlib
		2	Oleic acid, eicosyl ester	395	428	5.1	22393-88-0	mainlib
		3	2-Nonadecanone 2,4-dinitrophenylhydrazine	389	431	4.0	28813-61-8	mainlib
		4	4H-Cyclopropa[5',6']benz[1',2':7,8]azuleno[5,6]oxiren-4-one, 8,8a-bis(acetyloxy)-2a-[(acetyloxy)methyl]-1,1a,1b,1c,2a,3,3a,6a,6b,7,8,8a-dodecahydro-6b-hydroxy-3a-methoxy-1,1,5,7-tetramethyl-, [1aR-(1aà,1bá,1cá,2aá,3aà,6aà,6bà,7à,8á,8aà)]-	379	397	2.8	64838-69-3	mainlib
		5	9-Octadecenoic acid (Z)-, octadecyl ester	378	413	2.7	17673-49-3	mainlib
		6	Coumarin-6-ol, 3,4-dihydro-4,4-dimethyl-5,7-dinitro-	376	472	2.5		mainlib
		7	2,5-Furandione, dihydro-3-octadecyl-	368	431	1.9	47458-32-2	mainlib
		8	Oleyl oleate	368	392	1.9	3687-45-4	replib
		9	12-Hydroxyoctadecanethioic acid, S-t-butyl ester	367	466	1.8	58587-08-9	mainlib
		10	Oleic Acid	365	450	1.6	112-80-1	replib

TI60 5538 (30.699) Cm (5537:5539-5529) Scan EI+ 4.09e5

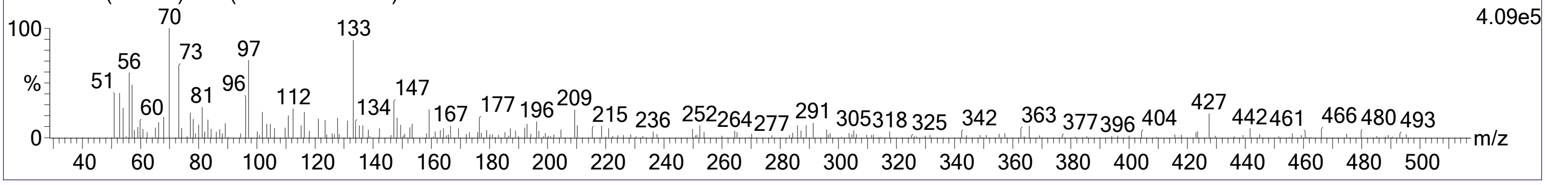

M:421 RM:432 P:17. mainlib 14237: 17á-Acetoxy-1',1'-dicarboethoxy-1á,2á-dihydro-17à-methyl-3'H-cycloprop[1,2]-5à-androst-1-en-3-one Hit: 1

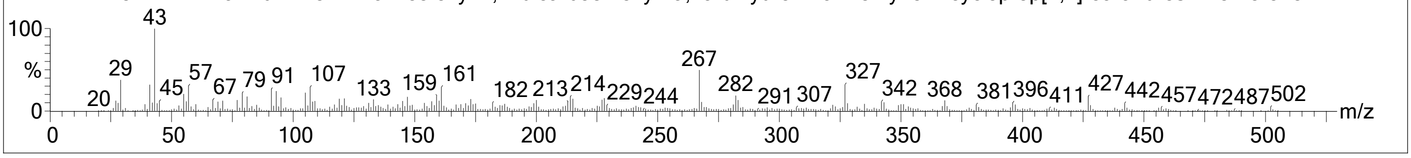

M:395 RM:428 P:5.08 mainlib 27467: Oleic acid, eicosyl ester Hit: 2

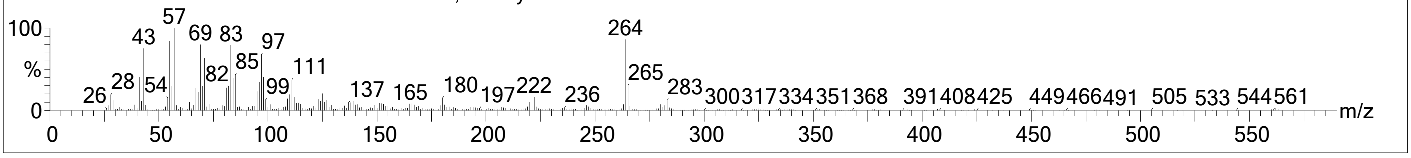

M:389 RM:431 P:3.99 mainlib 24752: 2-Nonadecanone 2,4-dinitrophenylhydrazine Hit: 3

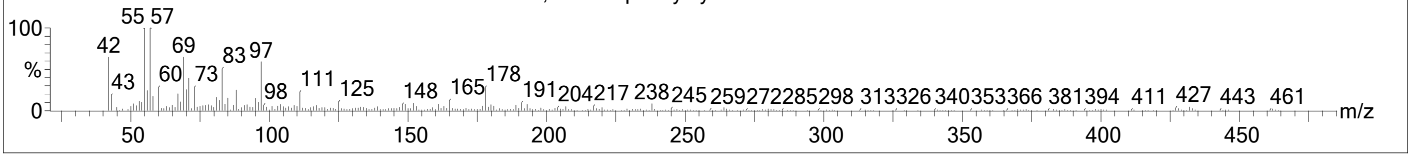

TAMILNADU AGRICULTURAL UNIVERSITY - AGRICULTURAL MICROBIOLOGY

INSTRUMENT: PERKIN ELMER CLARUS SQ8C COLOUMN: DB-5 MS CAPILARY STANDARD NON - POLARCOLOUMN
INJECTION VOL: 1 MICRO LITER DIMENSION: 30Mts, ID: 0.25 mm, FILM: 0.25 IM CARRIER GAS: He
SAMPLE ID : TI60

#	RT	Scan	Height	Area	Area %	Norm %
18	30.759	5550	10,673,964	600,823.9	0.964	13.96

Pk #	RT	Hit	Compound Name	Match	R.Match	Prob.	CAS	Library
18	30.759	1	9,19-Cyclolanostan-3-ol, 24,24-epoxymethano-, acetate	388	412	9.5		mainlib
		2	Pregnan-20-one, 5,6-epoxy-3,17-dihydroxy-16-methyl-, (3á,5à,6à,16à)-	377	414	6.5	56630-87-6	mainlib
		3	1-(á-d-Arabinofuranosyl)-4-difluoromethyl-5-bromouracil	371	474	5.1	102302-68-1	mainlib
		4	Pregnan-20-one, 3,11-dihydroxy-, (3á,5à,11à)-	367	440	4.3	565-91-3	mainlib
		5	16-Allopregnen-3á-ol-20-one	363	433	3.7	566-61-0	mainlib
		6	d-Mannitol, 1-decylsulfonyl-	362	484	3.5		mainlib
		7	3á-Acetoxy-17á-methyl-16-d-homoandrosten-17a-one	359	505	3.1		mainlib
		8	Pregnenolone	352	404	2.4	145-13-1	mainlib
		9	9,19-Cyclo-25,26-epoxyergostan-3-ol, 4,4,14-trimethyl-, acetate	352	376	2.4		mainlib
		10	1-(á-d-Ribofuranosyl)-4-difluoromethyl-5-bromouracil	351	463	2.3	102302-61-4	mainlib

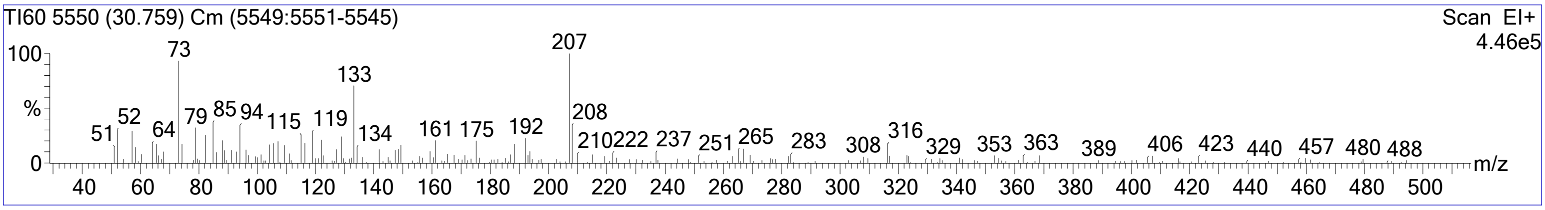

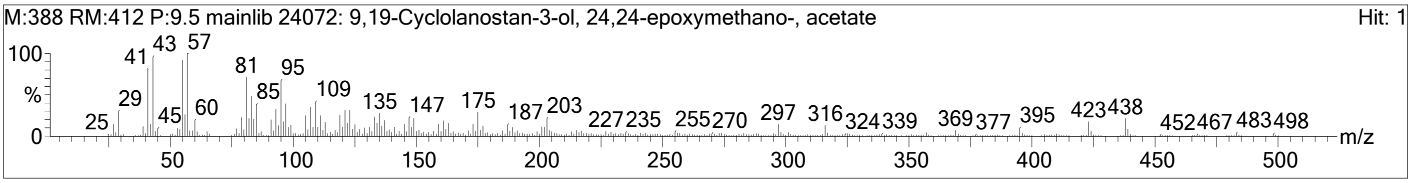

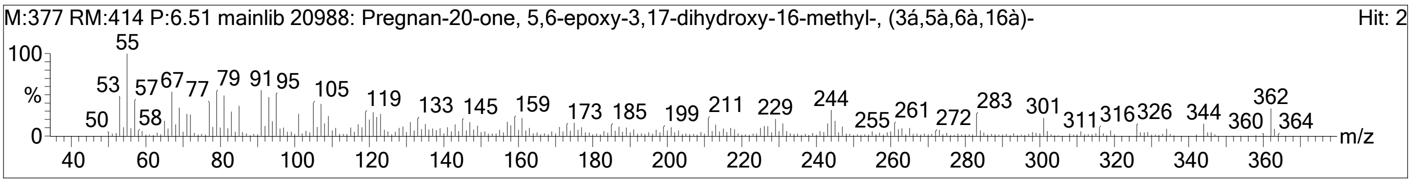

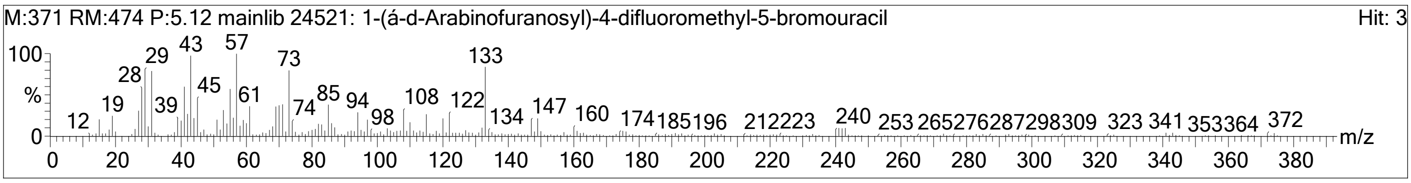

TAMILNADU AGRICULTURAL UNIVERSITY - AGRICULTURAL MICROBIOLOGY

INSTRUMENT: PERKIN ELMER CLARUS SQ8C COLOUMN: DB-5 MS CAPILARY STANDARD NON - POLARCOLOUMN
INJECTION VOL: 1 MICRO LITER DIMENSION: 30Mts, ID: 0.25 mm, FILM: 0.25 IM CARRIER GAS: He
SAMPLE ID : TI60

#	RT	Scan	Height	Area	Area %	Norm %
19	30.919	5582	8,685,801	345,571.8	0.554	8.03

Pk #	RT	Hit	Compound Name	Match	R.Match	Prob.	CAS	Library
19	30.919	1	3-Isopropyl-6a,10b-dimethyl-8-(2-oxo-2-phenyl-ethyl)-dodecahydro-benzo[f]chromen-7-one	411	459	11.9		mainlib
		2	3-Isopropyl-6a,7,10b-trimethyl-8-(2-oxo-2-phenylethyl)dodecahydrobenzo[f]chromene-7-carboxylic acid, methyl ester	407	421	10.0		mainlib
		3	.psi.,.psi.-Carotene, 1,1',2,2'-tetrahydro-1,1'-dimethoxy-	404	405	8.9	13833-01-7	mainlib
		4	d-Mannitol, 1-decylsulfonyl-	390	494	5.5		mainlib
		5	Thymol, TBDMS derivative	379	558	3.8	330455-64-6	mainlib
		6	9-Octadecenoic acid, (2-phenyl-1,3-dioxolan-4-yl)methyl ester, cis-	379	419	3.8	56599-45-2	mainlib
		7	4,4,6a,6b,8a,11,11,14b-Octamethyl-docosahydropicen-3-ol	373	400	3.0		mainlib
		8	2-Picenol, 4,4,6a,6b,8a,11,11,14b-octamethylperhydro	372	391	2.9		mainlib
		9	Sebacic acid, 4-bromo-2,6-difluorobenzyl isobutyl ester	370	422	2.7		mainlib
		10	Sebacic acid, 4-bromo-2,6-difluorobenzyl propyl ester	370	420	2.7		mainlib

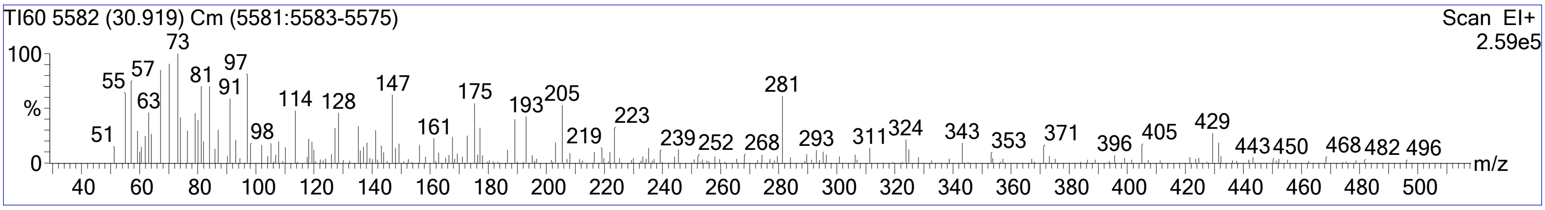

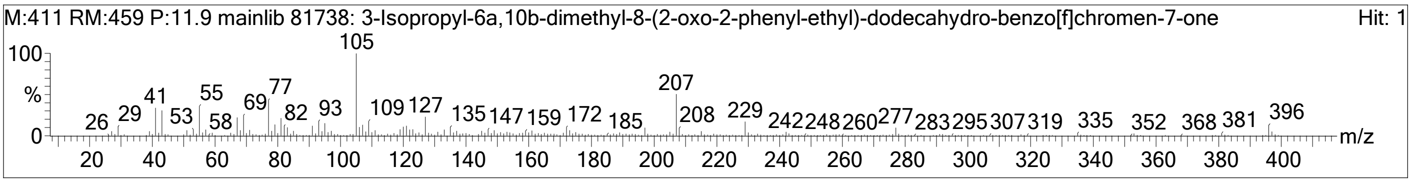

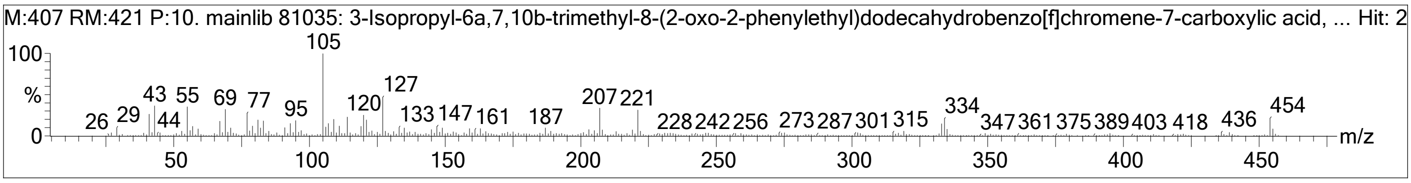

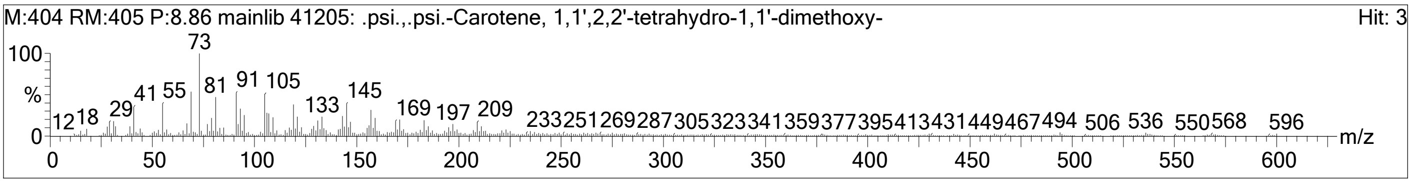

TAMILNADU AGRICULTURAL UNIVERSITY - AGRICULTURAL MICROBIOLOGY

INSTRUMENT: PERKIN ELMER CLARUS SQ8C COLOUMN: DB-5 MS CAPILARY STANDARD NON - POLARCOLOUMN
INJECTION VOL: 1 MICRO LITER DIMENSION: 30Mts, ID: 0.25 mm, FILM: 0.25 IM CARRIER GAS: He
SAMPLE ID : TI60

#	RT	Scan	Height	Area	Area %	Norm %
20	31.009	5600	8,529,466	270,024.3	0.433	6.27

Pk #	RT	Hit	Compound Name	Match	R.Match	Prob.	CAS	Library
20	31.009	1	17-(1,5-Dimethylhexyl)-10,13-dimethyl-3-styrylhexadecahydrocyclopenta[a]phenanthren-2-one	426	440	19.5		mainlib
		2	Octadecane, 3-ethyl-5-(2-ethylbutyl)-	421	457	15.7	55282-12-7	mainlib
		3	1,3-Dioxane, 4-(2-bromo-1-hydroxyethyl)-5,6-dimethyl-2-phenyl-	392	486	4.4		mainlib
		4	Octadecane, 5,14-dibutyl-	383	443	3.2	55282-13-8	mainlib
		5	Heptadecane, 9-hexyl-	382	433	3.1	55124-79-3	mainlib
		6	Octadecane, 3-ethyl-5-(2-ethylbutyl)-	377	451	15.7	55282-12-7	replib
		7	1-Dodecanol, 3,7,11-trimethyl-	370	502	2.1	6750-34-1	replib
		8	1-Chloroeicosane	370	454	2.1	42217-02-7	mainlib
		9	4-Methyldocosane	364	480	1.6	25117-30-0	mainlib
		10	Tetratetracontane	364	417	1.6	7098-22-8	replib

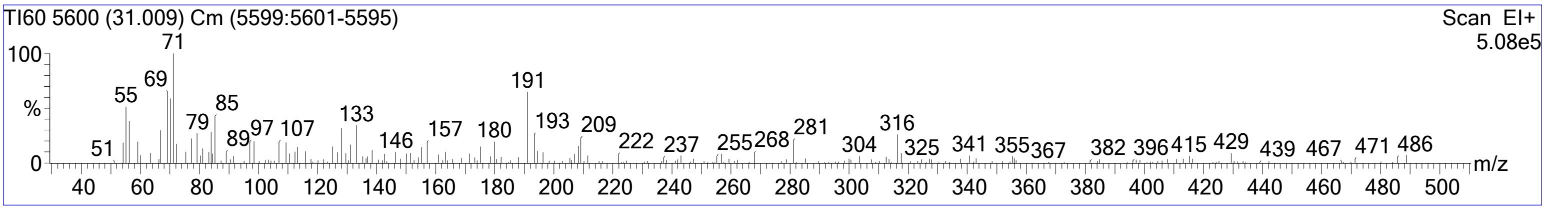

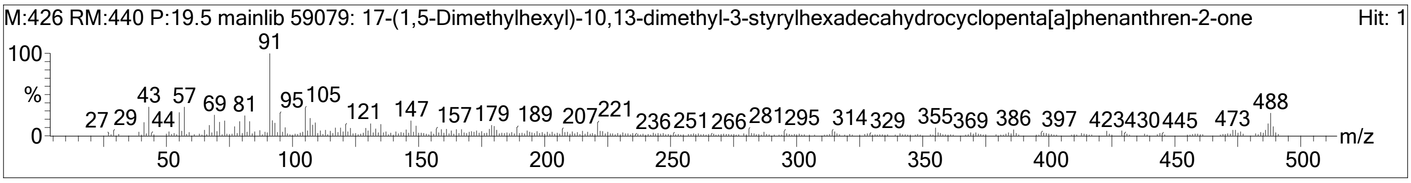

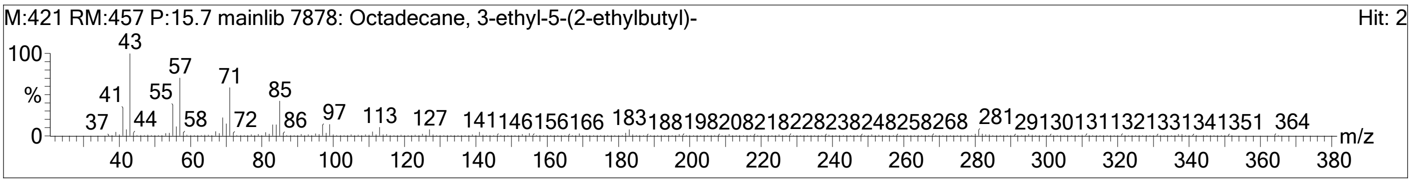

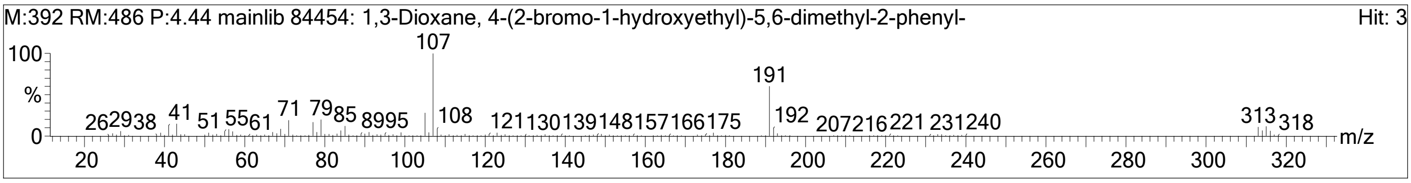

TAMILNADU AGRICULTURAL UNIVERSITY - AGRICULTURAL MICROBIOLOGY

INSTRUMENT: PERKIN ELMER CLARUS SQ8C COLOUMN: DB-5 MS CAPILARY STANDARD NON - POLARCOLOUMN
INJECTION VOL: 1 MICRO LITER DIMENSION: 30Mts, ID: 0.25 mm, FILM: 0.25 IM CARRIER GAS: He
SAMPLE ID : TI60

#	RT	Scan	Height	Area	Area %	Norm %
21	31.069	5612	7,490,828	326,423.5	0.524	7.58

Pk #	RT	Hit	Compound Name	Match	R.Match	Prob.	CAS	Library
21	31.069	1	(+)-Prostaglandin F2à, 4TMS derivative	356	377	7.5	50669-95-9	mainlib
		2	2-Thiophenecarboxylic acid, 4-(chloromethyl)-5-nonyl-, methyl ester	341	426	4.5	59782-43-3	mainlib
		3	Butyl 9-octadecenoate or 9-18:1	340	429	4.4		mainlib
		4	Allopegnan-3á,9à-diol-20-one	332	410	3.2		mainlib
		5	Acetylandromedol	330	401	3.0	4720-09-6	mainlib
		6	cis-10-Pentadecenoic acid, isobutyl ester	327	507	2.7		mainlib
		7	Acetic acid, 3,4-diacetoxy-7-cyano-1,4a,7-trimethyl-8-(2-oxoethyl)tetradecahydrophenanthren-2-yl ester	325	348	2.4		mainlib
		8	i-Propyl 11,12-methylene-octadecanoate	323	434	2.2		mainlib
		9	1,1,2,2,4,4-Hexa-t-butyl-3,5-dioxa-1,2,4-trisilolane	322	405	2.2	93194-13-9	mainlib
		10	3-Methoxy-D-homoestra-1,3.5(10)-trien-14-á,17aà-diol (.8alpha.)	319	490	1.9		mainlib

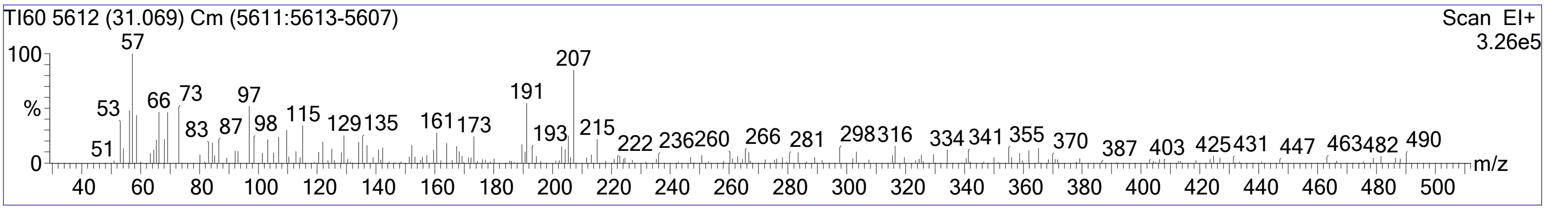

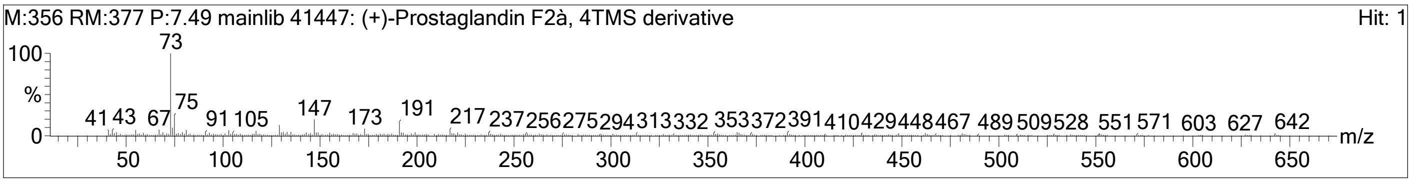

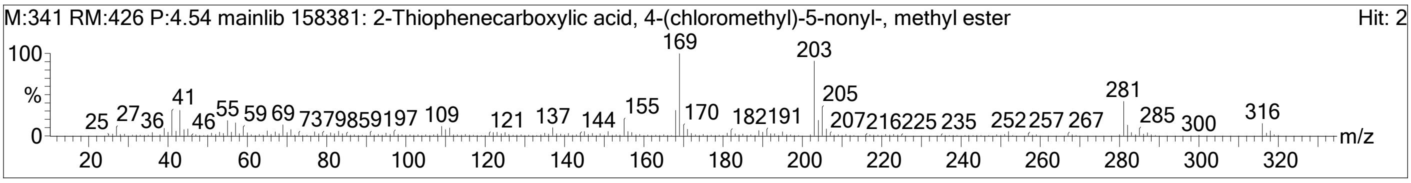

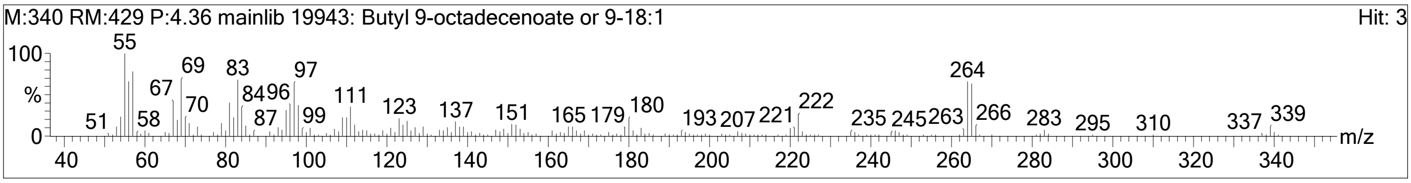

TAMILNADU AGRICULTURAL UNIVERSITY - AGRICULTURAL MICROBIOLOGY

INSTRUMENT: PERKIN ELMER CLARUS SQ8C   COLOUMN: DB-5 MS CAPILARY STANDARD NON - POLARCOLOUMN
INJECTION VOL: 1 MICRO LITER   DIMENSION: 30Mts, ID: 0.25 mm, FILM: 0.25 IM   CARRIER GAS: He
SAMPLE ID : TI60

#	RT	Scan	Height	Area	Area %	Norm %
22	31.124	5623	6,563,559	607,000.8	0.974	14.10

Pk #	RT	Hit	Compound Name	Match	R.Match	Prob.	CAS	Library
22	31.124	1	Strychane, 1-acetyl-20à-hydroxy-16-methylene-	379	432	20.2	2111-98-0	mainlib
		2	Propanoic acid, 2-(3-acetoxy-4,4,14-trimethylandrost-8-en-17-yl)-	377	395	18.6		mainlib
		3	2,6-Dihydroxyacetophenone, 2TMS derivative	346	400	5.1		mainlib
		4	Aspidospermidine, 1,2-didehydro-, (5à,12á,19à)-	339	422	3.9	56245-51-3	mainlib
		5	2,4-Dihydroxyacetophenone, 2TMS derivative	339	394	3.9		mainlib
		6	Carbamic acid, N-[10,11-dihydro-5-(2-methylamino-1-oxoethyl)-3-5H-dibenzo[b,f]azepinyl]-, ethyl ester	339	391	3.9	102821-92-1	mainlib
		7	Acridin-1(2H)-one, 3,4-dihydro-9-allylamino-3,3-dimethyl-	338	393	3.7	300359-68-6	mainlib
		8	2,5-Ethano-2H-azocino[4,3-b]indole, 4-ethylidene-1,3,4,5,6,7-hexahydro-6-methylene-, [r-(E)]-	335	419	3.3	3463-93-2	mainlib
		9	1-Methoxy-4-nitro-2,3,5,6-tetramethylbenzene	325	484	2.3	52415-08-4	mainlib
		10	Glycine, N-[(3à,5á)-24-oxo-3-[(trimethylsilyl)oxy]cholan-24-yl]-, methyl ester	323	369	2.1	57326-15-5	mainlib

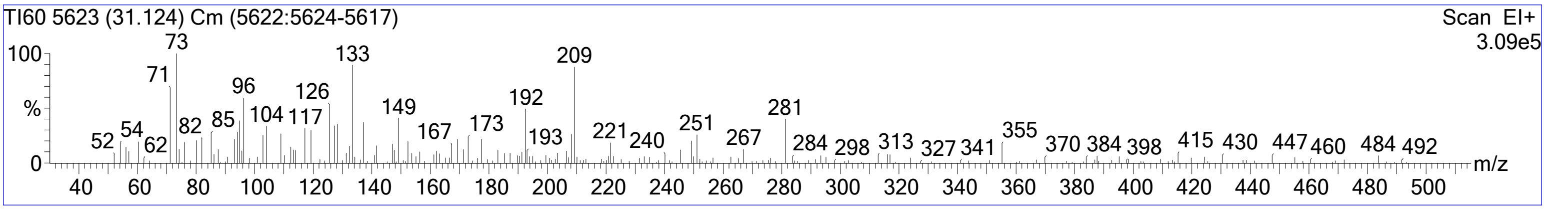

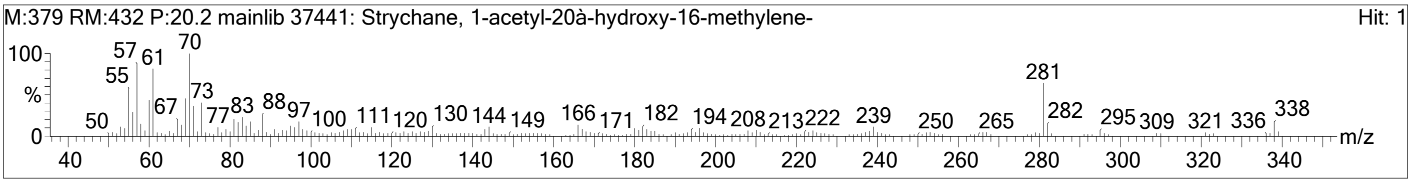

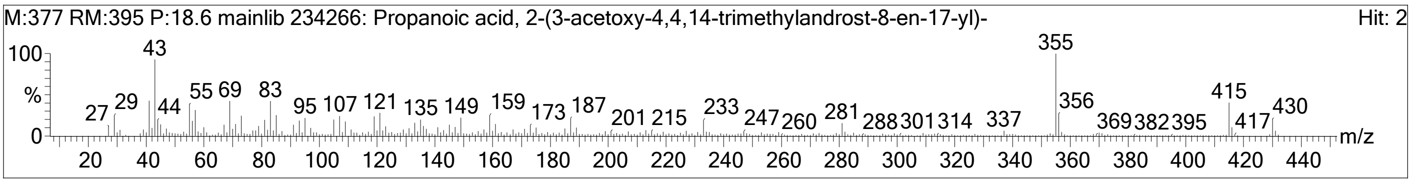

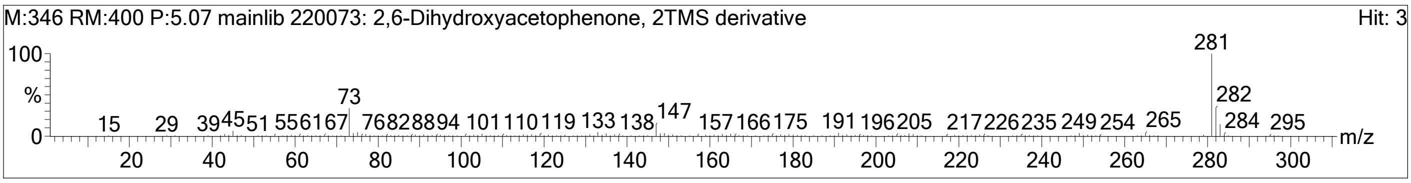

TAMILNADU AGRICULTURAL UNIVERSITY - AGRICULTURAL MICROBIOLOGY

INSTRUMENT: PERKIN ELMER CLARUS SQ8C COLOUMN: DB-5 MS CAPILARY STANDARD NON - POLARCOLOUMN
INJECTION VOL: 1 MICRO LITER DIMENSION: 30Mts, ID: 0.25 mm, FILM: 0.25 IM CARRIER GAS: He
SAMPLE ID : TI60

#	RT	Scan	Height	Area	Area %	Norm %
23	31.530	5704	5,010,848	273,521.4	0.439	6.36

Pk #	RT	Hit	Compound Name	Match	R.Match	Prob.	CAS	Library
23	31.530	1	Pregn-5-ene-3,11-dione, 17,20:20,21-bis[methylenebis(oxy)]-, cyclic 3-(1,2-ethanediyl acetal)	373	423	9.9	52248-40-5	mainlib
		2	Octadecanoic acid, (2-phenyl-1,3-dioxolan-4-yl)methyl ester, cis-	372	427	9.5	56599-88-3	mainlib
		3	(5á)Pregnane-3,20á-diol, 14à,18à-[4-methyl-3-oxo-(1-oxa-4-azabutane-1,4-diyl)]-, diacetate	369	393	8.4		mainlib
		4	7aH-Cyclopenta[a]cyclopropa[f]cycloundecene-2,4,7,7a,10,11-hexol, 1,1a,2,3,4,4a,5,6,7,10,11,11a-dodecahydro-1,1,3,6,9-pentamethyl-, 2,4,7,10,11-pentaacetate	362	386	6.4	51906-08-2	mainlib
		5	Cholestan-3-one, cyclic 1,2-ethanediyl aetal, (5á)-	353	409	4.7	25328-53-4	mainlib
		6	Propenenitrile, 2-(4-chlorophenylsulfonyl)-3-cycllohexylamino-	345	411	3.5		mainlib
		7	Spirost-8-en-11-one, 3-hydroxy-, (3á,5à,14á,20á,22á,25R)-	338	386	2.7	58072-54-1	mainlib
		8	18,19-Secoyohimban-19-oic acid, 16,17,20,21-tetradehydro-16-(hydroxymethyl)-, methyl ester, (15á,16E)-	334	424	2.3	5523-49-9	mainlib
		9	Stearic acid, 2-phenyl-m-dioxan-5-yl ester, trans-	329	368	1.8	10564-35-9	mainlib
		10	3'H-Cycloprop(1,2)-5-cholest-1-en-3-one, 1'-carboethoxy-1'-cyano-1,2-dihydro-	329	338	1.8	75857-80-6	mainlib

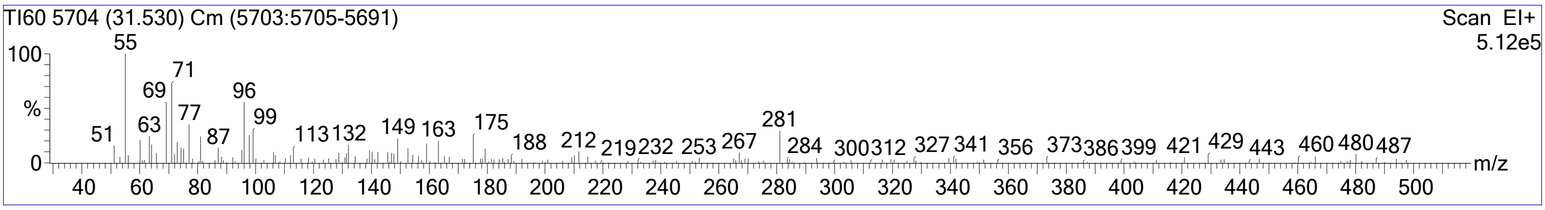

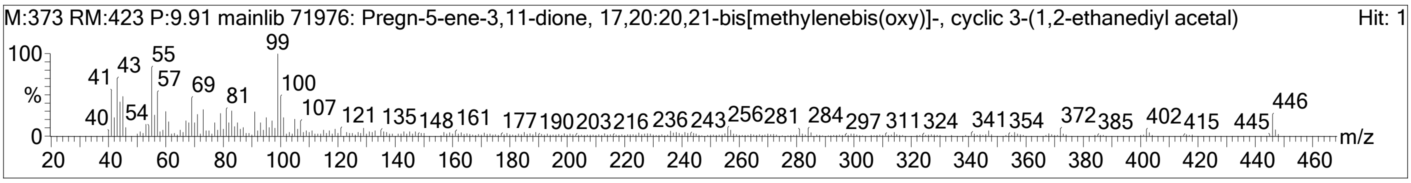

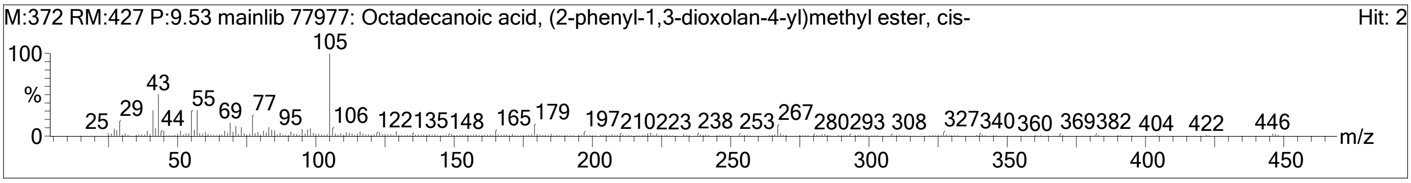

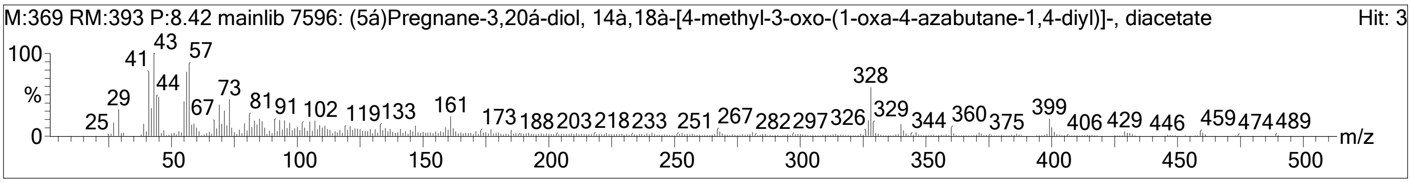

TAMILNADU AGRICULTURAL UNIVERSITY - AGRICULTURAL MICROBIOLOGY

INSTRUMENT: PERKIN ELMER CLARUS SQ8C COLOUMN: DB-5 MS CAPILARY STANDARD NON - POLARCOLOUMN
INJECTION VOL: 1 MICRO LITER DIMENSION: 30Mts, ID: 0.25 mm, FILM: 0.25 IM CARRIER GAS: He
SAMPLE ID : TI60

#	RT	Scan	Height	Area	Area %	Norm %
24	32.625	5923	7,678,466	348,128.7	0.559	8.09

Pk #	RT	Hit	Compound Name	Match	R.Match	Prob.	CAS	Library
24	32.625	1	Phosphorothioic acid, O-(4,5-dichloro-2-methoxyphenyl) O,O-dimethyl ester	468	529	27.0	55975-98-9	mainlib
		2	2,6-Dihydroxyacetophenone, 2TMS derivative	431	492	6.6		mainlib
		3	Propanoic acid, 2-(3-acetoxy-4,4,14-trimethylandrost-8-en-17-yl)-	430	447	6.3		mainlib
		4	2,5-Dihydroxyacetophenone, 2TMS derivative	421	474	4.6		mainlib
		5	2,4-Dihydroxyacetophenone, 2TMS derivative	415	467	3.6		mainlib
		6	Haloxazolam	414	460	3.5	59128-97-1	mainlib
		7	Octadecane, 1,1'-[1,3-propanediylbis(oxy)]bis-	410	427	2.9	17367-38-3	mainlib
		8	Benzenesulfonamide, N-(bicyclo[2.2.1]hept-5-en-2-ylmethyl)-4-(5-oxo-3-pyrrolidinyl)-	409	542	2.8		mainlib
		9	1,2,3-Triazole-4-carboxamide, N-(2,6-dichlorophenyl)-1-methyl-5-methylthio-	407	478	2.6		mainlib
		10	1,2-Cinnolinedicarboxylic acid, 1,2,3,5,6,7,8,8a-octahydro-4-trimethylsilyloxy-, diethyl ester	402	452	2.1	215712-69-9	mainlib

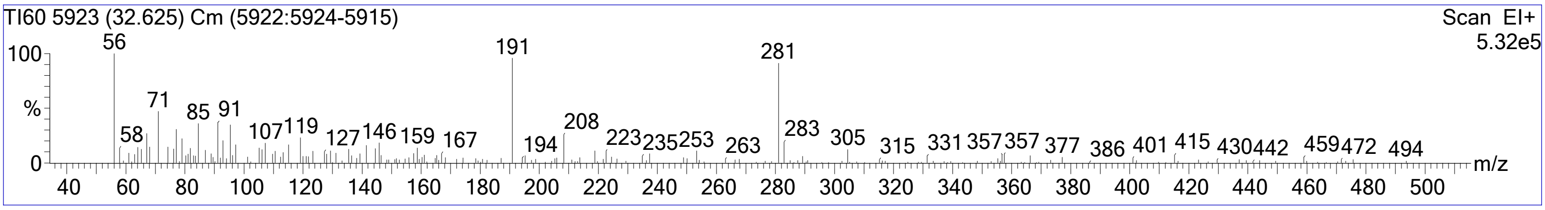

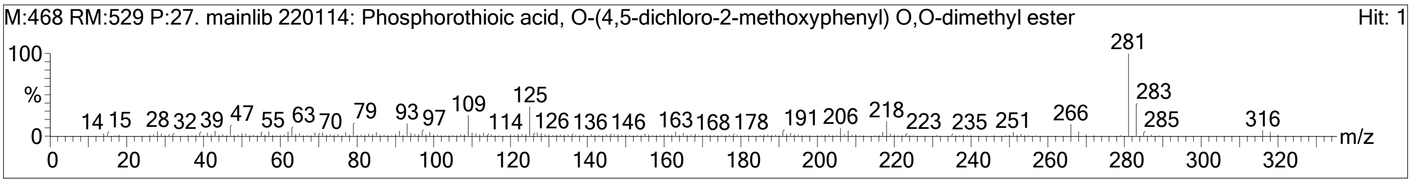

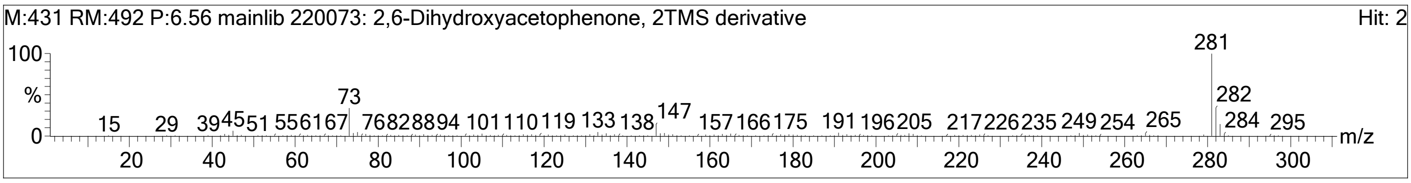

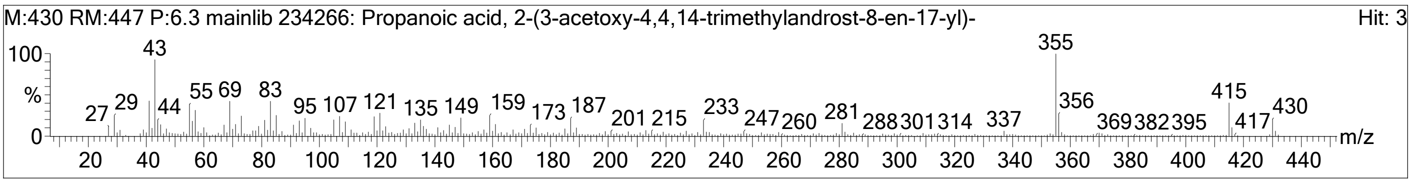

TAMILNADU AGRICULTURAL UNIVERSITY - AGRICULTURAL MICROBIOLOGY

INSTRUMENT: PERKIN ELMER CLARUS SQ8C COLOUMN: DB-5 MS CAPILARY STANDARD NON - POLARCOLOUMN
INJECTION VOL: 1 MICRO LITER DIMENSION: 30Mts, ID: 0.25 mm, FILM: 0.25 IM CARRIER GAS: He
SAMPLE ID : TI60

#	RT	Scan	Height	Area	Area %	Norm %
25	32.735	5945	9,274,968	376,123.0	0.603	8.74

Pk #	RT	Hit	Compound Name	Match	R.Match	Prob.	CAS	Library
25	32.735	1	1,8-Dioxa-5-thiaoctane, 8-(9-borabicyclo[3.3.1]non-9-yl)-3-(9-borabicyclo[3.3.1]non-9-yloxy)-1-phenyl-	400	462	8.1		mainlib
		2	Propanoic acid, 2-(3-acetoxy-4,4,14-trimethylandrost-8-en-17-yl)-	399	412	7.8		mainlib
		3	Ethyl 5-(([3-cyano-4-(methoxymethyl)-6-methylpyridin-2-yl]sulfanyl)methyl)-1,2-oxazole-3-carboxylate	384	461	4.7		mainlib
		4	3-Buten-2-one, 4-(2,6,6-trimethyl-2-cyclohexen-1-yl)-, (2,4-dinitrophenyl)hydrazone	377	420	3.6	6998-49-8	mainlib
		5	Glafenin	376	704	3.5	3820-67-5	nist_msms
		6	Hexasiloxane, 1,1,3,3,5,5,7,7,9,9,11,11-dodecamethyl-	373	484	3.1	995-82-4	mainlib
		7	trans-4-(2-(5-Nitro-2-furyl)vinyl)-2-quinolinamine	365	499	2.3	847-10-9	mainlib
		8	Phenol, 2,6-dichloro-4-nitro-	364	540	2.2	618-80-4	mainlib
		9	4á-Methylandrostane2,3-diol-1,17-dione	362	414	2.0		mainlib
		10	1,5,8-Trimethoxy-12a-methyl-1,2,3,3a,3b,4,5,6,7,8,9,10,10b,11,12,12a-hexadecahydro-benzo[3,4]cyclohepta[1,2-E]indene	361	398	1.9		mainlib

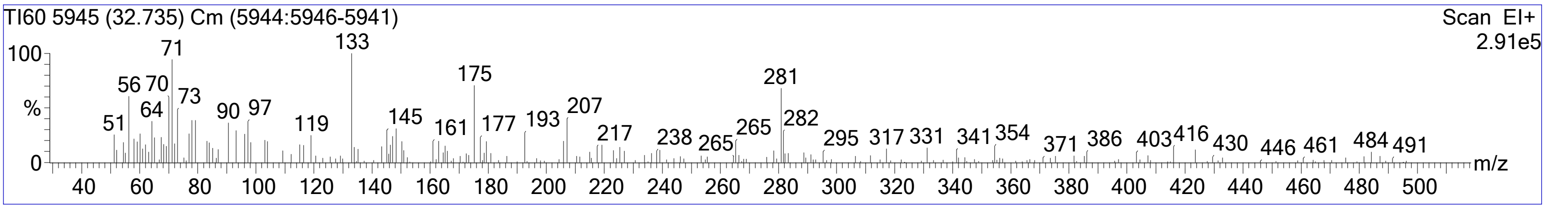

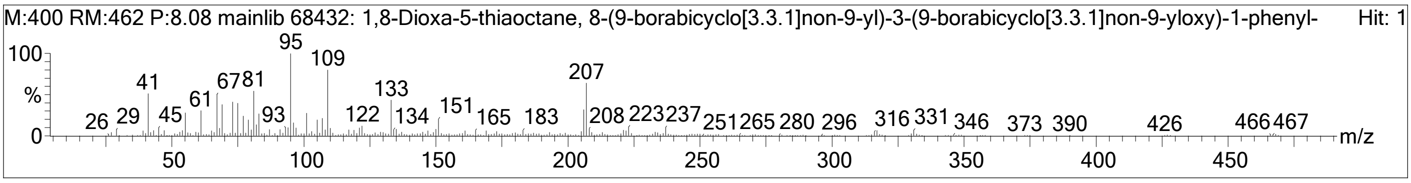

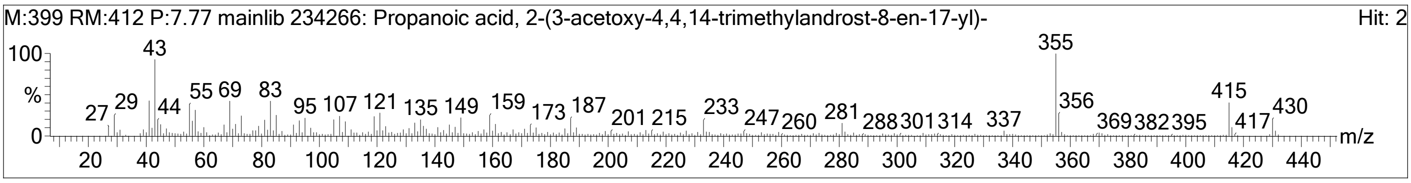

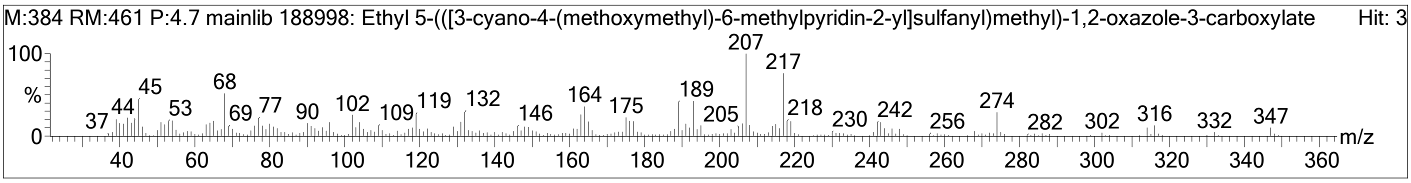

TAMILNADU AGRICULTURAL UNIVERSITY - AGRICULTURAL MICROBIOLOGY

INSTRUMENT: PERKIN ELMER CLARUS SQ8C COLOUMN: DB-5 MS CAPILARY STANDARD NON - POLARCOLOUMN
INJECTION VOL: 1 MICRO LITER DIMENSION: 30Mts, ID: 0.25 mm, FILM: 0.25 IM CARRIER GAS: He
SAMPLE ID : TI60

#	RT	Scan	Height	Area	Area %	Norm %
26	32.835	5965	10,323,956	585,406.2	0.939	13.60

Pk #	RT	Hit	Compound Name	Match	R.Match	Prob.	CAS	Library
26	32.835	1	5,5,6-Exo-8,9,10-hexachlorocamphene	363	419	9.7	165820-10-0	mainlib
		2	Propanedioic acid, mononitrile, 2-[tetrahydro-4-(4-fluorophenyl)-2,2-dimethyl-4-pyranyl]-, ethyl ester	362	448	9.4	120729-52-4	mainlib
		3	17á-Acetoxy-1',1'-dicarboethoxy-1á,2á-dihydro-17à-methyl-3'H-cycloprop[1,2]-5à-androst-1-en-3-one	360	364	8.6	80097-22-9	mainlib
		4	4,4,6a,6b,8a,11,12,14b-Octamethyl-docosahydropicene-3,13-diol	358	402	8.0		mainlib
		5	1-(2-Acetoxyethyl)-3,6-diazahomoadamantan-9-one oxime	338	422	3.6		mainlib
		6	8,13-Epoxy-labadan-1,6,7,9-tetraol-11-one, 7-O-acetate(ester)	330	412	2.7	66428-88-4	mainlib
		7	d-Homo-24-nor-17-oxachola-20,22-diene-3,16-dione, 7-(acetyloxy)-14,15:21,23-diepoxy-4,4,8-trimethyl-, (5à,7à,13à,14á,15á,17aà)-	329	339	2.6	2629-11-0	mainlib
		8	Silane, diphenyldodecyloxy(pent-4-en-1-yloxy)-	327	364	2.4		mainlib
		9	à-Conotoxin MI	325	388	2.2		nist_msms2
		10	6-Fluoro-2-trifluoromethylbenzoic acid, nonadecyl ester	316	447	1.6		mainlib

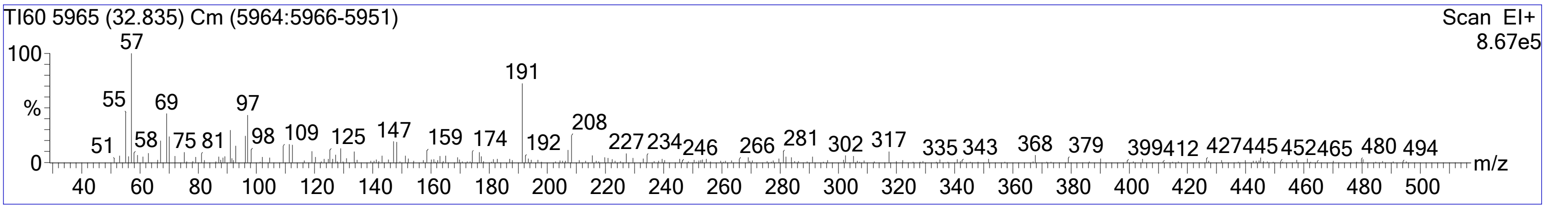

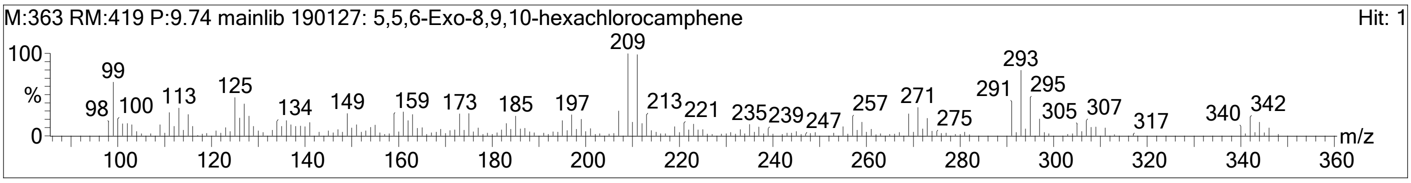

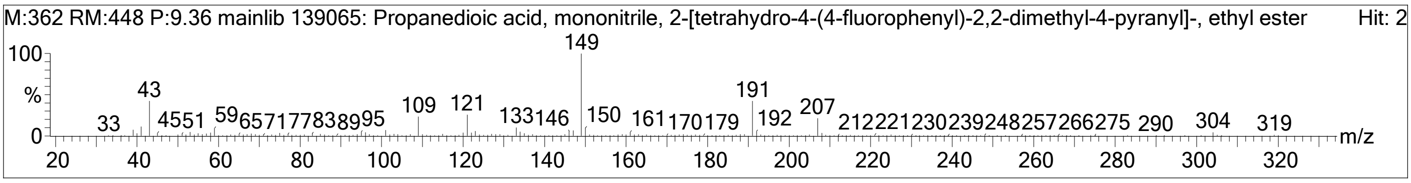

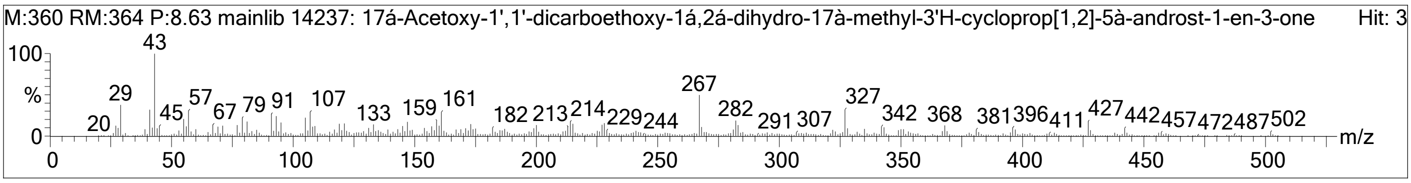

TAMILNADU AGRICULTURAL UNIVERSITY - AGRICULTURAL MICROBIOLOGY

INSTRUMENT: PERKIN ELMER CLARUS SQ8C COLOUMN: DB-5 MS CAPILARY STANDARD NON - POLARCOLOUMN
INJECTION VOL: 1 MICRO LITER DIMENSION: 30Mts, ID: 0.25 mm, FILM: 0.25 IM CARRIER GAS: He
SAMPLE ID : TI60

#	RT	Scan	Height	Area	Area %	Norm %
27	32.945	5987	9,896,200	451,159.4	0.724	10.48

Pk #	RT	Hit	Compound Name	Match	R.Match	Prob.	CAS	Library
27	32.945	1	Rhodopin	393	398	7.9	105-92-0	mainlib
		2	8-Amino-5-[3-chlorophenylthio]-6-methoxyquinoline	389	459	6.7	64895-56-3	mainlib
		3	17a-Methyl-3á-methoxy-17a-aza-D-homoandrost-5-ene-17-one	388	456	6.4	149942-10-9	mainlib
		4	1,1-Cyclobutanedicarboxamide, 2-phenyl-N,N'-bis(1-phenylethyl)-	382	409	5.0		mainlib
		5	Cholesta-8,24-dien-3-ol, 4-methyl-, (3á,4à)-	378	406	4.2	7199-92-0	mainlib
		6	3-Isopropyl-6a,7,10b-trimethyl-8-(2-oxo-2-phenylethyl)dodecahydrobenzo[f]chromene-7-carboxylic acid, methyl ester	378	399	4.2		mainlib
		7	3-[3-(3,4-Dimethoxy-phenyl)-acryloyl]-6-methyl-pyran-2,4-dione	376	532	3.9	59144-91-1	mainlib
		8	Spirost-8-en-11-one, 3-hydroxy-, (3á,5à,14á,20á,22á,25R)-	367	418	2.8	58072-54-1	mainlib
		9	Neronine, 4á,5-dihydro-	364	423	2.5	19483-30-8	mainlib
		10	Bicyclo[5.3.0]decan-2-one, 9-(diphenylmethylene)-	362	416	2.3	345938-61-6	mainlib

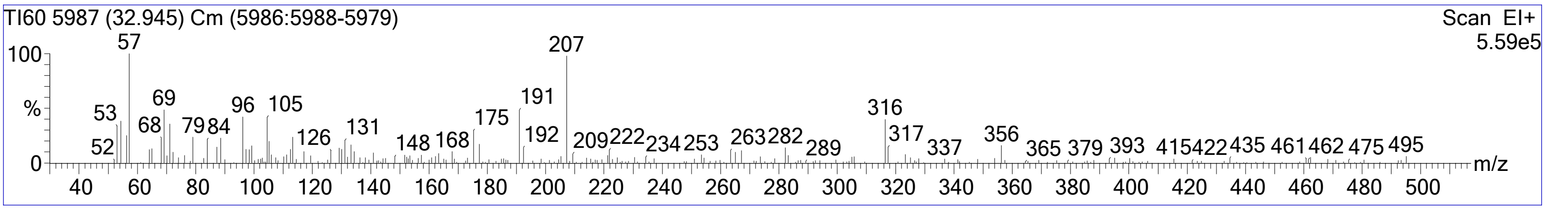

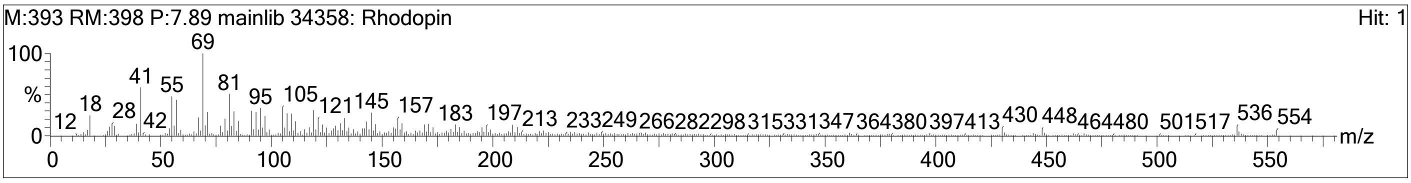

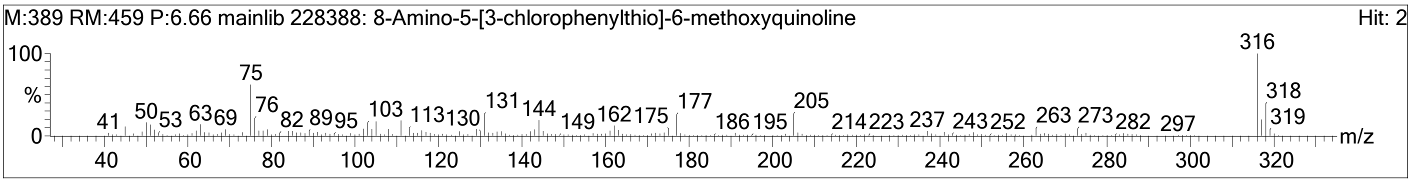

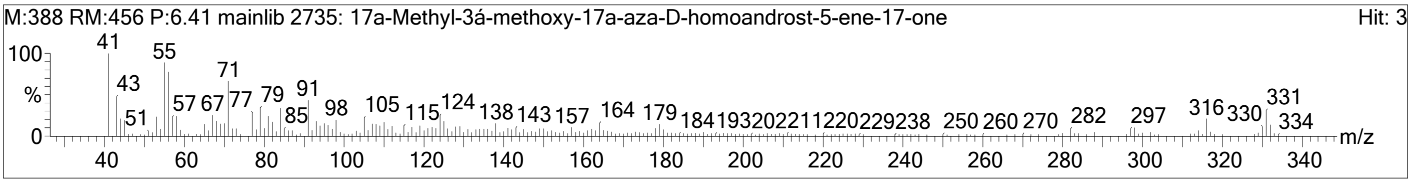

TAMILNADU AGRICULTURAL UNIVERSITY - AGRICULTURAL MICROBIOLOGY

INSTRUMENT: PERKIN ELMER CLARUS SQ8C COLOUMN: DB-5 MS CAPILARY STANDARD NON - POLARCOLOUMN
INJECTION VOL: 1 MICRO LITER DIMENSION: 30Mts, ID: 0.25 mm, FILM: 0.25 IM CARRIER GAS: He
SAMPLE ID : TI60

#	RT	Scan	Height	Area	Area %	Norm %
28	33.035	6005	10,013,573	770,499.3	1.236	17.90

Pk #	RT	Hit	Compound Name	Match	R.Match	Prob.	CAS	Library
28	33.035	1	10-Acetoxy-2-hydroxy-1,2,6a,6b,9,9,12a-heptamethyl-1,3,4,5,6,6a,6b,7,8,8a,9,10,11,12,12a,12b,13,14b-octadecahydro-2H-picene-4a-carboxylic acid, methyl ester	411	430	9.4	14356-56-0	mainlib
		2	Octadecane, 3-ethyl-5-(2-ethylbutyl)-	394	421	5.1	55282-12-7	mainlib
		3	2-Nonadecanone 2,4-dinitrophenylhydrazine	391	439	4.5	28813-61-8	mainlib
		4	Heptadecane, 9-hexyl-	391	427	4.5	55124-79-3	mainlib
		5	Pregn-5-en-20-one, 11-(acetyloxy)-3,14-dihydroxy-12-(2-hydroxy-3-methyl-1-oxobutoxy)-, (3á,11à,12á,14á)-	382	417	3.3	20230-37-9	mainlib
		6	Hexadecanoic acid, 1-(1-methylethyl)-1,2-ethanediyl ester	381	419	3.2	56599-93-0	mainlib
		7	Docosanoic acid, 1,2,3-propanetriyl ester	380	400	3.0	18641-57-1	mainlib
		8	Dodecanoic acid, 1a,2,5,5a,6,9,10,10a-octahydro-5a-hydroxy-4-(hydroxymethyl)-1,1,7,9-tetramethyl-6,11-dioxo-1H-2,8a-methanocyclopenta[a]cyclopropa[e]cyclodecen-5-yl ester, [1aR-(1aà,2à,5á,5aá,8aà,9à,10aà)]-	380	380	3.0	77508-68-0	mainlib
		9	1,2-Nonadecanediol	377	469	2.7	39516-65-9	mainlib
		10	Oleic acid, 3-(octadecyloxy)propyl ester	374	417	2.4	17367-41-8	mainlib

TI60 6005 (33.035) Cm (6004:6006-5991) Scan EI+ 5.68e5

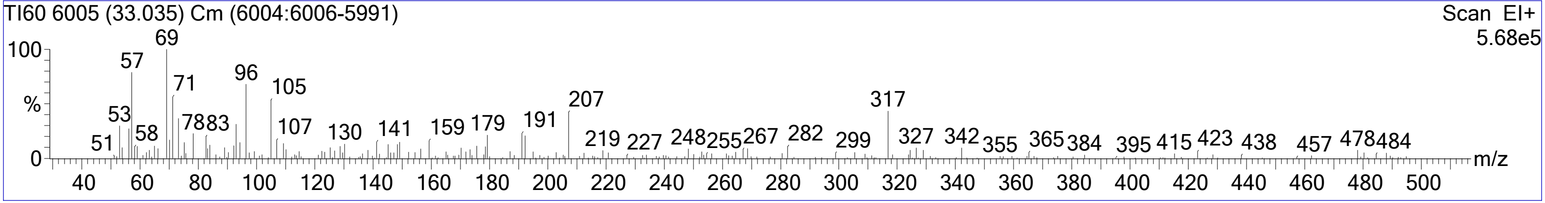

M:411 RM:430 P:9.35 mainlib 7689: 10-Acetoxy-2-hydroxy-1,2,6a,6b,9,9,12a-heptamethyl-1,3,4,5,6,6a,6b,7,8,8a,9,10,11,12,12a,12b,13,14b... Hit: 1

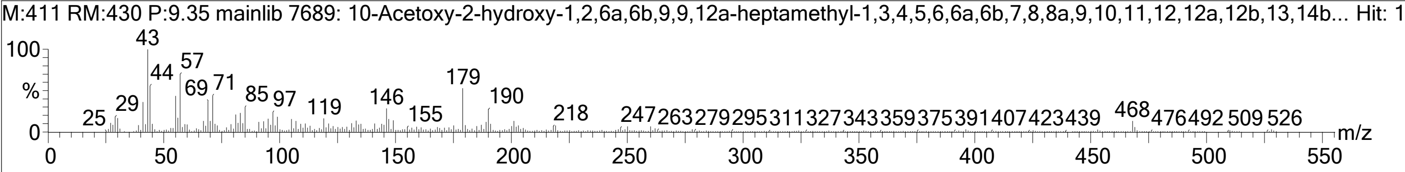

M:394 RM:421 P:5.1 mainlib 7878: Octadecane, 3-ethyl-5-(2-ethylbutyl)- Hit: 2

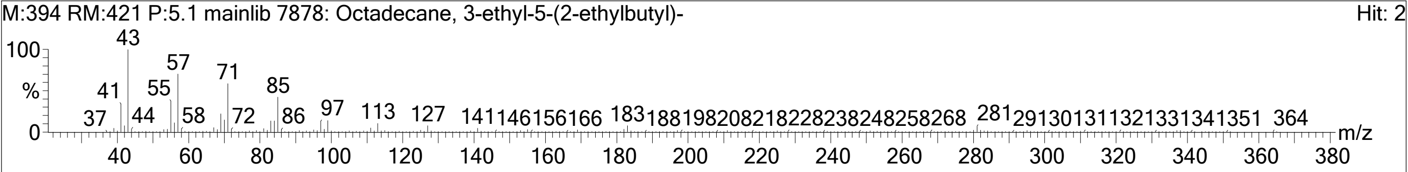

M:391 RM:439 P:4.51 mainlib 24752: 2-Nonadecanone 2,4-dinitrophenylhydrazine Hit: 3

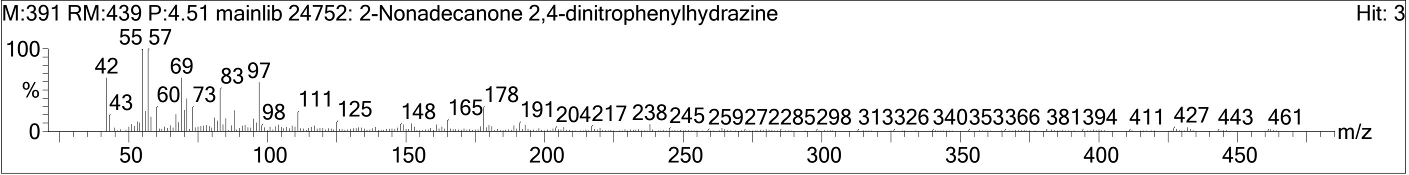

TAMILNADU AGRICULTURAL UNIVERSITY - AGRICULTURAL MICROBIOLOGY

INSTRUMENT: PERKIN ELMER CLARUS SQ8C COLOUMN: DB-5 MS CAPILARY STANDARD NON - POLARCOLOUMN
INJECTION VOL: 1 MICRO LITER DIMENSION: 30Mts, ID: 0.25 mm, FILM: 0.25 IM CARRIER GAS: He
SAMPLE ID : TI60

#	RT	Scan	Height	Area	Area %	Norm %
29	33.180	6034	8,248,066	296,008.5	0.475	6.88

Pk #	RT	Hit	Compound Name	Match	R.Match	Prob.	CAS	Library
29	33.180	1	7,8-Epoxylanostan-11-ol, 3-acetoxy-	429	431	7.7		mainlib
		2	1,8-Dioxa-5-thiaoctane, 8-(9-borabicyclo[3.3.1]non-9-yl)-3-(9-borabicyclo[3.3.1]non-9-yloxy)-1-phenyl-	417	479	5.1		mainlib
		3	Ursodeoxycholic acid	414	439	4.5	128-13-2	mainlib
		4	9,12-Octadecadienoic acid, (2-phenyl-1,3-dioxolan-4-yl)methyl ester, trans-	413	426	4.3	56599-48-5	mainlib
		5	Betulin	411	429	4.0	473-98-3	replib
		6	17.alfa.,21á-28,30-Bisnorhopane	410	497	3.8		mainlib
		7	Rhodopin	410	416	3.8	105-92-0	mainlib
		8	9,12-Octadecadienoic acid, (2-phenyl-1,3-dioxolan-4-yl)methyl ester, cis-	409	421	3.7	56599-47-4	mainlib
		9	29-Nor-8à,9á,13à,14á-dammaran-21-oic acid, 3à, 11à,16á-trihydroxy-, ç-lactone	406	450	3.3	4701-59-1	mainlib
		10	Pregnan-18-oic acid, 20-hydroxy-, (5à)-	404	484	3.0	56143-33-0	mainlib

TI60 6034 (33.180) Cm (6033:6035-6029) Scan EI+ 2.93e5

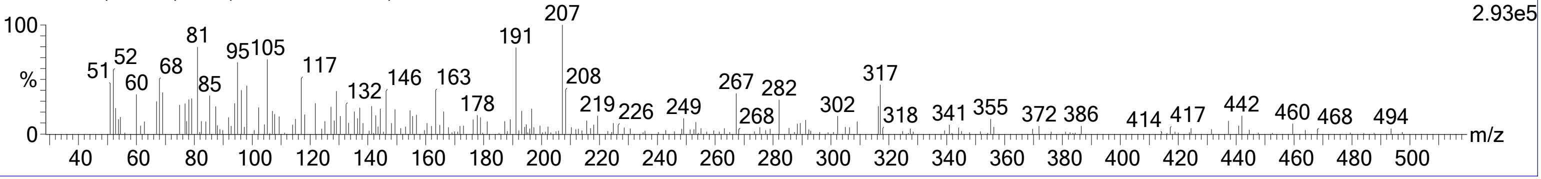

M:429 RM:431 P:7.69 mainlib 15359: 7,8-Epoxylanostan-11-ol, 3-acetoxy- Hit: 1

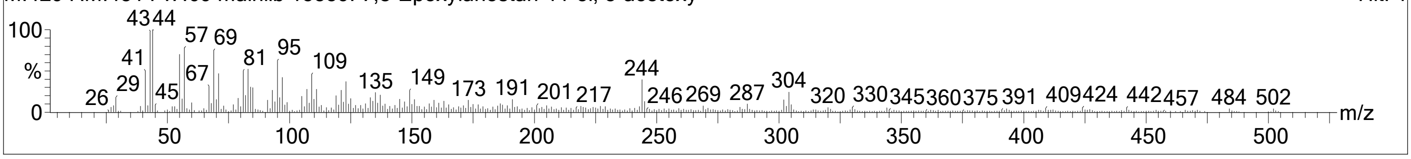

M:417 RM:479 P:5.12 mainlib 68432: 1,8-Dioxa-5-thiaoctane, 8-(9-borabicyclo[3.3.1]non-9-yl)-3-(9-borabicyclo[3.3.1]non-9-yloxy)-1-phenyl- Hit: 2

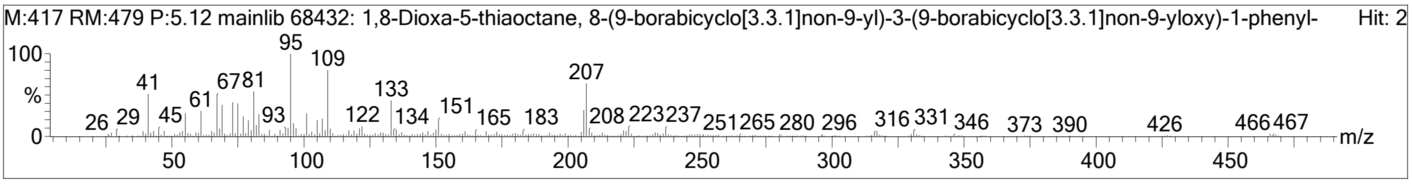

M:414 RM:439 P:4.52 mainlib 20532: Ursodeoxycholic acid Hit: 3

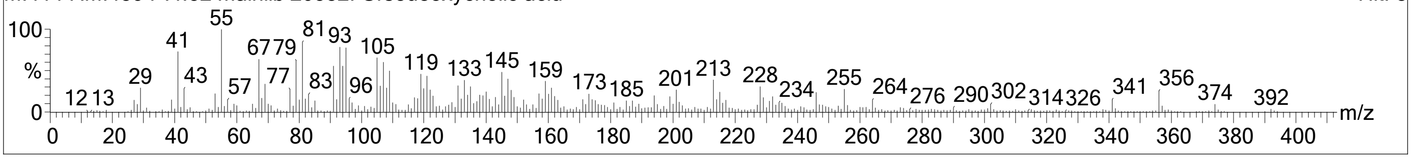

TAMILNADU AGRICULTURAL UNIVERSITY - AGRICULTURAL MICROBIOLOGY

INSTRUMENT: PERKIN ELMER CLARUS SQ8C COLOUMN: DB-5 MS CAPILARY STANDARD NON - POLARCOLOUMN
INJECTION VOL: 1 MICRO LITER DIMENSION: 30Mts, ID: 0.25 mm, FILM: 0.25 IM CARRIER GAS: He
SAMPLE ID : TI60

#	RT	Scan	Height	Area	Area %	Norm %
30	33.310	6060	7,496,352	302,432.3	0.485	7.03

Pk #	RT	Hit	Compound Name	Match	R.Match	Prob.	CAS	Library
30	33.310	1	l-Methionine, N-(5-chlorovaleryl)-, methyl ester	376	517	8.7		mainlib
		2	1,2-Benzisothiazol-3-amine, TMS derivative	358	491	4.5		mainlib
		3	1,2-Benzisothiazol-3-amine, TBDMS derivative	353	498	3.6		mainlib
		4	Betulin	350	364	3.2	473-98-3	replib
		5	8H-Pyrano[3,4-b]pyrimido[5,4-d]furane, 5,6-dihydro-4-hydrazino-6,6-dimethyl-2-methylthio-	349	403	3.1	253146-95-1	mainlib
		6	5-Bromo-4-nitroimidazole-2-[2-thioacetic acid]	348	494	3.0		mainlib
		7	Acetic acid, 2,3-dibromo-4-methoxymethoxy-1-methyl-pent-2-enyl ester	348	451	3.0		mainlib
		8	2,4,6-Cycloheptatrien-1-one, 3,5-bis-trimethylsilyl-	347	465	2.8		mainlib
		9	Acetamide, 2-chloro-N-(3-cyano-4,6-dihydro-4,4,6,6-tetramethylthieno[2,3-c]furan-2-yl)-	345	439	2.6		mainlib
		10	Adamantane-2,6-dione, bis(ethylene ketal)-	344	435	2.5	60797-89-9	mainlib

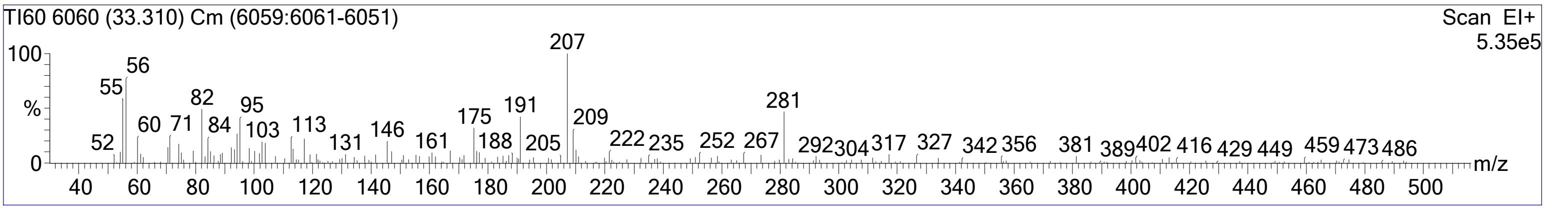

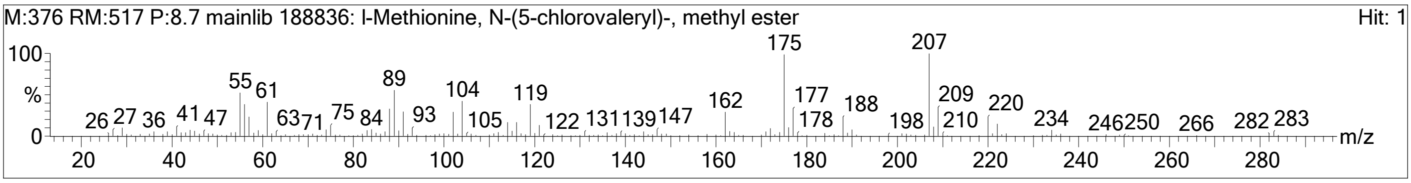

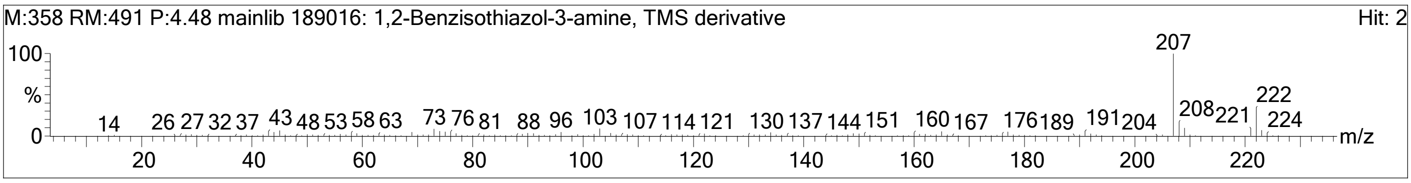

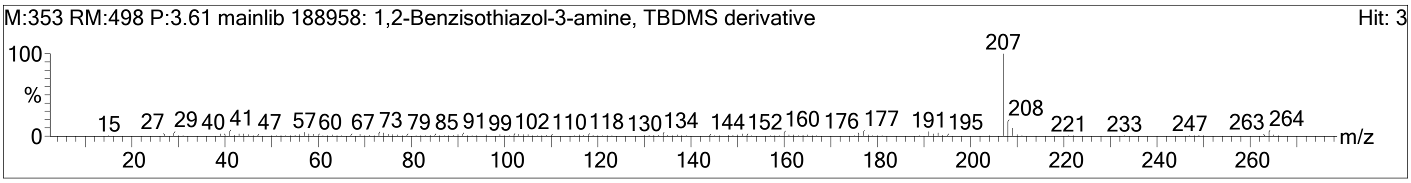

Supplement: S3 Fig — (PDF) [file pone.0219014.s009.pdf]
