# Supplementary material for: Enhancement of disease resistance, growth potential, and photosynthesis in tomato (Solanum lycopersicum) by inoculation with an endophytic actinobacterium, Streptomyces thermocarboxydus strain BPSAC147
Source: PLoS One. 2019 Jul 3;14(7):e0219014. doi: 10.1371/journal.pone.0219014 (PMC6608948; doi:10.1371/journal.pone.0219014)

TAMILNADU AGRICULTURAL UNIVERSITY - AGRICULTURAL MICROBIOLOGY

INSTRUMENT: PERKIN ELMER CLARUS SQ8C COLOUMN: DB-5 MS CAPILARY STANDARD NON - POLARCOLOUMN
INJECTION VOL: 1 MICRO LITER DIMENSION: 30Mts, ID: 0.25 mm, FILM: 0.25 IM CARRIER GAS: He
SAMPLE ID : T260

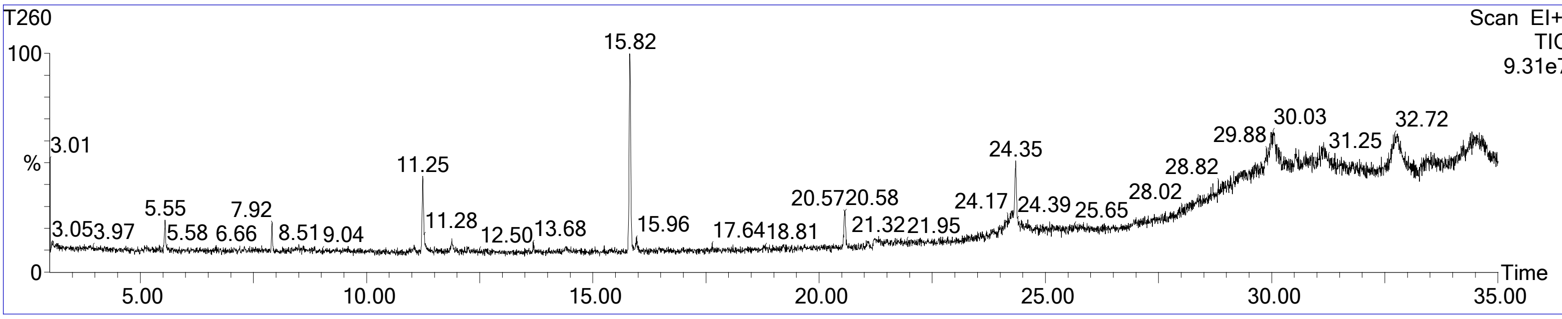

#	RT	Scan	Height	Area	Area %	Norm %
1	11.247	1649	32,632,418	1,480,199.5	1.268	40.13

Pk #	RT	Hit	Compound Name	Match	R.Match	Prob.	CAS	Library
1	11.247	1	1-Undecanol	796	919	9.4	112-42-5	mainlib
		2	1-Undecanol	778	904	9.4	112-42-5	replib
		3	1-Dodecanol	777	869	4.6	112-53-8	replib
		4	Cyclodecane	769	882	3.4	293-96-9	replib
		5	1-Dodecanol	768	870	4.6	112-53-8	replib
		6	1-Dodecanol	768	848	4.6	112-53-8	replib
		7	1-Hexadecanol	767	860	3.1	36653-82-4	mainlib
		8	1-Tetradecanol	765	815	2.9	112-72-1	replib
		9	1-Undecanol	763	873	9.4	112-42-5	replib
		10	1-Dodecanol	762	859	4.6	112-53-8	replib

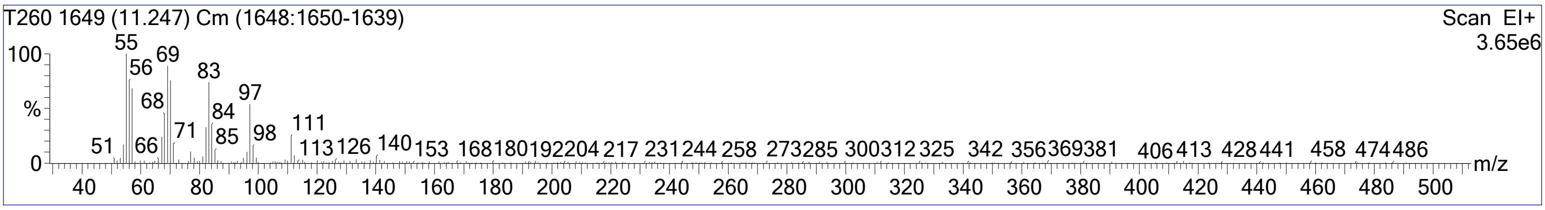

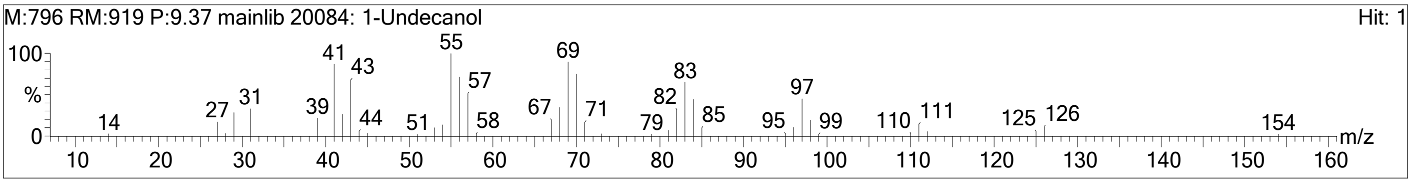

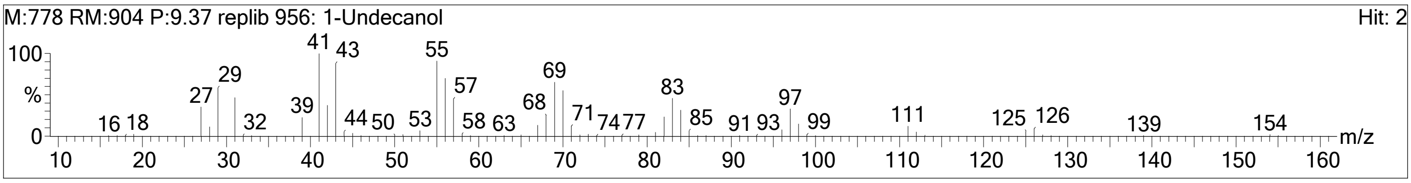

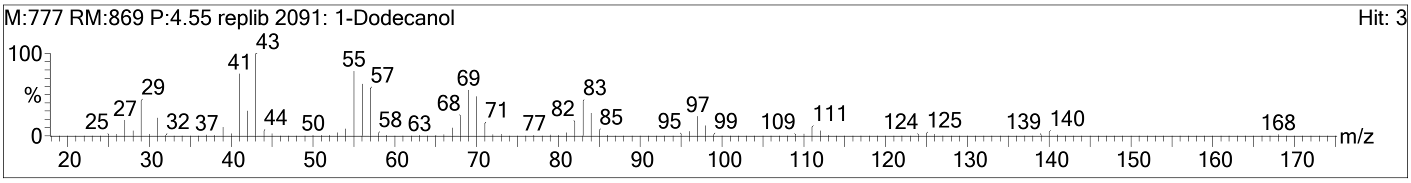

TAMILNADU AGRICULTURAL UNIVERSITY - AGRICULTURAL MICROBIOLOGY

INSTRUMENT: PERKIN ELMER CLARUS SQ8C COLOUMN: DB-5 MS CAPILARY STANDARD NON - POLARCOLOUMN
INJECTION VOL: 1 MICRO LITER DIMENSION: 30Mts, ID: 0.25 mm, FILM: 0.25 IM CARRIER GAS: He
SAMPLE ID : T260

#	RT	Scan	Height	Area	Area %	Norm %
2	15.818	2563	85,449,568	3,663,354.2	3.139	99.31

Pk #	RT	Hit	Compound Name	Match	R.Match	Prob.	CAS	Library
2	15.818	1	Dodecyl acrylate	876	912	48.0	2156-97-0	replib
		2	Dodecyl acrylate	875	901	48.0	2156-97-0	replib
		3	Dodecyl acrylate	831	860	48.0	2156-97-0	mainlib
		4	2-Propenoic acid, tridecyl ester	827	861	9.9	3076-04-8	mainlib
		5	2-Propenoic acid, pentadecyl ester	795	819	2.6	43080-23-5	mainlib
		6	1-Dodecanol	784	841	1.8	112-53-8	replib
		7	Ethylene diacrylate	778	820	1.4	2274-11-5	mainlib
		8	4-Trifluoroacetoxytridecane	776	813	1.3		mainlib
		9	Dichloroacetic acid, dodecyl ester	774	805	1.2	83005-01-0	mainlib
		10	5-Eicosene, (E)-	772	830	1.1	74685-30-6	mainlib

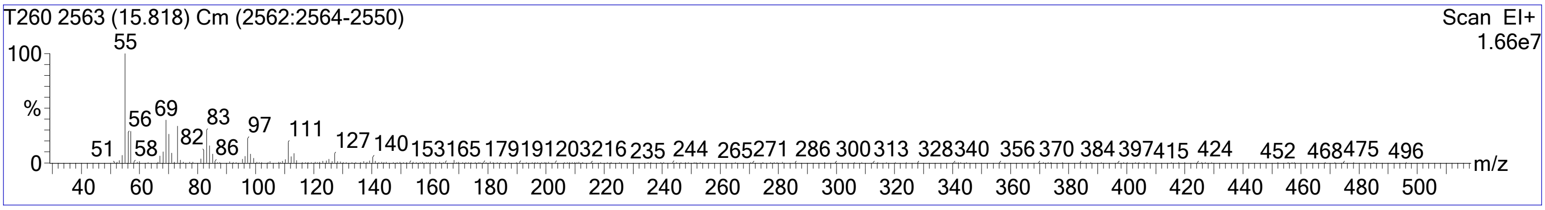

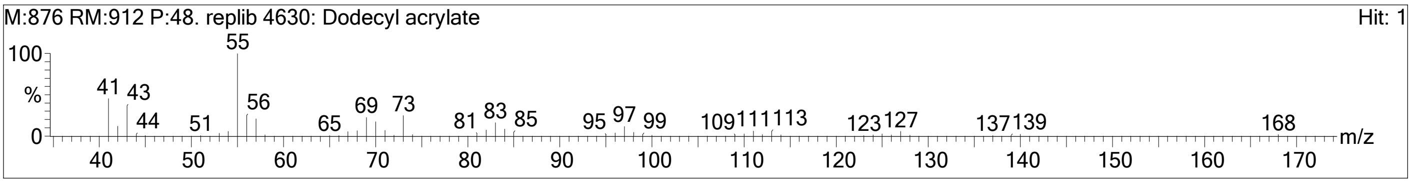

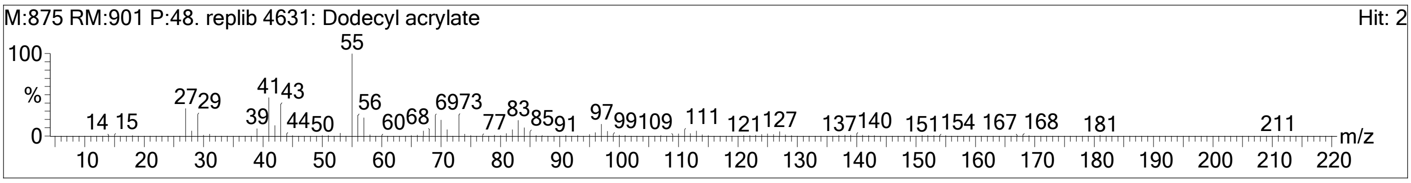

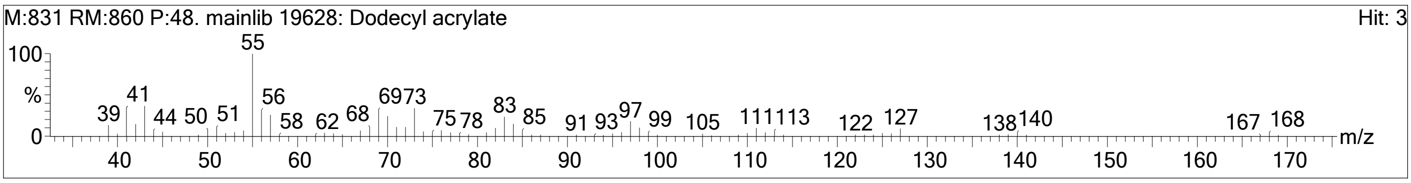

TAMILNADU AGRICULTURAL UNIVERSITY - AGRICULTURAL MICROBIOLOGY

INSTRUMENT: PERKIN ELMER CLARUS SQ8C　　COLOUMN: DB-5 MS CAPILARY STANDARD NON - POLARCOLOUMN
INJECTION VOL: 1 MICRO LITER　　DIMENSION: 30Mts, ID: 0.25 mm, FILM: 0.25 IM　　CARRIER GAS: He
SAMPLE ID : T260

#	RT	Scan	Height	Area	Area %	Norm %
3	20.575	3514	17,308,590	928,657.2	0.796	25.17

Pk #	RT	Hit	Compound Name	Match	R.Match	Prob.	CAS	Library
3	20.575	1	Hexadecanoic acid, methyl ester	616	728	15.9	112-39-0	mainlib
		2	Hexadecanoic acid, methyl ester	606	809	15.9	112-39-0	replib
		3	Pentadecanoic acid, 14-methyl-, methyl ester	599	765	8.7	5129-60-2	mainlib
		4	Pentadecanoic acid, 13-methyl-, methyl ester	596	655	7.7	5487-50-3	mainlib
		5	Hexadecanoic acid, methyl ester	594	844	15.9	112-39-0	replib
		6	Hexadecanoic acid, 14-methyl-, methyl ester	593	640	6.8	2490-49-5	mainlib
		7	Methyl tetradecanoate	591	765	6.2	124-10-7	replib
		8	Hexadecanoic acid, methyl ester	591	720	15.9	112-39-0	replib
		9	Hexadecanoic acid, methyl ester	588	690	15.9	112-39-0	replib
		10	Tetradecanoic acid, 12-methyl-, methyl ester	587	670	5.3	5129-66-8	mainlib

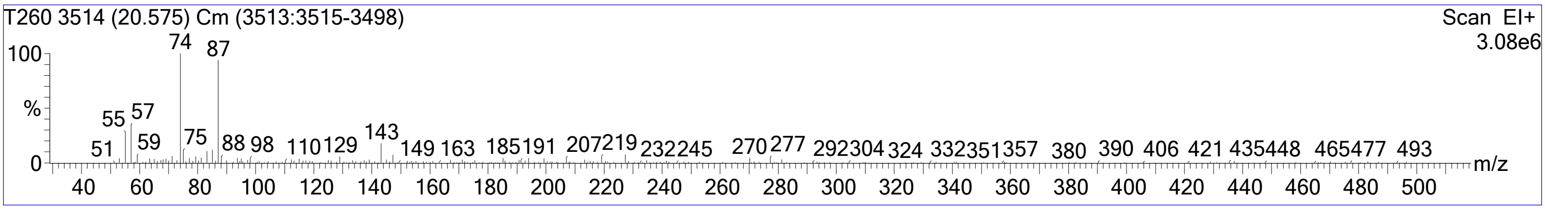

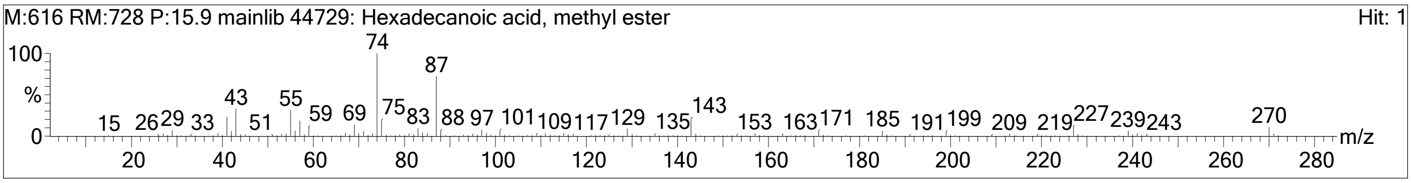

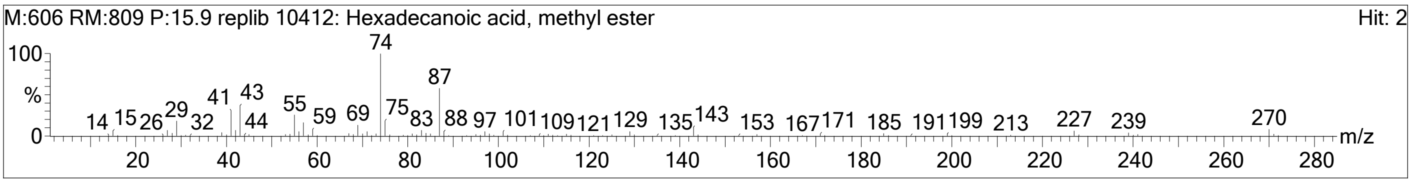

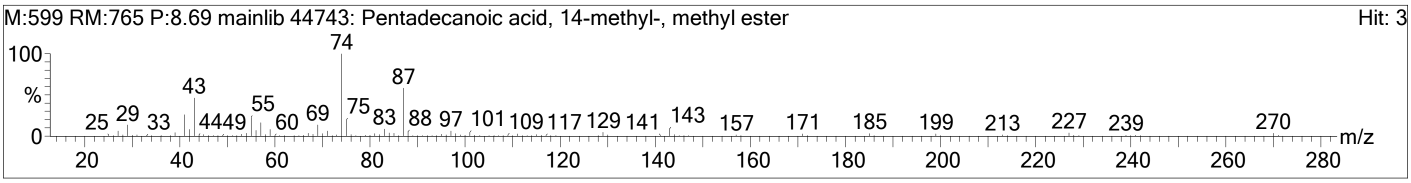

TAMILNADU AGRICULTURAL UNIVERSITY - AGRICULTURAL MICROBIOLOGY

INSTRUMENT: PERKIN ELMER CLARUS SQ8C COLOUMN: DB-5 MS CAPILARY STANDARD NON - POLARCOLOUMN
INJECTION VOL: 1 MICRO LITER DIMENSION: 30Mts, ID: 0.25 mm, FILM: 0.25 IM CARRIER GAS: He
SAMPLE ID : T260

#	RT	Scan	Height	Area	Area %	Norm %
4	24.257	4250	11,163,678	726,894.6	0.623	19.71

Pk #	RT	Hit	Compound Name	Match	R.Match	Prob.	CAS	Library
4	24.257	1	Ethyl iso-allocholate	464	486	9.1		mainlib
		2	1-Heptatriacotanol	456	515	6.8	105794-58-9	mainlib
		3	1-Hexadecanol, 2-methyl-	442	515	4.2	2490-48-4	mainlib
		4	Morphinan-4,5-epoxy-3,6-di-ol, 6-[7-nitrobenzofurazan-4-yl]amino-	440	488	3.9		mainlib
		5	Hexadecane, 1,1-bis(dodecyloxy)-	438	485	3.6	56554-64-4	mainlib
		6	Cholest-22-ene-21-ol, 3,5-dehydro-6-methoxy-, pivalate	434	509	3.0		mainlib
		7	Stigmastane-3,6-dione, (5à)-	431	512	2.7	22149-69-5	mainlib
		8	Glycidyl oleate	427	487	2.3		mainlib
		9	Rhodopin	424	426	2.0	105-92-0	mainlib
		10	7-Heptadecene, 17-chloro-	423	505	1.9	56554-79-1	mainlib

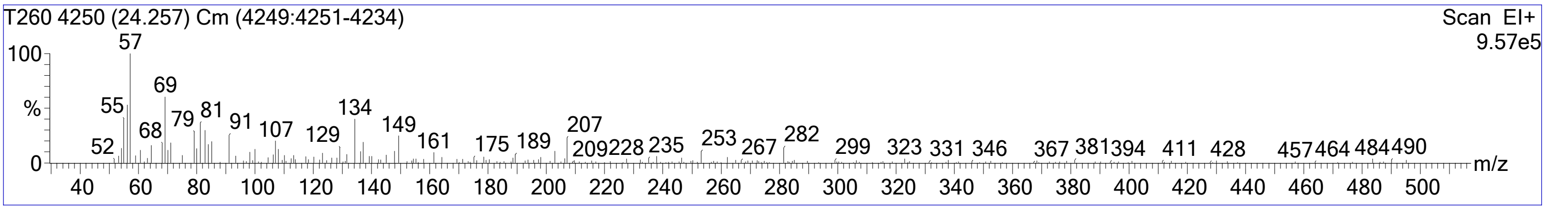

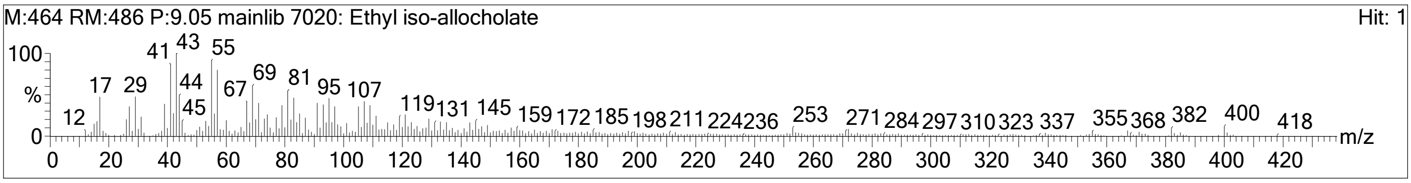

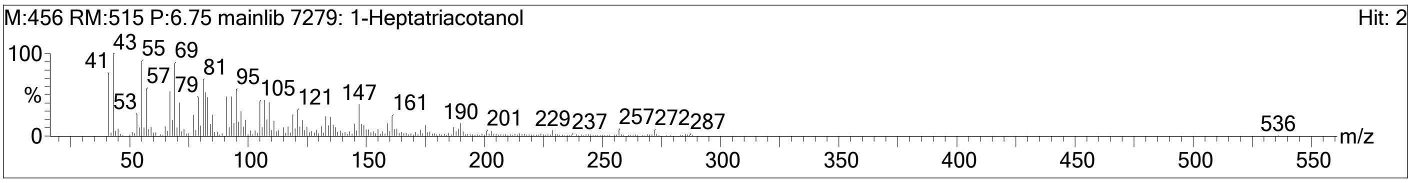

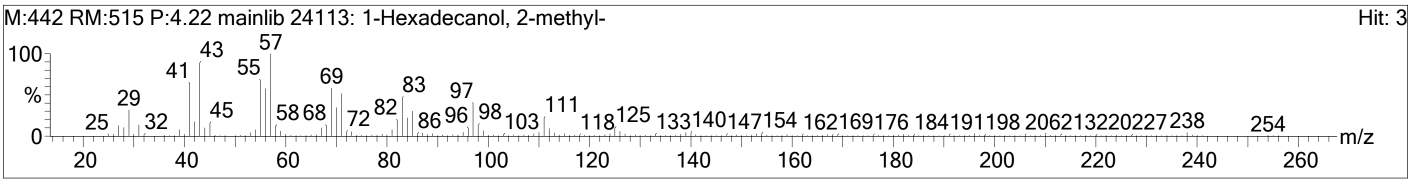

TAMILNADU AGRICULTURAL UNIVERSITY - AGRICULTURAL MICROBIOLOGY

INSTRUMENT: PERKIN ELMER CLARUS SQ8C	COLOUMN: DB-5 MS CAPILARY STANDARD NON - POLARCOLOUMN
INJECTION VOL: 1 MICRO LITER	DIMENSION: 30Mts, ID: 0.25 mm, FILM: 0.25 IM	CARRIER GAS: He
SAMPLE ID : T260

#	RT	Scan	Height	Area	Area %	Norm %
5	24.347	4268	32,221,488	1,923,259.0	1.648	52.14

Pk #	RT	Hit	Compound Name	Match	R.Match	Prob.	CAS	Library
5	24.347	1	Methyl stearate	675	771	54.6	112-61-8	replib
		2	Methyl stearate	664	774	54.6	112-61-8	replib
		3	Methyl stearate	662	765	54.6	112-61-8	replib
		4	Methyl stearate	659	820	54.6	112-61-8	replib
		5	Methyl stearate	659	750	54.6	112-61-8	mainlib
		6	Methyl stearate	658	789	54.6	112-61-8	replib
		7	Heptadecanoic acid, 16-methyl-, methyl ester	649	722	16.4	5129-61-3	mainlib
		8	Heptadecanoic acid, 16-methyl-, methyl ester	643	721	16.4	5129-61-3	replib
		9	Heptadecanoic acid, 16-methyl-, methyl ester	637	757	16.4	5129-61-3	replib
		10	Methyl stearate	629	723	54.6	112-61-8	replib

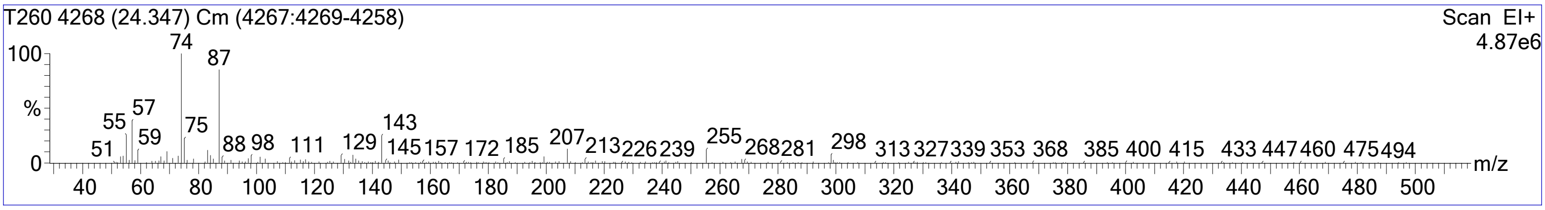

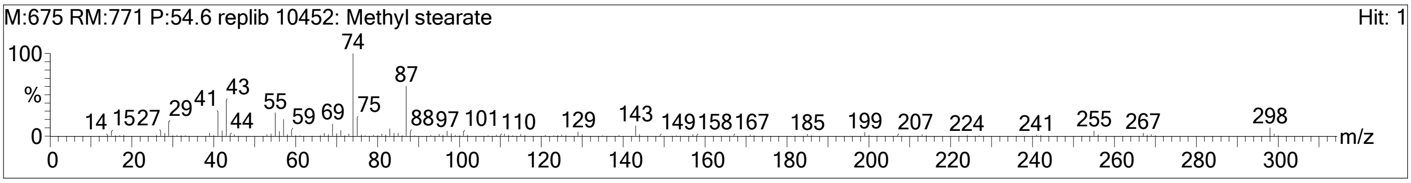

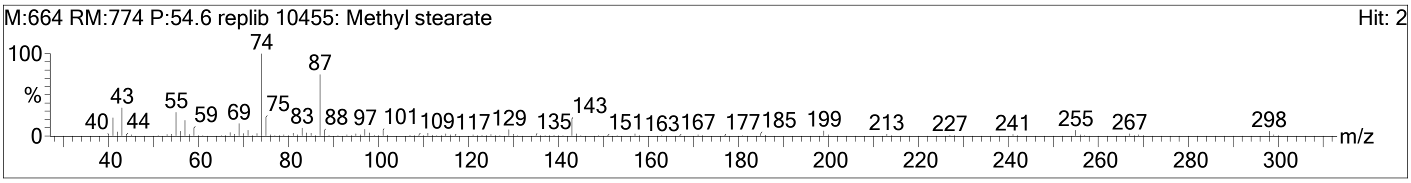

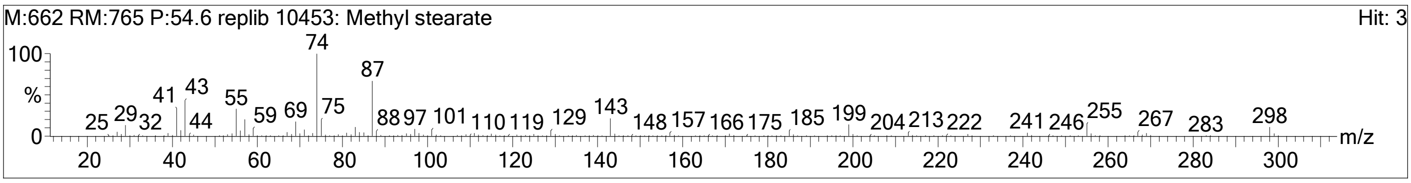

TAMILNADU AGRICULTURAL UNIVERSITY - AGRICULTURAL MICROBIOLOGY

INSTRUMENT: PERKIN ELMER CLARUS SQ8C COLOUMN: DB-5 MS CAPILARY STANDARD NON - POLARCOLOUMN
INJECTION VOL: 1 MICRO LITER DIMENSION: 30Mts, ID: 0.25 mm, FILM: 0.25 IM CARRIER GAS: He
SAMPLE ID : T260

#	RT	Scan	Height	Area	Area %	Norm %
6	29.419	5282	12,617,741	953,241.6	0.817	25.84

Pk #	RT	Hit	Compound Name	Match	R.Match	Prob.	CAS	Library
6	29.419	1	5Z,?8Z,?11Z,?14Z-Eeicosatetraenoic acid,? 3-?theinylmethyl ester	403	497	17.7	390824-17-6	mainlib
		2	Eicosapentaenoic Acid, TBDMS derivative	372	412	4.8		mainlib
		3	Doconexent, TBDMS derivative	369	413	4.3		mainlib
		4	7,10,13,16-Docosatetraenoic acid, (Z)-, TMS derivative	368	415	4.1		mainlib
		5	Arachidonic Acid, TBDMS derivative	364	412	3.5		mainlib
		6	Doconexent, TMS derivative	361	411	3.1		mainlib
		7	2,4a-Oxymethano-1,2,3,4,4a,4b,5,6,7,8,8a,9-dodecahydrophenanthren-9-one, 8-cyanomethyl-2-methoxy-7-methoxycarbonyl-1,1,7-trimethyl-	359	380	2.8		mainlib
		8	Eicosapentaenoic Acid, TMS derivative	354	405	2.3		mainlib
		9	17-(1,5-Dimethylhexyl)-2,3-dihydroxy-10,13-dimethyl-1,2,3,7,8,9,10,11,12,13,14,15,16,17-tetradecahydrocyclopenta[a]phenanthren-6-one	353	369	2.2		mainlib
		10	5-Hydroxymethyl-4-pentanoyl-1-(1-phenylethyl)imidazolidin-2-one	352	491	2.1		mainlib

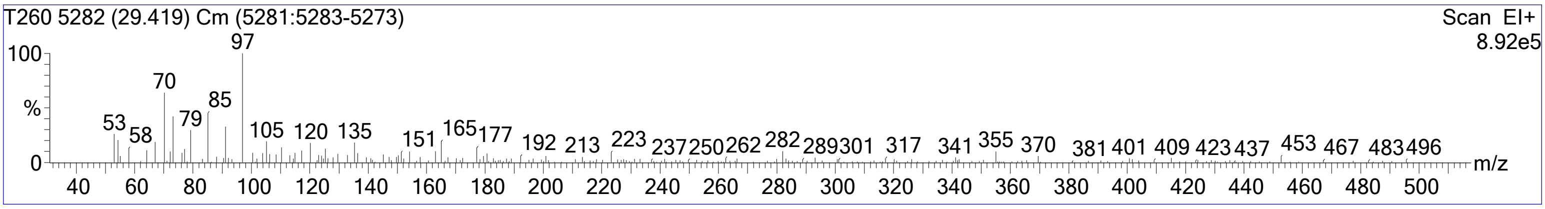

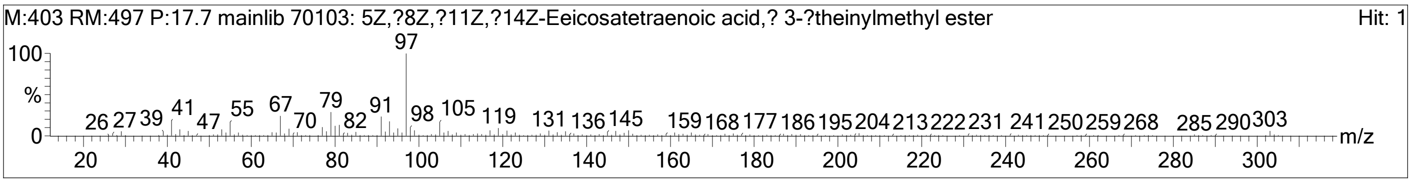

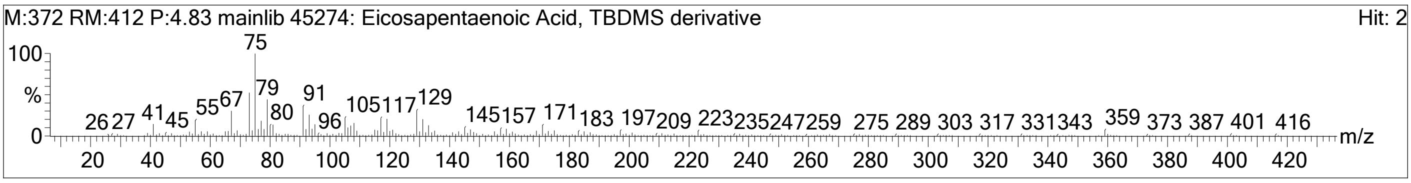

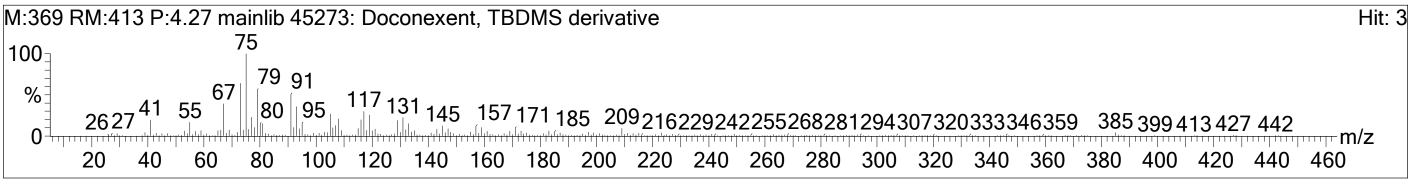

TAMILNADU AGRICULTURAL UNIVERSITY - AGRICULTURAL MICROBIOLOGY

INSTRUMENT: PERKIN ELMER CLARUS SQ8C COLOUMN: DB-5 MS CAPILARY STANDARD NON - POLARCOLOUMN
INJECTION VOL: 1 MICRO LITER DIMENSION: 30Mts, ID: 0.25 mm, FILM: 0.25 IM CARRIER GAS: He
SAMPLE ID : T260

#	RT	Scan	Height	Area	Area %	Norm %
7	29.639	5326	15,488,421	697,858.4	0.598	18.92

Pk #	RT	Hit	Compound Name	Match	R.Match	Prob.	CAS	Library
7	29.639	1	Strychane, 1-acetyl-20à-hydroxy-16-methylene-	496	551	28.2	2111-98-0	mainlib
		2	Rhodopin	444	446	5.6	105-92-0	mainlib
		3	Glafenin	439	731	4.5	3820-67-5	nist_msms
		4	7,8-Epoxylanostan-11-ol, 3-acetoxy-	431	435	3.4		mainlib
		5	12-Hydroxyoctadecanethioic acid, S-t-butyl ester	428	482	3.0	58587-08-9	mainlib
		6	1-Heptatriacotanol	425	504	2.6	105794-58-9	mainlib
		7	1-Phenazinecarboxylic acid, 6-(1-methoxyethyl)-, methyl ester	421	492	2.2	73634-71-6	mainlib
		8	2,6-Dihydroxyacetophenone, 2TMS derivative	421	483	2.2		mainlib
		9	2,5-Dihydroxyacetophenone, 2TMS derivative	418	476	2.0		mainlib
		10	Benzofuran-6-ol-3-one, 2-(4-ethoxycarbonyl)benzylidene-	415	503	1.7		mainlib

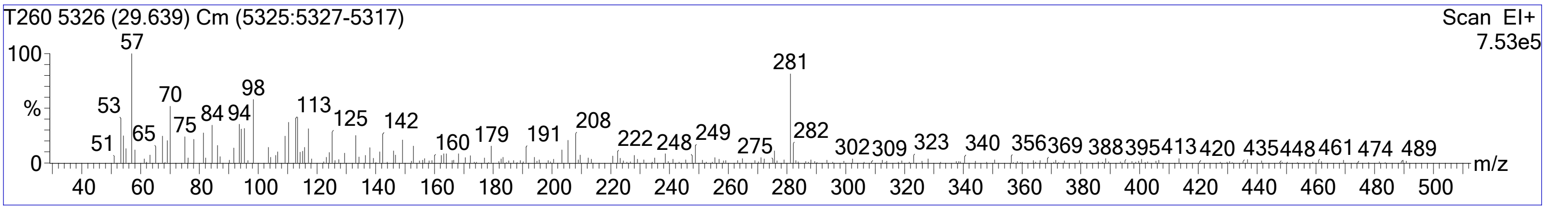

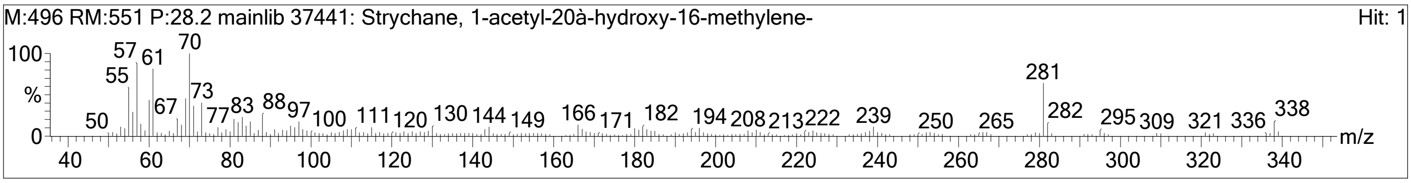

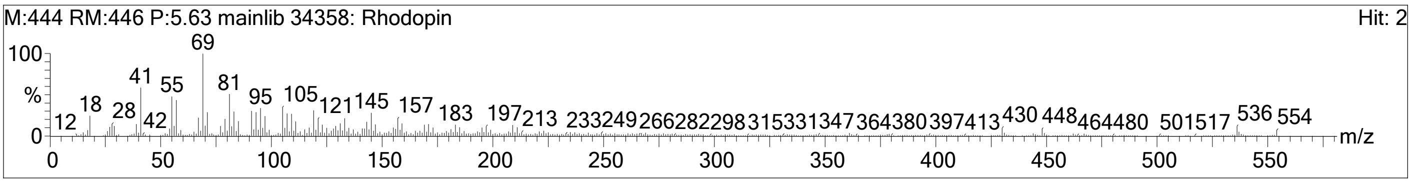

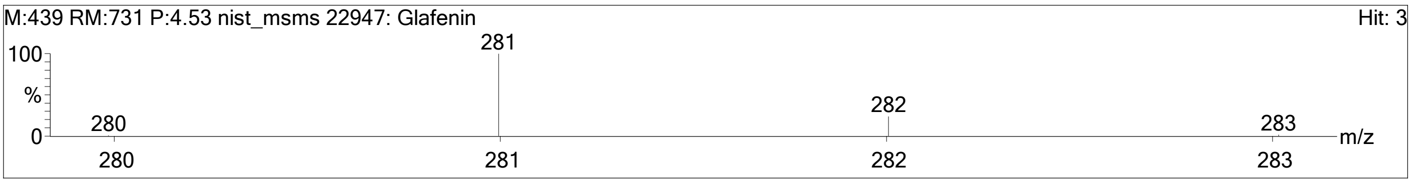

TAMILNADU AGRICULTURAL UNIVERSITY - AGRICULTURAL MICROBIOLOGY

INSTRUMENT: PERKIN ELMER CLARUS SQ8C
COLOUMN: DB-5 MS CAPILARY STANDARD NON - POLARCOLOUMN
INJECTION VOL: 1 MICRO LITER
DIMENSION: 30Mts, ID: 0.25 mm, FILM: 0.25 IM
CARRIER GAS: He
SAMPLE ID : T260

#	RT	Scan	Height	Area	Area %	Norm %
8	29.689	5336	14,803,180	787,371.8	0.675	21.34

Pk #	RT	Hit	Compound Name	Match	R.Match	Prob.	CAS	Library
8	29.689	1	Oleic acid, 3-(octadecyloxy)propyl ester	407	455	10.0	17367-41-8	mainlib
		2	1,2-Dihydroprednisolone, 21-O-acetyl-11-thiocaynato-	401	463	7.9	50733-59-0	mainlib
		3	9-Methylcorticosterone acetate	397	461	6.7		mainlib
		4	Spirost-8-en-11-one, 3-hydroxy-, (3á,5à,14á,20á,22á,25R)-	391	450	5.2	58072-54-1	mainlib
		5	Pregnan-20-one, 3-(acetyloxy)-5,6-epoxy-, (3á,5á,6á)-	388	438	4.6	6661-94-5	mainlib
		6	17Beta-acetoxy-17alpha-ethynyl-6beta-methoxy-3alpha,5-cyclo-5alpha-androstan-19-oic acid	384	422	3.9		mainlib
		7	1,9-Dioxa-5-thianonane, 3,7-bis(9-borabicyclo[3.3.1]non-9-yloxy)-1,9-diphenyl-	381	403	3.5		mainlib
		8	1h-Pyrrole-3,4-diacetic acid, 2-acetoxymethyl-5-methoxycarbonyl-, dimethyl ester	379	434	3.2		mainlib
		9	4-Acetyloxyimino-6,6-dimethyl-3-methylsulfanyl-4,5,6,7-tetrahydro-benzo[c]thiophene-1-carboxylic acid methyl ester	375	435	2.7		mainlib
		10	9à-Fluoro-11á,17á-diol-17à-methyl-5à-androstan-3-one	370	441	2.2		mainlib

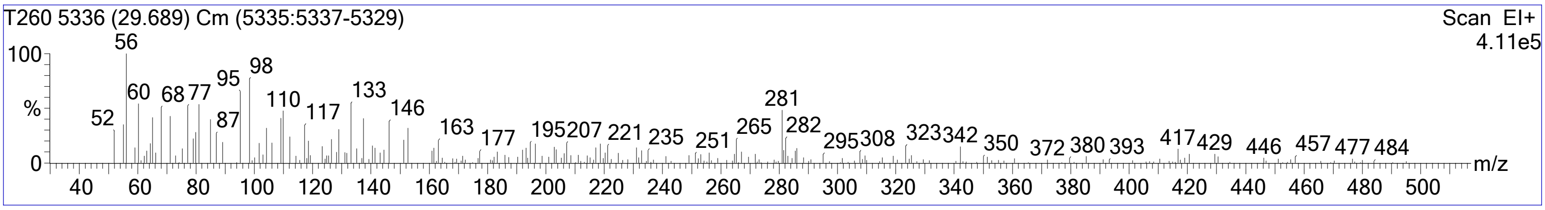

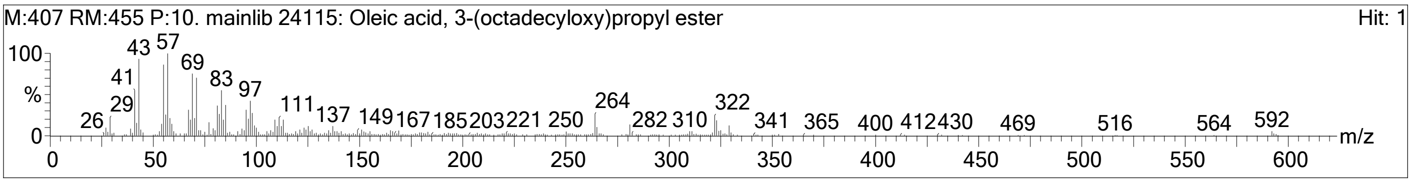

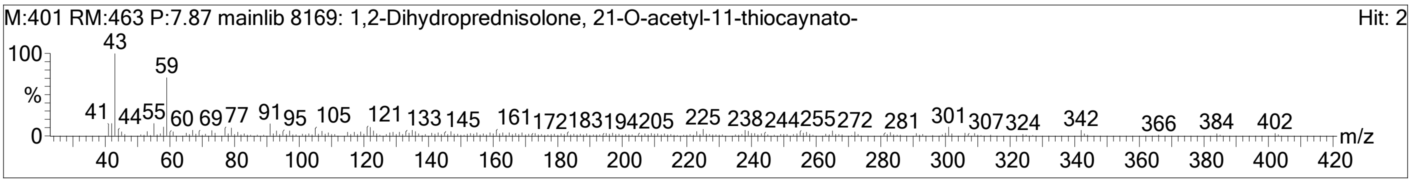

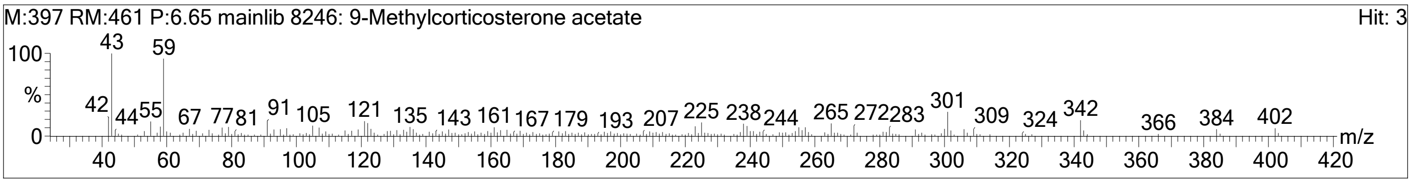

TAMILNADU AGRICULTURAL UNIVERSITY - AGRICULTURAL MICROBIOLOGY

INSTRUMENT: PERKIN ELMER CLARUS SQ8C COLOUMN: DB-5 MS CAPILARY STANDARD NON - POLARCOLOUMN
INJECTION VOL: 1 MICRO LITER DIMENSION: 30Mts, ID: 0.25 mm, FILM: 0.25 IM CARRIER GAS: He
SAMPLE ID : T260

#	RT	Scan	Height	Area	Area %	Norm %
9	29.764	5351	15,481,806	1,236,610.0	1.060	33.52

Pk #	RT	Hit	Compound Name	Match	R.Match	Prob.	CAS	Library
9	29.764	1	7aH-Cyclopenta[a]cyclopropa[f]cycloundecene-2, 4,7,7a,10,11-hexol, 1,1a,2,3,4,4a,5,6,7,10,11,11a-dodecahydro-1,1,3,6,9-pentamethyl-, 2,4,7,10,11-pentaacetate	391	414	5.8	51906-08-2	mainlib
		2	4-Acetyloxyimino-6,6-dimethyl-3-methylsulfanyl-4, 5,6,7-tetrahydro-benzo[c]thiophene-1-carboxylic acid methyl ester	389	444	5.3		mainlib
		3	3-Isopropyl-6a,7,10b-trimethyl-dodecahydro-benzo[f]chromene-7,8-dicarboxylic acid, dimethyl ester	389	426	5.3		mainlib
		4	5H-Cyclopropa[3,4]benz[1,2-e]azulen-5-one, 9,9a-bis(acetyloxy)-3-[(acetyloxy)methyl]-1,1a,1b,4,4a, 7a,7b,8,9,9a-decahydro-4,4a,7b-trihydroxy-1,1,6, 8-tetramethyl-	380	401	3.8	77646-80-1	mainlib
		5	8H-Pyrano[3,4-b]pyrimido[5,4-d]furane, 5,6-dihydro-4-hydrazino-6,6-dimethyl-2-methylthio-	377	448	3.4	253146-95-1	mainlib
		6	3-[3-(1,5-Dimethylhexyl)-7-(2-hydroxy-1-methylethyl)-3a,6,9b-trimethyl-2,3,3a,4,5,6,7,8,9, 9b-decahydro-1H-cyclopenta[a]naphthalen-6-yl]propanoic acid, methyl ester	375	391	3.1		mainlib
		7	3,9-Ethyleno-1,4,6,8,11,13-hexaazatetracyclo[9.3.0.0(3,10).0(4,8)]tetradeca-5,7,12,14-tetraone, 2,6,13,trimethyl-2-phenyl-	373	406	2.9		mainlib
		8	Octadecane, 1,1'-[1,3-propanediylbis(oxy)]bis-	371	387	2.7	17367-38-3	mainlib
		9	5H-Cyclopropa(3,4)benz(1,2-e)azulen-5-one, 1,1a-à,1b-á,4,4a,7a-à,7b,8,9,9a-decahydro-4a-á,7b-à, 9a-à-trihydroxy-3-(hydroxymethyl)-1,1,6,8-à-tetramethyl-, 9a-isobutyrate	367	388	2.2	25090-74-8	mainlib
		10	25-Norisopropyl-9,19-cyclolanostan-22-en-24-one, 3-acetoxy-24-phenyl-4,4,14-trimethyl-	366	374	2.2		mainlib

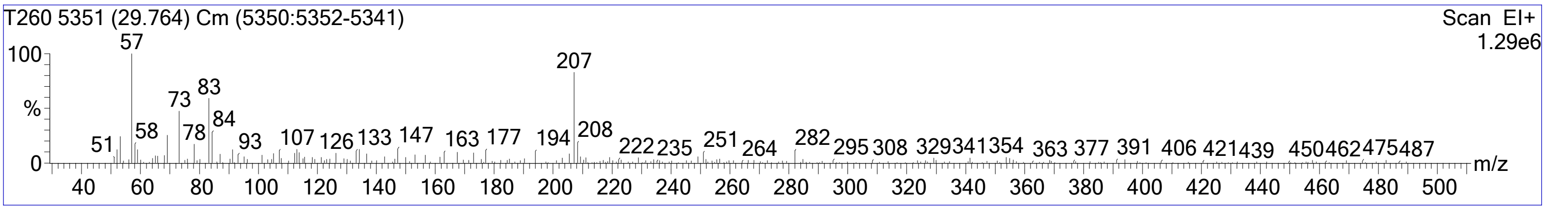

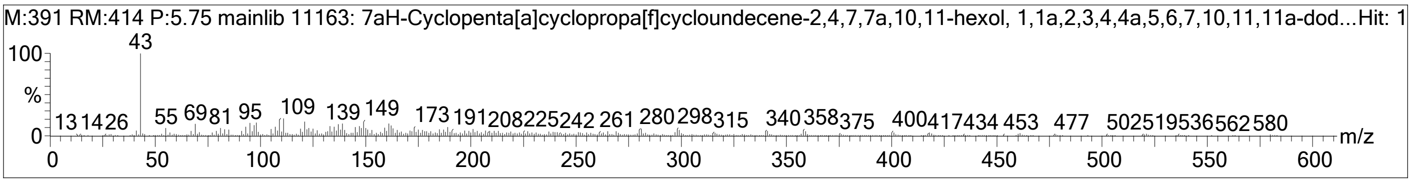

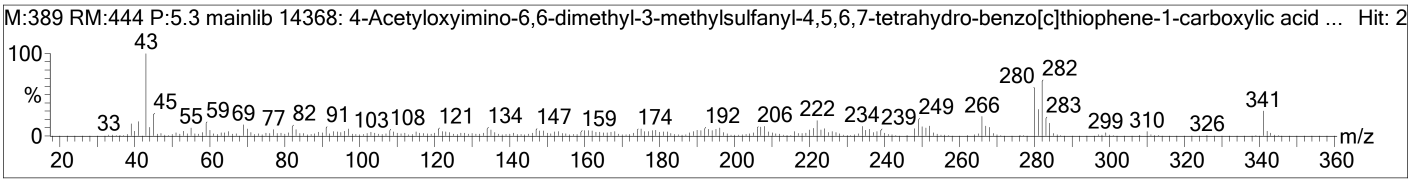

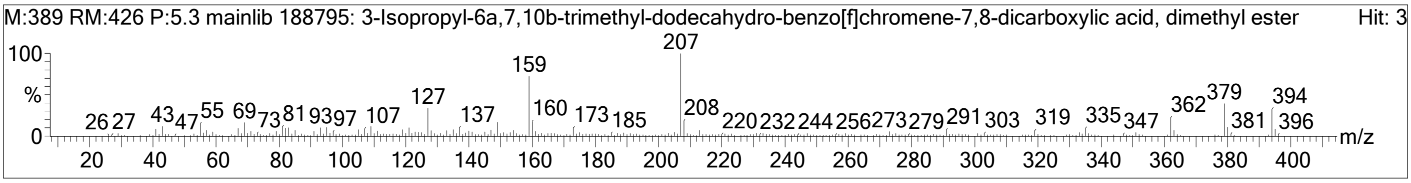

TAMILNADU AGRICULTURAL UNIVERSITY - AGRICULTURAL MICROBIOLOGY

INSTRUMENT: PERKIN ELMER CLARUS SQ8C COLOUMN: DB-5 MS CAPILARY STANDARD NON - POLARCOLOUMN
INJECTION VOL: 1 MICRO LITER DIMENSION: 30Mts, ID: 0.25 mm, FILM: 0.25 IM CARRIER GAS: He
SAMPLE ID : T260

#	RT	Scan	Height	Area	Area %	Norm %
10	29.884	5375	21,090,470	1,435,067.6	1.230	38.90

Pk #	RT	Hit	Compound Name	Match	R.Match	Prob.	CAS	Library
10	29.884	1	1-(2-Acetoxyethyl)-3,6-diazahomoadamantan-9-one oxime	413	503	12.9		mainlib
		2	Glycine, N-[(3à,5á)-24-oxo-3-[(trimethylsilyl)oxy]cholan-24-yl]-, methyl ester	399	456	8.1	57326-15-5	mainlib
		3	1,8-Dioxa-5-thiaoctane, 8-(9-borabicyclo[3.3.1]non-9-yl)-3-(9-borabicyclo[3.3.1]non-9-yloxy)-1-phenyl-	387	425	5.4		mainlib
		4	N-[3,5-Dinitropyridin-2-yl]glutamic acid	386	455	5.2		mainlib
		5	Phenol, 2,6-dichloro-4-nitro-	383	489	4.6	618-80-4	mainlib
		6	Acetic acid, 2,3-dibromo-4-methoxymethoxy-1-methyl-pent-2-enyl ester	379	484	3.9		mainlib
		7	Octyltrichlorosilane	378	515	3.7	5283-66-9	replib
		8	4,7,7-Trimethyl-3-oxo-2-oxabicyclo[2.2.1]heptane-1-carboxylic acid, 2,4,4-trimethyl-3-(3-oxo-but-1-enyl)-cyclohex-2-enyl ester	377	463	3.6		mainlib
		9	1-Bromo-11-iodoundecane	376	480	3.4	139123-69-6	mainlib
		10	3,6,9,12-Tetraoxatetradecan-1-ol, 14-[4-(1,1,3,3-tetramethylbutyl)phenoxy]-	375	457	3.3	2315-64-2	mainlib

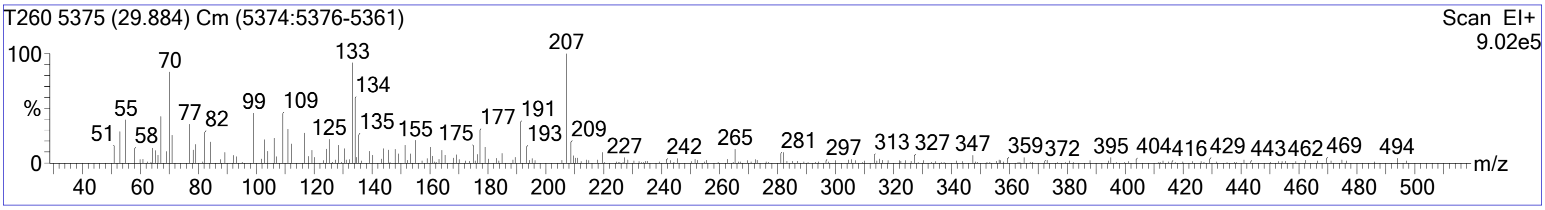

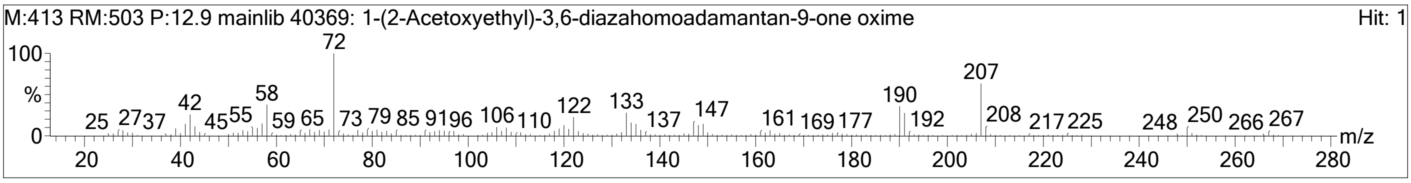

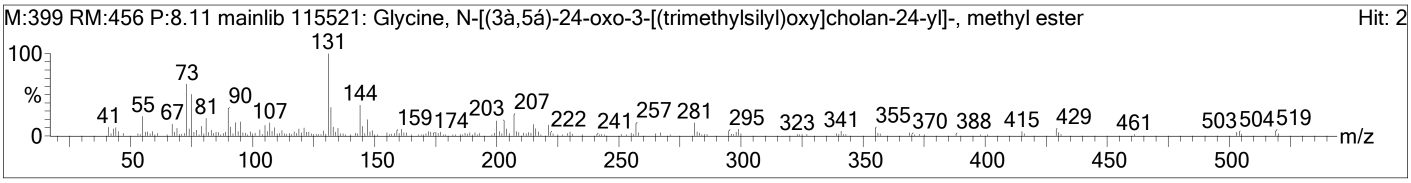

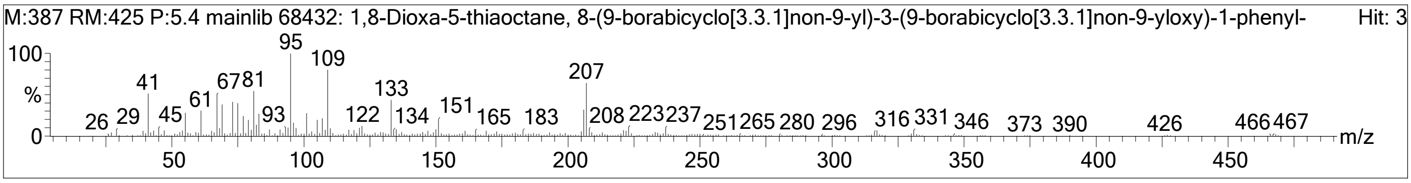

TAMILNADU AGRICULTURAL UNIVERSITY - AGRICULTURAL MICROBIOLOGY

INSTRUMENT: PERKIN ELMER CLARUS SQ8C COLOUMN: DB-5 MS CAPILARY STANDARD NON - POLARCOLOUMN
INJECTION VOL: 1 MICRO LITER DIMENSION: 30Mts, ID: 0.25 mm, FILM: 0.25 IM CARRIER GAS: He
SAMPLE ID : T260

#	RT	Scan	Height	Area	Area %	Norm %
11	30.034	5405	28,574,880	3,688,861.0	3.161	100.00

Pk #	RT	Hit	Compound Name	Match	R.Match	Prob.	CAS	Library
11	30.034	1	2,4,6-Decatrienoic acid, 1a,2,5,5a,6,9,10,10a-octahydro-5,5a-dihydroxy-4-(hydroxymethyl)-1,7,9-trimethyl-1-[[(2-methyl-1-oxo-2-butenyl)oxy]methyl]-11-oxo-1H-2,8a-methanocyclopenta[a]cyclopropa[e]cyclodecen-6-yl ester	457	457	18.3	77646-20-9	mainlib
		2	Corynan-17-ol, 18,19-didehydro-10-methoxy-, acetate (ester)	447	495	12.9	56053-13-5	mainlib
		3	Bufa-20,22-dienolide, 14,15-epoxy-3,11-dihydroxy-, (3á,5á,11à,15á)-	442	468	10.4	39005-15-7	mainlib
		4	Aconitine	421	518	4.4	302-27-2	nist_msms
		5	Pyrano[4,3-b]benzopyran-1,9-dione, 5a-methoxy-9a-methyl-3-(1-propenyl)perhydro-	420	457	4.3		mainlib
		6	Cholestan-3-ol, 2-methylene-, (3á,5à)-	409	537	2.9	22599-96-8	mainlib
		7	Fumaric acid, 10-chlorodecyl octyl ester	407	474	2.7		mainlib
		8	1,2,3,4-Tetrahydroisoquinolin, 2-acetyl-6,7-dimethoxy-1-phenmethylene-	407	458	2.7		mainlib
		9	Doconexent	406	459	2.6	6217-54-5	mainlib
		10	Aconitine	403	456	4.4	302-27-2	nist_msms

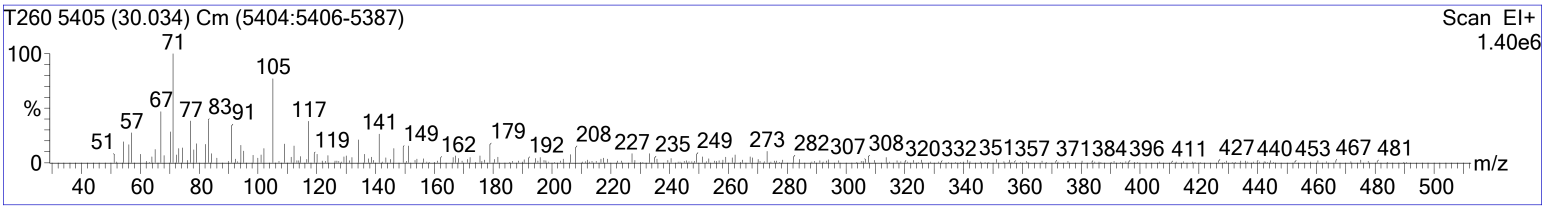

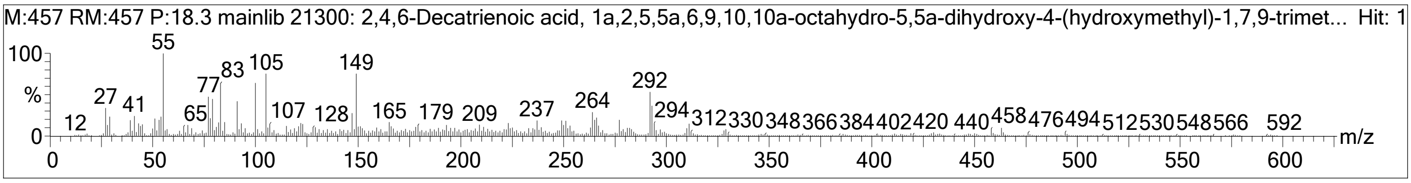

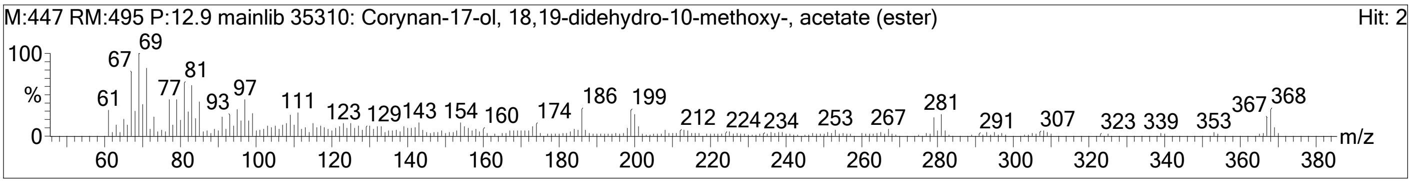

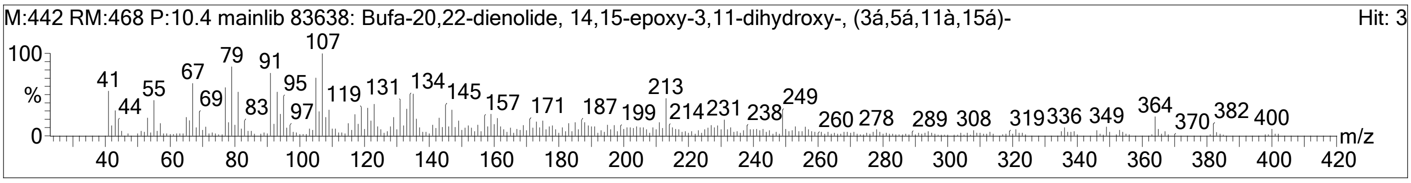

TAMILNADU AGRICULTURAL UNIVERSITY - AGRICULTURAL MICROBIOLOGY

INSTRUMENT: PERKIN ELMER CLARUS SQ8C COLOUMN: DB-5 MS CAPILARY STANDARD NON - POLARCOLOUMN
INJECTION VOL: 1 MICRO LITER DIMENSION: 30Mts, ID: 0.25 mm, FILM: 0.25 IM CARRIER GAS: He
SAMPLE ID : T260

#	RT	Scan	Height	Area	Area %	Norm %
12	30.149	5428	20,890,436	788,216.6	0.675	21.37

Pk #	RT	Hit	Compound Name	Match	R.Match	Prob.	CAS	Library
12	30.149	1	Tris(2-butoxyethyl) phosphate	423	720	8.1	78-51-3	nist_msms
		2	7,8-Epoxylanostan-11-ol, 3-acetoxy-	423	426	8.1		mainlib
		3	9,19-Cyclolanostan-3-ol, 24,24-epoxymethano-, acetate	418	430	6.5		mainlib
		4	Rhodopin	412	413	5.1	105-92-0	mainlib
		5	à-Hydroxybutyric acid	410	980	4.7	20016-85-7	nist_msms
		6	2-n-Propyl-1-heptanol	409	991	4.6	10042-59-8	nist_msms
		7	Perhydroindene-4-carboxylic acid, 6-acetoxy-2,3-epoxy-1,1-epoxymethyl-3a-hydroxy-5-isopropenyl-7a-methyl-7-oxo-, methyl ester	406	464	4.0		mainlib
		8	Cholestane, 3,5-dichloro-6-nitro-, (3á,5à,6á)-	403	408	3.5	15505-92-7	mainlib
		9	Valproic Acid	398	981	2.9	99-66-1	nist_msms
		10	Tris(2-butoxyethyl) phosphate	395	620	8.1	78-51-3	nist_msms

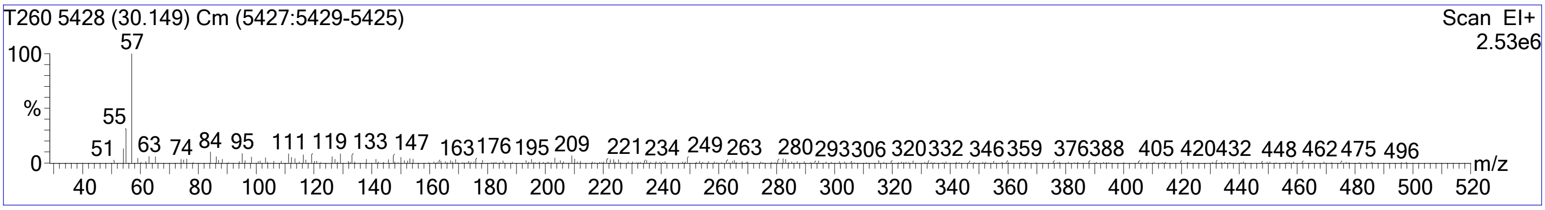

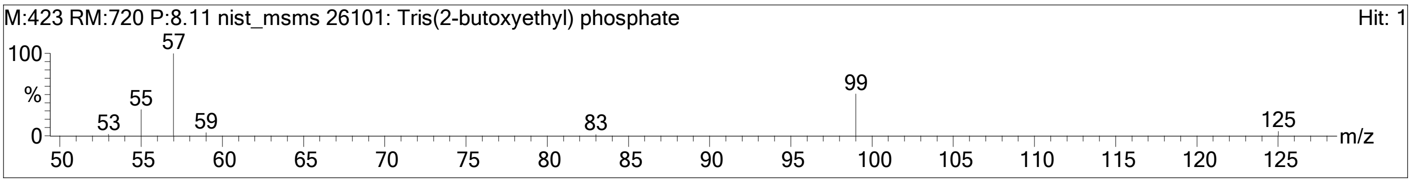

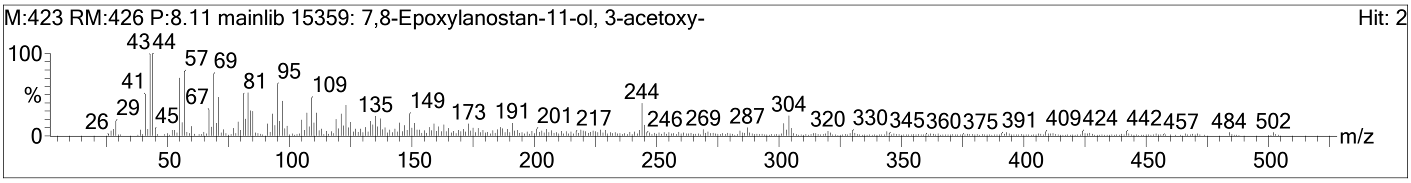

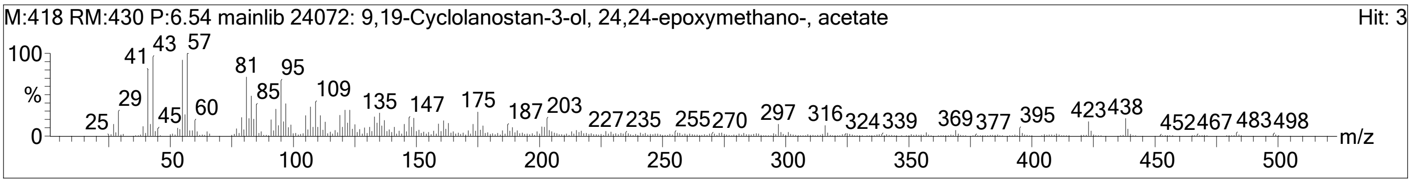

TAMILNADU AGRICULTURAL UNIVERSITY - AGRICULTURAL MICROBIOLOGY

INSTRUMENT: PERKIN ELMER CLARUS SQ8C   COLOUMN: DB-5 MS CAPILARY STANDARD NON - POLARCOLOUMN
INJECTION VOL: 1 MICRO LITER   DIMENSION: 30Mts, ID: 0.25 mm, FILM: 0.25 IM   CARRIER GAS: He
SAMPLE ID : T260

#	RT	Scan	Height	Area	Area %	Norm %
13	30.204	5439	18,941,208	809,714.3	0.694	21.95

Pk #	RT	Hit	Compound Name	Match	R.Match	Prob.	CAS	Library
13	30.204	1	10-Acetoxy-2-hydroxy-1,2,6a,6b,9,9,12a-heptamethyl-1,3,4,5,6,6a,6b,7,8,8a,9,10,11,12,12a,12b,13,14b-octadecahydro-2H-picene-4a-carboxylic acid, methyl ester	450	472	9.7	14356-56-0	mainlib
		2	Octadecane, 3-ethyl-5-(2-ethylbutyl)-	449	477	9.3	55282-12-7	mainlib
		3	Docosanoic acid, 1,2,3-propanetriyl ester	429	448	4.2	18641-57-1	mainlib
		4	Dodecanoic acid, 1a,2,5,5a,6,9,10,10a-octahydro-5a-hydroxy-4-(hydroxymethyl)-1,1,7,9-tetramethyl-6,11-dioxo-1H-2,8a-methanocyclopenta[a]cyclopropa[e]cyclodecen-5-yl ester, [1aR-(1aà,2à,5á,5aá,8aà,9à,10aà)]-	427	427	3.9	77508-68-0	mainlib
		5	2-Myristynoyl pantetheine	420	487	3.0		mainlib
		6	Oleic acid, 3-(octadecyloxy)propyl ester	420	462	3.0	17367-41-8	mainlib
		7	Octadecane, 3-ethyl-5-(2-ethylbutyl)-	417	473	9.3	55282-12-7	replib
		8	2-Nonadecanone 2,4-dinitrophenylhydrazine	417	447	2.6	28813-61-8	mainlib
		9	6,19-Cycloandrostane-3,7-diol, 3á-methoxy-	416	486	2.5		mainlib
		10	1,25-Dihydroxyvitamin D3, TMS derivative	414	517	2.3	55759-94-9	mainlib

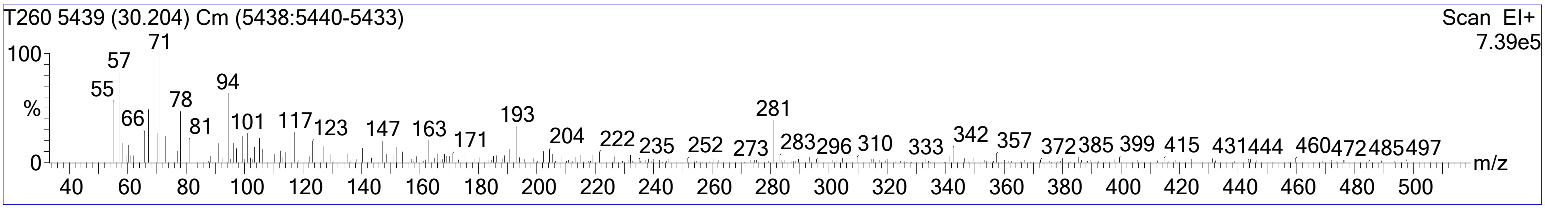

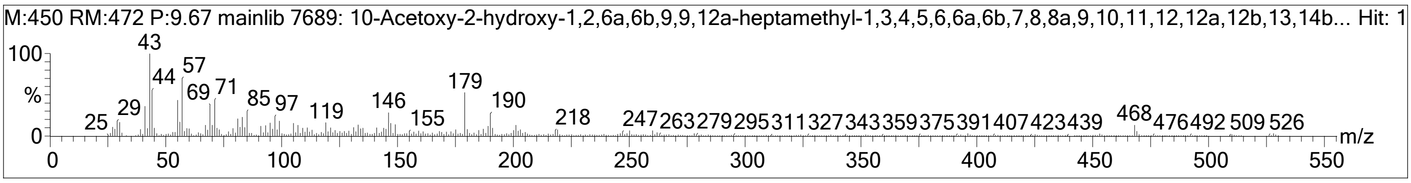

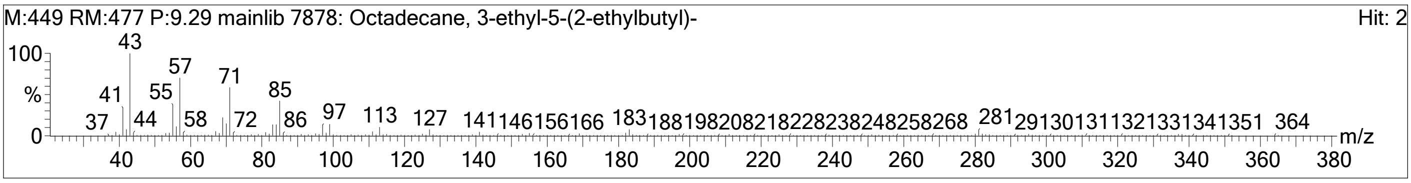

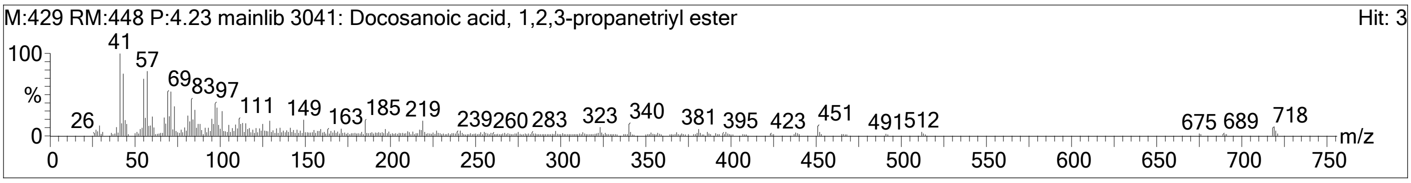

TAMILNADU AGRICULTURAL UNIVERSITY - AGRICULTURAL MICROBIOLOGY

INSTRUMENT: PERKIN ELMER CLARUS SQ8C COLOUMN: DB-5 MS CAPILARY STANDARD NON - POLARCOLOUMN
INJECTION VOL: 1 MICRO LITER DIMENSION: 30Mts, ID: 0.25 mm, FILM: 0.25 IM CARRIER GAS: He
SAMPLE ID : T260

#	RT	Scan	Height	Area	Area %	Norm %
14	30.309	5460	16,295,227	699,237.6	0.599	18.96

Pk #	RT	Hit	Compound Name	Match	R.Match	Prob.	CAS	Library
14	30.309	1	2-Nonadecanone 2,4-dinitrophenylhydrazine	396	427	15.5	28813-61-8	mainlib
		2	Hexadecanoic acid, 1a,2,5,5a,6,9,10,10a-octahydro-5a-hydroxy-4-(hydroxymethyl)-1,1,7,9-tetramethyl-6,11-dioxo-1H-2,8a-methanocyclopenta[a]cyclopropa[e]cyclodecen-5-yl ester, [1aR-(1aà,2à,5á,5aá,8aà,9à,10aà)]-	373	373	5.6	77508-69-1	mainlib
		3	2-Pyrrolidinethione, 4,4,5-trimethyl-5-(4,4-dimethyl-2-thioxo-5-pyrrolidinylidene)methyl-	366	435	4.3		mainlib
		4	17-Pentatriacontene	361	382	3.5	6971-40-0	mainlib
		5	Azuleno[4,5-b]furan-2(3H)-one, decahydro-8,9-dihydroxy-6,9a-dimethyl-3-methylene-, [3aS-(3aà,6á,6aà,8à,9à,9aá,9bà)]-	360	460	3.3	5090-67-5	mainlib
		6	Milbemycin b, 5-O-demethyl-28-deoxy-6,28-epoxy-25-(1-methylethyl)-13-(phenylthio)-, (6R,13R,25R)-	359	419	3.2	104568-15-2	mainlib
		7	i-Propyl 9-tetradecenoate	354	482	2.6		mainlib
		8	Carbamic acid, N-[10,11-dihydro-5-(2-methylamino-1-oxoethyl)-3-5H-dibenzo[b,f]azepinyl]-, ethyl ester	351	430	2.3	102821-92-1	mainlib
		9	1-Hexacosanol	346	376	1.8	506-52-5	replib
		10	12-Dimethylamino-10-oxododecanoic acid	344	473	1.7	100913-51-7	mainlib

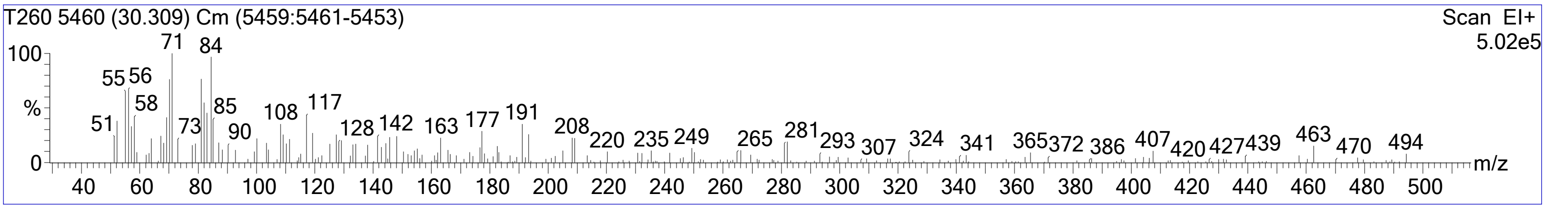

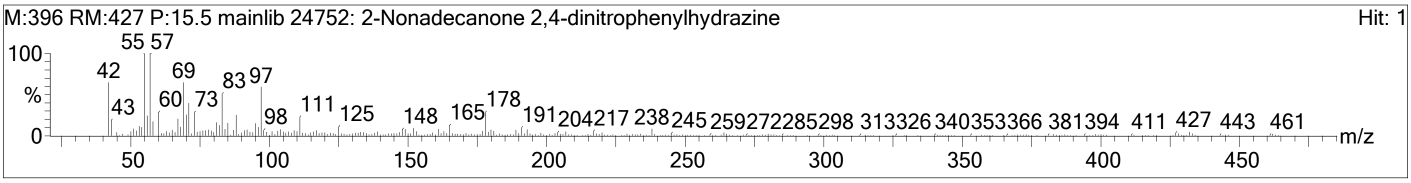

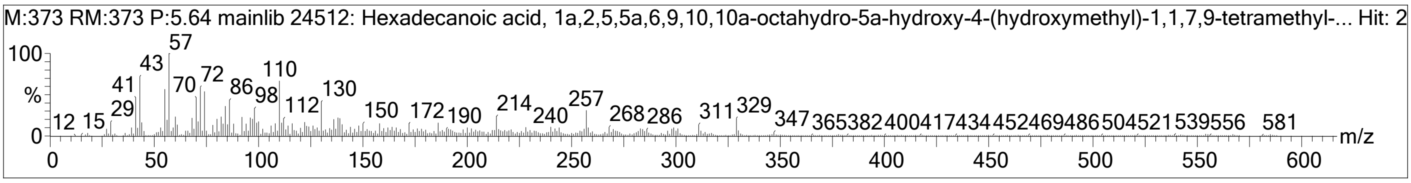

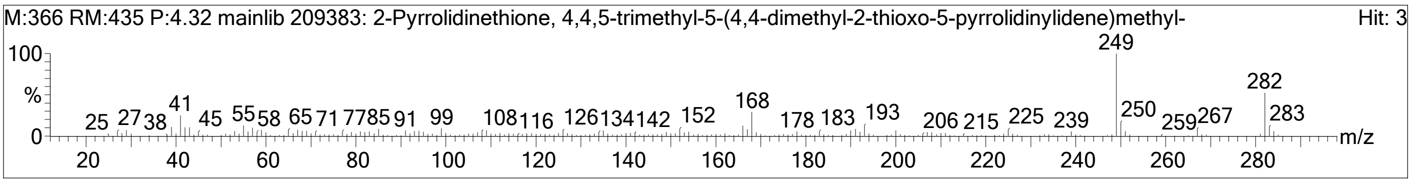

TAMILNADU AGRICULTURAL UNIVERSITY - AGRICULTURAL MICROBIOLOGY

INSTRUMENT: PERKIN ELMER CLARUS SQ8C COLOUMN: DB-5 MS CAPILARY STANDARD NON - POLARCOLOUMN
INJECTION VOL: 1 MICRO LITER DIMENSION: 30Mts, ID: 0.25 mm, FILM: 0.25 IM CARRIER GAS: He
SAMPLE ID : T260

#	RT	Scan	Height	Area	Area %	Norm %
15	30.534	5505	19,734,680	1,467,774.9	1.258	39.79

Pk #	RT	Hit	Compound Name	Match	R.Match	Prob.	CAS	Library
15	30.534	1	Oleic acid, eicosyl ester	449	463	6.8	22393-88-0	mainlib
		2	2-Nonadecanone 2,4-dinitrophenylhydrazine	439	463	4.8	28813-61-8	mainlib
		3	9-Octadecenoic acid (Z)-, octadecyl ester	436	451	4.2	17673-49-3	mainlib
		4	9-Octadecenoic acid (Z)-, tetradecyl ester	431	453	3.4	22393-85-7	mainlib
		5	7,8-Epoxylanostan-11-ol, 3-acetoxy-	428	431	3.0		mainlib
		6	17-Pentatriacontene	427	442	2.9	6971-40-0	mainlib
		7	17-Pentatriacontene	422	462	2.9	6971-40-0	replib
		8	Undec-10-enoic acid, t-butyl ester	419	547	2.2	93757-41-6	mainlib
		9	9-Hexadecenoic acid, eicosyl ester, (Z)-	418	437	2.1	22522-34-5	mainlib
		10	Cyclohexane, 1,3,5-trimethyl-2-octadecyl-	416	426	1.9	55282-34-3	mainlib

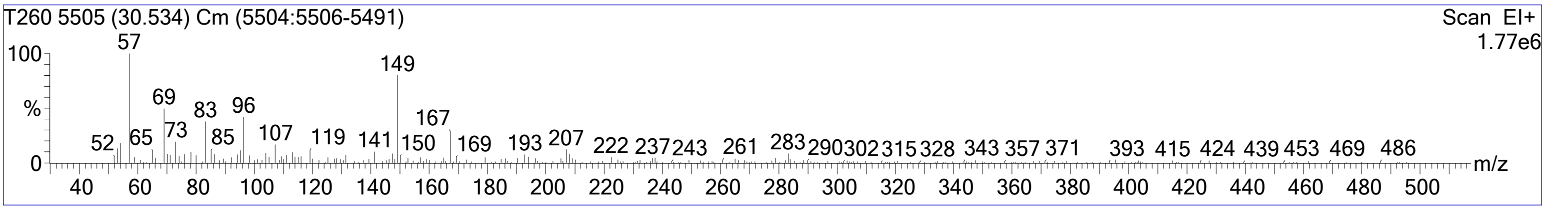

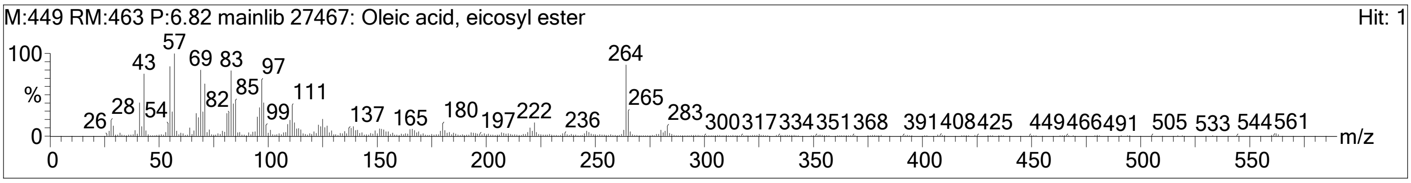

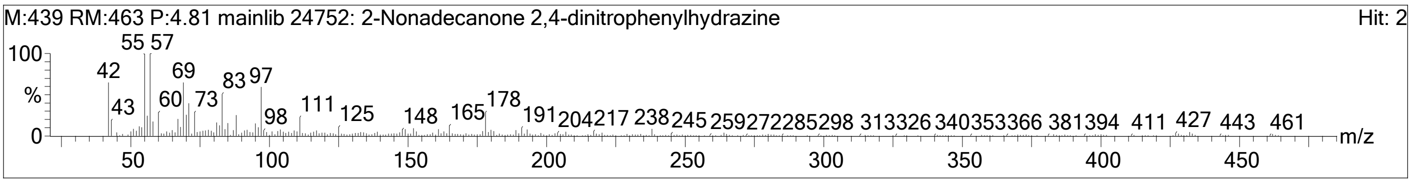

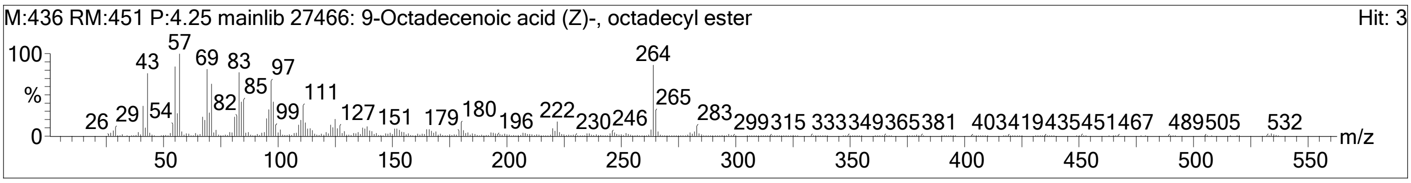

TAMILNADU AGRICULTURAL UNIVERSITY - AGRICULTURAL MICROBIOLOGY

INSTRUMENT: PERKIN ELMER CLARUS SQ8C COLOUMN: DB-5 MS CAPILARY STANDARD NON - POLARCOLOUMN
INJECTION VOL: 1 MICRO LITER DIMENSION: 30Mts, ID: 0.25 mm, FILM: 0.25 IM CARRIER GAS: He
SAMPLE ID : T260

#	RT	Scan	Height	Area	Area %	Norm %
16	30.634	5525	14,682,743	784,324.6	0.672	21.26

Pk #	RT	Hit	Compound Name	Match	R.Match	Prob.	CAS	Library
16	30.634	1	Docosahexaenoic acid, 1,2,3-propanetriyl ester	460	484	8.0	11094-59-0	mainlib
		2	1H-Cyclopropa[3,4]benz[1,2-e]azulene-5,7b,9,9a-tetrol, 1a,1b,4,4a,5,7a,8,9-octahydro-3-(hydroxymethyl)-1,1,6,8-tetramethyl-, 5,9,9a-triacetate, [1aR-(1aà,1bá,4aá,5á,7aà,7bà,8à,9á,9aà)]-	458	486	7.4	77508-64-6	mainlib
		3	5H-Cyclopropa[3,4]benz[1,2-e]azulen-5-one, 4,9,9a-tris(acetyloxy)-3-[(acetyloxy)methyl]-1,1a,1b,4,4a,7a,7b,8,9,9a-decahydro-4a,7b-dihydroxy-1,1,6,8-tetramethyl-	458	481	7.4	77573-16-1	mainlib
		4	2-[4-methyl-6-(2,6,6-trimethylcyclohex-1-enyl)hexa-1,3,5-trienyl]cyclohex-1-en-1-carboxaldehyde	452	494	5.8		mainlib
		5	Fluoxymesterone	442	489	4.1	76-43-7	replib
		6	Corynan-17-ol, 18,19-didehydro-10-methoxy-, acetate (ester)	440	488	3.8	56053-13-5	mainlib
		7	Bufa-20,22-dienolide, 14,15-epoxy-3,11-dihydroxy-, (3á,5á,11à,15á)-	438	469	3.5	39005-15-7	mainlib
		8	Fluoxymesterone	433	479	4.1	76-43-7	replib
		9	17a-Ethyl-3á-methoxy-17a-aza-D-homoandrost-5-ene-17-one	429	496	2.5	149942-11-0	mainlib
		10	5,6,7,3',4'-Pentamethoxyflavone	426	509	2.2	2306-27-6	nist_msms

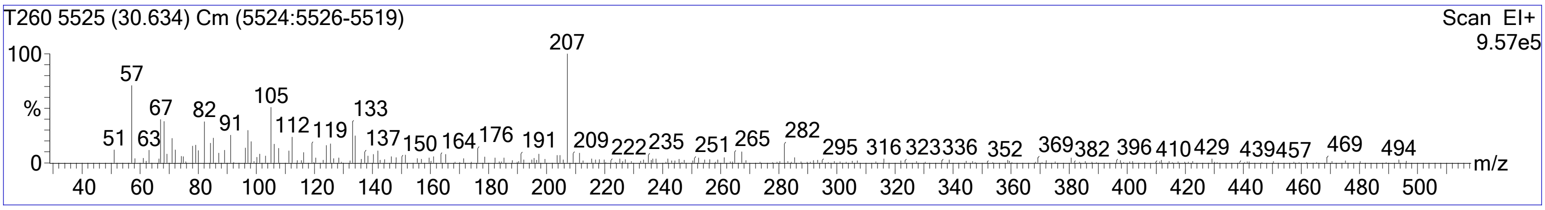

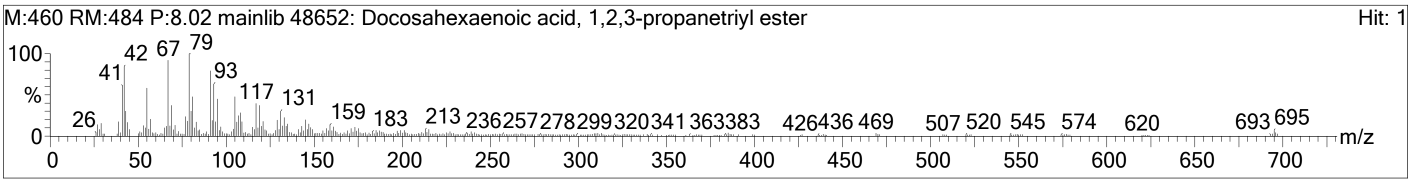

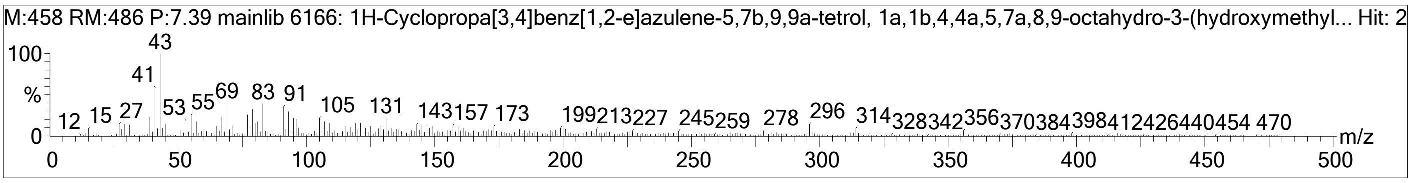

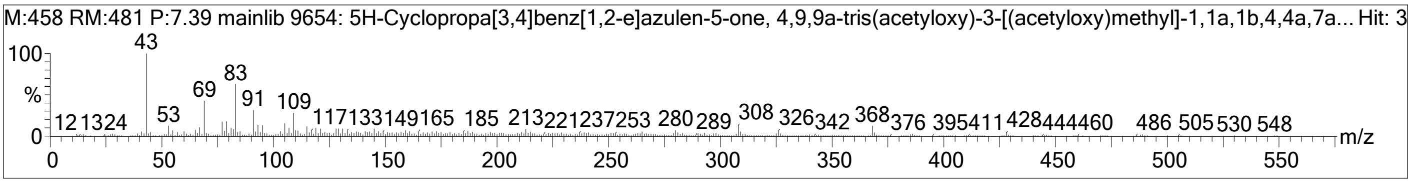

TAMILNADU AGRICULTURAL UNIVERSITY - AGRICULTURAL MICROBIOLOGY

INSTRUMENT: PERKIN ELMER CLARUS SQ8C COLOUMN: DB-5 MS CAPILARY STANDARD NON - POLARCOLOUMN
INJECTION VOL: 1 MICRO LITER DIMENSION: 30Mts, ID: 0.25 mm, FILM: 0.25 IM CARRIER GAS: He
SAMPLE ID : T260

#	RT	Scan	Height	Area	Area %	Norm %
17	30.749	5548	17,942,240	781,243.6	0.669	21.18

Pk #	RT	Hit	Compound Name	Match	R.Match	Prob.	CAS	Library
17	30.749	1	3-Isopropyl-6a,7,10b-trimethyl-dodecahydro-benzo[f]chromene-7,8-dicarboxylic acid, dimethyl ester	409	466	9.6		mainlib
		2	4,4,6a,6b,8a,11,11,14b-Octamethyl-docosahydropicen-3-ol	401	428	7.1		mainlib
		3	Dynorphin A (7-17), porcine	398	413	6.3		nist_msms2
		4	3-(2-Ethoxycarbonylmethylamino)-2-(4-chlorphenyl)-thioacrylsaeuremorpholid	397	471	6.1		mainlib
		5	2-Picenol, 4,4,6a,6b,8a,11,11,14b-octamethylperhydro	397	425	6.1		mainlib
		6	1,2-Bis[1-(2-hydroxyethyl)-3,6-diazahomoadamantantydene-9]hydrazine	396	429	5.8		mainlib
		7	2-[2-(4-Chlorophenyl)-3-morpholin-4-yl-3-thioxo-propenylamino]malonic acid, diethyl ester	395	434	5.6		mainlib
		8	Dynorphin A (7-17), porcine	392	420	4.9		nist_msms2
		9	Dynorphin A (7-17), porcine	391	417	4.7		nist_msms2
		10	Dynorphin A (7-17), porcine	384	399	3.6		nist_msms2

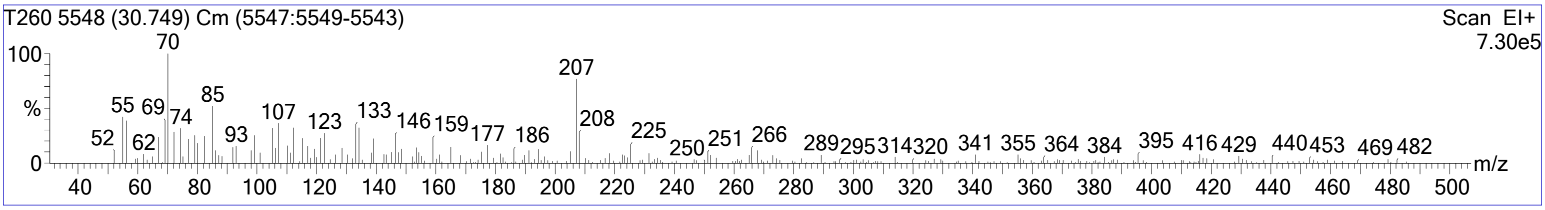

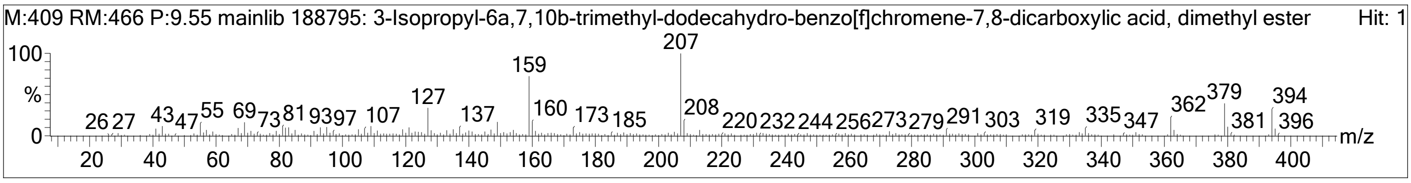

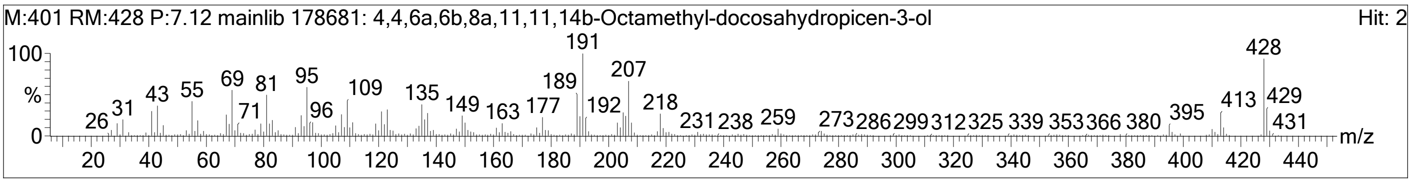

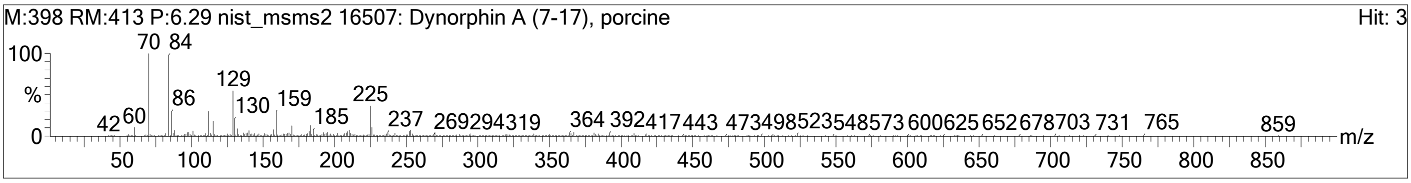

TAMILNADU AGRICULTURAL UNIVERSITY - AGRICULTURAL MICROBIOLOGY

INSTRUMENT: PERKIN ELMER CLARUS SQ8C COLOUMN: DB-5 MS CAPILARY STANDARD NON - POLARCOLOUMN
INJECTION VOL: 1 MICRO LITER DIMENSION: 30Mts, ID: 0.25 mm, FILM: 0.25 IM CARRIER GAS: He
SAMPLE ID : T260

#	RT	Scan	Height	Area	Area %	Norm %
18	30.874	5573	16,203,522	1,420,392.1	1.217	38.50

Pk #	RT	Hit	Compound Name	Match	R.Match	Prob.	CAS	Library
18	30.874	1	Octadecane, 1,1'-[1,3-propanediylbis(oxy)]bis-	432	443	6.0	17367-38-3	mainlib
		2	Spirost-8-en-11-one, 3-hydroxy-, (3á,5à,14á,20á, 22á,25R)-	429	473	5.3	58072-54-1	mainlib
		3	Stearic acid, 3-(octadecyloxy)propyl ester	429	450	5.3	17367-40-7	mainlib
		4	2,4-Di-tert-butylthiophenol	419	586	3.7	19728-43-9	mainlib
		5	9-Octadecenoic acid, (2-phenyl-1,3-dioxolan-4-yl)methyl ester, cis-	419	449	3.7	56599-45-2	mainlib
		6	4-Dehydroxy-N-(4,5-methylenedioxy-2-nitrobenzylidene)tyramine	412	494	2.9		mainlib
		7	Strychane, 1-acetyl-20à-hydroxy-16-methylene-	412	465	2.9	2111-98-0	mainlib
		8	3-Isopropyl-6a,10b-dimethyl-8-(2-oxo-2-phenyl-ethyl)-dodecahydro-benzo[f]chromen-7-one	412	449	2.9		mainlib
		9	Demecolcine	405	465	2.2	477-30-5	replib
		10	Octadecanoic acid, (2-phenyl-1,3-dioxolan-4-yl)methyl ester, cis-	405	441	2.2	56599-88-3	mainlib

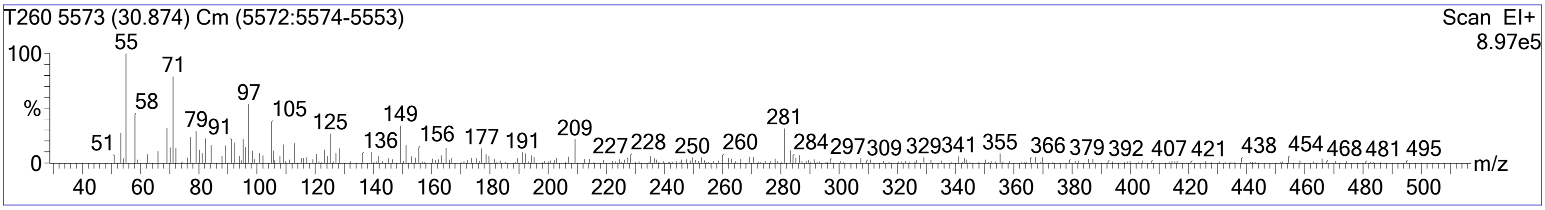

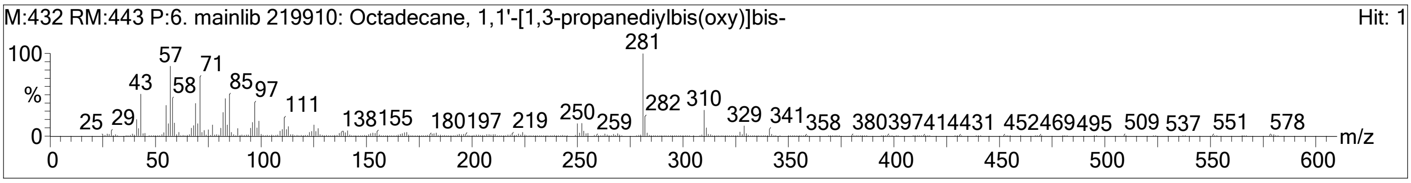

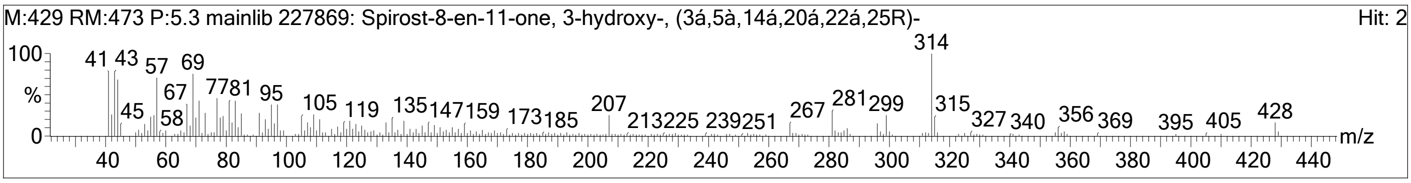

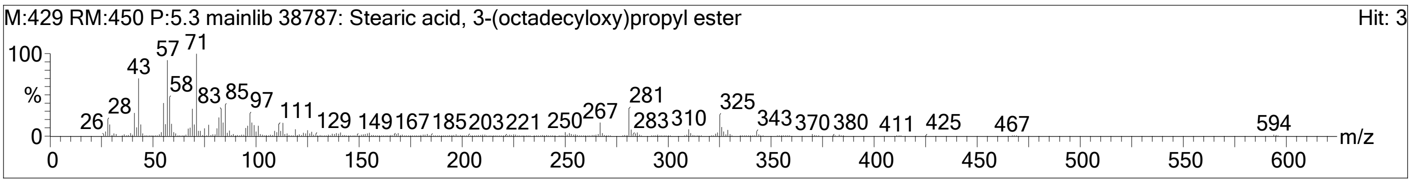

TAMILNADU AGRICULTURAL UNIVERSITY - AGRICULTURAL MICROBIOLOGY

INSTRUMENT: PERKIN ELMER CLARUS SQ8C COLOUMN: DB-5 MS CAPILARY STANDARD NON - POLARCOLOUMN
INJECTION VOL: 1 MICRO LITER DIMENSION: 30Mts, ID: 0.25 mm, FILM: 0.25 IM CARRIER GAS: He
SAMPLE ID : T260

#	RT	Scan	Height	Area	Area %	Norm %
19	30.904	5579	17,055,568	726,942.8	0.623	19.71

Pk #	RT	Hit	Compound Name	Match	R.Match	Prob.	CAS	Library
19	30.904	1	17-Pentatriacontene	437	464	10.4	6971-40-0	mainlib
		2	4,7-Methano-1H-inden-1-one, 3,4,5,6,7,8,8-heptachloro-3a,4,5,6,7,7a-hexahydro-, (3aà,4á,5à,6à,7á,7aà)-	435	470	9.6	51179-71-6	mainlib
		3	Hexanoic acid, 3,5,5-trimethyl-, 1,2,3-propanetriyl ester	420	522	5.8	56554-53-1	mainlib
		4	Propanoic acid, 2-(3-acetoxy-4,4,14-trimethylandrost-8-en-17-yl)-	418	431	5.4		mainlib
		5	1-Dodecanol, 3,7,11-trimethyl-	411	535	4.1	6750-34-1	replib
		6	Norgestrel, bis(trimethylsilyl) derivative	409	435	3.8		mainlib
		7	Azuleno[4,5-b]furan-2(3H)-one, decahydro-8,9-dihydroxy-6,9a-dimethyl-3-methylene-, [3aS-(3aà,6á,6aà,8à,9à,9aá,9bà)]-	408	509	3.7	5090-67-5	mainlib
		8	n-Butyl ricinoleate	407	470	3.5	151-13-3	mainlib
		9	Hexadecane, 1,1-bis(dodecyloxy)-	402	460	2.8	56554-64-4	mainlib
		10	2-Eicosanol	399	456	2.5	4340-76-5	mainlib

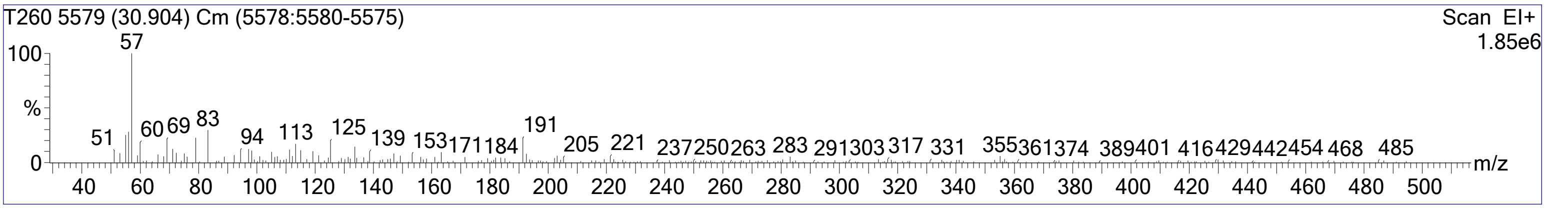

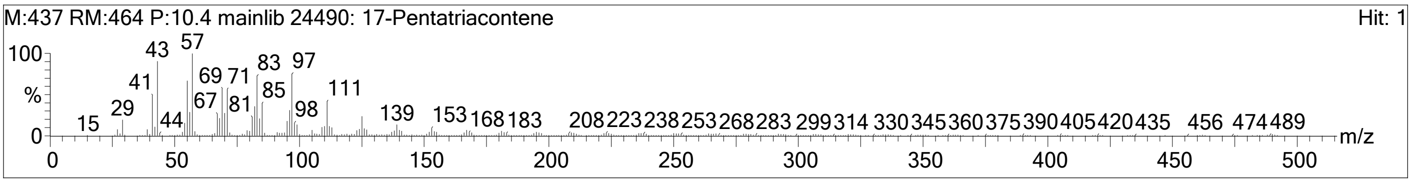

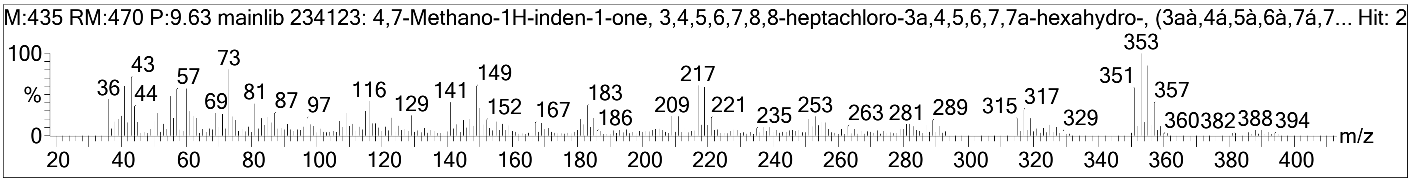

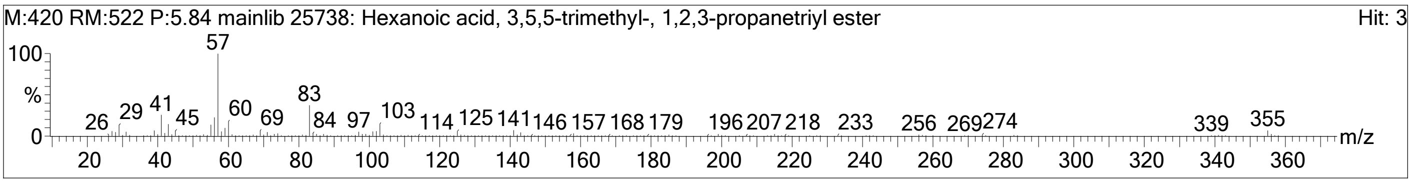

TAMILNADU AGRICULTURAL UNIVERSITY - AGRICULTURAL MICROBIOLOGY

INSTRUMENT: PERKIN ELMER CLARUS SQ8C COLOUMN: DB-5 MS CAPILARY STANDARD NON - POLARCOLOUMN
INJECTION VOL: 1 MICRO LITER DIMENSION: 30Mts, ID: 0.25 mm, FILM: 0.25 IM CARRIER GAS: He
SAMPLE ID : T260

#	RT	Scan	Height	Area	Area %	Norm %
20	31.054	5609	19,840,662	1,248,544.1	1.070	33.85

Pk #	RT	Hit	Compound Name	Match	R.Match	Prob.	CAS	Library
20	31.054	1	2-Nonadecanone 2,4-dinitrophenylhydrazine	459	483	30.5	28813-61-8	mainlib
		2	1,5-Bis(4-methoxyphenyl)bicyclo[3.2.0]heptane	422	470	7.4	157367-56-1	mainlib
		3	2a,6a-Methano-1H-cyclopropa[b]naphthalene-1,1,8,8-tetrachloro-1a,2,3,6,7,7a-hexahydro-	414	477	5.5	102618-60-0	mainlib
		4	18,19-Secoyohimban-19-oic acid, 16,17,20,21-tetradehydro-16-(hydroxymethyl)-, methyl ester, (15á,16E)-	404	462	3.9	5523-49-9	mainlib
		5	Chromone, 5-hydroxy-6,7,8-trimethoxy-2,3-dimethyl-	397	446	3.0		mainlib
		6	16-Nitrobicyclo[10.4.0]hexadecan-1-ol-13-one	396	447	2.9	79880-69-6	mainlib
		7	Fumaric acid, heptadecyl propyl ester	392	464	2.4		mainlib
		8	Isocalamendiol	391	512	2.3		mainlib
		9	9-Octadecenoic acid, 1,2,3-propanetriyl ester, (E,E,E)-	391	417	2.3	537-39-3	mainlib
		10	Eicosanoic acid, 2-(acetyloxy)-1-[(acetyloxy)methyl]ethyl ester	389	565	2.2	55429-68-0	mainlib

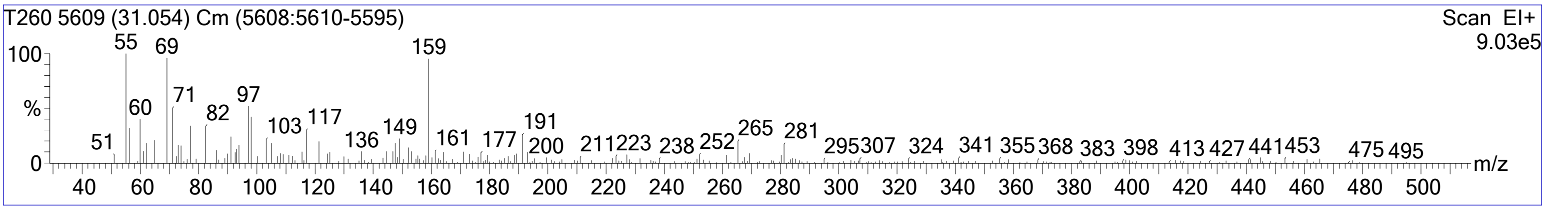

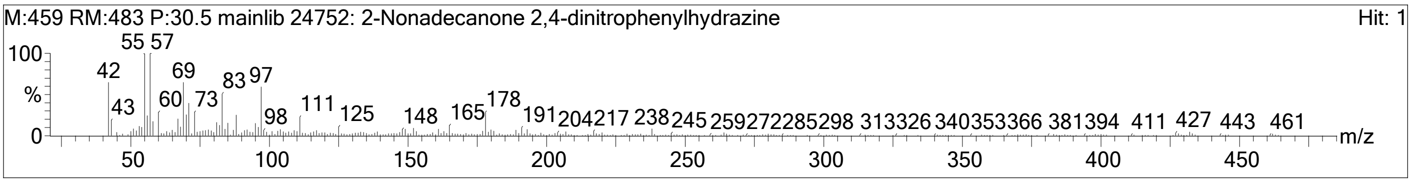

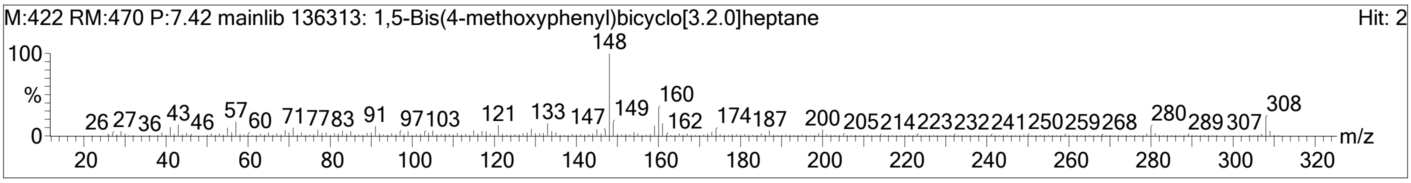

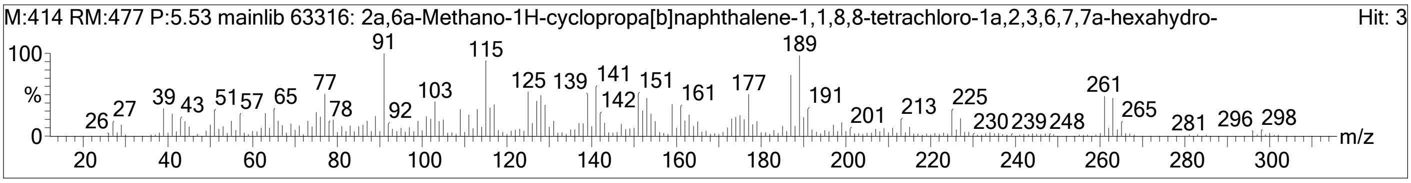

TAMILNADU AGRICULTURAL UNIVERSITY - AGRICULTURAL MICROBIOLOGY

INSTRUMENT: PERKIN ELMER CLARUS SQ8C COLOUMN: DB-5 MS CAPILARY STANDARD NON - POLARCOLOUMN
INJECTION VOL: 1 MICRO LITER DIMENSION: 30Mts, ID: 0.25 mm, FILM: 0.25 IM CARRIER GAS: He
SAMPLE ID : T260

#	RT	Scan	Height	Area	Area %	Norm %
21	31.149	5628	21,113,964	1,459,715.2	1.251	39.57

Pk #	RT	Hit	Compound Name	Match	R.Match	Prob.	CAS	Library
21	31.149	1	N,N'-Trimethylenebis[s-3-aminopropylthiosulfuric acid]	456	522	27.9	35871-60-4	mainlib
		2	2-Myristynoyl pantetheine	435	498	11.9		mainlib
		3	2-[2-(4-Chlorophenyl)-3-morpholin-4-yl-3-thioxo-propenylamino]acetamide	422	466	7.7		mainlib
		4	1,4,10,13-Tetraoxa-7,16-diazacyclooctadecane, 7, 16-bis(1-oxodecyl)-	406	413	4.4	105400-04-2	mainlib
		5	á,î-Carotene-3,3'-diol, (3R,3'R,6'R)-	394	482	2.9	127-40-2	replib
		6	Gentamicin a	392	496	2.7	13291-74-2	mainlib
		7	Spirostan-9-ol, 3-amino-, (3á,5à,25R)-	386	433	2.1	16577-35-8	mainlib
		8	1-Hexadecanol, 2-methyl-	382	447	1.8	2490-48-4	mainlib
		9	Anthracene, 9-dodecyltetradecahydro-	375	400	1.4	55401-75-7	replib
		10	Eicosanoic acid, 2-(acetyloxy)-1-[(acetyloxy)methyl]ethyl ester	373	534	1.3	55429-68-0	mainlib

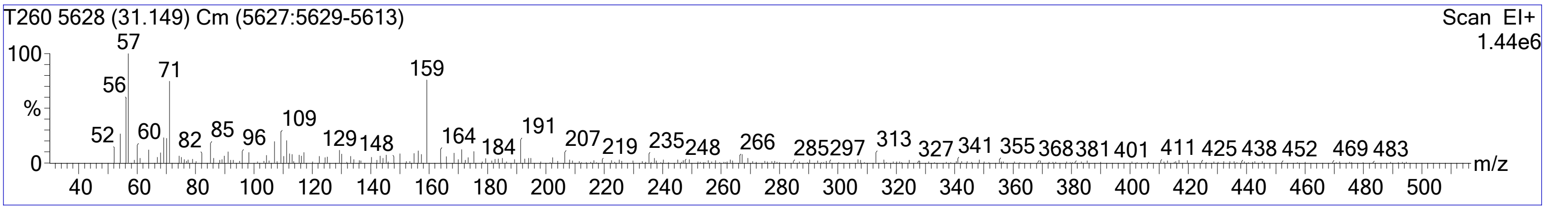

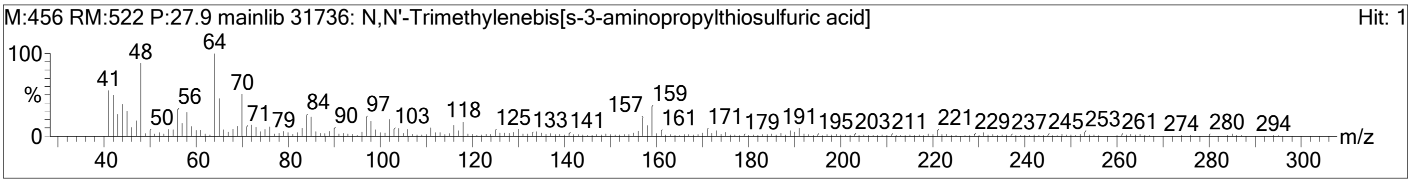

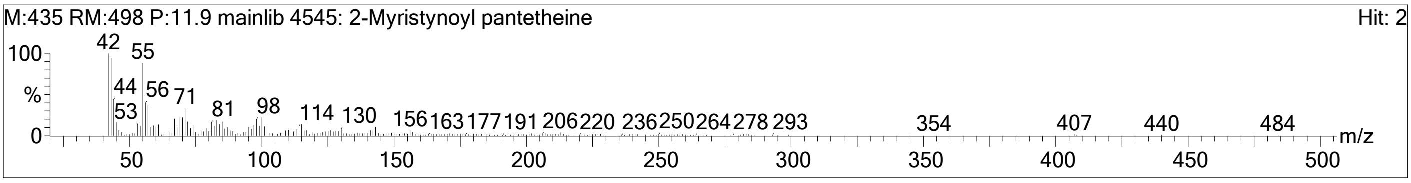

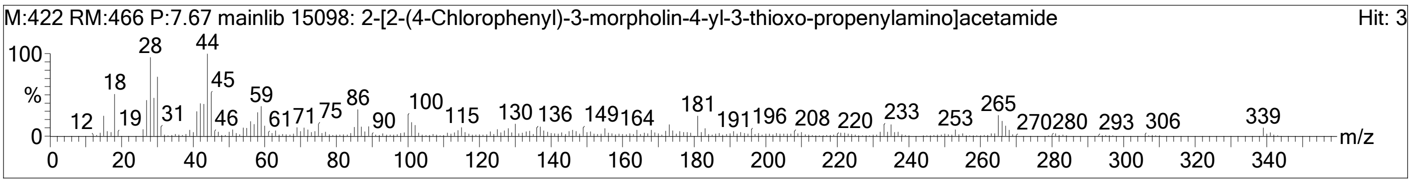

TAMILNADU AGRICULTURAL UNIVERSITY - AGRICULTURAL MICROBIOLOGY

INSTRUMENT: PERKIN ELMER CLARUS SQ8C COLOUMN: DB-5 MS CAPILARY STANDARD NON - POLARCOLOUMN
INJECTION VOL: 1 MICRO LITER DIMENSION: 30Mts, ID: 0.25 mm, FILM: 0.25 IM CARRIER GAS: He
SAMPLE ID : T260

#	RT	Scan	Height	Area	Area %	Norm %
22	31.174	5633	18,674,802	1,229,540.4	1.053	33.33

Pk #	RT	Hit	Compound Name	Match	R.Match	Prob.	CAS	Library
22	31.174	1	8,14-Seco-3,19-epoxyandrostane-8,14-dione, 17-acetoxy-3á-methoxy-4,4-dimethyl-	487	518	27.9		mainlib
		2	9,12,15-Octadecatrienoic acid, 2-phenyl-1,3-dioxan-5-yl ester	455	479	7.5	56700-76-6	mainlib
		3	9,12-Octadecadienoic acid, (2-phenyl-1,3-dioxolan-4-yl)methyl ester, trans-	440	455	4.5	56599-48-5	mainlib
		4	9,12-Octadecadienoic acid, (2-phenyl-1,3-dioxolan-4-yl)methyl ester, cis-	437	451	4.0	56599-47-4	mainlib
		5	Ursodeoxycholic acid	429	457	3.0	128-13-2	mainlib
		6	E,E,Z-1,3,12-Nonadecatriene-5,14-diol	425	491	2.5		mainlib
		7	Pregnane-3,11,17,20-tetrol, cyclic 17,20-[(1,1-dimethylethyl)boronate], (3à,5á,11á,20R)-	425	476	2.5	30882-63-4	mainlib
		8	9,12-Octadecadienoic acid, 2-phenyl-1,3-dioxan-5-yl ester, cis-	423	437	2.3	56687-50-4	mainlib
		9	5,14,23-Octadecatrien-14,15-diol	421	488	2.1		mainlib
		10	6-(1,5-Dimethyl-hex-4-enyl)-1,6-dihydroxy-1,8a-dimethyl-3-oxo-1,2,3,3a,5a,6,7,8,8a,9,10,10a-dodecahydrodicyclopenta[a,e]cyclooctene	421	451	2.1		mainlib

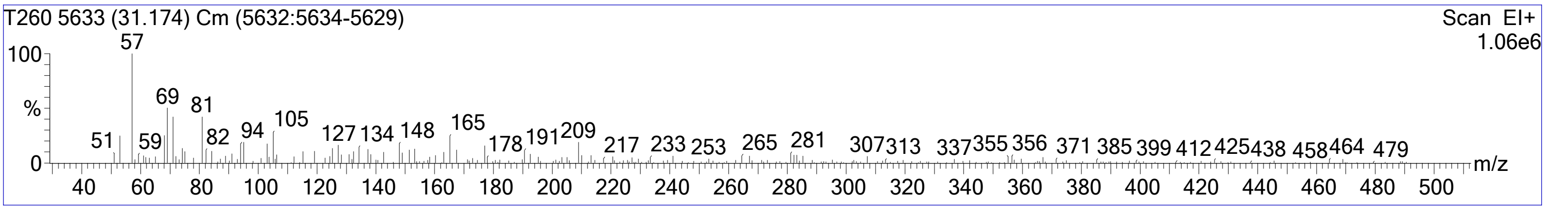

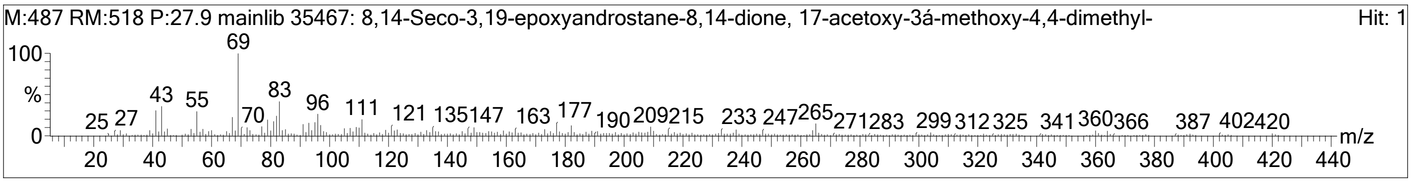

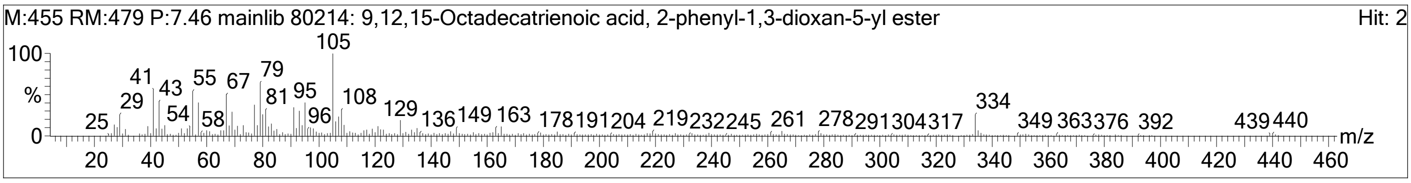

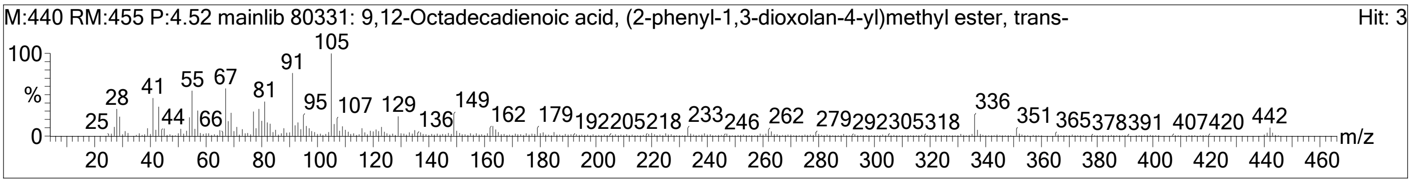

TAMILNADU AGRICULTURAL UNIVERSITY - AGRICULTURAL MICROBIOLOGY

INSTRUMENT: PERKIN ELMER CLARUS SQ8C COLOUMN: DB-5 MS CAPILARY STANDARD NON - POLARCOLOUMN
INJECTION VOL: 1 MICRO LITER DIMENSION: 30Mts, ID: 0.25 mm, FILM: 0.25 IM CARRIER GAS: He
SAMPLE ID : T260

#	RT	Scan	Height	Area	Area %	Norm %
23	31.330	5664	14,161,799	976,075.2	0.836	26.46

Pk #	RT	Hit	Compound Name	Match	R.Match	Prob.	CAS	Library
23	31.330	1	9-Desoxo-9-x-acetoxy-3,8,12-tri-O-acetylingol	457	487	13.7		mainlib
		2	4a,7a-Epoxy-5H-cyclopenta[a]cyclopropa[f]cycloundecene-2,4,7,10,11-pentol, 1,1a,2,3,4,6,7,10,11,11a-decahydro-1,1,3,6,9-pentamethyl-, 2,7,10,11-tetraacetate	443	475	8.6	51950-35-7	mainlib
		3	Corticosterone, bis(O-ethyloxime)	442	472	8.2		mainlib
		4	1H-Cyclopropa[3,4]benz[1,2-e]azulene-5,7b,9,9a-tetrol, 3-[(acetyloxy)methyl]-1a,1b,4,4a,5,7a,8,9-octahydro-1,1,6,8-tetramethyl-, 9,9a-diacetate, [1aR-(1aà,1bá,4aà,5á,7aà,7bà,8à,9á,9aà)]-	442	455	8.2	77550-15-3	mainlib
		5	4,13,20-Tri-O-methylphorbol 12-acetate	430	453	5.5		mainlib
		6	Acetic acid, 17-acetoxy-4,4,10,13-tetramethyl-7-oxo-2,3,4,7,8,9,10,11,12,13,14,15,16,17-tetradecahydro-1H-cyclopenta[a]phenanthren-3-yl (ester)	412	447	2.8		mainlib
		7	Milbemycin b, 5-O-demethyl-28-deoxy-6,28-epoxy-25-(1-methylethyl)-13-(methylthio)-, (6R,13R,25R)-	412	441	2.8	104568-10-7	mainlib
		8	2á,4a-Epoxymethylphenanthrene-7-methanol, 1,1-dimethyl-2-methoxy-8-(1,3-dithiin-2-ylidene)methyl-1,2,3,4,4a,4b,5,6,7,8,8a,9-dodecahydro-, acetate	405	413	2.2		mainlib
		9	Voaluteine, 20-hydroxy-, (20S)-	404	459	2.1	18646-15-6	mainlib
		10	1,2-Bis[1-(2-hydroxyethyl)-3,6-diazahomoadamantantydene-9]hydrazine	404	435	2.1		mainlib

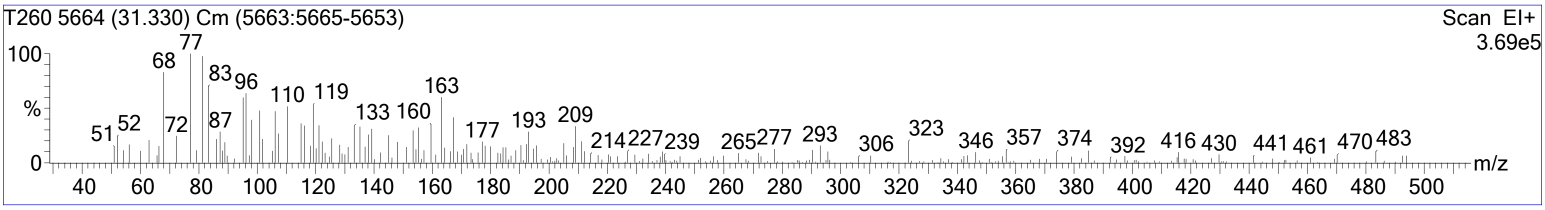

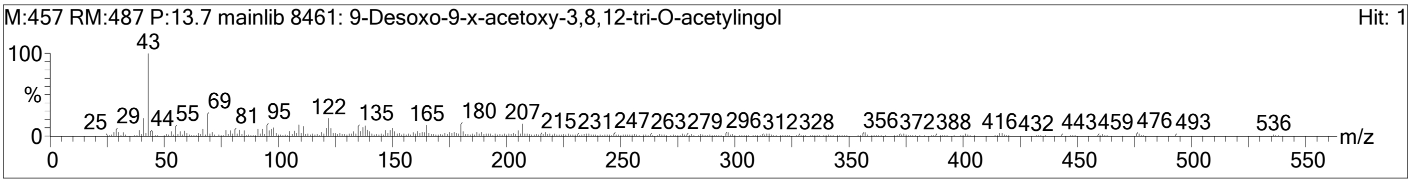

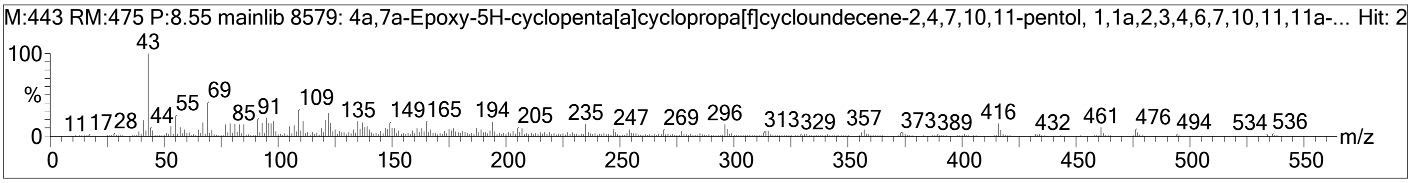

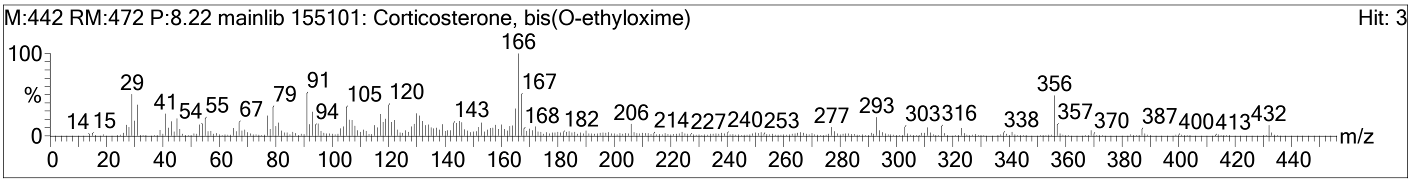

TAMILNADU AGRICULTURAL UNIVERSITY - AGRICULTURAL MICROBIOLOGY

INSTRUMENT: PERKIN ELMER CLARUS SQ8C COLOUMN: DB-5 MS CAPILARY STANDARD NON - POLARCOLOUMN
INJECTION VOL: 1 MICRO LITER DIMENSION: 30Mts, ID: 0.25 mm, FILM: 0.25 IM CARRIER GAS: He
SAMPLE ID : T260

#	RT	Scan	Height	Area	Area %	Norm %
24	31.790	5756	11,670,283	847,591.8	0.726	22.98

Pk #	RT	Hit	Compound Name	Match	R.Match	Prob.	CAS	Library
24	31.790	1	Propanoic acid, 2-(3-acetoxy-4,4,14-trimethylandrost-8-en-17-yl)-	422	436	14.1		mainlib
		2	Hydrocortisone 21-acetate, bis(O-ethyloxime)	417	427	11.4		mainlib
		3	2-Myristynoyl pantetheine	401	471	6.6		mainlib
		4	5H-Cyclopropa[3,4]benz[1,2-e]azulen-5-one, 2,4a,9,9a-tetrakis(acetyloxy)-3,[(acetyloxy)methyl]-1,1a,1b,2,3,4,4a,7a,7b,8,9,9a-dodecahydro-2,7b-dihydroxy-1,1,6,8-tetramethyl-, [1aR-(1aà,1bá,2à,3á,4aá,7aà,7bà,8à,9á,9aà)]-	400	409	6.3	77573-25-2	mainlib
		5	Cyclopropa[3',4']benz[1',2':4,5]azuleno[1,8a-d]-1,3-dioxole-5b,7,7a-triol, 3a,5a,6,7,8,8a,8b,11-octahydro-10-(hydroxymethyl)-2,2,4,6,8,8-hexamethyl-, 7,7a-diacetate, [3aS-(3aà,5aà,5bà,6à,7á,7aà,8aà,8bá,11aS*)]-	400	403	6.3	77573-38-7	mainlib
		6	Spirostan-9-ol, 3-amino-, (3á,5à,25R)-	391	440	4.6	16577-35-8	mainlib
		7	Cholest-4-ene, 3á-(methoxymethoxy)-	390	411	4.4	4707-85-1	mainlib
		8	3-[3-Bromophenyl]-7-chloro-3,4-dihydro-10-hydroxy-1,9(2H,10H)-acridinedione	385	454	3.5	144128-92-7	mainlib
		9	N-(5-Hydroxy-2-oxo-5-phenyl-1-aza-bicyclo[4.2.0]oct-3-yl)carbamic acid, benzyl ester	372	421	2.3		mainlib
		10	2-Oxazolamine, 4,5-dihydro-5-(phenoxymethyl)-N-phenyl-	371	447	2.2	25409-59-0	mainlib

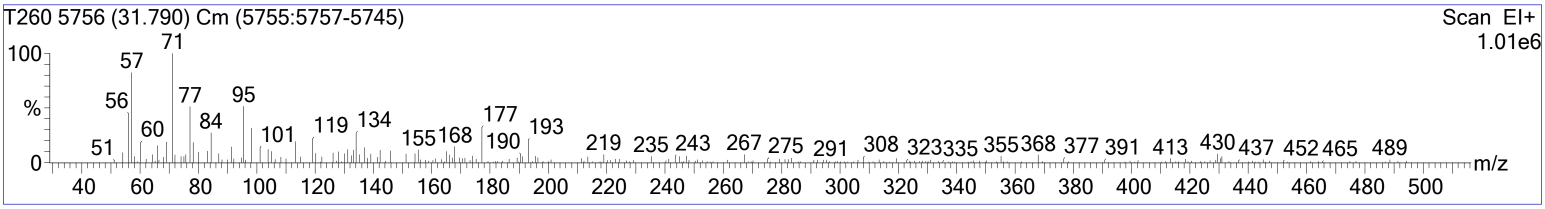

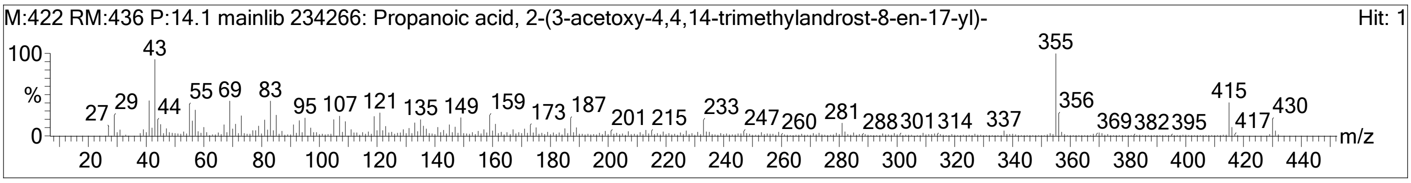

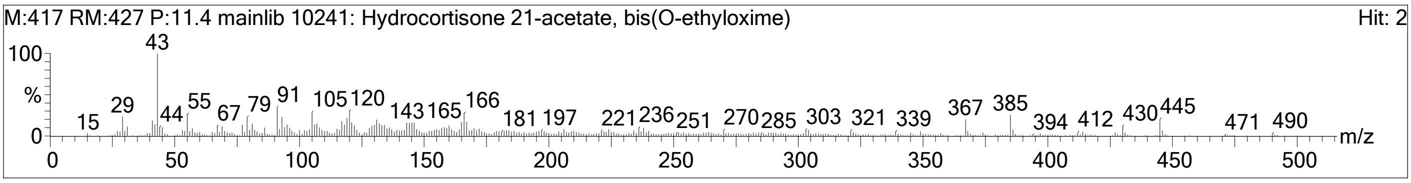

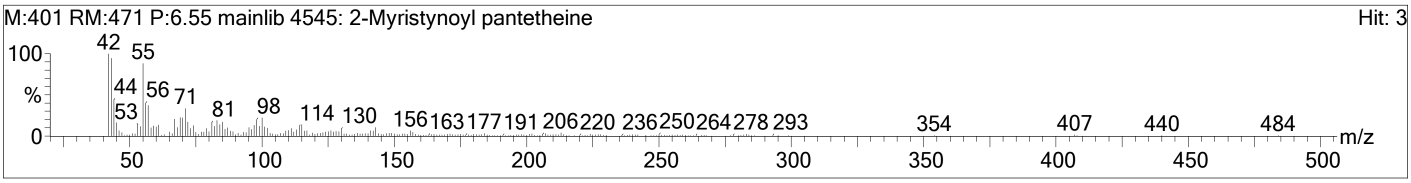

TAMILNADU AGRICULTURAL UNIVERSITY - AGRICULTURAL MICROBIOLOGY

INSTRUMENT: PERKIN ELMER CLARUS SQ8C COLOUMN: DB-5 MS CAPILARY STANDARD NON - POLARCOLOUMN
INJECTION VOL: 1 MICRO LITER DIMENSION: 30Mts, ID: 0.25 mm, FILM: 0.25 IM CARRIER GAS: He
SAMPLE ID : T260

#	RT	Scan	Height	Area	Area %	Norm %
25	32.685	5935	21,193,474	943,316.8	0.808	25.57

Pk #	RT	Hit	Compound Name	Match	R.Match	Prob.	CAS	Library
25	32.685	1	10,13-Octadecadiynoic acid, methyl ester	423	477	8.6	18202-24-9	mainlib
		2	4,11-Dispiro(2'-cyclobutanone)tricyclo[6.2.2.0(2,7)]dodeca-5,9-diene, 1,3,3,5,12,12-hexamethyl-	419	465	7.2		mainlib
		3	Androst-5-en-3-ol-17-one, 16,16-trimethylenedithio-	414	478	5.8		mainlib
		4	17a-Methyl-3á-methoxy-17a-aza-D-homoandrost-5-ene-17-one	409	474	4.7	149942-10-9	mainlib
		5	Calcitriol	404	489	3.8	32222-06-3	mainlib
		6	Spiro[(tricyclo[6.2.2.0(2,7)]dodeca-5,9-diene)-4,1'-cyclobutane]-11,2'-dione, 1,3,3,5,12,12-hexamethyl-	402	476	3.5		mainlib
		7	Mifepristone	397	505	2.8	84371-65-3	nist_msms
		8	Benzoic acid, 3,5-dimethyl-, (3,5-dimethylphenyl)methyl ester	393	475	2.4	55000-47-0	mainlib
		9	Ethane, 1,2-bis(2-methyl-5-nitrophenyl)-	393	450	2.4		mainlib
		10	Doconexent, TBDMS derivative	392	432	2.3		mainlib

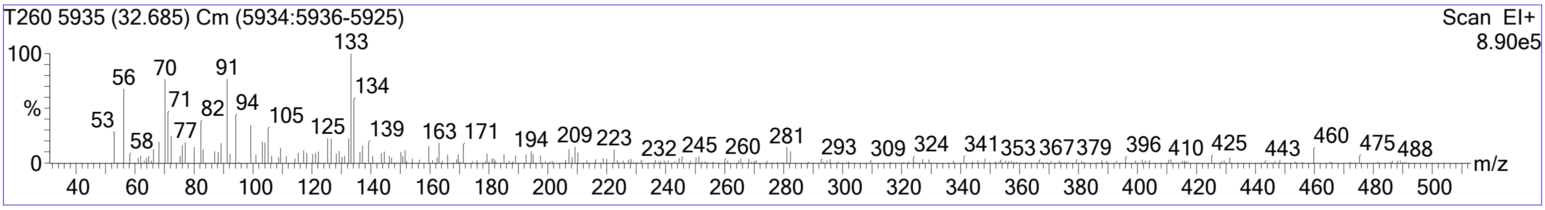

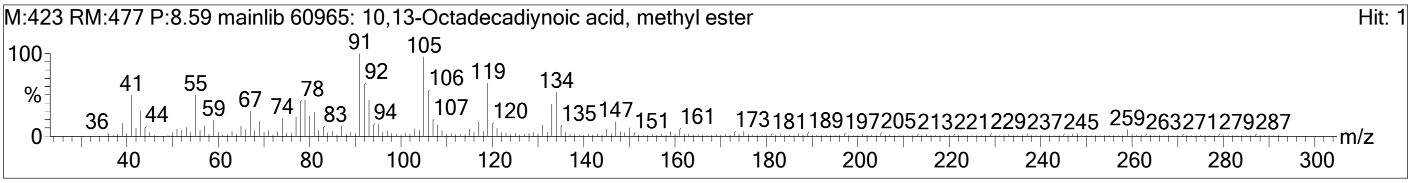

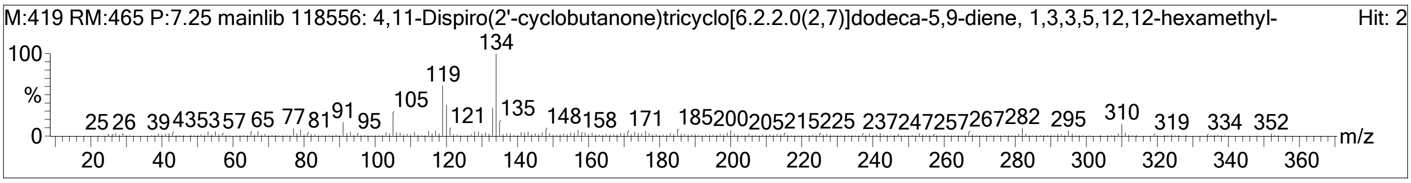

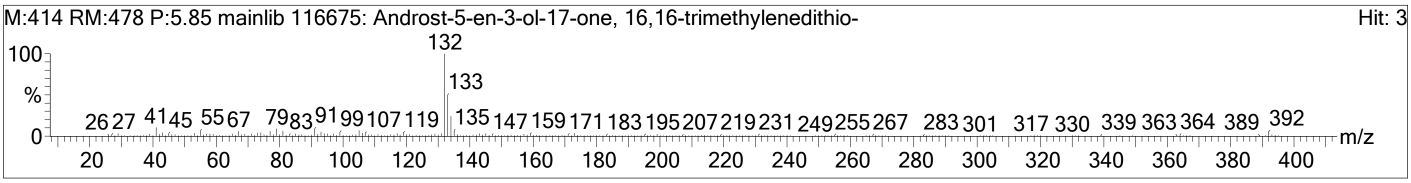

TAMILNADU AGRICULTURAL UNIVERSITY - AGRICULTURAL MICROBIOLOGY

INSTRUMENT: PERKIN ELMER CLARUS SQ8C COLOUMN: DB-5 MS CAPILARY STANDARD NON - POLARCOLOUMN
INJECTION VOL: 1 MICRO LITER DIMENSION: 30Mts, ID: 0.25 mm, FILM: 0.25 IM CARRIER GAS: He
SAMPLE ID : T260

#	RT	Scan	Height	Area	Area %	Norm %
26	32.780	5954	21,464,390	1,914,271.1	1.640	51.89

Pk #	RT	Hit	Compound Name	Match	R.Match	Prob.	CAS	Library
26	32.780	1	Lycopene	397	398	10.7	502-65-8	replib
		2	Cholestan-3-ol, 5-chloro-6-nitro-, (3á,5à,6á)-	392	402	8.6	15505-91-6	replib
		3	Pregn-5-en-20-one, 12-(acetyloxy)-3,8,14-trihydroxy-, (3á,12á,14á)-	386	434	6.8	55955-56-1	mainlib
		4	Cholestan-3-ol, 5-chloro-6-nitro-, (3á,5à,6á)-	383	396	8.6	15505-91-6	mainlib
		5	Spiro-6-(bicyclo[3.2.1]octane)-2'-(oxirane), 7,8-di(hydroxymethyl)-5-methyl-2-isopropyl-	382	465	5.7		mainlib
		6	Cholestan-3-ol, 5-chloro-6-nitro-, acetate (ester), (3á,5à,6á)-	381	388	5.5	1431-22-7	mainlib
		7	Cholestan-3-ol, 5-chloro-6-nitro-, acetate (ester), (3á,5à,6á)-	372	381	5.5	1431-22-7	replib
		8	Spironolactone	371	446	3.9	52-01-7	nist_msms
		9	5Z,?8Z,?11Z,?14Z-Eeicosatetraenoic acid,? 3-?theinylmethyl ester	366	427	3.1	390824-17-6	mainlib
		10	Androstan-3-one, 17-methoxy-, 3-methoxime, (5à, 17á)-	365	419	3.0		mainlib

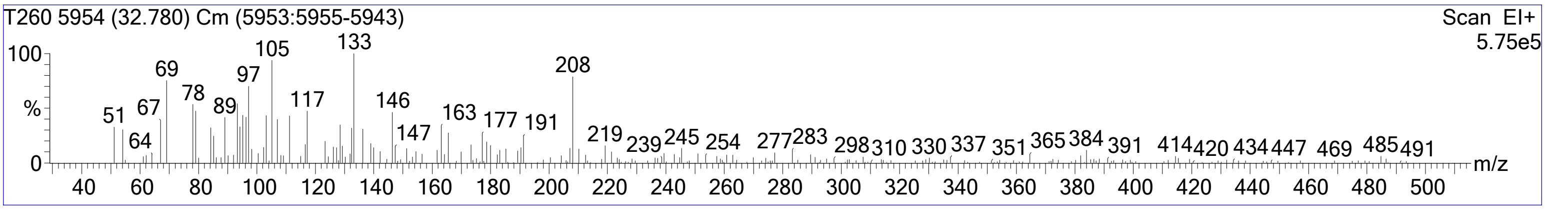

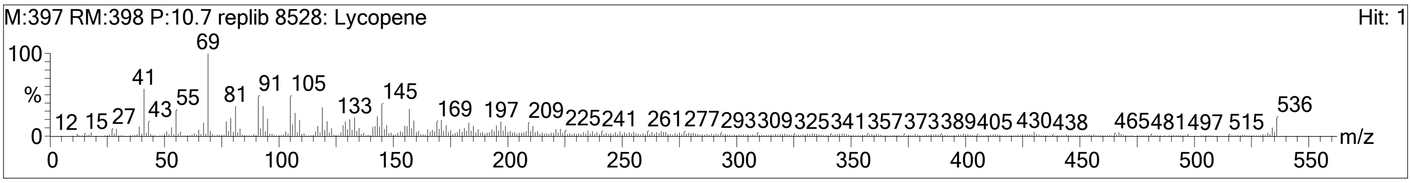

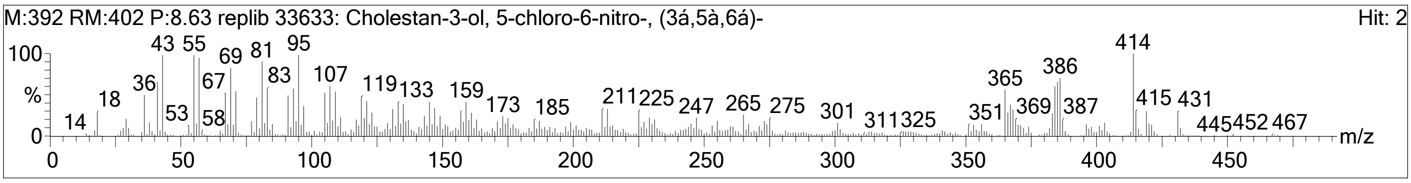

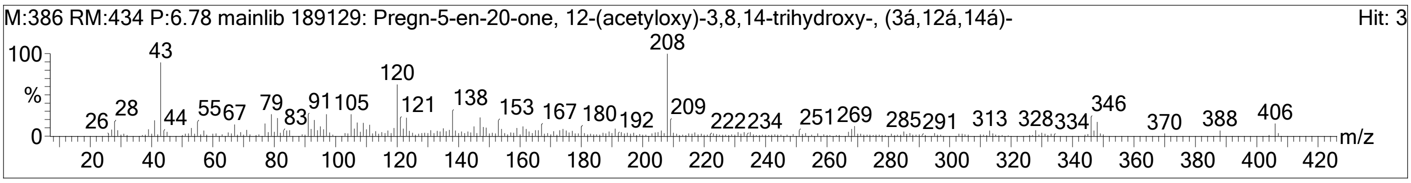

TAMILNADU AGRICULTURAL UNIVERSITY - AGRICULTURAL MICROBIOLOGY

INSTRUMENT: PERKIN ELMER CLARUS SQ8C　　COLOUMN: DB-5 MS CAPILARY STANDARD NON - POLARCOLOUMN
INJECTION VOL: 1 MICRO LITER　　DIMENSION: 30Mts, ID: 0.25 mm, FILM: 0.25 IM　　CARRIER GAS: He
SAMPLE ID : T260

#	RT	Scan	Height	Area	Area %	Norm %
27	32.845	5967	18,290,328	1,214,588.5	1.041	32.93

Pk #	RT	Hit	Compound Name	Match	R.Match	Prob.	CAS	Library
27	32.845	1	Olean-12-ene-3,16,21,22,28-pentol, (3á,16à,21á, 22à)-	415	501	17.8	13844-01-4	mainlib
		2	Testolactone	395	455	8.1	968-93-4	mainlib
		3	Salinomycin	390	490	6.6	53003-10-4	nist_msms
		4	3-Hydroxy-2,5,5,8a-tetramethyl-3,4,4a,5,6,7,8,8a-octahydronaphthalene-1-carboxylic acid, methyl ester	390	472	6.6		mainlib
		5	Testolactone	390	465	8.1	968-93-4	replib
		6	7-Oxabicyclo[2.2.1]heptane, 2,5-diethoxy-1,4-bis(trifluoromethyl)-	389	521	6.3	128097-71-2	mainlib
		7	Cyclododecanone, 4-(12-bromo-1-oxododecyl)-	383	436	4.9		mainlib
		8	Salinomycin	371	493	6.6	53003-10-4	nist_msms
		9	1-Cyclohexene-1-acrylic acid, 2,6,6-trimethyl-3-oxo-, methyl ester	371	456	3.3	28043-03-0	mainlib
		10	8-(2,5-Dimethoxyphenyl)-6-methyl-2-(4-methylpent-3-enyl)octa-2,6-dienoic acid, ethyl ester	368	407	2.9		mainlib

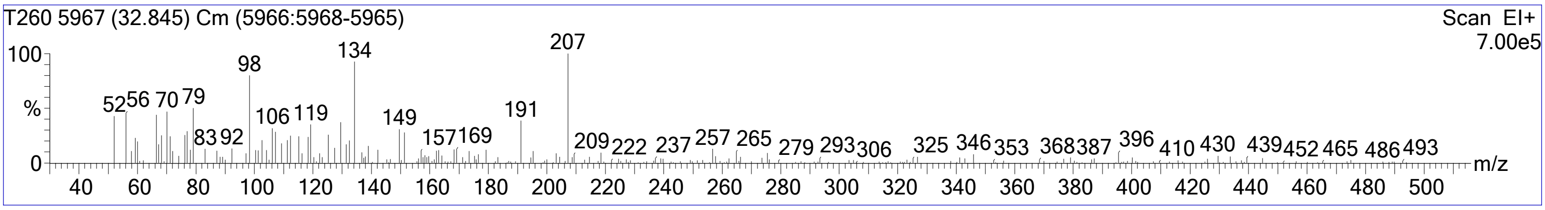

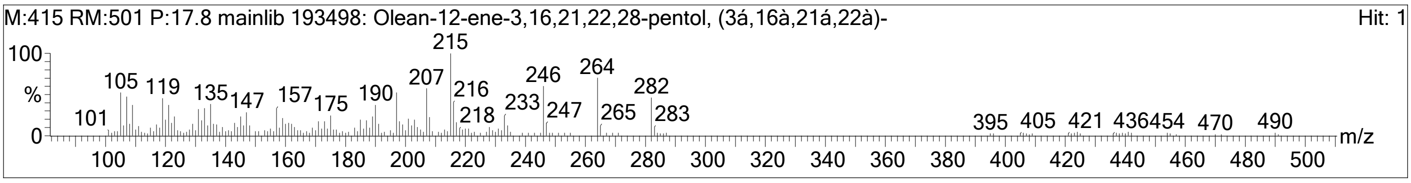

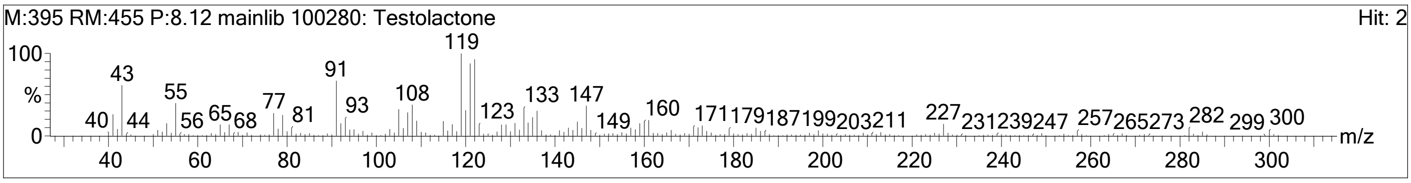

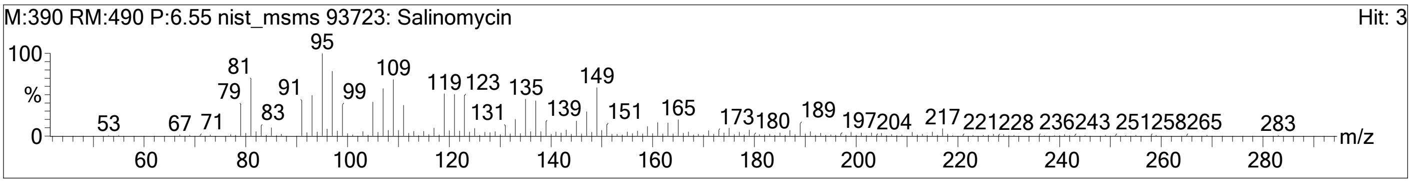

TAMILNADU AGRICULTURAL UNIVERSITY - AGRICULTURAL MICROBIOLOGY

INSTRUMENT: PERKIN ELMER CLARUS SQ8C COLOUMN: DB-5 MS CAPILARY STANDARD NON - POLARCOLOUMN
INJECTION VOL: 1 MICRO LITER DIMENSION: 30Mts, ID: 0.25 mm, FILM: 0.25 IM CARRIER GAS: He
SAMPLE ID : T260

#	RT	Scan	Height	Area	Area %	Norm %
28	34.456	6289	15,484,870	857,798.4	0.735	23.25

Pk #	RT	Hit	Compound Name	Match	R.Match	Prob.	CAS	Library
28	34.456	1	Ethyl iso-allocholate	484	507	33.2		mainlib
		2	4-Piperidineacetic acid, 1-acetyl-5-ethyl-2-[3-(2-hydroxyethyl)-1H-indol-2-yl]-à-methyl-, methyl ester	456	486	9.6	55724-47-5	mainlib
		3	Demecolcine	455	493	9.2	477-30-5	mainlib
		4	1H-2,8a-Methanocyclopenta[a]cyclopropa[e]cyclodecen-11-one, 5,6-bis(acetyloxy)-4-[(acetyloxy)methyl]-1a,2,5,5a,6,9,10,10a-octahydro-5a-hydroxy-1,1,7,9-tetramethyl-, [1aR-(1aà,2à,5á,5aá,6á,8aà,9à,10aà)]-	445	459	6.5	30220-45-2	mainlib
		5	Card-20(22)-enolide, 3-[(6-deoxy-3,4-O-methylenehexopyranos-2-ulos-1-yl)oxy]-5,11,14-trihydroxy-12-oxo-, (3á,5à,11à)-	431	446	4.1	29428-86-2	mainlib
		6	2,4a-Oxymethano-1,2,3,4,4a,4b,5,6,7,8,8a,9-dodecahydrophenanthren-9-one, 8-cyanomethyl-2-methoxy-7-methoxycarbonyl-1,1,7-trimethyl-	421	439	2.9		mainlib
		7	Demecolcine	419	508	9.2	477-30-5	replib
		8	Ibogamine-18-carboxylic acid, 16,17-didehydro-9,17-dihydro-9,20-dihydroxy-12-methoxy-, methyl ester, (20S)-	413	466	2.1	15215-86-8	mainlib
		9	Allopregnane-7à,11à-diol-3,20-dione	412	489	2.1		mainlib
		10	17-(2-Hydroxy-1,5-dimethyl-hex-4-enyl)-4,4,10,13,14-pentamethyl-2,3,4,5,6,7,10,11,12,13,14,15,16,17-tetradecahydro-1H-cyclopenta[a]phenanthrene	411	442	2.0		mainlib

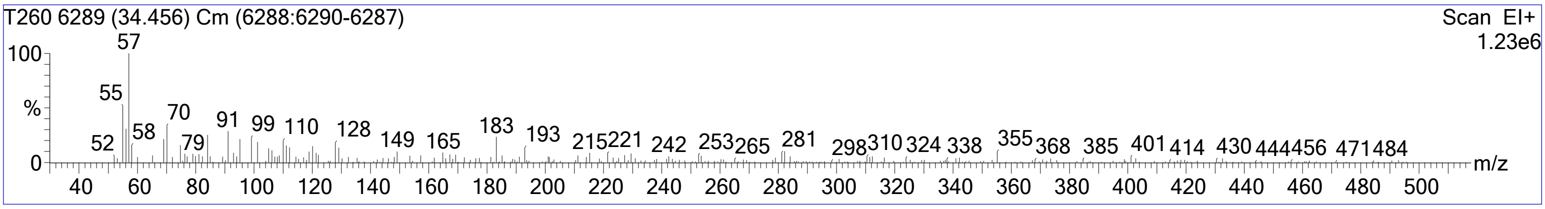

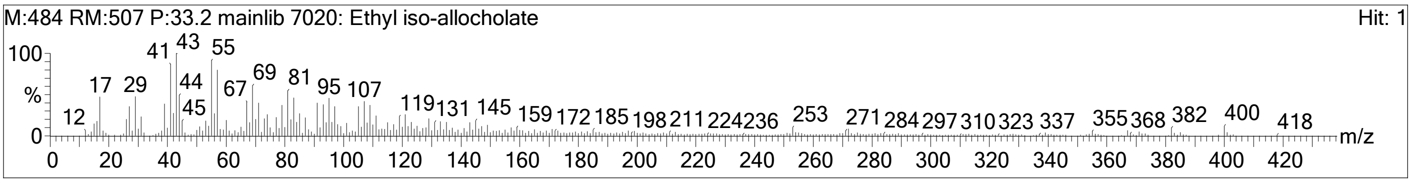

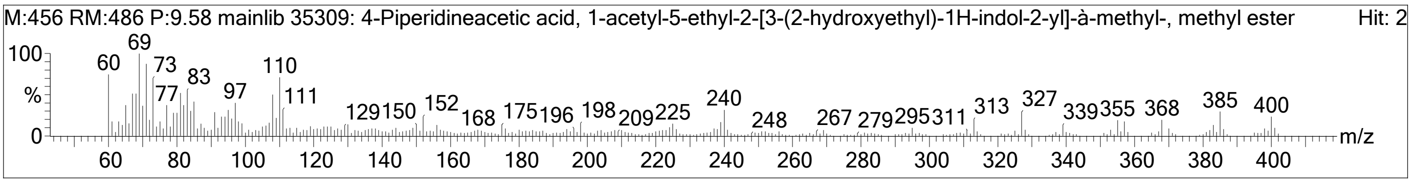

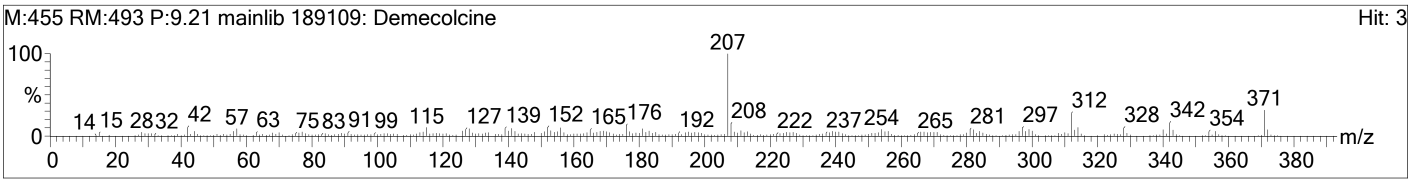

TAMILNADU AGRICULTURAL UNIVERSITY - AGRICULTURAL MICROBIOLOGY

INSTRUMENT: PERKIN ELMER CLARUS SQ8C COLOUMN: DB-5 MS CAPILARY STANDARD NON - POLARCOLOUMN
INJECTION VOL: 1 MICRO LITER DIMENSION: 30Mts, ID: 0.25 mm, FILM: 0.25 IM CARRIER GAS: He
SAMPLE ID : T260

#	RT	Scan	Height	Area	Area %	Norm %
29	34.526	6303	15,164,177	826,650.9	0.708	22.41

Pk #	RT	Hit	Compound Name	Match	R.Match	Prob.	CAS	Library
29	34.526	1	Strychane, 1-acetyl-20à-hydroxy-16-methylene-	439	499	11.0	2111-98-0	mainlib
		2	Dasycarpidan-1-methanol, acetate (ester)	438	494	10.5	55724-48-6	mainlib
		3	Spirost-8-en-11-one, 3-hydroxy-, (3á,5à,14á,20á, 22á,25R)-	424	473	6.6	58072-54-1	mainlib
		4	4-Piperidineacetic acid, 1-acetyl-5-ethyl-2-[3-(2-hydroxyethyl)-1H-indol-2-yl]-à-methyl-, methyl ester	423	448	6.3	55724-47-5	mainlib
		5	Octadecane, 3-ethyl-5-(2-ethylbutyl)-	420	469	5.6	55282-12-7	replib
		6	Pyrrolidine, 1-(1-oxo-2,5-octadecadienyl)-	417	548	4.9	56666-48-9	mainlib
		7	á-Hydroxyquebrachamine	417	465	4.9		mainlib
		8	Octadecane, 1,1'-[1,3-propanediylbis(oxy)]bis-	415	429	4.6	17367-38-3	mainlib
		9	Phorbol 12,13-dihexanoate	407	421	3.4	37558-17-1	mainlib
		10	Cholest-1-eno[2,1-a]naphthalene, 3',4'-dihydro-	404	419	3.0		mainlib

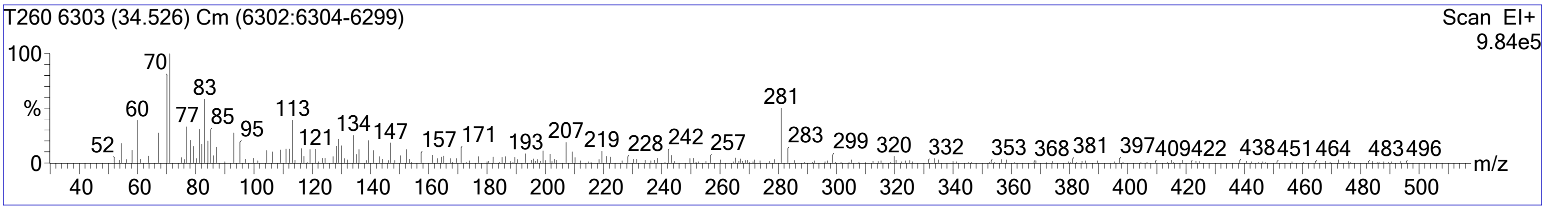

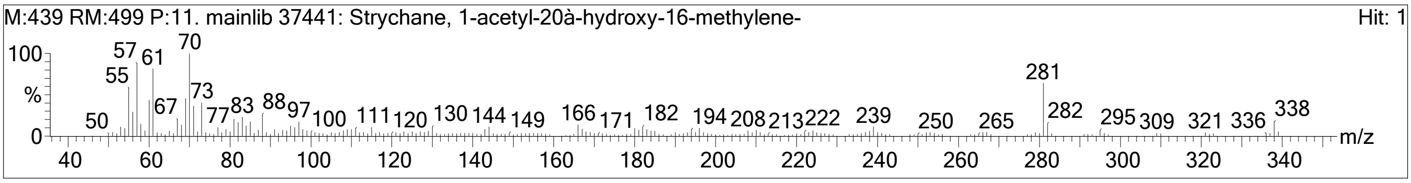

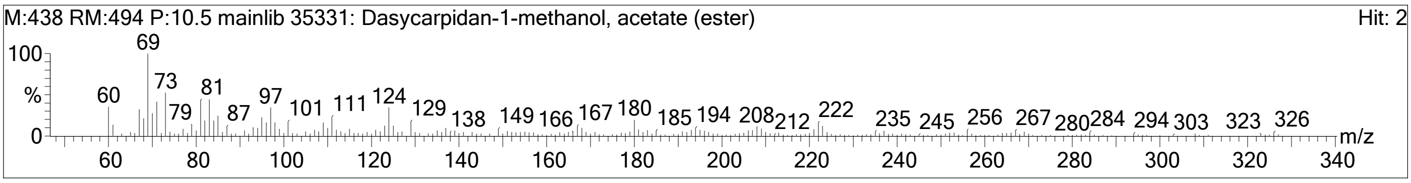

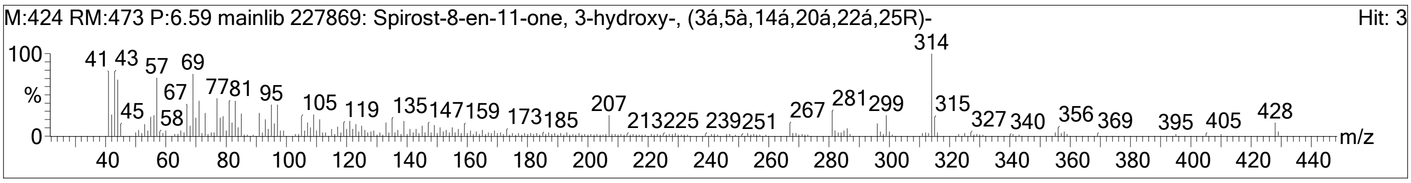

TAMILNADU AGRICULTURAL UNIVERSITY - AGRICULTURAL MICROBIOLOGY

INSTRUMENT: PERKIN ELMER CLARUS SQ8C   COLOUMN: DB-5 MS CAPILARY STANDARD NON - POLARCOLOUMN
INJECTION VOL: 1 MICRO LITER   DIMENSION: 30Mts, ID: 0.25 mm, FILM: 0.25 IM   CARRIER GAS: He
SAMPLE ID : T260

#	RT	Scan	Height	Area	Area %	Norm %
30	34.636	6325	14,621,751	793,083.9	0.680	21.50

Pk #	RT	Hit	Compound Name	Match	R.Match	Prob.	CAS	Library
30	34.636	1	9à-Fluoro-17à-methyl-5à-Androstan-3á,11á,17á-triol	426	494	14.6		mainlib
		2	9-Octadecenoic acid (Z)-, 2,3-dihydroxypropyl ester	424	460	13.5	111-03-5	mainlib
		3	Norgestrel	412	482	9.0	6533-00-2	replib
		4	Elaidic acid, isopropyl ester	404	501	6.7	22147-34-8	mainlib
		5	11-Acetamidooctadecanoic acid	390	425	4.2	80509-30-4	mainlib
		6	1,4-Dioxa-7,14-diazacyclohexadecane-6,15-dione, 7,14-dihexyl-	390	410	4.2	67456-24-0	mainlib
		7	Norgestrel	387	484	9.0	6533-00-2	replib
		8	Norgestrel	387	483	9.0	6533-00-2	replib
		9	2-Tridecyl-5-(acetylamino)tetrahydro-ç-pyrone	385	455	3.4	57633-80-4	mainlib
		10	(14á)3,19-Epoxyandrosta-5,7-diene, 4,4-dimethyl-3-methoxy-17-methylthiomethoxy-	383	408	3.1		mainlib

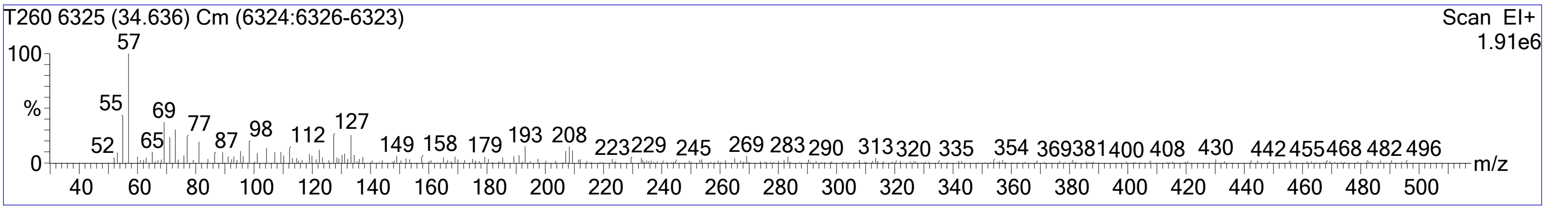

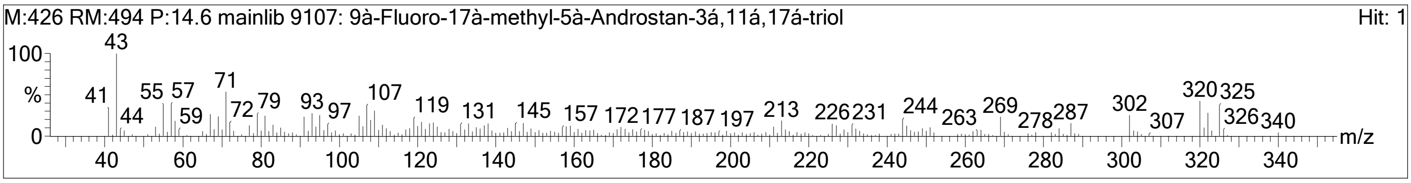

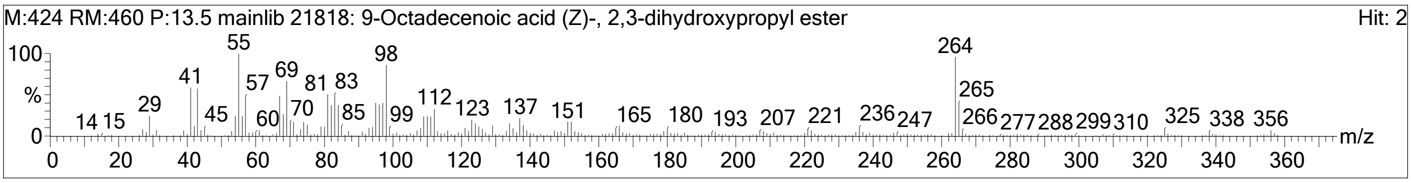

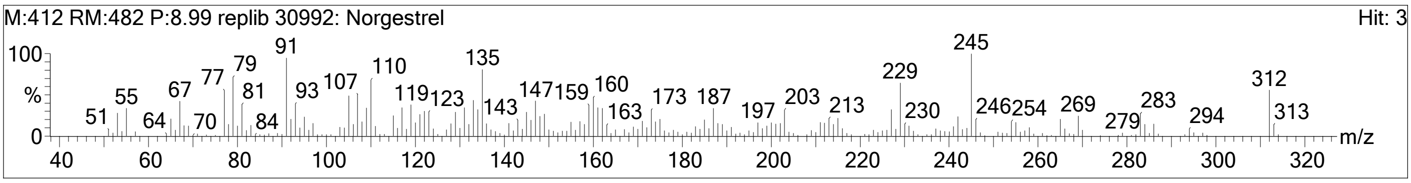

Supplement: S4 Fig — (PDF) [file pone.0219014.s010.pdf]
