# Supplementary material for: Enhancement of disease resistance, growth potential, and photosynthesis in tomato (Solanum lycopersicum) by inoculation with an endophytic actinobacterium, Streptomyces thermocarboxydus strain BPSAC147
Source: PLoS One. 2019 Jul 3;14(7):e0219014. doi: 10.1371/journal.pone.0219014 (PMC6608948; doi:10.1371/journal.pone.0219014)

TAMILNADU AGRICULTURAL UNIVERSITY - AGRICULTURAL MICROBIOLOGY

INSTRUMENT: PERKIN ELMER CLARUS SQ8C   COLOUMN: DB-5 MS CAPILARY STANDARD NON - POLARCOLOUMN
INJECTION VOL: 1 MICRO LITER   DIMENSION: 30Mts, ID: 0.25 mm, FILM: 0.25 IM   CARRIER GAS: He
SAMPLE ID : T360

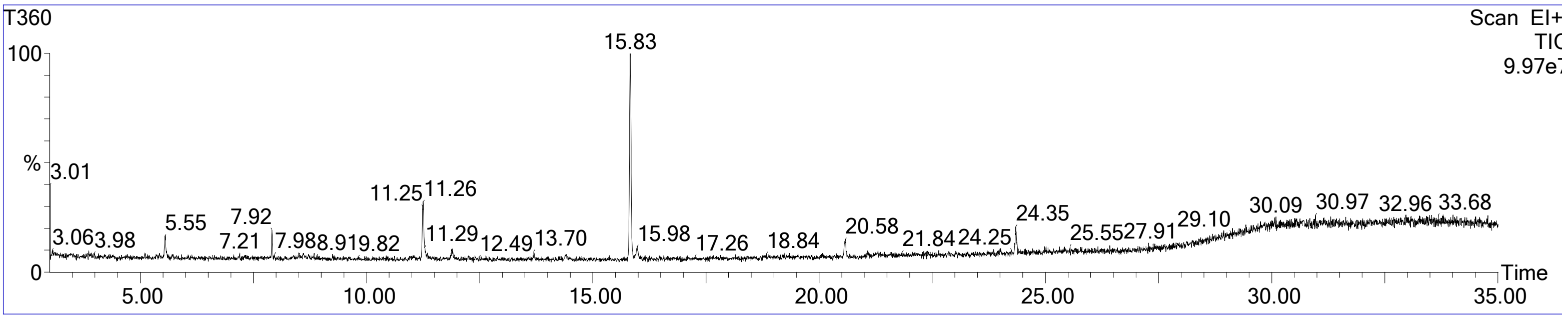

#	RT	Scan	Height	Area	Area %	Norm %
1	3.063	13	4,308,520	166,280.5	0.345	4.31

Pk #	RT	Hit	Compound Name	Match	R.Match	Prob.	CAS	Library
1	3.063	1	2-Myristynoyl pantetheine	439	502	9.2		mainlib
		2	1,8-Di(4-nitrophenylmethyl)-3,6-diazahomoadamantan-9-one	418	563	3.9		mainlib
		3	Propoxyphene	417	516	3.8	469-62-5	replib
		4	2-t-Butyl-5-methyl-10H-acridin-9-one	416	475	3.6		mainlib
		5	3,4-Dimethoxycinnamic acid	408	552	2.7	2316-26-9	replib
		6	Ni(ii)-2,7-bis[2-hydroxy-5,5-dimethyl-4,5-dihydro-1H-pyrrol-4-on-3-yl]-3,6-diazaocta-2,6-dien	408	454	2.7		mainlib
		7	Thiocyanic acid	407	792	2.6	463-56-9	nist_msms
		8	9-Oximino-2,7-diethoxyfluorene	407	532	2.6	327041-58-7	mainlib
		9	Propoxyphene	406	505	3.8	469-62-5	replib
		10	2-Propenoic acid, 3-(3,4-dimethoxyphenyl)-, (E)-	403	501	2.2	14737-89-4	mainlib

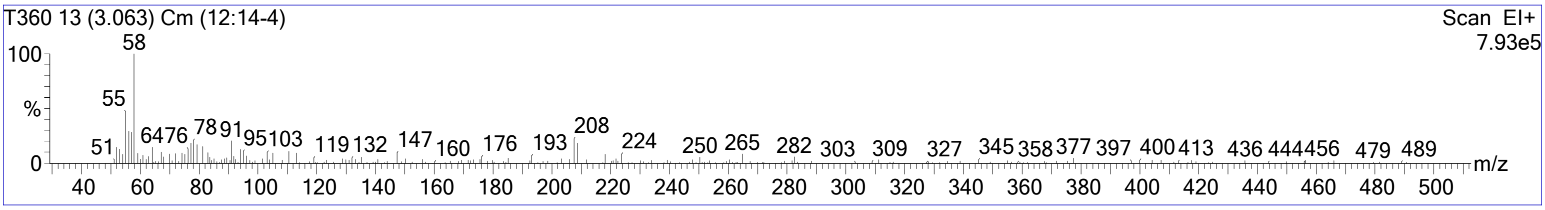

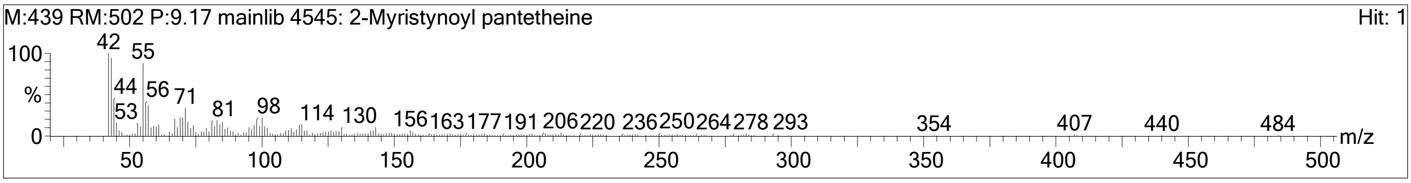

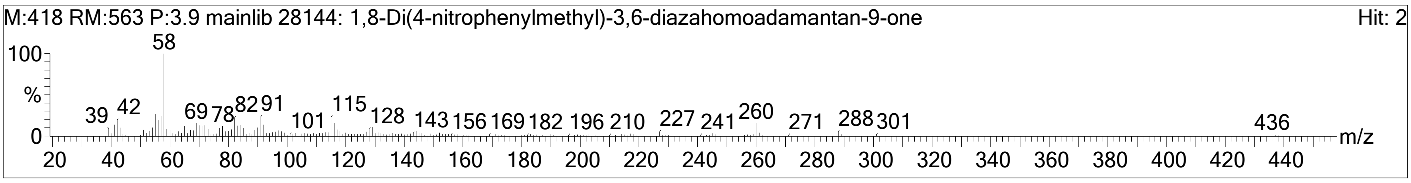

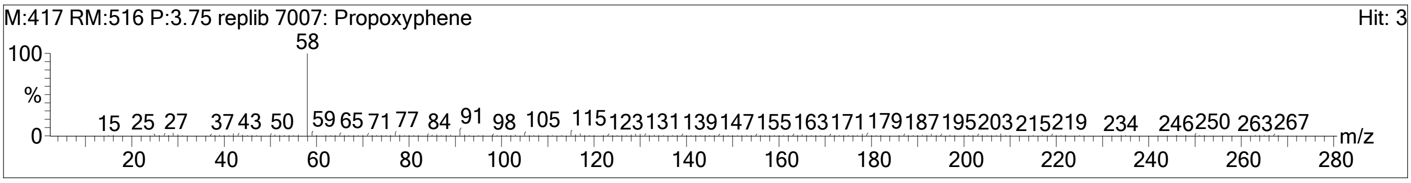

TAMILNADU AGRICULTURAL UNIVERSITY - AGRICULTURAL MICROBIOLOGY

INSTRUMENT: PERKIN ELMER CLARUS SQ8C COLOUMN: DB-5 MS CAPILARY STANDARD NON - POLARCOLOUMN
INJECTION VOL: 1 MICRO LITER DIMENSION: 30Mts, ID: 0.25 mm, FILM: 0.25 IM CARRIER GAS: He
SAMPLE ID : T360

#	RT	Scan	Height	Area	Area %	Norm %
2	3.469	94	2,786,442	223,332.5	0.463	5.78

Pk #	RT	Hit	Compound Name	Match	R.Match	Prob.	CAS	Library
2	3.469	1	Furane-3,4(2H,5H)-dione, 2,2-dimethyl-5-spiro-cyclohexane-, dioxime	391	528	4.1	104116-53-2	mainlib
		2	Thieno[2,3-c]furan-3-carbonitrile, 2-amino-4,6-dihydro-4,4,6,6-tetramethyl-	391	518	4.1	447412-24-0	mainlib
		3	3-Dimethylamino-2-(4-chlorphenyl)-thioacrylamide	388	527	3.6		mainlib
		4	Tetradecanoic acid, (3,3a,4,6a,7,8,9,10,10a,10b-decahydro-3a,8,10a-trihydroxy-2,10-dimethyl-3-oxobenz[e]azulen-5-yl)methyl ester, [3aR-(3aà,6aà,8á,10á,10aá,10bá)]-	386	460	3.3	77573-28-5	mainlib
		5	Strychane, 1-acetyl-20à-hydroxy-16-methylene-	385	463	3.2	2111-98-0	mainlib
		6	2-Oleoylglycerol, 2TMS derivative	381	468	2.7	56554-42-8	mainlib
		7	1,2-Benzisothiazol-3-amine, TBDMS derivative	379	530	2.5		mainlib
		8	d-Mannitol, 1-decylsulfonyl-	376	484	2.2		mainlib
		9	i-Propyl 9-tetradecenoate	373	545	2.0		mainlib
		10	Pyridine-4-carboxylic acid, 2,6-dichloro-, [5-(4-chlorophenyl)-2-thienylmethyl] ester	373	481	2.0		mainlib

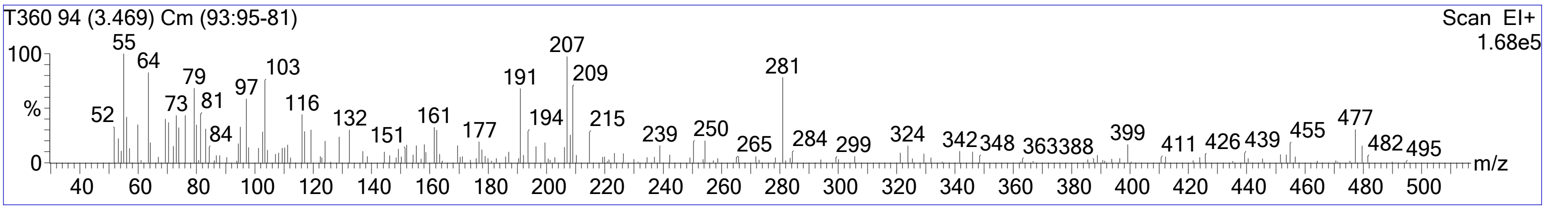

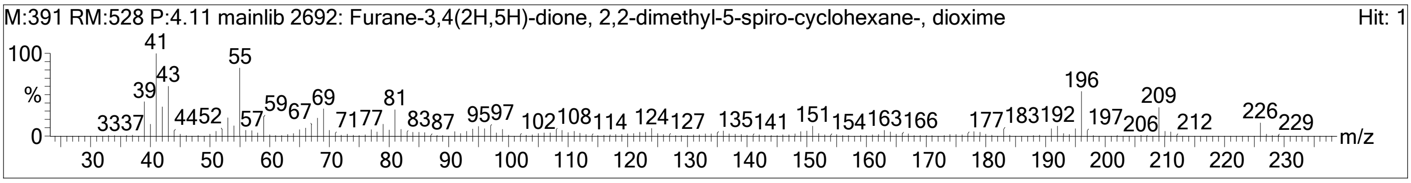

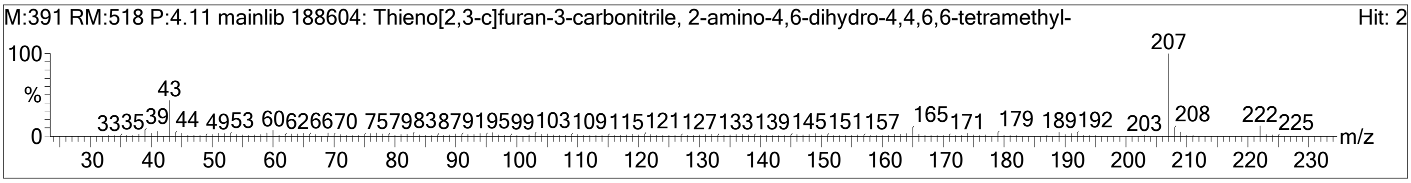

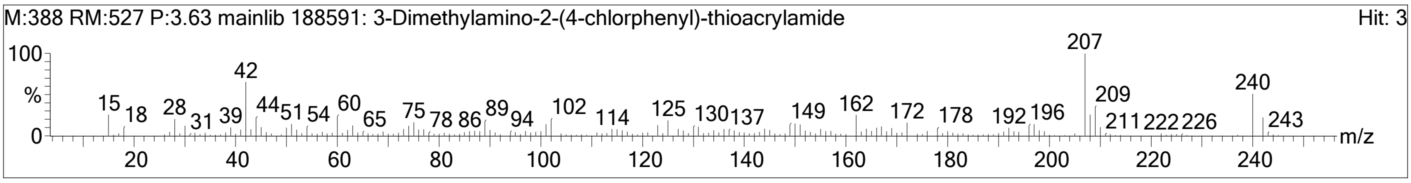

TAMILNADU AGRICULTURAL UNIVERSITY - AGRICULTURAL MICROBIOLOGY

INSTRUMENT: PERKIN ELMER CLARUS SQ8C   COLOUMN: DB-5 MS CAPILARY STANDARD NON - POLARCOLOUMN
INJECTION VOL: 1 MICRO LITER   DIMENSION: 30Mts, ID: 0.25 mm, FILM: 0.25 IM   CARRIER GAS: He
SAMPLE ID : T360

#	RT	Scan	Height	Area	Area %	Norm %
3	3.984	197	3,394,792	142,729.2	0.296	3.70

Pk #	RT	Hit	Compound Name	Match	R.Match	Prob.	CAS	Library
3	3.984	1	Glafenin	366	759	5.7	3820-67-5	nist_msms
		2	Hexadecanoic acid, 2-(octadecyloxy)ethyl ester	355	419	3.9	29899-13-6	mainlib
		3	12-Hydroxyoctadecanethioic acid, S-t-butyl ester	353	423	3.6	58587-08-9	mainlib
		4	(5á,13á) Androst-8-en-3-one, 17-19-diacetoxy-4,4-dimethyl-	351	400	3.3		mainlib
		5	3-Fluoro-3-(2-oxo-2-phenyl-ethylsulfanyl)-2-trifluoromethyl-acrylic acid methyl ester	349	410	3.1	329735-34-4	mainlib
		6	Glafenin	346	569	5.7	3820-67-5	nist_msms
		7	Methyl 2-hydroxy-eicosanoate	342	483	2.3		mainlib
		8	Sebacic acid, di(2,2-dichloroethyl) ester	340	480	2.2		mainlib
		9	1,2-Propanediol, 3-(octadecyloxy)-, diacetate	340	411	2.2	21994-81-0	mainlib
		10	6,8-Dichloro-2-trifluoromethyl-4-quinolinol	339	445	2.1	18706-23-5	mainlib

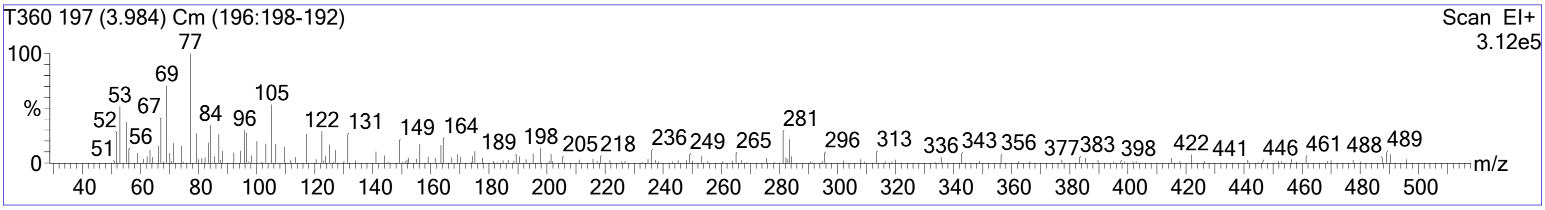

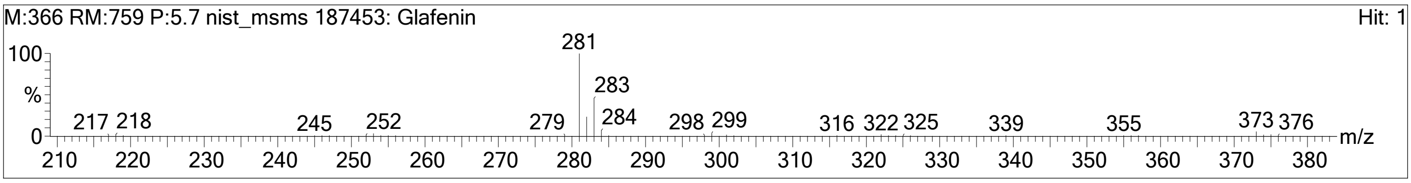

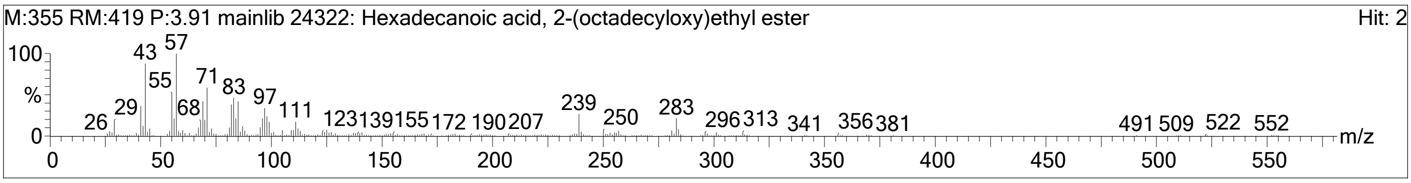

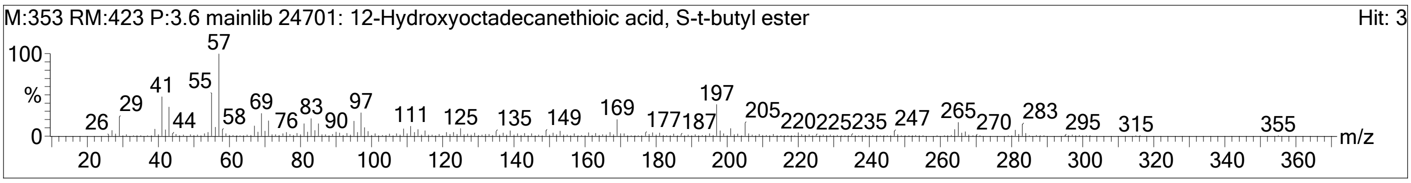

TAMILNADU AGRICULTURAL UNIVERSITY - AGRICULTURAL MICROBIOLOGY

INSTRUMENT: PERKIN ELMER CLARUS SQ8C COLOUMN: DB-5 MS CAPILARY STANDARD NON - POLARCOLOUMN
INJECTION VOL: 1 MICRO LITER DIMENSION: 30Mts, ID: 0.25 mm, FILM: 0.25 IM CARRIER GAS: He
SAMPLE ID : T360

#	RT	Scan	Height	Area	Area %	Norm %
4	4.224	245	3,168,833	194,806.7	0.404	5.05

Pk #	RT	Hit	Compound Name	Match	R.Match	Prob.	CAS	Library
4	4.224	1	13,14-Epoxyursan-3-ol, acetate	379	422	15.5		mainlib
		2	9-Desoxo-9-x-acetoxy-3,8,12-tri-O-acetylingol	371	401	11.6		mainlib
		3	13,14-Epoxyoleanan-3-ol, acetate	367	413	9.8		mainlib
		4	Ethyl iso-allocholate	367	412	9.8		mainlib
		5	6-Amino-5-cyano-4-(5-cyano-2,4-dimethyl-1H-pyrrol-3-yl)-2-methyl-4H-pyran-3-carboxylic acid ethyl ester	358	448	7.1		mainlib
		6	9,10-Secocholesta-5,7,10(19)-triene-3,24,25-triol, (3á,5Z,7E)-	346	450	4.7	40013-87-4	mainlib
		7	à-Cortolone, 4TMS derivative	337	354	3.4	56196-46-4	replib
		8	1H-2,8a-Methanocyclopenta[a]cyclopropa[e]cyclodecen-11-one, 5,6-bis(acetyloxy)-4-[(acetyloxy)methyl]-1a,2,5,5a,6,9,10,10a-octahydro-5a-hydroxy-1,1,7,9-tetramethyl-, [1aR-(1aà,2à,5á,5aá,6á,8aà,9à,10aà)]-	327	358	2.4	30220-45-2	mainlib
		9	Aminoacetamide, N-methyl-N-[4-(1-pyrrolidinyl)-2-butynyl]-N',N'-bis(trifluoroacetyl)	324	390	2.1		mainlib
		10	3à,5à-Cyclo-ergosta-7,9(11),22t-triene-6á-ol	323	401	2.1	118978-72-6	mainlib

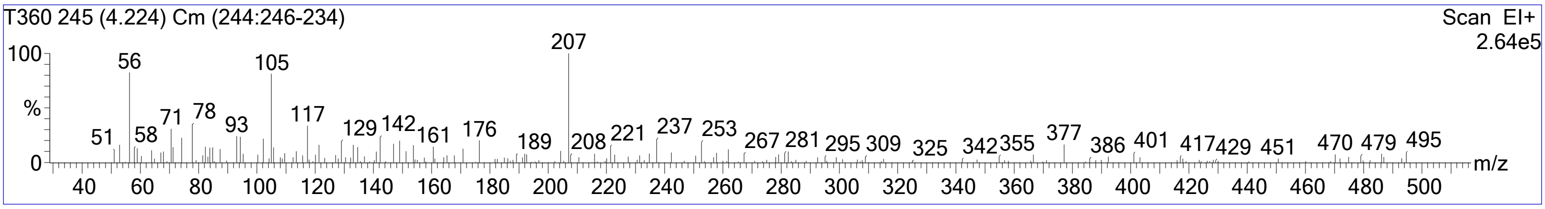

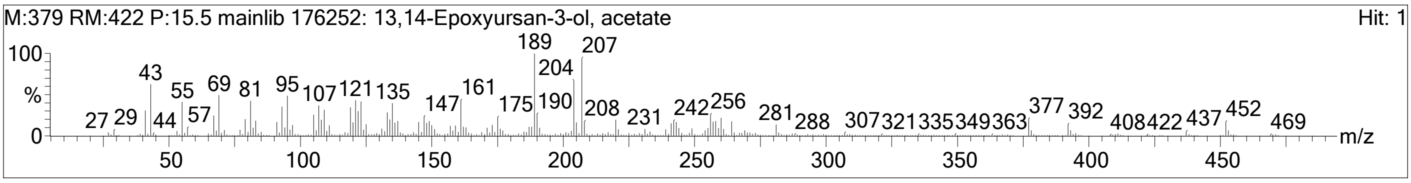

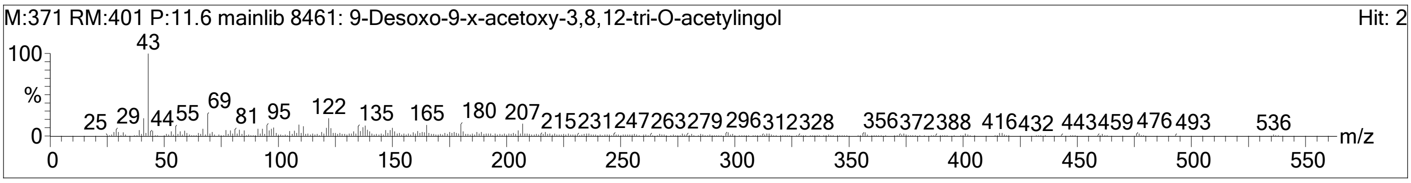

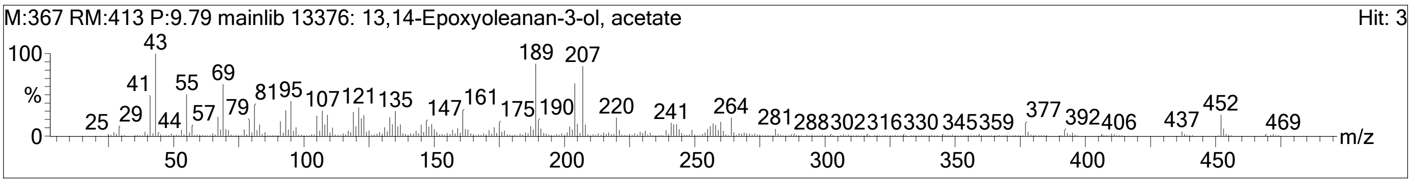

TAMILNADU AGRICULTURAL UNIVERSITY - AGRICULTURAL MICROBIOLOGY

INSTRUMENT: PERKIN ELMER CLARUS SQ8C COLOUMN: DB-5 MS CAPILARY STANDARD NON - POLARCOLOUMN
INJECTION VOL: 1 MICRO LITER DIMENSION: 30Mts, ID: 0.25 mm, FILM: 0.25 IM CARRIER GAS: He
SAMPLE ID : T360

#	RT	Scan	Height	Area	Area %	Norm %
5	4.409	282	2,950,548	126,625.3	0.263	3.28

Pk #	RT	Hit	Compound Name	Match	R.Match	Prob.	CAS	Library
5	4.409	1	1,4-Pentanediamine	382	747	12.3	591-77-5	mainlib
		2	Carbamate, N-(2,6,6-trimethyl-1-[3-oxo-1-butenyl]-2-cyclohexenyl)-, methyl ester	374	444	9.2		mainlib
		3	Strychane, 1-acetyl-20à-hydroxy-16-methylene-	360	422	5.7	2111-98-0	mainlib
		4	L-Carnitine	350	689	4.1	541-15-1	nist_msms
		5	5-Thianonane, 3,7-bis(9-borabicyclo[3.3.1]non-9-yloxy)-	344	376	3.2		mainlib
		6	Bethanechol cation	334	873	2.2	674-38-4	nist_msms
		7	1,2,4-Methenocyclopenta[cd]pentalene-5-carboxaldehyde, 2,2a,3,3,4,7-hexachlorodecahydro-, (1à,2á,2aá,4á,4aá,5á,6aá,6bá,7R*)-	334	370	2.2	7421-93-4	replib
		8	Curan-17-oic acid, 19,20-dihydroxy-, methyl ester, (19S)-	331	428	2.0	2111-90-2	mainlib
		9	8,13-Epoxy-labadan-1,6,7,9-tetraol-11-one, 7-O-acetate(ester)	331	388	2.0	66428-88-4	mainlib
		10	Acetic acid, 3-acetoxy-6-(2-cyanovinyl)-3a,6-dimethyl-2,3,3a,4,5,5a,6,9,9a,9b-decahydro-1H-cyclopenta[a]naphthalen-7-ylmethyl ester	331	371	2.0		mainlib

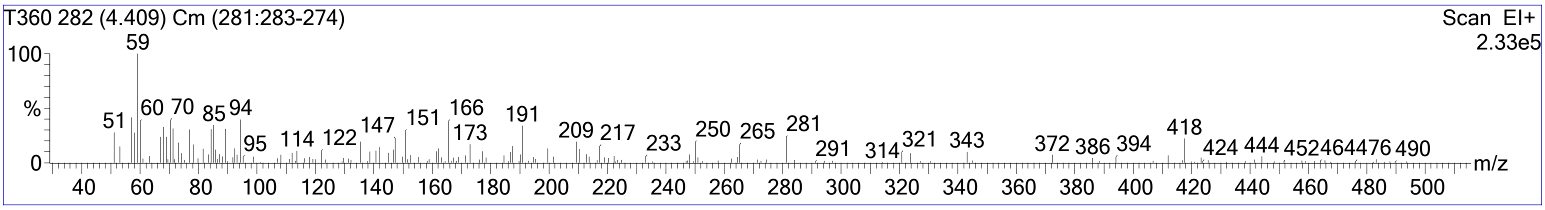

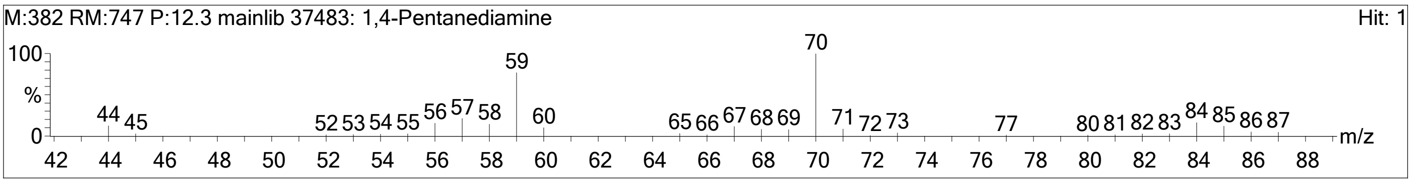

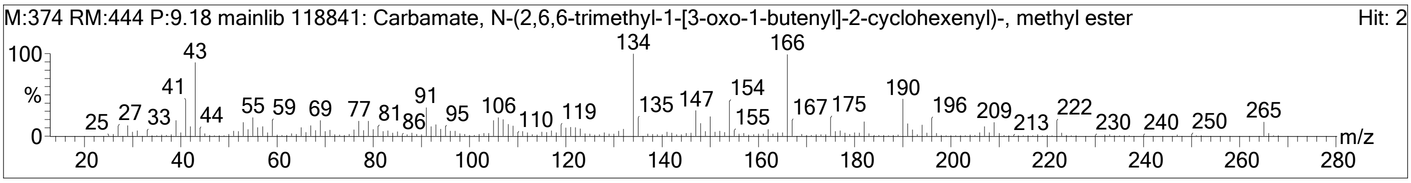

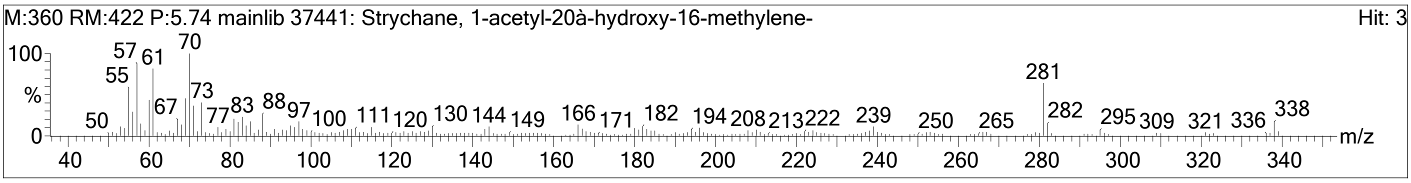

TAMILNADU AGRICULTURAL UNIVERSITY - AGRICULTURAL MICROBIOLOGY

INSTRUMENT: PERKIN ELMER CLARUS SQ8C COLOUMN: DB-5 MS CAPILARY STANDARD NON - POLARCOLOUMN
INJECTION VOL: 1 MICRO LITER DIMENSION: 30Mts, ID: 0.25 mm, FILM: 0.25 IM CARRIER GAS: He
SAMPLE ID : T360

#	RT	Scan	Height	Area	Area %	Norm %
6	5.559	512	10,848,842	452,753.3	0.940	11.73

Pk #	RT	Hit	Compound Name	Match	R.Match	Prob.	CAS	Library
6	5.559	1	Benzaldehyde, 4-methyl-	675	855	26.5	104-87-0	replib
		2	Benzaldehyde, 3-methyl-	667	846	19.8	620-23-5	replib
		3	Benzaldehyde, 4-methyl-	658	845	26.5	104-87-0	replib
		4	Benzaldehyde, 4-methyl-	658	838	26.5	104-87-0	mainlib
		5	Benzaldehyde, 3-methyl-	650	837	19.8	620-23-5	replib
		6	Benzaldehyde, 4-methyl-	650	827	26.5	104-87-0	replib
		7	Bicyclo[4.2.0]octa-1,3,5-trien-7-ol	649	822	10.2	35447-99-5	mainlib
		8	Benzaldehyde, 2-methyl-	643	816	8.0	529-20-4	replib
		9	Benzaldehyde, 4-methyl-	641	818	26.5	104-87-0	replib
		10	Benzaldehyde, 3-methyl-	629	796	19.8	620-23-5	mainlib

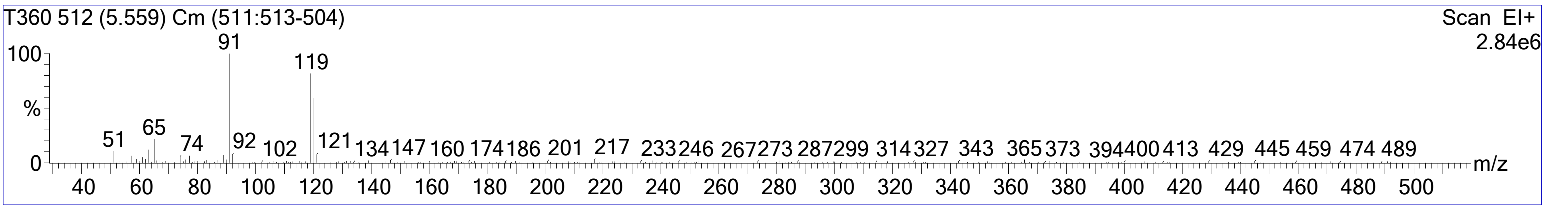

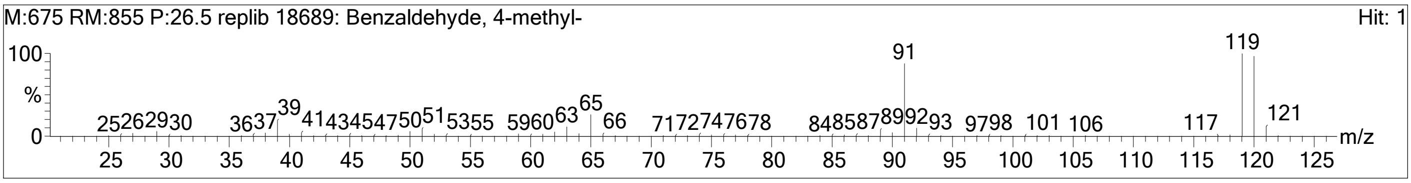

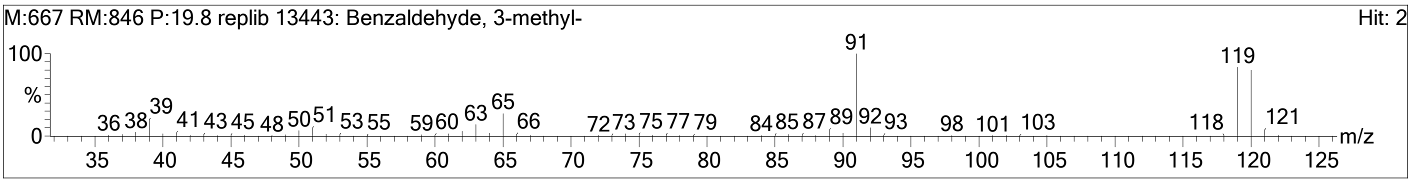

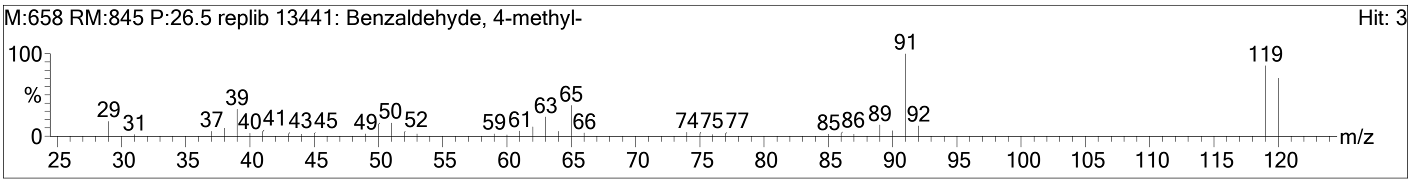

TAMILNADU AGRICULTURAL UNIVERSITY - AGRICULTURAL MICROBIOLOGY

INSTRUMENT: PERKIN ELMER CLARUS SQ8C
COLOUMN: DB-5 MS CAPILARY STANDARD NON - POLARCOLOUMN
INJECTION VOL: 1 MICRO LITER
DIMENSION: 30Mts, ID: 0.25 mm, FILM: 0.25 IM
CARRIER GAS: He
SAMPLE ID : T360

#	RT	Scan	Height	Area	Area %	Norm %
7	7.915	983	13,734,306	346,631.9	0.719	8.98

Pk #	RT	Hit	Compound Name	Match	R.Match	Prob.	CAS	Library
7	7.915	1	Benzene, 1,3-bis(1,1-dimethylethyl)-	719	820	40.6	1014-60-4	replib
		2	Benzene, 1,3-bis(1,1-dimethylethyl)-	719	818	40.6	1014-60-4	replib
		3	Benzene, 1,3-bis(1,1-dimethylethyl)-	711	811	40.6	1014-60-4	mainlib
		4	Benzene, 1,4-dimethyl-2,5-bis(1-methylethyl)-	686	747	10.6	10375-96-9	replib
		5	Benzene, 1,4-bis(1,1-dimethylethyl)-	680	757	8.4	1012-72-2	mainlib
		6	Benzene, 1,4-bis(1,1-dimethylethyl)-	678	765	8.4	1012-72-2	replib
		7	m-Cymene, 5-tert-butyl-	670	740	5.9	29577-19-3	mainlib
		8	Benzene, 1,5-dimethyl-2,4-bis(1-methylethyl)-	669	757	5.7	5186-68-5	mainlib
		9	Precocene I	664	741	4.6	17598-02-6	replib
		10	Benzene, 1,4-dimethyl-2,5-bis(1-methylethyl)-	661	731	10.6	10375-96-9	replib

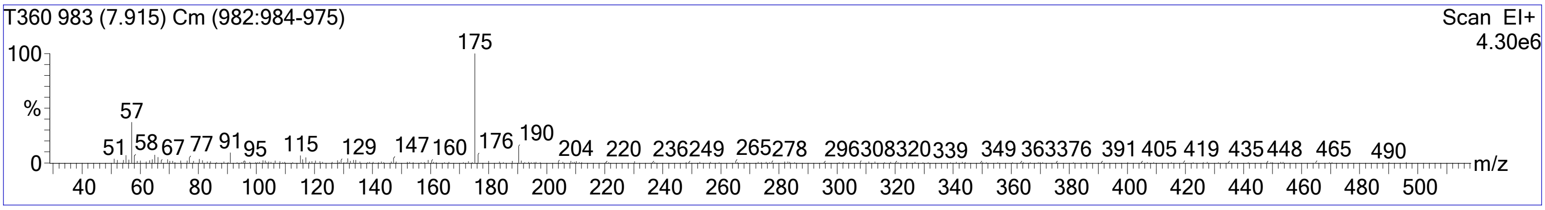

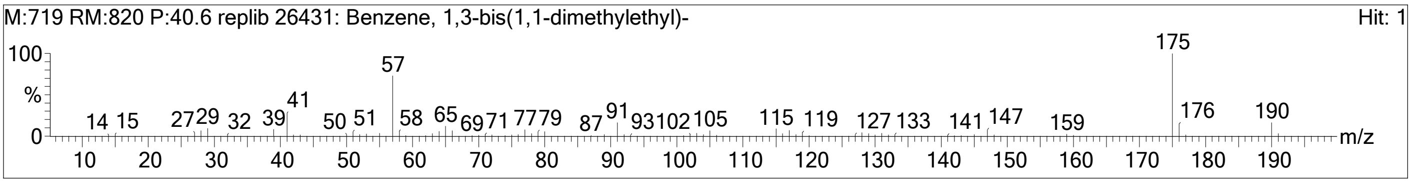

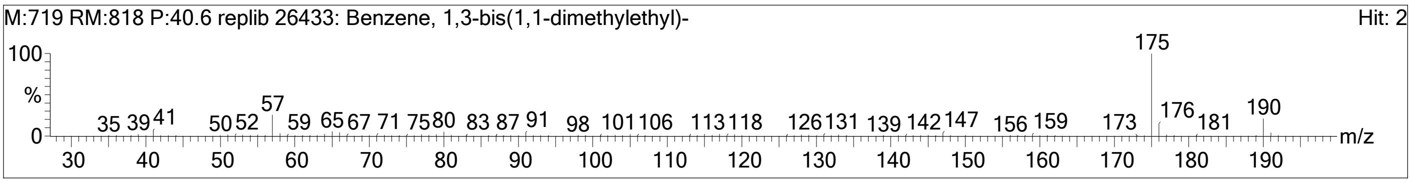

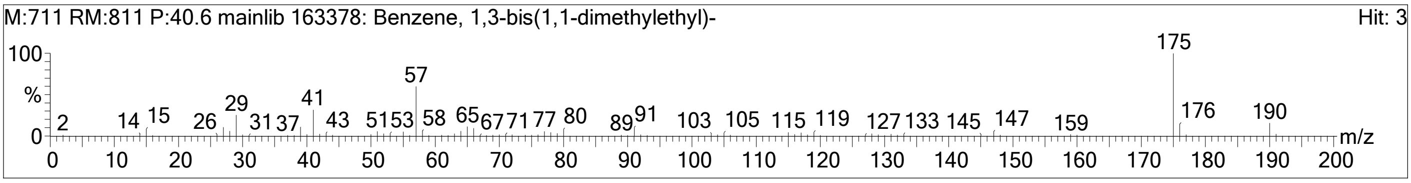

TAMILNADU AGRICULTURAL UNIVERSITY - AGRICULTURAL MICROBIOLOGY

INSTRUMENT: PERKIN ELMER CLARUS SQ8C COLOUMN: DB-5 MS CAPILARY STANDARD NON - POLARCOLOUMN
INJECTION VOL: 1 MICRO LITER DIMENSION: 30Mts, ID: 0.25 mm, FILM: 0.25 IM CARRIER GAS: He
SAMPLE ID : T360

#	RT	Scan	Height	Area	Area %	Norm %
8	11.257	1651	26,787,280	1,240,238.1	2.574	32.12

Pk #	RT	Hit	Compound Name	Match	R.Match	Prob.	CAS	Library
8	11.257	1	Cyclotetradecane	778	897	5.9	295-17-0	replib
		2	1-Dodecanol	775	882	5.2	112-53-8	replib
		3	1-Dodecanol	772	880	5.2	112-53-8	replib
		4	n-Tridecan-1-ol	772	878	4.6	112-70-9	replib
		5	1-Dodecene	771	877	4.4	112-41-4	replib
		6	1-Undecanol	766	898	3.6	112-42-5	mainlib
		7	1-Dodecanol	766	878	5.2	112-53-8	replib
		8	Cyclododecane	763	855	3.2	294-62-2	replib
		9	Cyclododecane	762	871	3.2	294-62-2	replib
		10	1-Dodecanol	762	868	5.2	112-53-8	replib

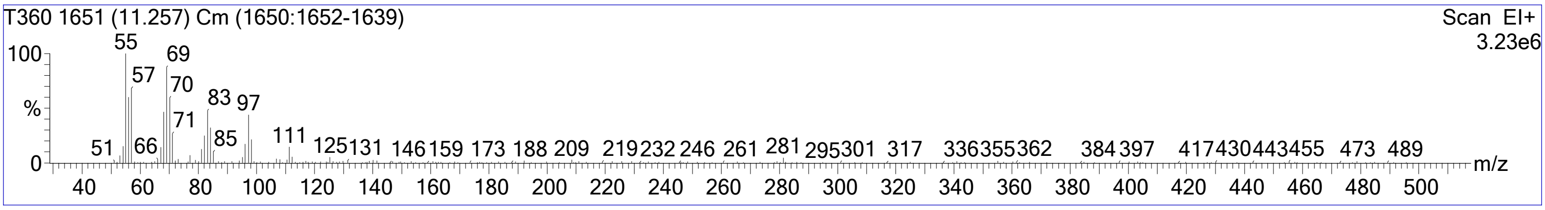

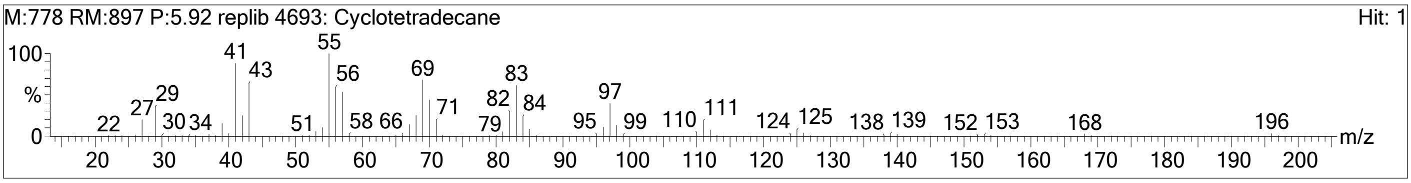

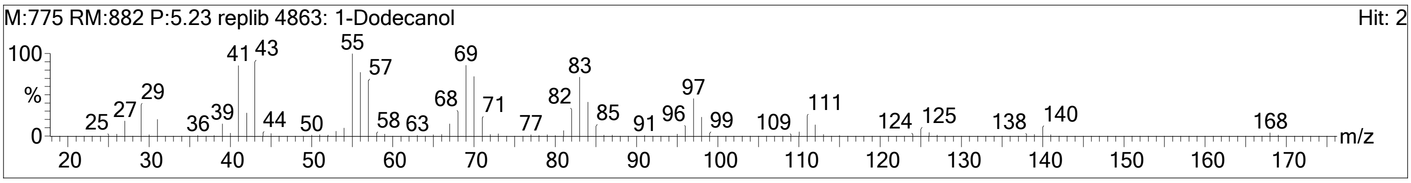

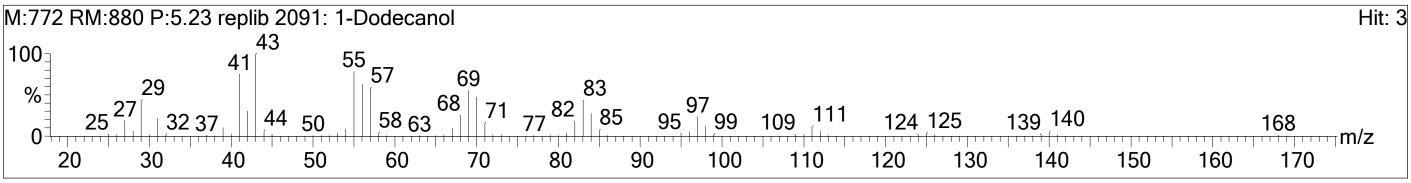

TAMILNADU AGRICULTURAL UNIVERSITY - AGRICULTURAL MICROBIOLOGY

INSTRUMENT: PERKIN ELMER CLARUS SQ8C
INJECTION VOL: 1 MICRO LITER
SAMPLE ID : T360

COLOUMN: DB-5 MS CAPILARY STANDARD NON - POLARCOLOUMN
DIMENSION: 30Mts, ID: 0.25 mm, FILM: 0.25 IM
CARRIER GAS: He

#	RT	Scan	Height	Area	Area %	Norm %
9	11.897	1779	5,271,523	237,998.5	0.494	6.16

Pk #	RT	Hit	Compound Name	Match	R.Match	Prob.	CAS	Library
9	11.897	1	2,4-Di-tert-butylphenol	612	733	27.6	96-76-4	replib
		2	2,4-Di-tert-butylphenol	602	771	27.6	96-76-4	replib
		3	Phenol, 3,5-bis(1,1-dimethylethyl)-	601	738	19.0	1138-52-9	replib
		4	2,4-Di-tert-butylphenol	593	743	27.6	96-76-4	mainlib
		5	Phenol, 3,5-bis(1,1-dimethylethyl)-	585	734	19.0	1138-52-9	replib
		6	Phenol, 2,6-bis(1,1-dimethylethyl)-	583	715	9.8	128-39-2	replib
		7	Phenol, 3,5-bis(1,1-dimethylethyl)-	576	733	19.0	1138-52-9	replib
		8	Phenol, 2,6-bis(1,1-dimethylethyl)-	570	711	9.8	128-39-2	replib
		9	Phenol, 2,6-bis(1,1-dimethylethyl)-	569	708	9.8	128-39-2	mainlib
		10	Phenol, 2,5-bis(1,1-dimethylethyl)-	568	695	5.9	5875-45-6	replib

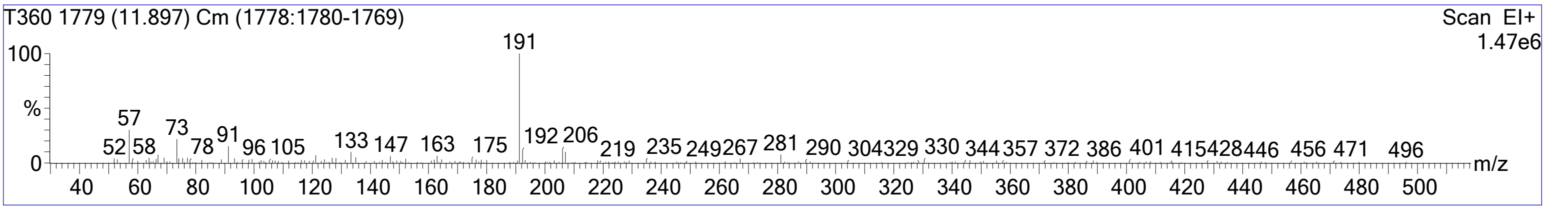

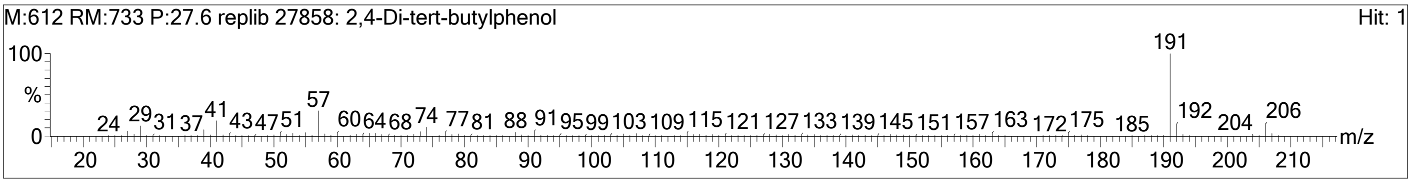

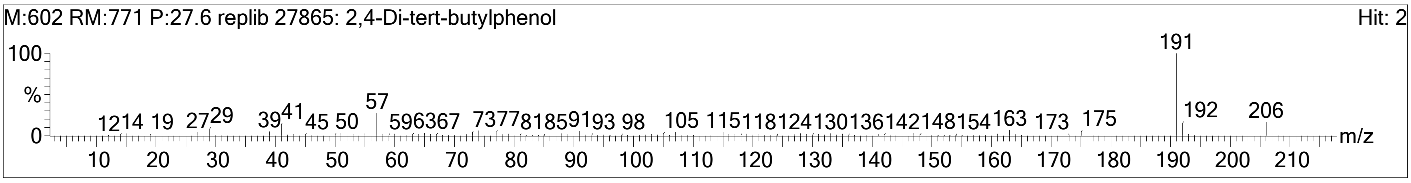

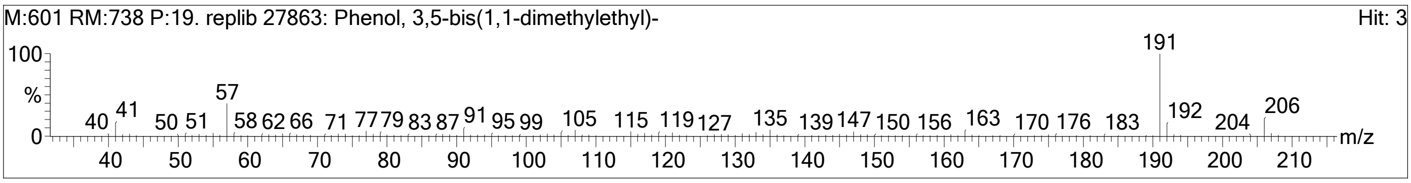

TAMILNADU AGRICULTURAL UNIVERSITY - AGRICULTURAL MICROBIOLOGY

INSTRUMENT: PERKIN ELMER CLARUS SQ8C   COLOUMN: DB-5 MS CAPILARY STANDARD NON - POLARCOLOUMN
INJECTION VOL: 1 MICRO LITER   DIMENSION: 30Mts, ID: 0.25 mm, FILM: 0.25 IM   CARRIER GAS: He
SAMPLE ID : T360

#	RT	Scan	Height	Area	Area %	Norm %
10	13.698	2139	4,552,752	168,251.2	0.349	4.36

Pk #	RT	Hit	Compound Name	Match	R.Match	Prob.	CAS	Library
10	13.698	1	D-Mannitol, 1,2:3,4:5,6-tris-O-(1-methylethylidene)-	418	713	31.1	3969-59-3	replib
		2	D-Mannitol, 1,2:3,4:5,6-tris-O-(1-methylethylidene)-	414	735	31.1	3969-59-3	mainlib
		3	1,2:5,6-Di-O-isopropylidene-à-d-glucofuranose, acetate	393	700	9.5		mainlib
		4	Acetate, [1-(5,5-dimethylperhydro[1,3]dioxolo[4,5-d]oxireno[2,3-b]pyran-1-yl)-1-methylethoxy]methyl ester	387	603	7.4	26289-49-6	mainlib
		5	5-(2-Chloro-6-fluorobenzyl)-6-methyl-2-(2-propynylsulfanyl)-4-pyrimidinol	384	458	6.6		mainlib
		6	à-D-Glucopyranose, 1,2:5,6-di-O-isopropi=ylidene, 3-acetyl-	381	668	5.8		mainlib
		7	4-Estren-17à-methyl-17á-ol-3-one, di-trimethylsilyl	378	417	5.1		mainlib
		8	Butanedioic acid, didodecyl ester	361	478	2.8	5980-15-4	mainlib
		9	Propionic acid, 3,3-bis(3,4-dimethoxyphenyl)-, methyl ester	354	434	2.1		mainlib
		10	3-Chloro-2-nitrobenzyl alcohol, pentafluoropropionate	337	447	1.2		mainlib

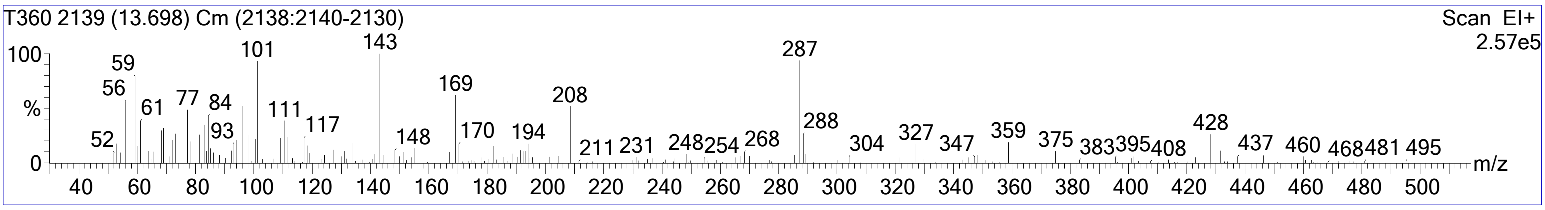

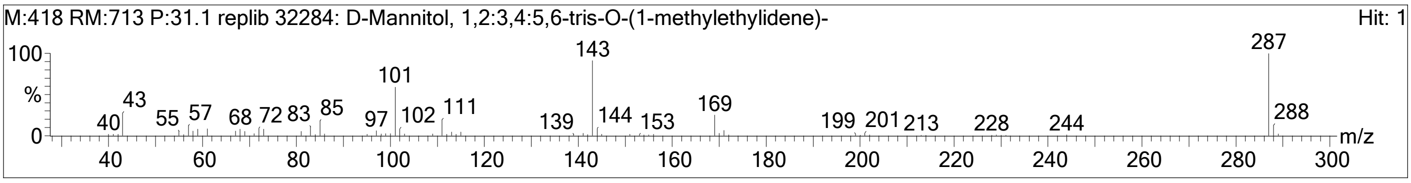

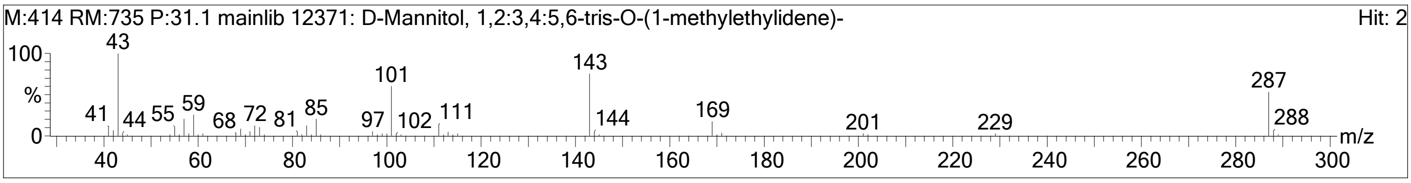

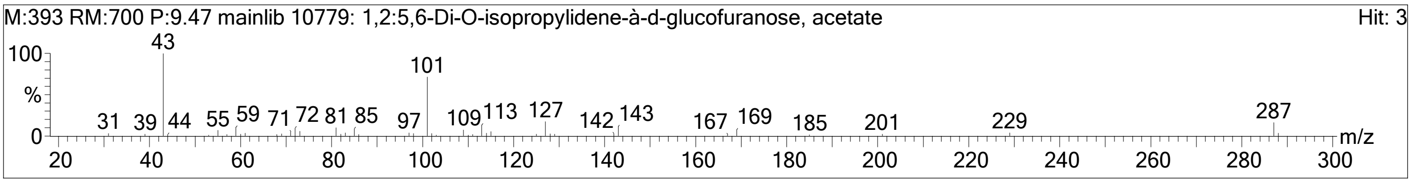

TAMILNADU AGRICULTURAL UNIVERSITY - AGRICULTURAL MICROBIOLOGY

INSTRUMENT: PERKIN ELMER CLARUS SQ8C　　COLOUMN: DB-5 MS CAPILARY STANDARD NON - POLARCOLOUMN
INJECTION VOL: 1 MICRO LITER　　DIMENSION: 30Mts, ID: 0.25 mm, FILM: 0.25 IM　　CARRIER GAS: He
SAMPLE ID : T360

#	RT	Scan	Height	Area	Area %	Norm %
11	14.418	2283	3,146,441	143,301.6	0.297	3.71

Pk #	RT	Hit	Compound Name	Match	R.Match	Prob.	CAS	Library
11	14.418	1	2,7-Diphenyl-1,6-dioxopyridazino[4,5:2',3']pyrrolo[4',5'-d]pyridazine	452	527	12.2	91757-06-1	mainlib
		2	Glafenin	439	870	7.9	3820-67-5	nist_msms
		3	Propanoic acid, 2-(3-acetoxy-4,4,14-trimethylandrost-8-en-17-yl)-	424	447	4.8		mainlib
		4	3,6,9,12-Tetraoxatetradecan-1-ol, 14-[4-(1,1,3,3-tetramethylbutyl)phenoxy]-	423	520	4.6	2315-64-2	mainlib
		5	Glycine, N-[(3à,5á)-24-oxo-3-[(trimethylsilyl)oxy]cholan-24-yl]-, methyl ester	423	498	4.6	57326-15-5	mainlib
		6	Corynan-17-ol, 18,19-didehydro-10-methoxy-, acetate (ester)	416	491	3.5	56053-13-5	mainlib
		7	Glafenin	413	835	7.9	3820-67-5	nist_msms
		8	Octadecane, 1,1'-[1,3-propanediylbis(oxy)]bis-	410	433	2.8	17367-38-3	mainlib
		9	Stearic acid, 3-(octadecyloxy)propyl ester	409	445	2.6	17367-40-7	mainlib
		10	2-Myristynoyl pantetheine	405	477	2.2		mainlib

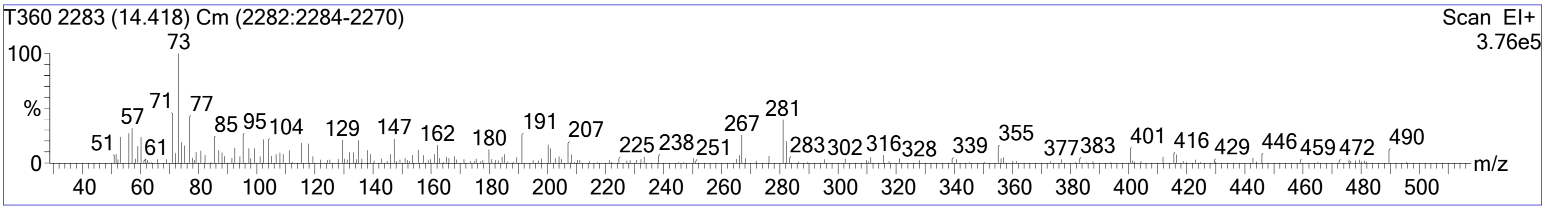

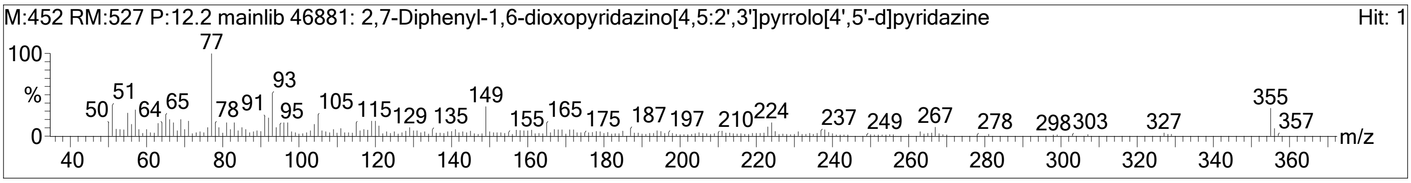

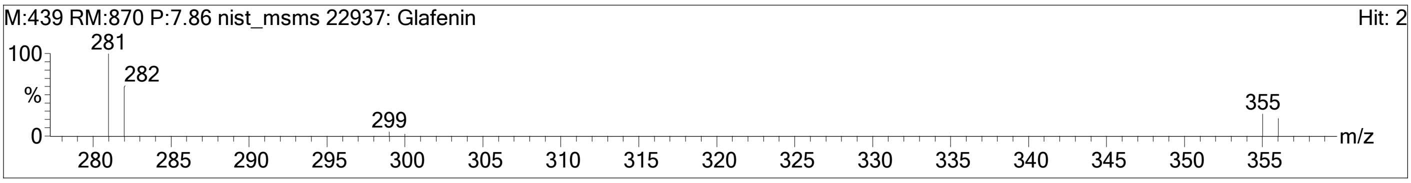

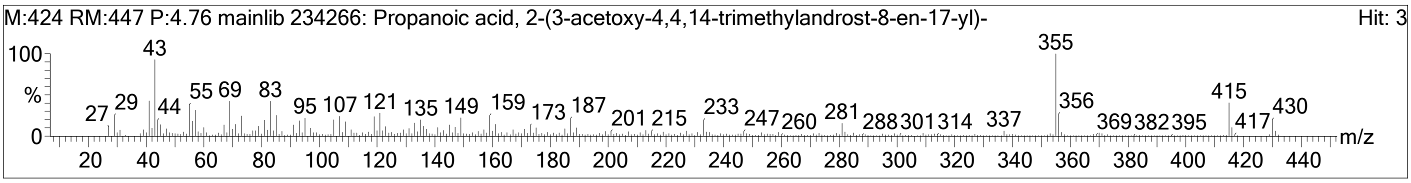

TAMILNADU AGRICULTURAL UNIVERSITY - AGRICULTURAL MICROBIOLOGY

INSTRUMENT: PERKIN ELMER CLARUS SQ8C COLOUMN: DB-5 MS CAPILARY STANDARD NON - POLARCOLOUMN
INJECTION VOL: 1 MICRO LITER DIMENSION: 30Mts, ID: 0.25 mm, FILM: 0.25 IM CARRIER GAS: He
SAMPLE ID : T360

#	RT	Scan	Height	Area	Area %	Norm %
12	15.828	2565	94,334,768	3,861,235.5	8.013	100.00

Pk #	RT	Hit	Compound Name	Match	R.Match	Prob.	CAS	Library
12	15.828	1	Dodecyl acrylate	874	899	46.7	2156-97-0	replib
		2	Dodecyl acrylate	848	876	46.7	2156-97-0	replib
		3	1-Dodecanol	820	872	9.1	112-53-8	replib
		4	Dodecyl acrylate	817	840	46.7	2156-97-0	mainlib
		5	2-Propenoic acid, tridecyl ester	795	824	2.8	3076-04-8	mainlib
		6	2-Propenoic acid, pentadecyl ester	787	811	2.1	43080-23-5	mainlib
		7	Z-10-Tetradecen-1-ol acetate	784	794	1.8		mainlib
		8	1-Dodecanol	779	811	9.1	112-53-8	replib
		9	Cyclododecane	778	802	1.4	294-62-2	replib
		10	2-Butenedioic acid (Z)-, monododecyl ester	776	787	1.3	2424-61-5	mainlib

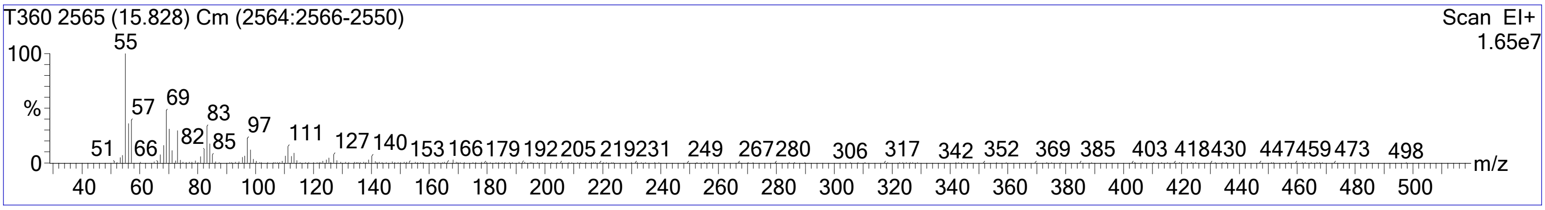

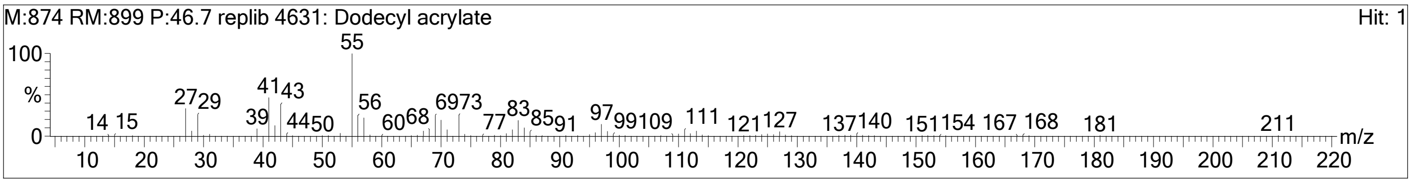

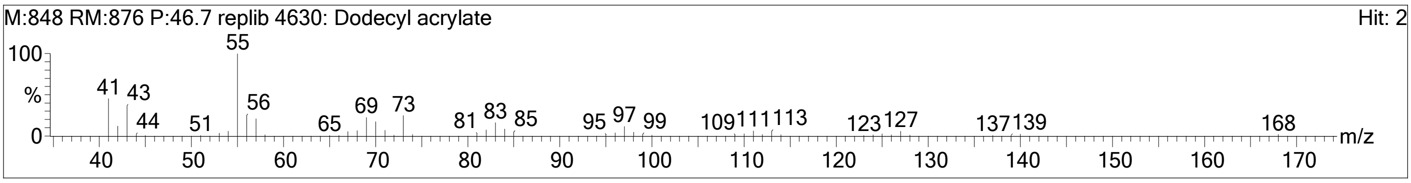

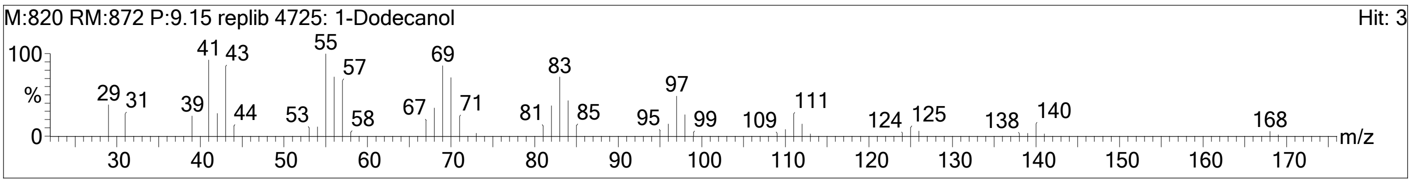

TAMILNADU AGRICULTURAL UNIVERSITY - AGRICULTURAL MICROBIOLOGY

INSTRUMENT: PERKIN ELMER CLARUS SQ8C   COLOUMN: DB-5 MS CAPILARY STANDARD NON - POLARCOLOUMN
INJECTION VOL: 1 MICRO LITER   DIMENSION: 30Mts, ID: 0.25 mm, FILM: 0.25 IM   CARRIER GAS: He
SAMPLE ID : T360

#	RT	Scan	Height	Area	Area %	Norm %
13	15.984	2596	5,659,704	294,735.8	0.612	7.63

Pk #	RT	Hit	Compound Name	Match	R.Match	Prob.	CAS	Library
13	15.984	1	3-Propionyloxypentadecane	487	723	4.2		mainlib
		2	1-Hexadecanol, 2-methyl-	484	572	3.7	2490-48-4	mainlib
		3	Acetic acid, chloro-, octadecyl ester	482	651	3.4	5348-82-3	mainlib
		4	E-10-Dodecen-1-ol propionate	479	648	3.0		mainlib
		5	2-Hexadecanol	479	575	3.0	14852-31-4	replib
		6	Cyclohexane, 1,4-dimethyl-2-octadecyl-	475	513	2.5	55282-02-5	mainlib
		7	1-Heptacosanol	473	634	2.3	2004-39-9	replib
		8	Propanoic acid, decyl ester	472	719	2.3	5454-19-3	mainlib
		9	Octatriacontyl pentafluoropropionate	472	533	2.3		mainlib
		10	5-Octadecenal	468	557	1.9	56554-88-2	mainlib

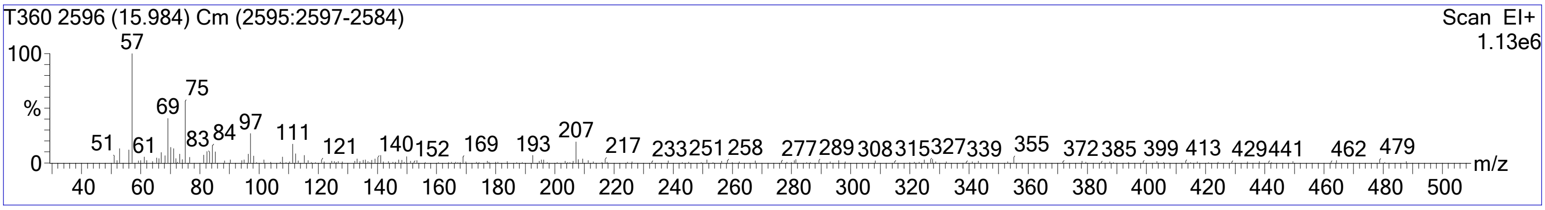

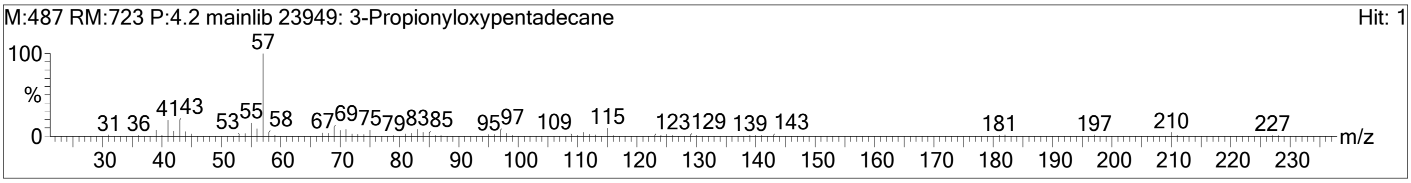

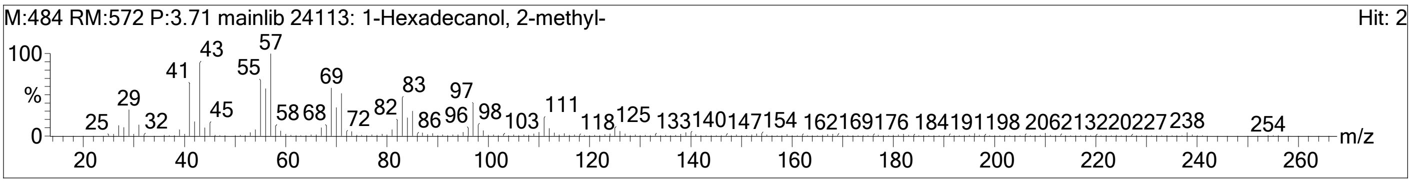

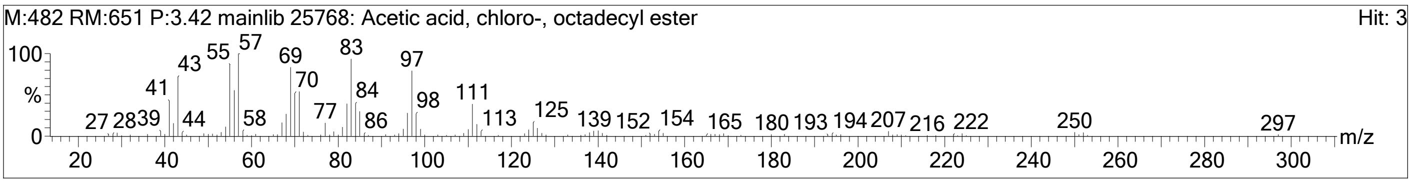

TAMILNADU AGRICULTURAL UNIVERSITY - AGRICULTURAL MICROBIOLOGY

INSTRUMENT: PERKIN ELMER CLARUS SQ8C COLOUMN: DB-5 MS CAPILARY STANDARD NON - POLARCOLOUMN
INJECTION VOL: 1 MICRO LITER DIMENSION: 30Mts, ID: 0.25 mm, FILM: 0.25 IM CARRIER GAS: He
SAMPLE ID : T360

#	RT	Scan	Height	Area	Area %	Norm %
14	20.580	3515	8,788,383	476,165.9	0.988	12.33

Pk #	RT	Hit	Compound Name	Match	R.Match	Prob.	CAS	Library
14	20.580	1	Hexadecanoic acid, methyl ester	604	877	36.8	112-39-0	replib
		2	Hexadecanoic acid, methyl ester	572	811	36.8	112-39-0	replib
		3	Hexadecanoic acid, methyl ester	571	713	36.8	112-39-0	mainlib
		4	Pentadecanoic acid, 14-methyl-, methyl ester	568	755	9.1	5129-60-2	mainlib
		5	Hexadecanoic acid, methyl ester	565	738	36.8	112-39-0	replib
		6	Hexadecanoic acid, methyl ester	558	727	36.8	112-39-0	replib
		7	Methyl tetradecanoate	550	737	4.7	124-10-7	replib
		8	Pentadecanoic acid, 13-methyl-, methyl ester	546	639	4.0	5487-50-3	mainlib
		9	Hexadecanoic acid, methyl ester	540	671	36.8	112-39-0	replib
		10	Pentadecanoic acid, methyl ester	537	721	2.9	7132-64-1	replib

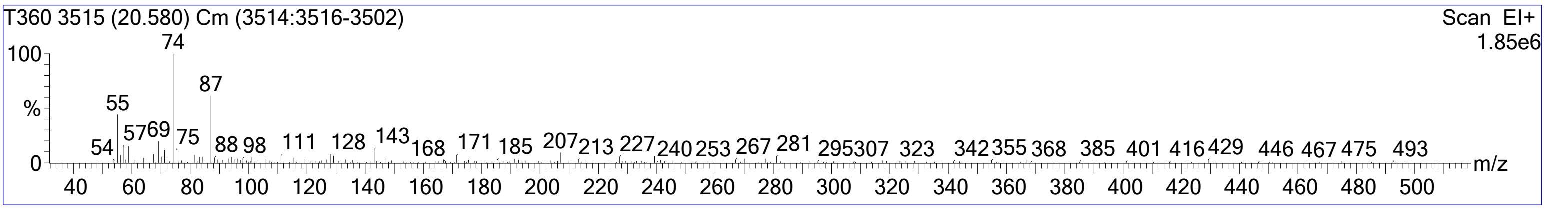

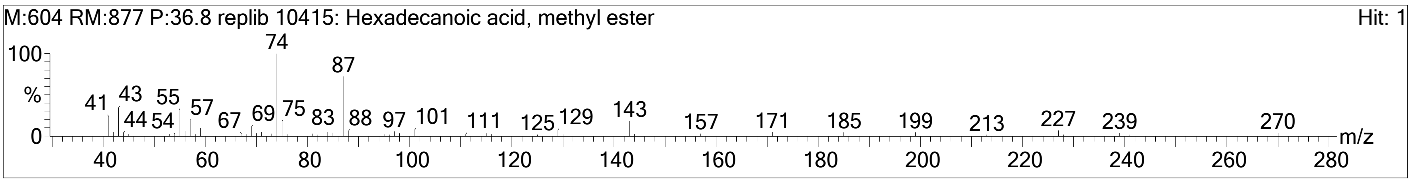

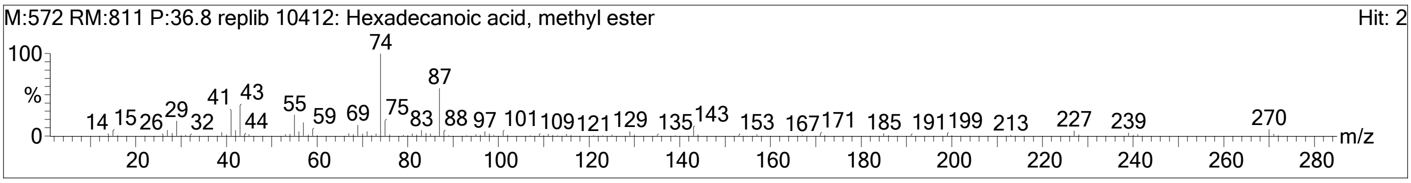

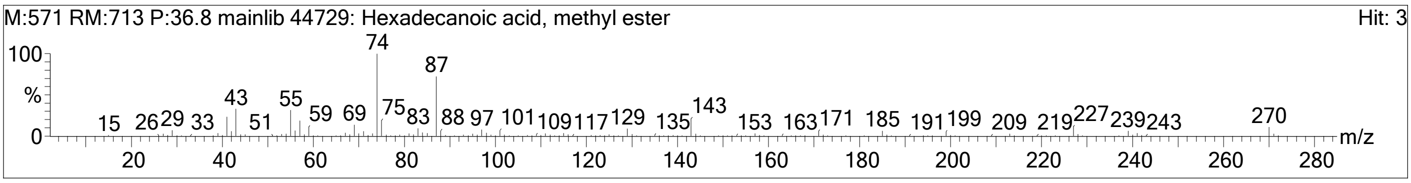

TAMILNADU AGRICULTURAL UNIVERSITY - AGRICULTURAL MICROBIOLOGY

INSTRUMENT: PERKIN ELMER CLARUS SQ8C COLOUMN: DB-5 MS CAPILARY STANDARD NON - POLARCOLOUMN
INJECTION VOL: 1 MICRO LITER DIMENSION: 30Mts, ID: 0.25 mm, FILM: 0.25 IM CARRIER GAS: He
SAMPLE ID : T360

#	RT	Scan	Height	Area	Area %	Norm %
15	21.601	3719	2,456,240	115,275.5	0.239	2.99

Pk #	RT	Hit	Compound Name	Match	R.Match	Prob.	CAS	Library
15	21.601	1	2-Nonadecanone 2,4-dinitrophenylhydrazine	377	420	7.8	28813-61-8	mainlib
		2	Androsta-3,5-dien-3-ol, 17-acetyl-3-O-(t-butyldimethylsilyl)-	375	493	7.2	99647-01-5	replib
		3	Norgestrel, trimethylsilyl ether	360	445	4.4		mainlib
		4	.psi.,.psi.-Carotene, 3,4-didehydro-1,2-dihydro-1-methoxy-	358	385	4.0	5085-16-5	mainlib
		5	(+)-Prostaglandin F2à, 4TMS derivative	358	383	4.0	50669-95-9	mainlib
		6	11à-Hydroxyprogesterone, trimethylsilyl ether, bis(O-methyloxime)	345	359	2.6		mainlib
		7	3-Heptafluorobutyriloxy-3,5,10-pregnatrien-20-one	344	432	2.5		mainlib
		8	(+)-Prostaglandin F2á, 4TMS derivative	342	358	2.3	50669-96-0	mainlib
		9	3,9-Epoxypregnan-14-ol-20-one, 3,11,18-triacetoxy-	340	361	2.1		mainlib
		10	4,4,6a,6b,8a,11,11,14b-Octamethyl-docosahydropicen-3-ol	338	377	2.0		mainlib

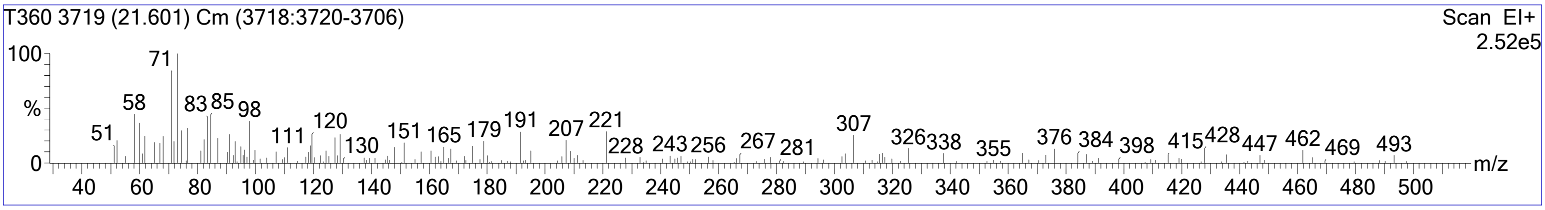

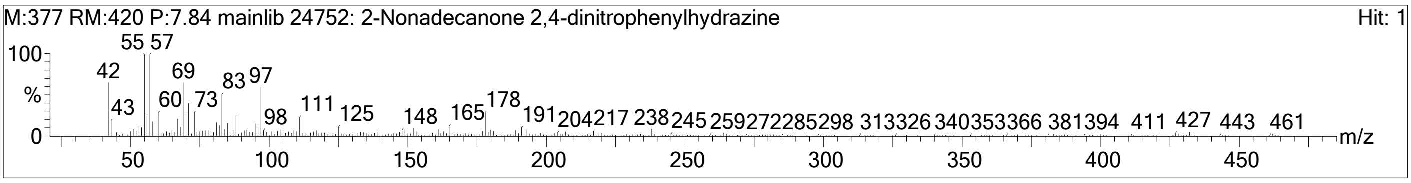

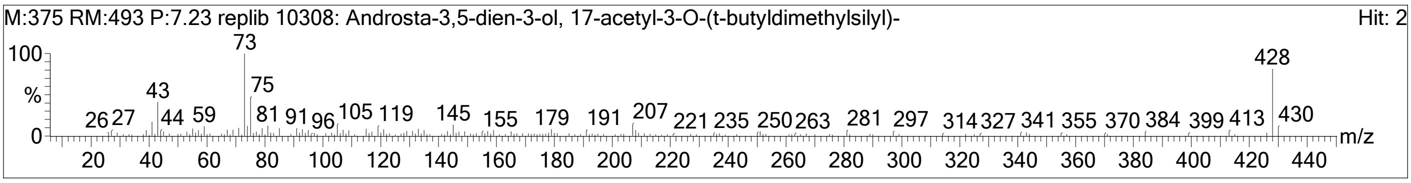

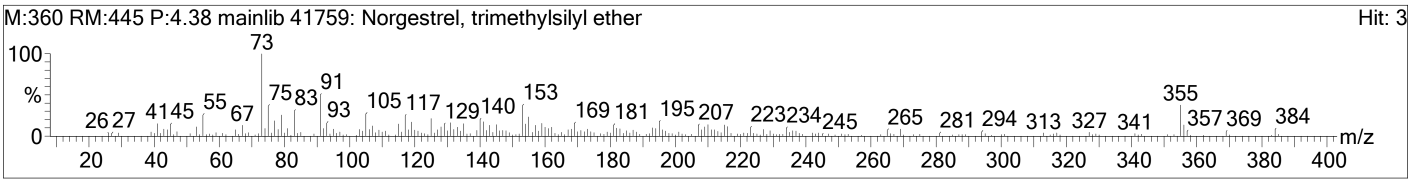

TAMILNADU AGRICULTURAL UNIVERSITY - AGRICULTURAL MICROBIOLOGY

INSTRUMENT: PERKIN ELMER CLARUS SQ8C COLOUMN: DB-5 MS CAPILARY STANDARD NON - POLARCOLOUMN
INJECTION VOL: 1 MICRO LITER DIMENSION: 30Mts, ID: 0.25 mm, FILM: 0.25 IM CARRIER GAS: He
SAMPLE ID : T360

#	RT	Scan	Height	Area	Area %	Norm %
16	24.012	4201	3,762,189	122,604.3	0.254	3.18

Pk #	RT	Hit	Compound Name	Match	R.Match	Prob.	CAS	Library
16	24.012	1	9-Octadecene, 1,1'-[1,2-ethanediylbis(oxy)]bis-, (Z, Z)-	414	454	8.7	17367-13-4	mainlib
		2	Oxiranedodecanoic acid, 3-octyl-, cis-	403	458	5.9	3420-36-8	mainlib
		3	trans-Traumatic acid	401	529	5.5	6402-36-4	mainlib
		4	8-Octadecenal	392	462	4.0	56554-94-0	mainlib
		5	9-Octadecene, 1-[2-(octadecyloxy)ethoxy]-	392	429	4.0	56599-41-8	mainlib
		6	2-Adamantanol, 6,6-ethylenedioxy-	391	512	3.8		mainlib
		7	Octadecanoic acid, 3-hydroxy-, methyl ester	391	484	3.8	2420-36-2	replib
		8	Eicosane, 2-cyclohexyl-	385	445	3.0	4443-56-5	mainlib
		9	Octadecanoic acid, 9-octadecenyl ester, (Z)-	384	417	2.9	17673-50-6	mainlib
		10	1,8,15,22-Tetraaza-2,7,16,21-cyclooctacosanetetrone	384	396	2.9	4238-35-1	mainlib

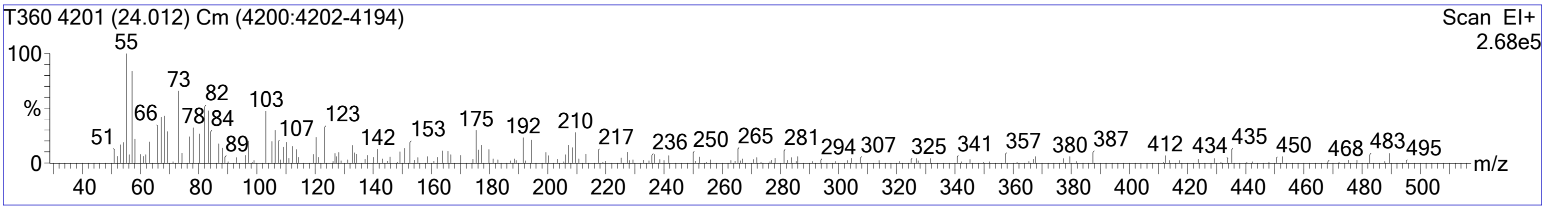

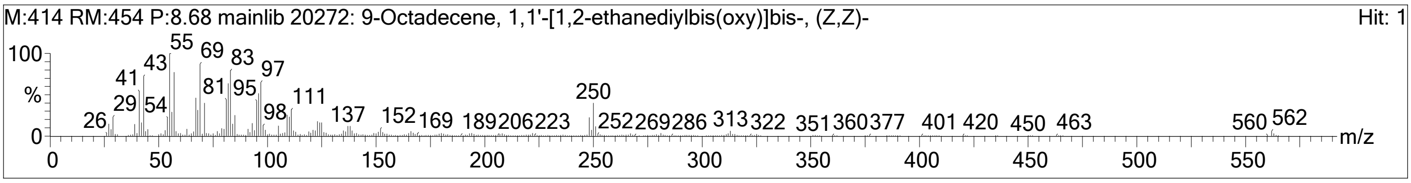

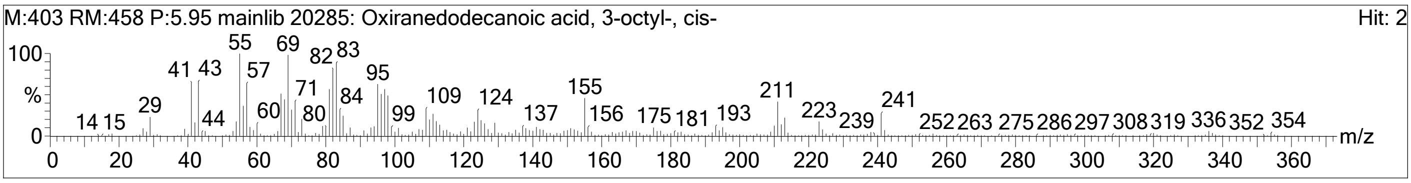

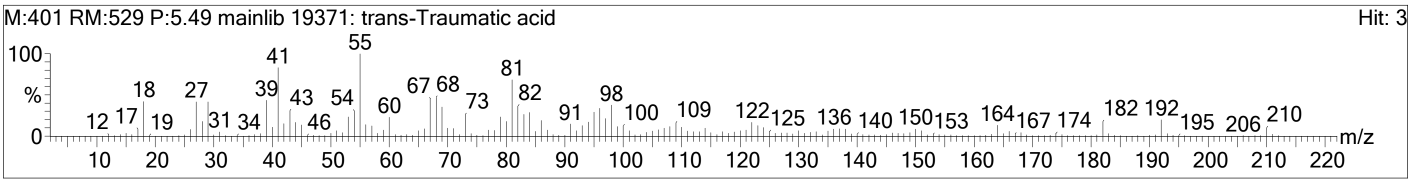

TAMILNADU AGRICULTURAL UNIVERSITY - AGRICULTURAL MICROBIOLOGY

INSTRUMENT: PERKIN ELMER CLARUS SQ8C COLOUMN: DB-5 MS CAPILARY STANDARD NON - POLARCOLOUMN
INJECTION VOL: 1 MICRO LITER DIMENSION: 30Mts, ID: 0.25 mm, FILM: 0.25 IM CARRIER GAS: He
SAMPLE ID : T360

#	RT	Scan	Height	Area	Area %	Norm %
17	24.347	4268	12,905,212	541,816.2	1.124	14.03

Pk #	RT	Hit	Compound Name	Match	R.Match	Prob.	CAS	Library
17	24.347	1	Heptadecanoic acid, 16-methyl-, methyl ester	641	719	30.3	5129-61-3	mainlib
		2	Methyl stearate	637	767	25.6	112-61-8	replib
		3	Methyl stearate	636	748	25.6	112-61-8	replib
		4	Methyl stearate	635	844	25.6	112-61-8	replib
		5	Methyl stearate	634	753	25.6	112-61-8	replib
		6	Methyl stearate	629	727	25.6	112-61-8	mainlib
		7	Methyl stearate	628	799	25.6	112-61-8	replib
		8	Heptadecanoic acid, 16-methyl-, methyl ester	616	704	30.3	5129-61-3	replib
		9	Heptadecanoic acid, 16-methyl-, methyl ester	613	744	30.3	5129-61-3	replib
		10	Heptadecanoic acid, 15-methyl-, methyl ester	594	657	5.7	54833-55-5	mainlib

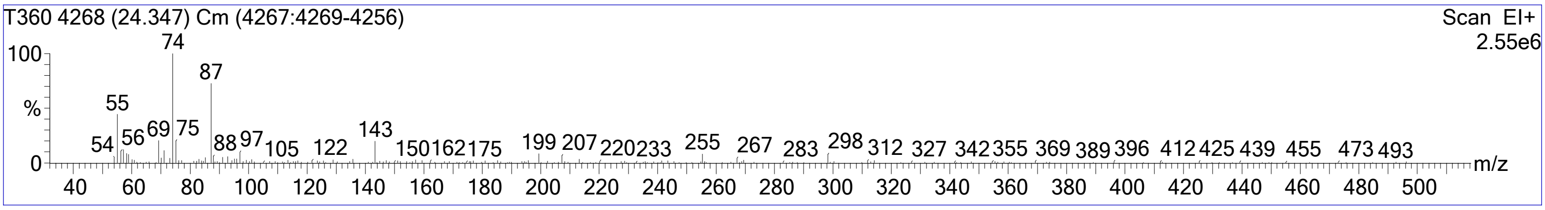

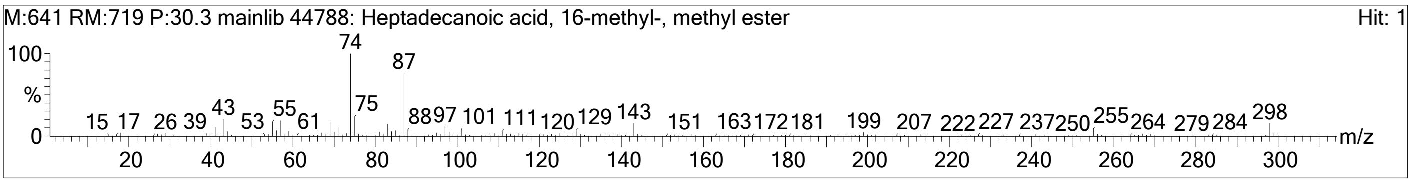

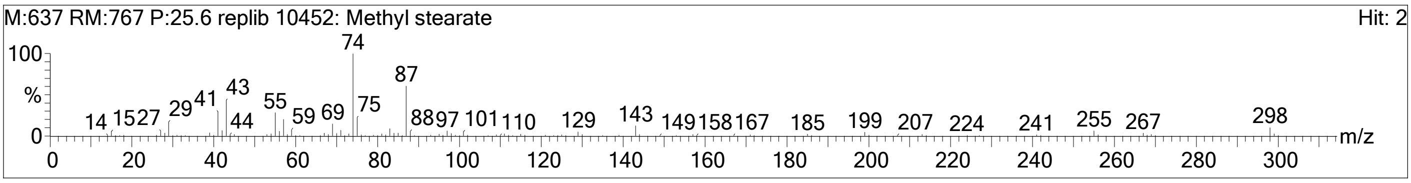

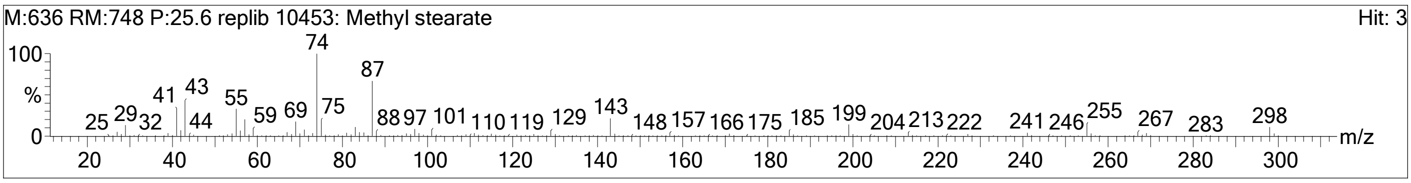

TAMILNADU AGRICULTURAL UNIVERSITY - AGRICULTURAL MICROBIOLOGY

INSTRUMENT: PERKIN ELMER CLARUS SQ8C COLOUMN: DB-5 MS CAPILARY STANDARD NON - POLARCOLOUMN
INJECTION VOL: 1 MICRO LITER DIMENSION: 30Mts, ID: 0.25 mm, FILM: 0.25 IM CARRIER GAS: He
SAMPLE ID : T360

#	RT	Scan	Height	Area	Area %	Norm %
18	27.958	4990	2,718,104	127,913.2	0.265	3.31

Pk #	RT	Hit	Compound Name	Match	R.Match	Prob.	CAS	Library
18	27.958	1	(4,4-Diphenyl-butyl)-(3-phenyl-piperidin-4-yl)-amine	412	465	9.8		mainlib
		2	2-(2-Azepan-1-yl-2-oxoethyl)-1-hydroxy-1-phenyl-octahydro-pyrido[1,2-a]azepin-4-one	404	483	7.3		mainlib
		3	Isodemecolcine, N-desmethyl-	390	442	4.6	102419-92-1	replib
		4	Morphinan-4,5-epoxy-3,6-di-ol, 6-[7-nitrobenzofurazan-4-yl]amino-	388	459	4.2		mainlib
		5	6,19-Cycloandrostane-3,7-diol, 3á-methoxy-	375	439	2.7		mainlib
		6	Stigmastane-3,6-dione, (5à)-	374	455	2.6	22149-69-5	mainlib
		7	5,6,7,8,9,10-Hexahydro-9-methyl-spiro[2H-1,3-benzoxazine-4,1'-cyclohexane]-2-thione	370	457	2.2		mainlib
		8	Pyrrolo[1,2-c][1,3,2]diazaphosphorine-4-carbonitrile, 1,5,6,7-tetrahydro-1-(methylthio)-3-(4-morpholinyl)-, 1-sulfide	367	452	2.0		mainlib
		9	EPPS	367	445	2.0	16052-06-5	mainlib
		10	Docosanedioic acid, dimethyl ester	367	417	2.0	22399-98-0	replib

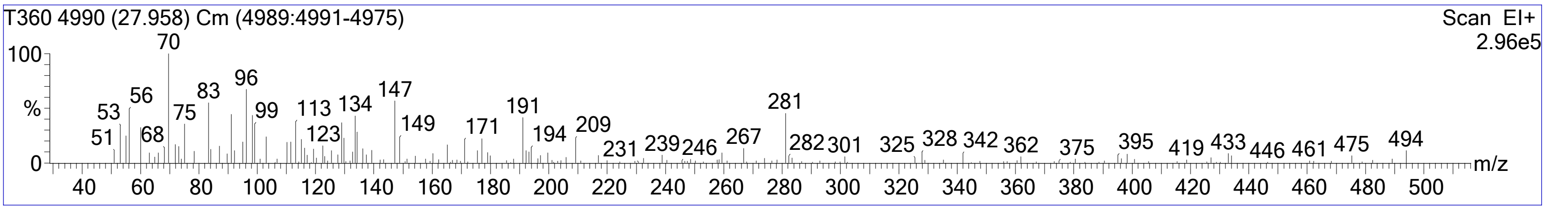

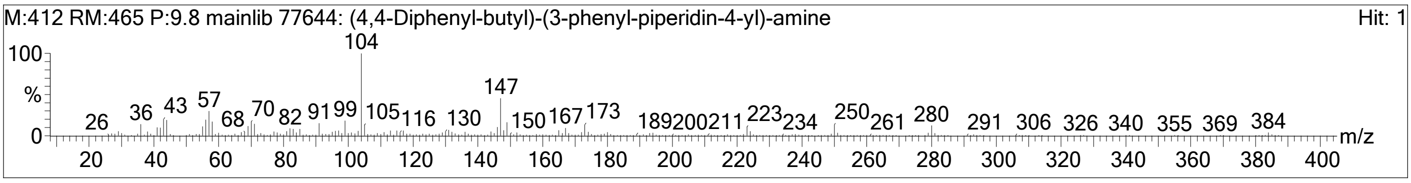

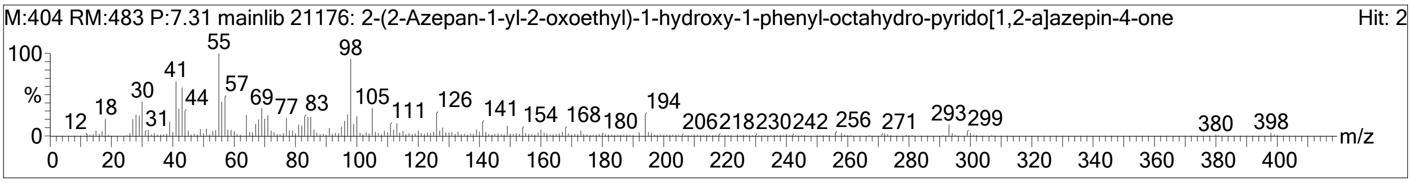

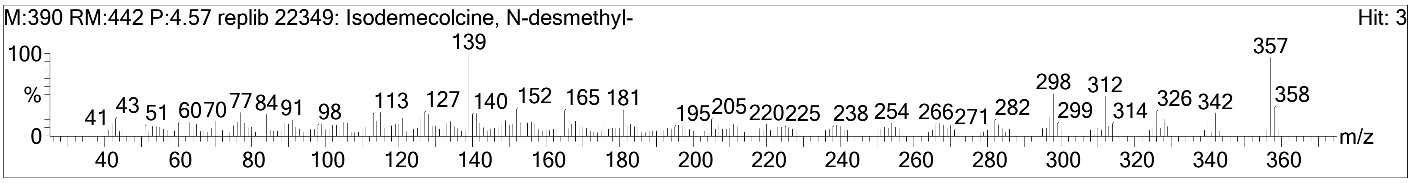

TAMILNADU AGRICULTURAL UNIVERSITY - AGRICULTURAL MICROBIOLOGY

INSTRUMENT: PERKIN ELMER CLARUS SQ8C COLOUMN: DB-5 MS CAPILARY STANDARD NON - POLARCOLOUMN
INJECTION VOL: 1 MICRO LITER DIMENSION: 30Mts, ID: 0.25 mm, FILM: 0.25 IM CARRIER GAS: He
SAMPLE ID : T360

#	RT	Scan	Height	Area	Area %	Norm %
19	28.553	5109	2,833,343	127,570.5	0.265	3.30

Pk #	RT	Hit	Compound Name	Match	R.Match	Prob.	CAS	Library
19	28.553	1	Octadecane, 1-chloro-	427	544	8.3	3386-33-2	replib
		2	1-Chloroeicosane	417	499	5.9	42217-02-7	mainlib
		3	14-Methyl-14-(3-oxobutyryloxy)-hexadec-15-enoic acid, methyl ester	412	479	4.7		mainlib
		4	Perhydro-2-oxo-3-stearoylfuran	410	450	4.4	62972-55-8	mainlib
		5	1,18-Nonadecadien-7,10-dione	409	493	4.2	100594-89-6	mainlib
		6	Ethanol, 2-(octadecyloxy)-	409	467	4.2	2136-72-3	mainlib
		7	Octadecane, 3-ethyl-5-(2-ethylbutyl)-	408	430	4.0	55282-12-7	mainlib
		8	2-Hydroxy-1,1,10-trimethyl-6,9-epidioxydecalin	406	514	3.7	108511-85-9	mainlib
		9	1,2-15,16-Diepoxyhexadecane	402	505	3.1		mainlib
		10	Behenyl chloride	402	485	3.1	42217-03-8	mainlib

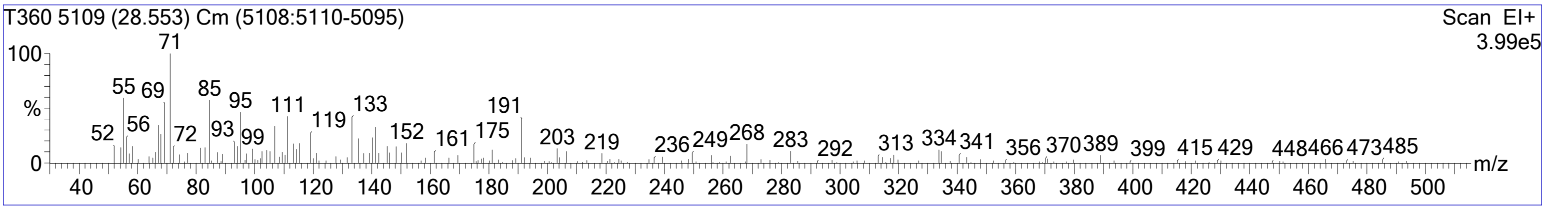

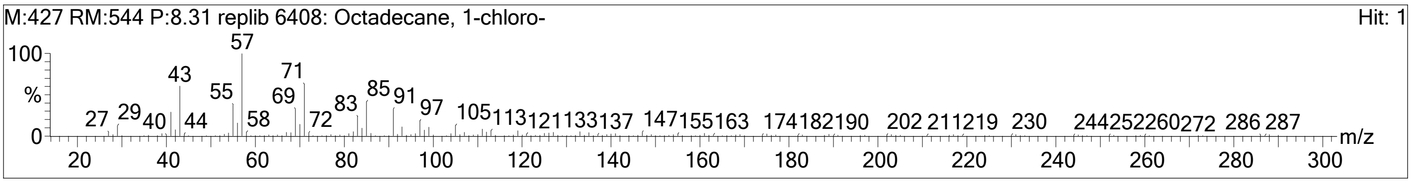

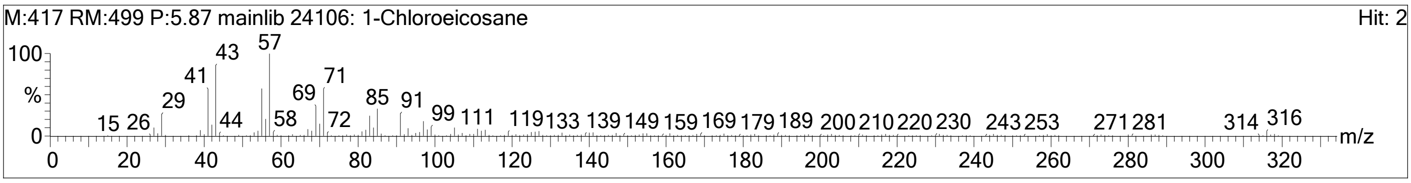

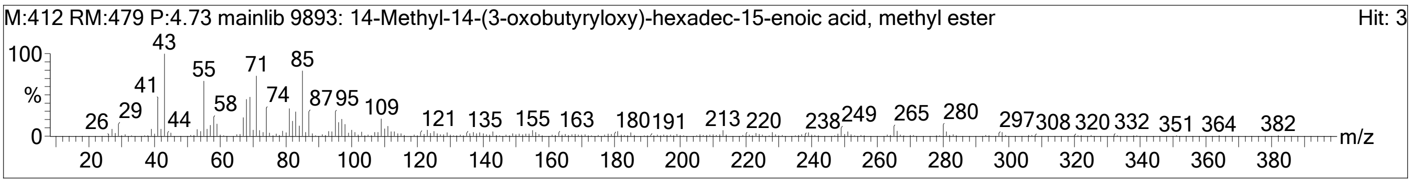

TAMILNADU AGRICULTURAL UNIVERSITY - AGRICULTURAL MICROBIOLOGY

INSTRUMENT: PERKIN ELMER CLARUS SQ8C COLOUMN: DB-5 MS CAPILARY STANDARD NON - POLARCOLOUMN
INJECTION VOL: 1 MICRO LITER DIMENSION: 30Mts, ID: 0.25 mm, FILM: 0.25 IM CARRIER GAS: He
SAMPLE ID : T360

#	RT	Scan	Height	Area	Area %	Norm %
20	29.854	5369	3,913,030	168,985.1	0.351	4.38

Pk #	RT	Hit	Compound Name	Match	R.Match	Prob.	CAS	Library
20	29.854	1	17-Pentatriacontene	408	435	6.3	6971-40-0	mainlib
		2	Oleic acid, eicosyl ester	404	430	5.3	22393-88-0	mainlib
		3	17-Pentatriacontene	388	463	6.3	6971-40-0	replib
		4	cis-11-Eicosenoic acid	387	462	2.9	5561-99-9	mainlib
		5	Spirostan-9-ol, 3-amino-, (3á,5à,25R)-	384	449	2.6	16577-35-8	mainlib
		6	2,5-Furandione, dihydro-3-octadecyl-	383	450	2.5	47458-32-2	mainlib
		7	1-Hexacosanol	382	419	2.4	506-52-5	replib
		8	9-Octadecenoic acid (Z)-, hexadecyl ester	380	429	2.2	22393-86-8	mainlib
		9	2-Nonadecanone 2,4-dinitrophenylhydrazine	380	424	2.2	28813-61-8	mainlib
		10	Oleic acid, 3-(octadecyloxy)propyl ester	379	445	2.1	17367-41-8	mainlib

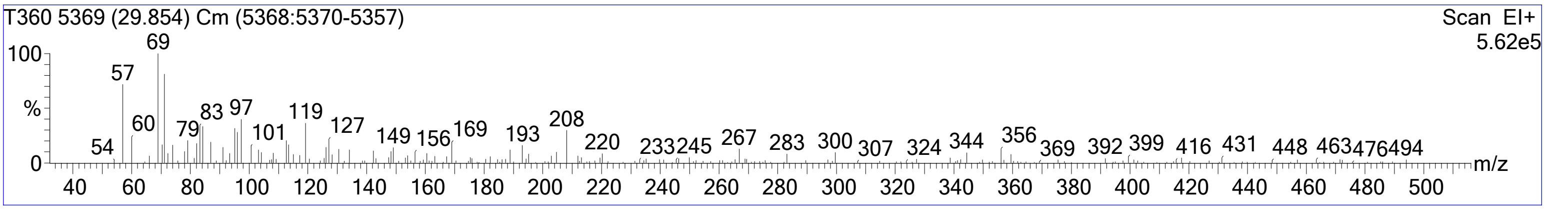

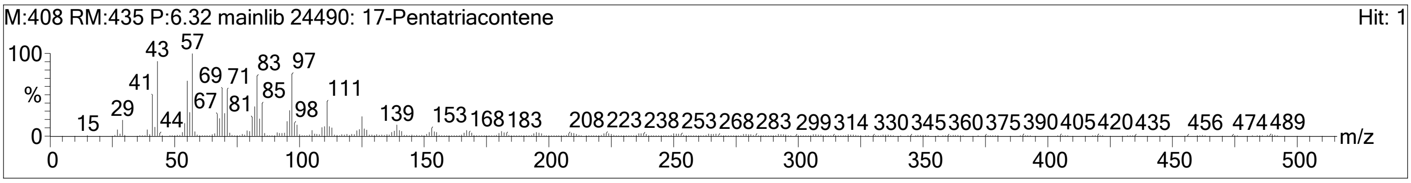

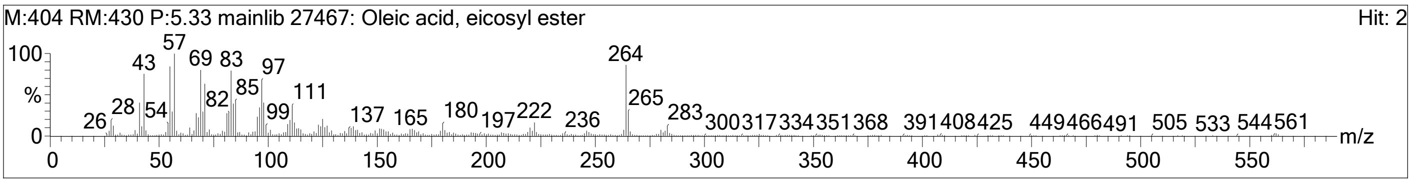

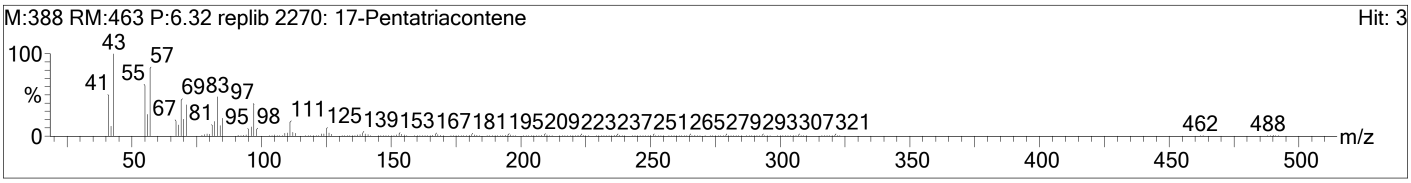

TAMILNADU AGRICULTURAL UNIVERSITY - AGRICULTURAL MICROBIOLOGY

INSTRUMENT: PERKIN ELMER CLARUS SQ8C   COLOUMN: DB-5 MS CAPILARY STANDARD NON - POLARCOLOUMN
INJECTION VOL: 1 MICRO LITER   DIMENSION: 30Mts, ID: 0.25 mm, FILM: 0.25 IM   CARRIER GAS: He
SAMPLE ID : T360

#	RT	Scan	Height	Area	Area %	Norm %
21	30.064	5411	6,108,846	143,558.7	0.298	3.72

Pk #	RT	Hit	Compound Name	Match	R.Match	Prob.	CAS	Library
21	30.064	1	3-Isopropyl-6a,10b-dimethyl-8-(2-oxo-2-phenyl-ethyl)-dodecahydro-benzo[f]chromen-7-one	453	491	16.9		mainlib
		2	Sulfide, bis(2-cyano-3,4-dihydro-2,3,3-trimethyl-2H-pyrrol-5-yl)-	430	500	6.2		mainlib
		3	Rhodopin	420	426	4.3	105-92-0	mainlib
		4	2-Picenol, 4,4,6a,6b,8a,11,11,14b-octamethylperhydro	411	429	3.2		mainlib
		5	(2R,3R,4aR,5S,8aS)-2-Hydroxy-4a,5-dimethyl-3-(prop-1-en-2-yl)octahydronaphthalen-1(2H)-one	410	504	3.0	66884-74-0	mainlib
		6	1-Benzazirene-1-carboxylic acid, 2,2,5a-trimethyl-1a-[3-oxo-1-butenyl] perhydro-, methyl ester	408	488	2.8		mainlib
		7	Cyclobutane, 1,3-bis[2-(2-isopropyl-3,3-dimethyloxiran-2-yl)ethenyl]-2,4-diacetyl-	408	435	2.8	70373-02-3	mainlib
		8	.psi.,.psi.-Carotene, 1,1',2,2'-tetrahydro-1,1'-dimethoxy-	408	408	2.8	13833-01-7	mainlib
		9	Corynan-17-ol, 18,19-didehydro-10-methoxy-, acetate (ester)	405	442	2.5	56053-13-5	mainlib
		10	6-Chloro-1-ethyl-4-oxoquinoline-3-carboxylic acid	403	544	2.3	66176-24-7	mainlib

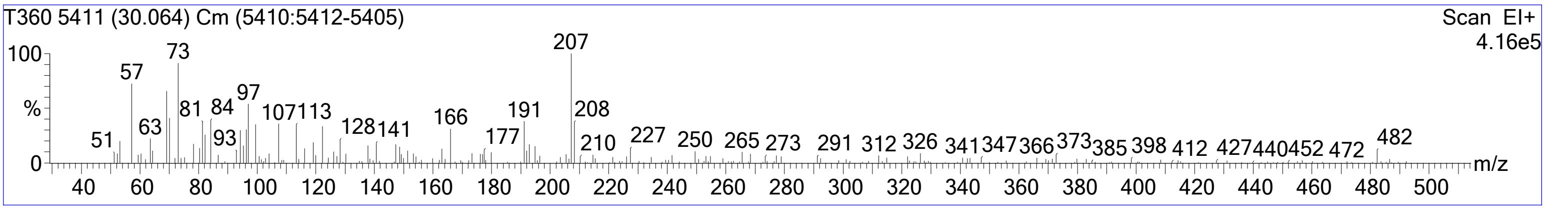

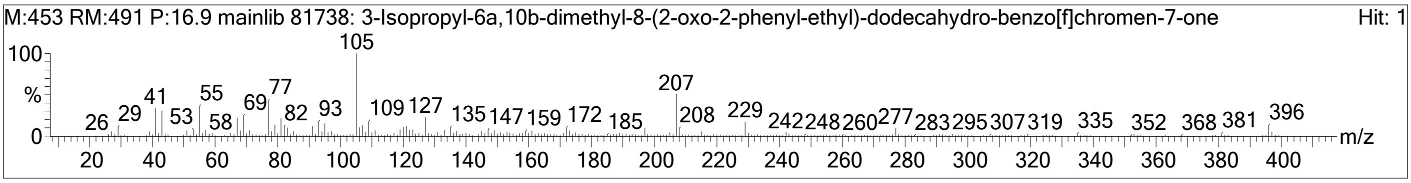

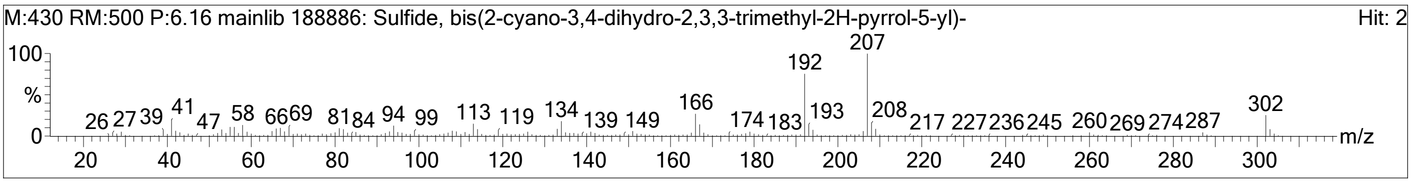

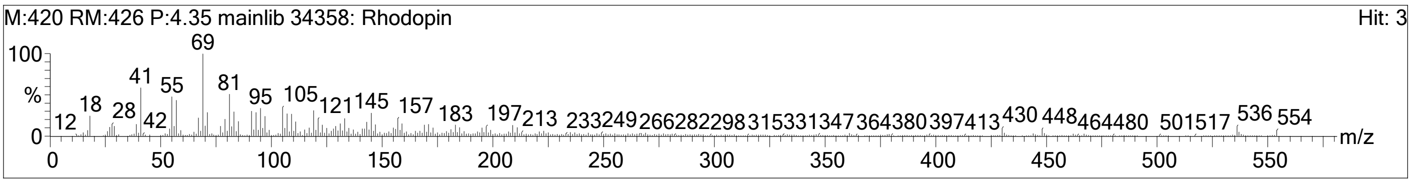

TAMILNADU AGRICULTURAL UNIVERSITY - AGRICULTURAL MICROBIOLOGY

INSTRUMENT: PERKIN ELMER CLARUS SQ8C COLOUMN: DB-5 MS CAPILARY STANDARD NON - POLARCOLOUMN
INJECTION VOL: 1 MICRO LITER DIMENSION: 30Mts, ID: 0.25 mm, FILM: 0.25 IM CARRIER GAS: He
SAMPLE ID : T360

#	RT	Scan	Height	Area	Area %	Norm %
22	30.699	5538	3,934,182	186,010.6	0.386	4.82

Pk #	RT	Hit	Compound Name	Match	R.Match	Prob.	CAS	Library
22	30.699	1	2-Chloroadenosine	444	746	19.6	146-77-0	nist_msms
		2	7,7,9,9,11,11-Hexamethyl-3,6,8,10,12,15-hexaoxa-7,9,11-trisilaheptadecane	409	513	4.9		mainlib
		3	6-Amino-5-cyano-4-(5-cyano-2,4-dimethyl-1H-pyrrol-3-yl)-2-methyl-4H-pyran-3-carboxylic acid ethyl ester	402	466	3.8		mainlib
		4	1,2-Benzisothiazol-3-amine, TBDMS derivative	396	534	3.0		mainlib
		5	6-Methoxypurine, TBDMS derivative	394	506	2.7		mainlib
		6	4-Hydroxychalcone	390	777	2.3	20426-12-4	nist_msms
		7	2-Oxa-4-azabicyclo[4.2.0]octa-3,7-diene-6-carboxylic acid, 1,7,8-tris(1,1-dimethylethyl)-3-(2,2-dimethylpropyl)-5-phenyl-, 1,1-dimethylethyl ester	390	438	2.3	101160-38-7	mainlib
		8	4'-Hydroxychalcone	387	769	2.0	2657-25-2	nist_msms
		9	à-N-Normethadol	387	437	2.0	38455-85-5	mainlib
		10	Demecolcine	386	420	2.0	477-30-5	mainlib

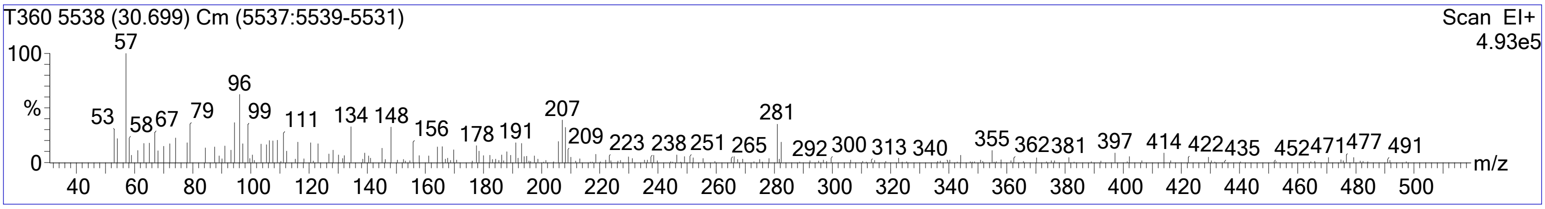

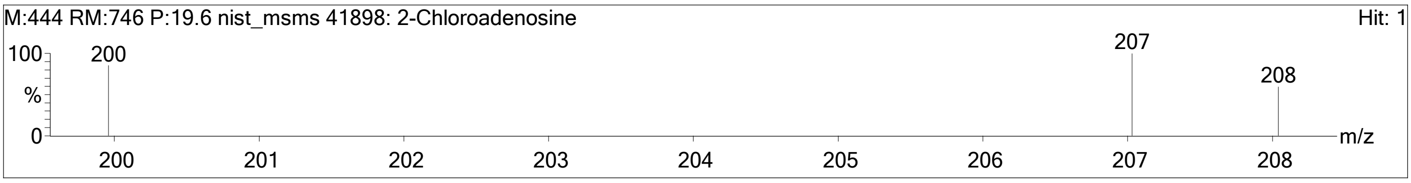

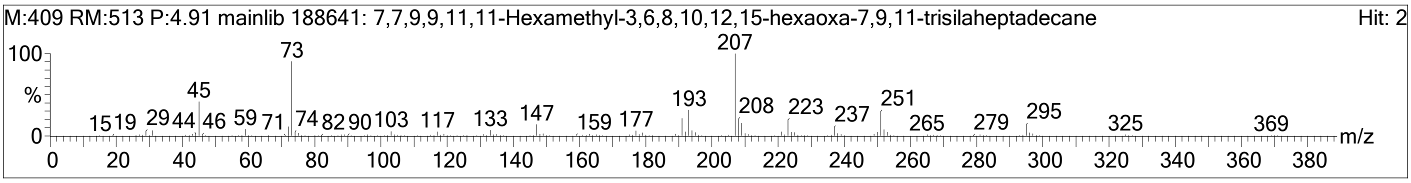

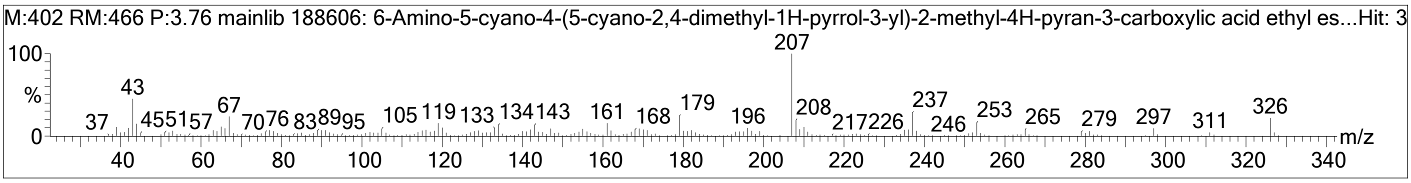

TAMILNADU AGRICULTURAL UNIVERSITY - AGRICULTURAL MICROBIOLOGY

INSTRUMENT: PERKIN ELMER CLARUS SQ8C COLOUMN: DB-5 MS CAPILARY STANDARD NON - POLARCOLOUMN
INJECTION VOL: 1 MICRO LITER DIMENSION: 30Mts, ID: 0.25 mm, FILM: 0.25 IM CARRIER GAS: He
SAMPLE ID : T360

#	RT	Scan	Height	Area	Area %	Norm %
23	31.155	5629	4,491,160	155,678.0	0.323	4.03

Pk #	RT	Hit	Compound Name	Match	R.Match	Prob.	CAS	Library
23	31.155	1	7,8-Epoxylanostan-11-ol, 3-acetoxy-	437	440	17.3		mainlib
		2	1-Heptatriacotanol	421	501	10.0	105794-58-9	mainlib
		3	Rhodopin	420	423	9.6	105-92-0	mainlib
		4	17-(1,5-Dimethylhexyl)-10,13-dimethylhexadecahydrocyclopenta[a]phenanthrene-3,5,6-triol	400	429	4.4	115510-05-9	mainlib
		5	Cholestane, 3,5-dichloro-6-nitro-, (3á,5à,6á)-	399	404	4.2	15505-92-7	mainlib
		6	Docosanedioic acid, dimethyl ester	398	463	4.0	22399-98-0	replib
		7	Z-10-Methyl-11-tetradecen-1-ol propionate	396	469	3.7		mainlib
		8	Boroxin, tris(2,3-dimethylbut-2-yl)-	390	458	2.9	169695-37-8	mainlib
		9	Cholest-5-en-3-ol, 6-nitro-, acetate (ester), (3á)-	389	437	2.8	1912-54-5	replib
		10	9,19-Cyclolanostane-3,7-diol	382	394	2.2	1036383-94-4	mainlib

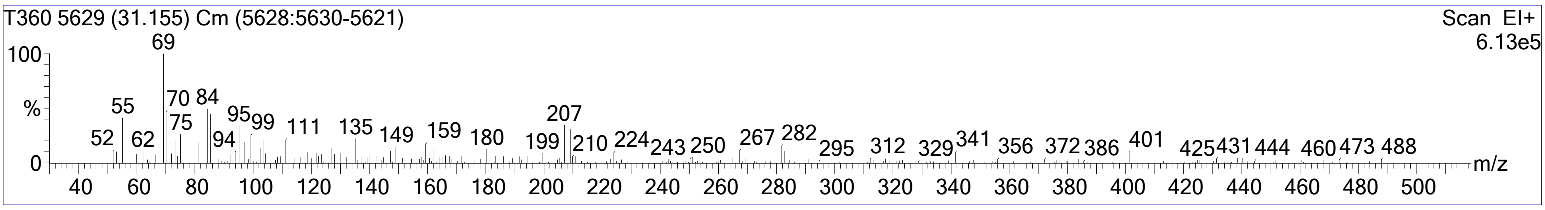

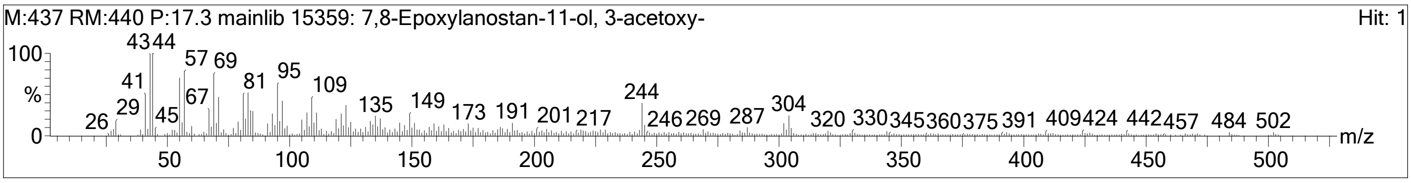

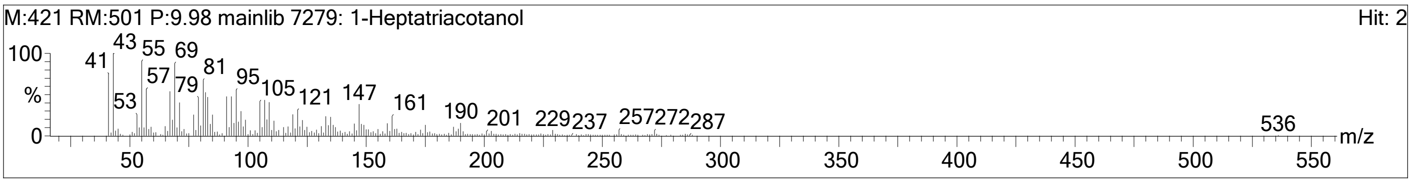

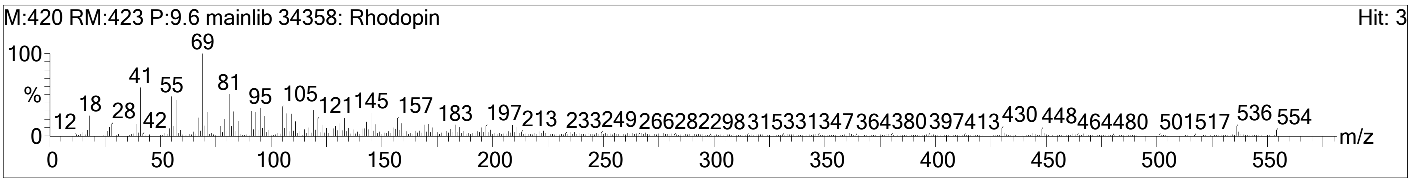

TAMILNADU AGRICULTURAL UNIVERSITY - AGRICULTURAL MICROBIOLOGY

INSTRUMENT: PERKIN ELMER CLARUS SQ8C　　COLOUMN: DB-5 MS CAPILARY STANDARD NON - POLARCOLOUMN
INJECTION VOL: 1 MICRO LITER　　DIMENSION: 30Mts, ID: 0.25 mm, FILM: 0.25 IM　　CARRIER GAS: He
SAMPLE ID : T360

#	RT	Scan	Height	Area	Area %	Norm %
24	31.430	5684	4,663,358	156,456.9	0.325	4.05

Pk #	RT	Hit	Compound Name	Match	R.Match	Prob.	CAS	Library
24	31.430	1	Rhodopin	429	433	16.5	105-92-0	mainlib
		2	Propanoic acid, 2-(3-acetoxy-4,4,14-trimethylandrost-8-en-17-yl)-	423	443	13.0		mainlib
		3	Glycine, N-[(3à,5á)-24-oxo-3-[(trimethylsilyl)oxy]cholan-24-yl]-, methyl ester	412	487	8.9	57326-15-5	mainlib
		4	11à-Hydroxyprogesterone, trimethylsilyl ether, bis(O-methyloxime)	392	411	4.1		mainlib
		5	Tibolone, trimethylsilyl ether	386	424	3.2		mainlib
		6	Arachidonoyl ethanolamide	385	480	3.1	94421-68-8	mainlib
		7	ent-3a-Acetoxy-10-hydroxy-13-iodomethyl-16-oxo-8,13-epi-17,20-dinorgibberell-1-ene-7,19-dioic acid 19,10-lactone	376	442	2.2		mainlib
		8	Trinexapac-ethyl, TMS derivative	374	437	2.0		mainlib
		9	2-[3-(4-tert-Butyl-phenoxy)-2-hydroxy-propylsulfanyl]-4,6-dimethyl-nicotinonitrile	370	504	1.7		mainlib
		10	5àAndrost-16-ol, 17-ethylidene-3,5-dedihydro-6-methoxy-, pivalate	370	462	1.7		mainlib

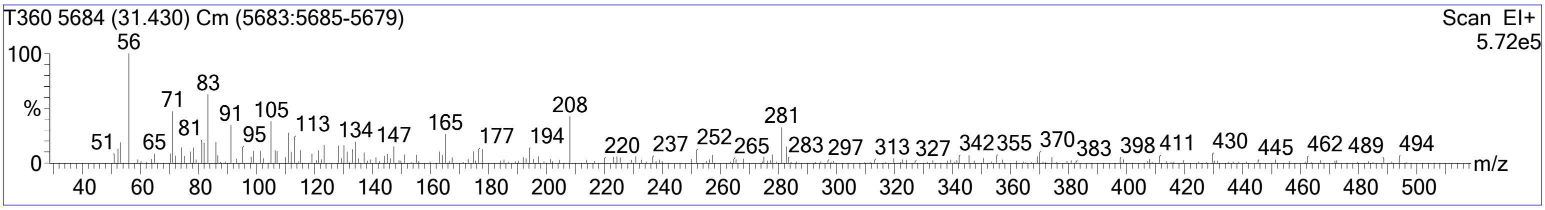

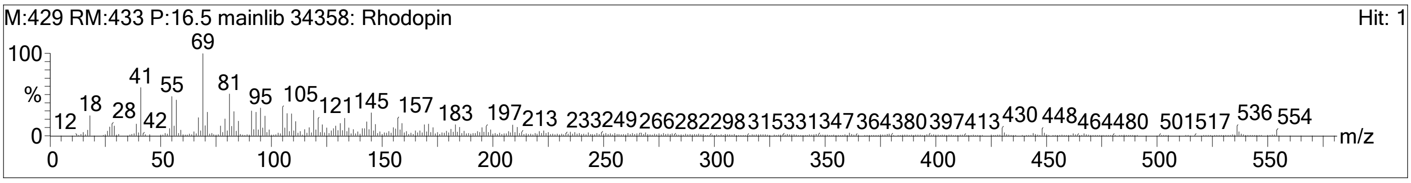

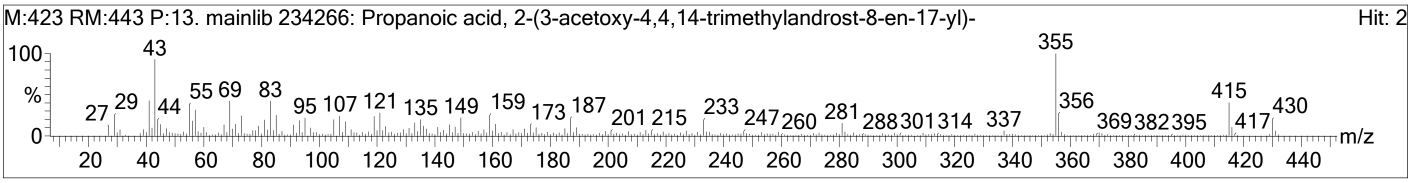

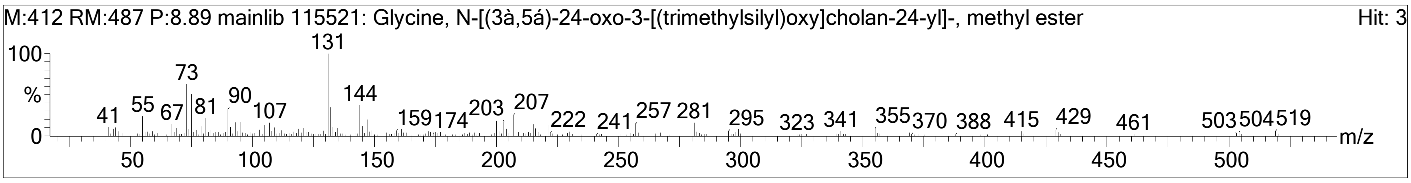

TAMILNADU AGRICULTURAL UNIVERSITY - AGRICULTURAL MICROBIOLOGY

INSTRUMENT: PERKIN ELMER CLARUS SQ8C   COLOUMN: DB-5 MS CAPILARY STANDARD NON - POLARCOLOUMN
INJECTION VOL: 1 MICRO LITER   DIMENSION: 30Mts, ID: 0.25 mm, FILM: 0.25 IM   CARRIER GAS: He
SAMPLE ID : T360

#	RT	Scan	Height	Area	Area %	Norm %
25	31.590	5716	4,413,812	117,499.8	0.244	3.04

Pk #	RT	Hit	Compound Name	Match	R.Match	Prob.	CAS	Library
25	31.590	1	Glafenin	452	789	13.0	3820-67-5	nist_msms
		2	4-[4-(2-Methoxyphenyl)-1H-pyrazol-3-yl]benzene-1,3-diol	434	512	6.7		mainlib
		3	Staurosporine	428	753	5.3	62996-74-1	nist_msms
		4	Gibb-3-ene-1,10-dicarboxylic acid, 2,4a-dihydroxy-1-methyl-8-methylene-, 1,4a-lactone, 10-methyl ester, (1à,2á,4aà,4bá,10á)-	426	510	4.9	5508-47-4	mainlib
		5	1h-Pyrrole-3,4-diacetic acid, 2-acetoxymethyl-5-methoxycarbonyl-, dimethyl ester	422	477	4.1		mainlib
		6	Propane-1,1,2,2-tetracarbonitrile, 3-(4-acetyl-2,5-dimethyl-3-furanoyl)-	419	482	3.6		mainlib
		7	2,6-Dihydroxyacetophenone, 2TMS derivative	416	507	3.2		mainlib
		8	5-(p-Aminophenyl)-4-(O-tolyl)-2-thiazolamine	414	502	3.0		mainlib
		9	2-(4-Chlorophenyl)-5,7-dimethylimidazo[1,2-a]pyridine-8-carbonitrile	412	530	2.7		mainlib
		10	Glafenin	410	631	13.0	3820-67-5	nist_msms

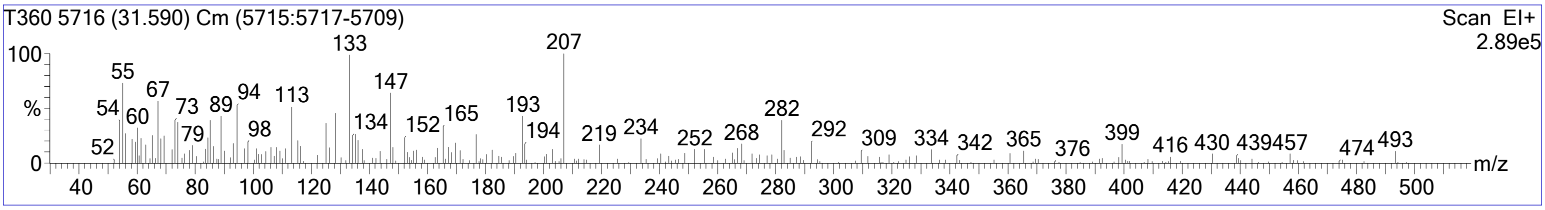

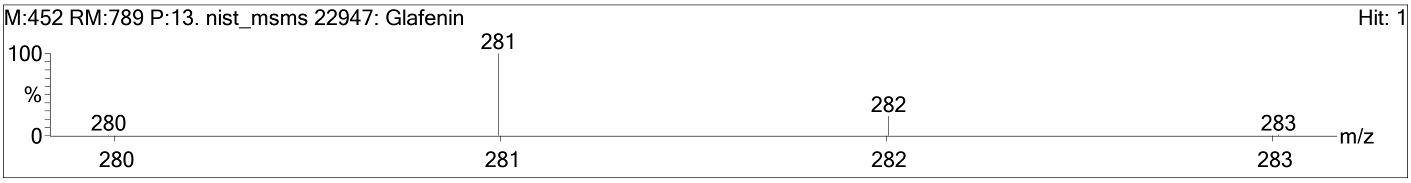

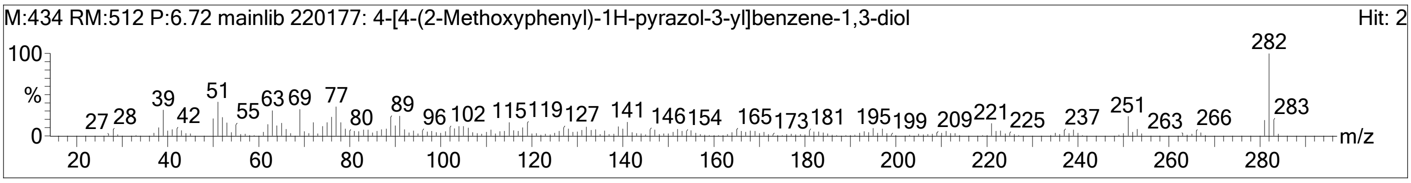

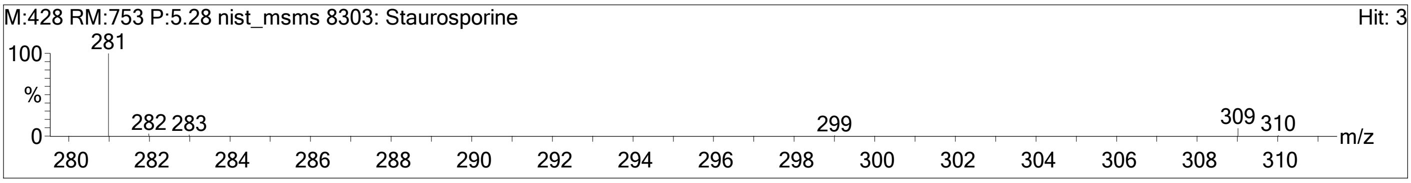

TAMILNADU AGRICULTURAL UNIVERSITY - AGRICULTURAL MICROBIOLOGY

INSTRUMENT: PERKIN ELMER CLARUS SQ8C COLOUMN: DB-5 MS CAPILARY STANDARD NON - POLARCOLOUMN
INJECTION VOL: 1 MICRO LITER DIMENSION: 30Mts, ID: 0.25 mm, FILM: 0.25 IM CARRIER GAS: He
SAMPLE ID : T360

#	RT	Scan	Height	Area	Area %	Norm %
26	31.635	5725	5,936,416	142,236.8	0.295	3.68

Pk #	RT	Hit	Compound Name	Match	R.Match	Prob.	CAS	Library
26	31.635	1	6-Azacholest-4-en-7-one, 6-benzyl-3à-hydroxy-	400	412	15.4	17373-01-2	mainlib
		2	3-[3-(1,5-Dimethylhexyl)-7-(2-hydroxy-1-methylethyl)-3a,6,9b-trimethyl-2,3,3a,4,5,6,7,8,9,9b-decahydro-1H-cyclopenta[a]naphthalen-6-yl]propanoic acid, methyl ester	387	396	10.0		mainlib
		3	Acetamide, 2-chloro-N-(3-cyano-4,6-dihydro-4,4,6,6-tetramethylthieno[2,3-c]furan-2-yl)-	376	465	6.8		mainlib
		4	2-(Trimethylsilyl)oxybenzylidene acetophenone	373	425	6.0		mainlib
		5	3-Hydroxy-4,4,6a,8a,11,11,14b-heptamethyl-1,3,4,4a,5,6,6a,7,8,8a,9,10,11,12,12a,14,14a,14b-octadecahydro-2H-picen-13-one	364	384	4.4		mainlib
		6	(E)-1-(2-Hydroxy-4,6-dimethoxyphenyl)-3-phenylprop-2-en-1-one	356	443	3.3	1775-97-9	mainlib
		7	Malonic acid, 3,3-dimethylbut-2-yl tetradecyl ester	354	573	3.0		mainlib
		8	9-à-Hydroxyarteether	354	444	3.0	130778-40-4	mainlib
		9	9-Methylcorticosterone	352	408	2.8		mainlib
		10	N-Ethyl-2-phenyl-N-(pyridin-4-ylmethyl)-3-[(trimethylsilyl)oxy]propanamide	351	389	2.7		mainlib

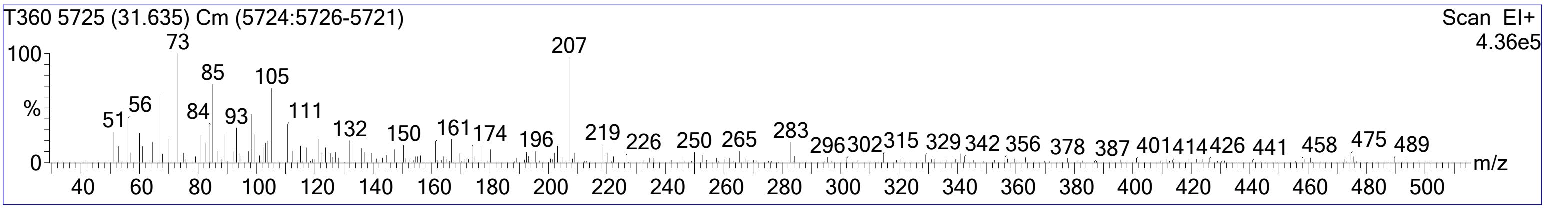

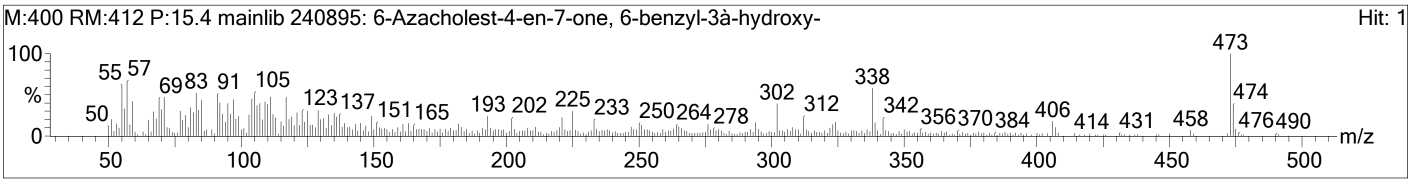

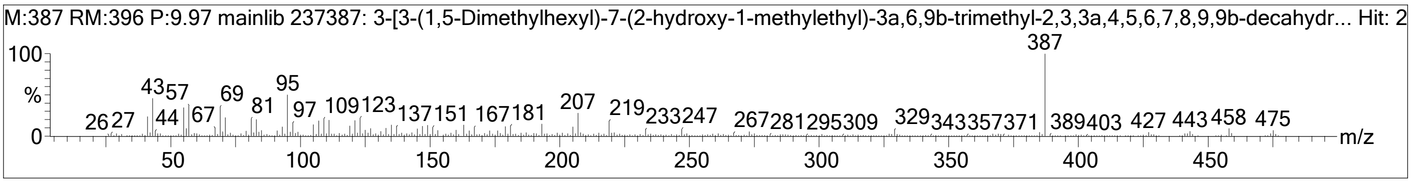

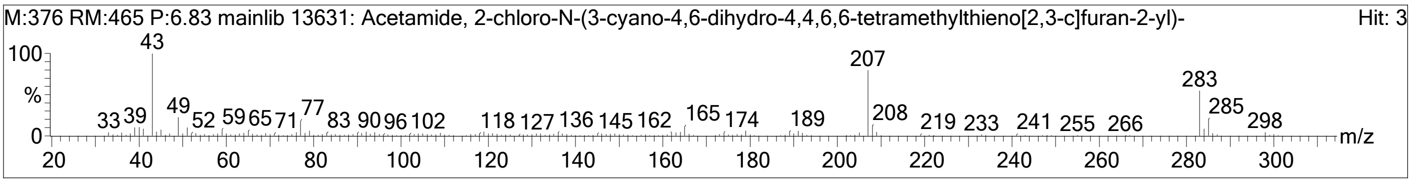

TAMILNADU AGRICULTURAL UNIVERSITY - AGRICULTURAL MICROBIOLOGY

INSTRUMENT: PERKIN ELMER CLARUS SQ8C COLOUMN: DB-5 MS CAPILARY STANDARD NON - POLARCOLOUMN
INJECTION VOL: 1 MICRO LITER DIMENSION: 30Mts, ID: 0.25 mm, FILM: 0.25 IM CARRIER GAS: He
SAMPLE ID : T360

#	RT	Scan	Height	Area	Area %	Norm %
27	31.795	5757	3,708,181	164,586.6	0.342	4.26

Pk #	RT	Hit	Compound Name	Match	R.Match	Prob.	CAS	Library
27	31.795	1	2,4-Di-tert-butylthiophenol	404	577	9.7	19728-43-9	mainlib
		2	5-Bromo-8-(5-nitrosalicylideneamino)quinoline	389	503	5.9	328030-21-3	mainlib
		3	2,4,6-Cycloheptatrien-1-one, 3,5-bis-trimethylsilyl-	388	543	5.7		mainlib
		4	Phenol, 2,6-dichloro-4-nitro-	378	498	4.0	618-80-4	mainlib
		5	1,4-Bis(trimethylsilyl)benzene	370	546	3.0	13183-70-5	mainlib
		6	1-(4-Chlorophenoxy)-1-(1H-imidazol-1-yl)-3,3-dimethylbutan-2-one	370	521	3.0		mainlib
		7	Clocortolone pivalate	370	406	3.0	34097-16-0	mainlib
		8	Octadecanoic acid, 4-hydroxy-, methyl ester	366	439	2.5	2420-38-4	mainlib
		9	1,2-Bis(trimethylsilyl)benzene	363	558	2.2	17151-09-6	mainlib
		10	Octadecane, 3-ethyl-5-(2-ethylbutyl)-	361	400	2.0	55282-12-7	mainlib

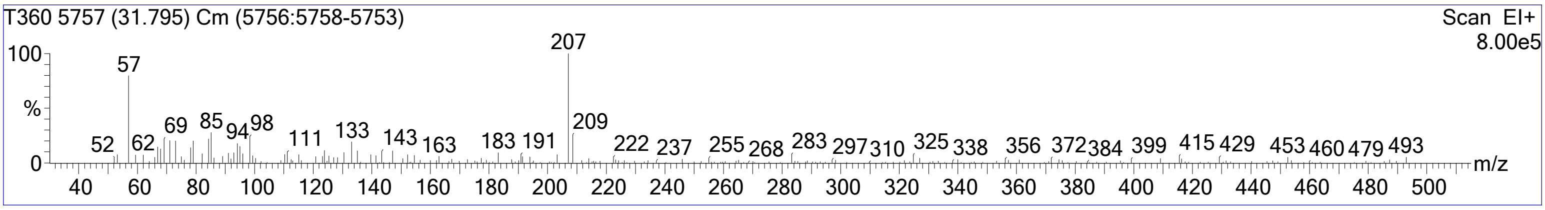

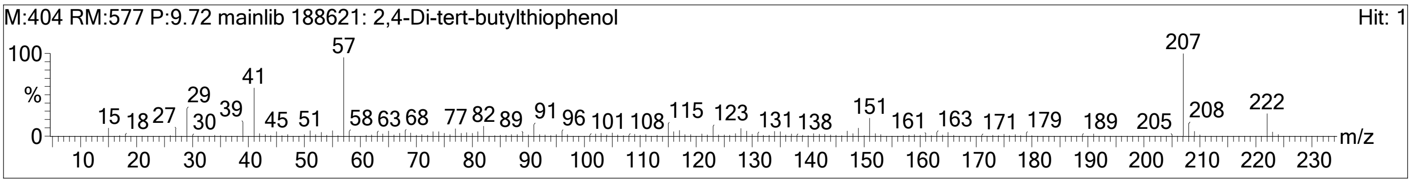

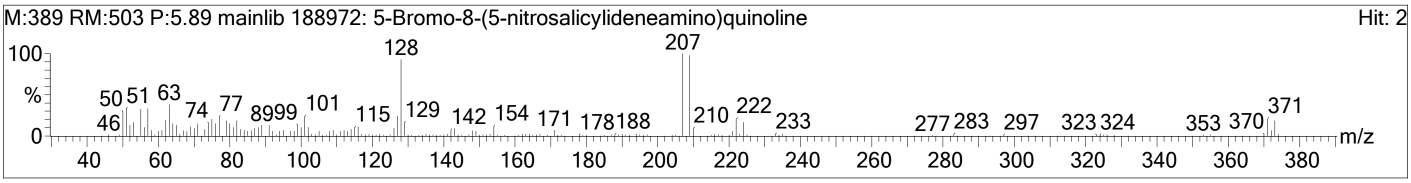

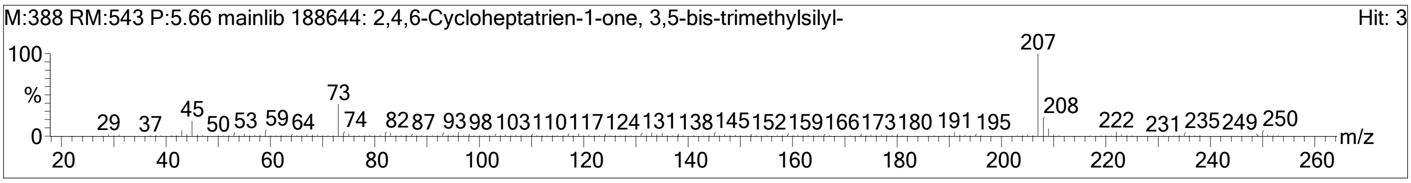

TAMILNADU AGRICULTURAL UNIVERSITY - AGRICULTURAL MICROBIOLOGY

INSTRUMENT: PERKIN ELMER CLARUS SQ8C	COLOUMN: DB-5 MS CAPILARY STANDARD NON - POLARCOLOUMN
INJECTION VOL: 1 MICRO LITER	DIMENSION: 30Mts, ID: 0.25 mm, FILM: 0.25 IM	CARRIER GAS: He
SAMPLE ID : T360

#	RT	Scan	Height	Area	Area %	Norm %
28	33.080	6014	5,036,046	121,006.1	0.251	3.13

Pk #	RT	Hit	Compound Name	Match	R.Match	Prob.	CAS	Library
28	33.080	1	Astaxanthin	432	433	28.7	472-61-7	mainlib
		2	4-Hydroxy-á-ionone	369	526	4.8	116296-75-4	mainlib
		3	3,4-Dimethoxycinnamic acid	364	528	3.9	2316-26-9	replib
		4	3,4-Dimethoxycinnamic acid	363	569	3.9	2316-26-9	mainlib
		5	2-Propenoic acid, 3-(3,4-dimethoxyphenyl)-, (E)-	363	500	3.7	14737-89-4	mainlib
		6	Stearic acid, 3-(octadecyloxy)propyl ester	356	386	2.8	17367-40-7	mainlib
		7	2,4-Dimethoxycinnamic acid	355	515	2.7	6972-61-8	mainlib
		8	10,12-Docosadiynedioic acid, 2TMS derivative	354	407	2.6		mainlib
		9	3,3'-Methylenebis(1,5,8,11-tetraoxacyclotridecane)	353	595	2.5	120343-85-3	mainlib
		10	3,4-Dimethoxycinnamic acid	353	544	3.9	2316-26-9	replib

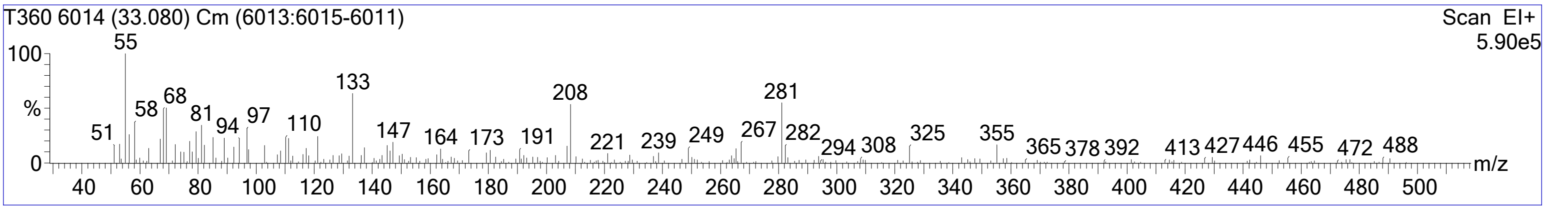

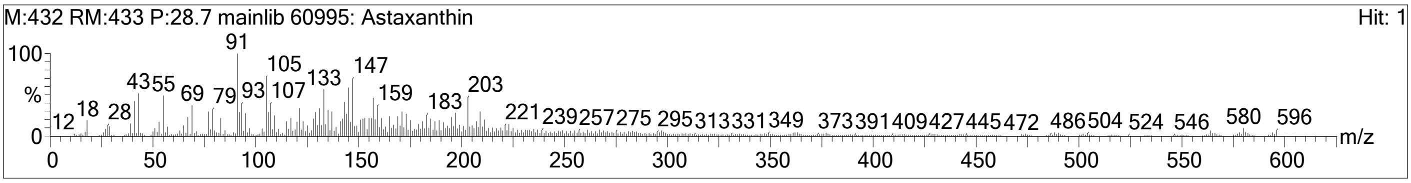

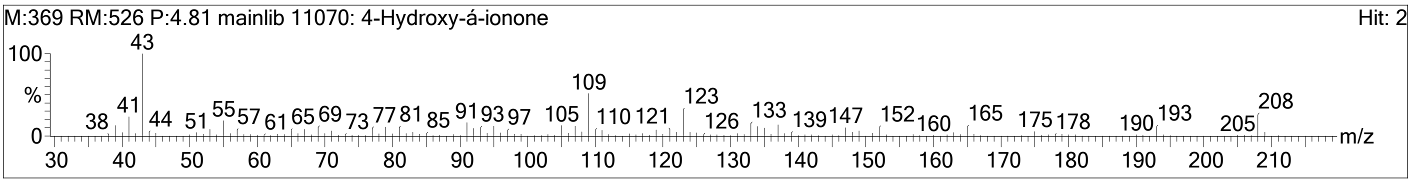

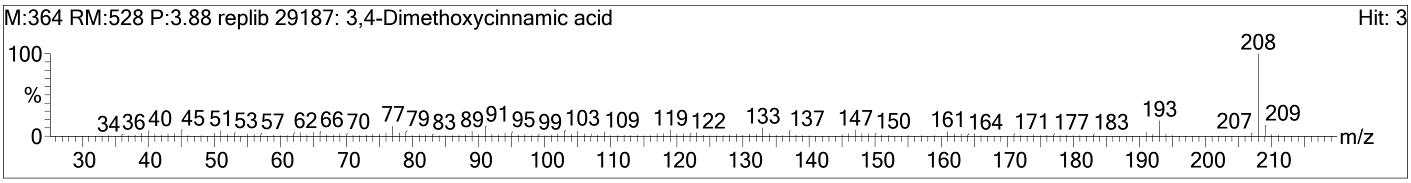

TAMILNADU AGRICULTURAL UNIVERSITY - AGRICULTURAL MICROBIOLOGY

INSTRUMENT: PERKIN ELMER CLARUS SQ8C COLOUMN: DB-5 MS CAPILARY STANDARD NON - POLARCOLOUMN
INJECTION VOL: 1 MICRO LITER DIMENSION: 30Mts, ID: 0.25 mm, FILM: 0.25 IM CARRIER GAS: He
SAMPLE ID : T360

#	RT	Scan	Height	Area	Area %	Norm %
29	33.596	6117	4,247,245	122,363.4	0.254	3.17

Pk #	RT	Hit	Compound Name	Match	R.Match	Prob.	CAS	Library
29	33.596	1	3,8,12-Tri-O-acetoxy-7-desoxyingol-7-one	388	419	27.1		mainlib
		2	Propanoic acid, 2-(3-acetoxy-4,4,14-trimethylandrost-8-en-17-yl)-	384	407	22.9		mainlib
		3	Corynan-17-ol, 18,19-didehydro-10-methoxy-, acetate (ester)	356	431	6.6	56053-13-5	mainlib
		4	Tibolone, trimethylsilyl ether	347	377	4.8		mainlib
		5	Glycine, N-[(3à,5á)-24-oxo-3-[(trimethylsilyl)oxy]cholan-24-yl]-, methyl ester	343	415	4.1	57326-15-5	mainlib
		6	5-Ethoxy-2-[4-(2-methoxyphenyl)-1H-pyrazol-3-yl]phenol	340	418	3.6		mainlib
		7	Acridin-1(2H)-one, 3,4-dihydro-9-allylamino-3,3-dimethyl-	337	415	3.2	300359-68-6	mainlib
		8	18,19-Seco-15á-yohimban-19-oic acid, 20,21-didehydro-16á-(hydroxymethyl)-, methyl ester	331	406	2.5	5552-25-0	mainlib
		9	Acetic acid, 17-(1,5-dimethylhexyl)-4,4,10,13,17-pentamethyl-2,3,4,5,6,7,10,11,12,13,16,17-dodecahydro-1H-cyclopenta[a]phenanthrene-3-ol (ester)	328	352	2.2		mainlib
		10	2-[p-Chlorostyryl]-5,8-dimethoxy-6-nitroquinoline	324	405	1.9		mainlib

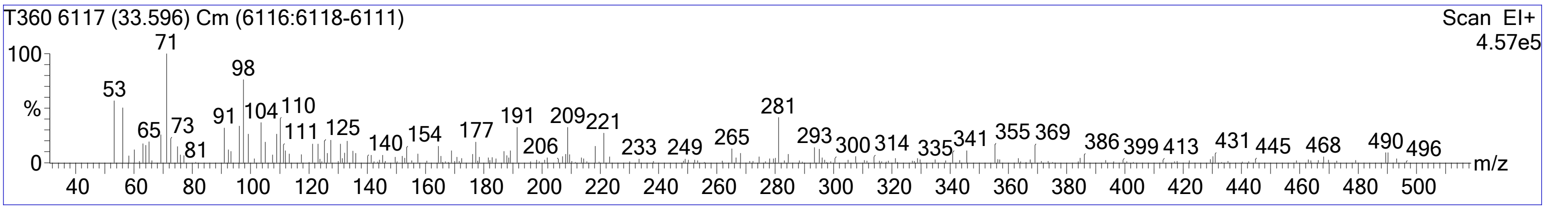

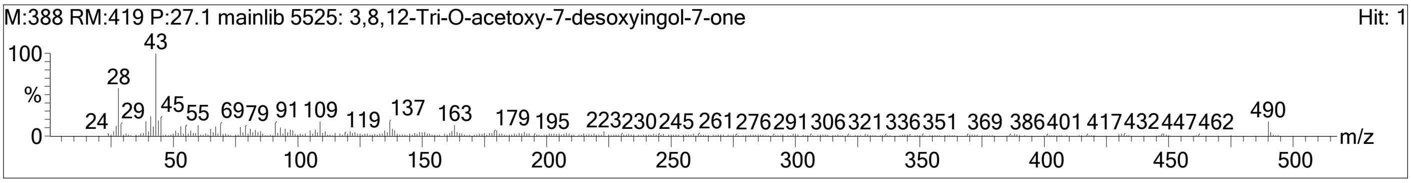

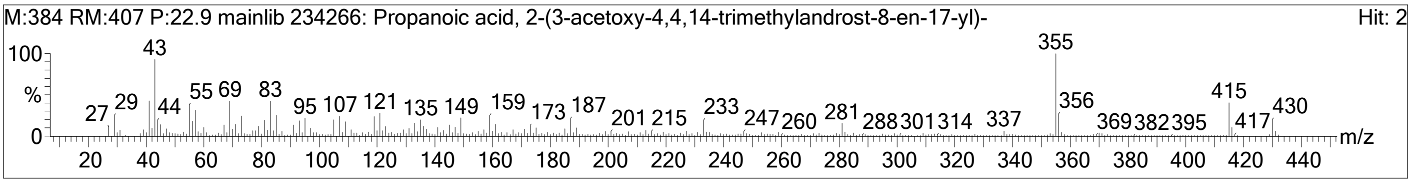

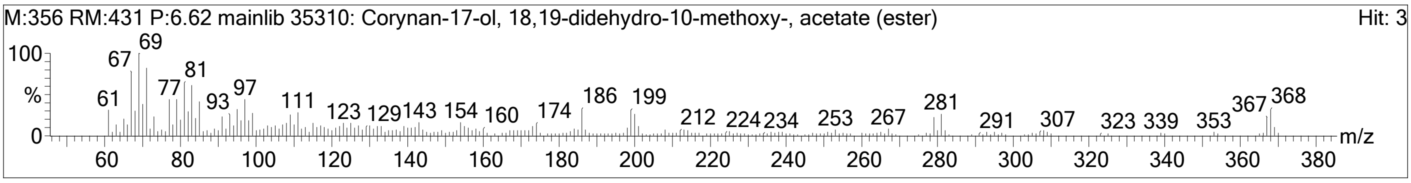

TAMILNADU AGRICULTURAL UNIVERSITY - AGRICULTURAL MICROBIOLOGY

INSTRUMENT: PERKIN ELMER CLARUS SQ8C COLOUMN: DB-5 MS CAPILARY STANDARD NON - POLARCOLOUMN
INJECTION VOL: 1 MICRO LITER DIMENSION: 30Mts, ID: 0.25 mm, FILM: 0.25 IM CARRIER GAS: He
SAMPLE ID : T360

#	RT	Scan	Height	Area	Area %	Norm %
30	34.171	6232	3,976,397	134,905.9	0.280	3.49

Pk #	RT	Hit	Compound Name	Match	R.Match	Prob.	CAS	Library
30	34.171	1	DISTEARIN	399	435	14.1	1188-58-5	mainlib
		2	Picrotoxin	377	511	5.6	124-87-8	mainlib
		3	9-Octadecenoic acid (Z)-, 2-hydroxy-3-[(1-oxooctadecyl)oxy]propyl ester	376	427	5.4	18266-27-8	mainlib
		4	Octadecanoic acid, 4-hydroxy-, methyl ester	375	465	5.2	2420-38-4	mainlib
		5	2(3H)-Furanone, dihydro-5-tetradecyl-	371	476	4.3	502-26-1	mainlib
		6	17á-Acetoxy-1',1'-dicarboethoxy-1á,2á-dihydro-17à-methyl-3'H-cycloprop[1,2]-5à-androst-1-en-3-one	367	382	3.7	80097-22-9	mainlib
		7	1,2-Propanediol, 3-(octadecyloxy)-, diacetate	366	445	3.5	21994-81-0	mainlib
		8	2H-3,7-Methanoazacycloundecino[5,4-b]indole-9-carboxylic acid, 5-ethyl-1,4,5,6,7,8,9,10-octahydro-, methyl ester, [5S-(5R*,7R*,9S*)]-	363	470	3.1	26251-90-1	replib
		9	3,5,9-Trioxa-4-phosphaheptacosan-1-aminium, 4-hydroxy-N,N,N-trimethyl-10-oxo-7-[(1-oxooctadecyl)oxy]-, hydroxide, inner salt, 4-oxide, (R)-	362	377	3.0	816-94-4	mainlib
		10	25-Norisopropyl-9,19-cyclolanostan-22-en-24-one, 3-acetoxy-24-phenyl-4,4,14-trimethyl-	360	381	2.8		mainlib

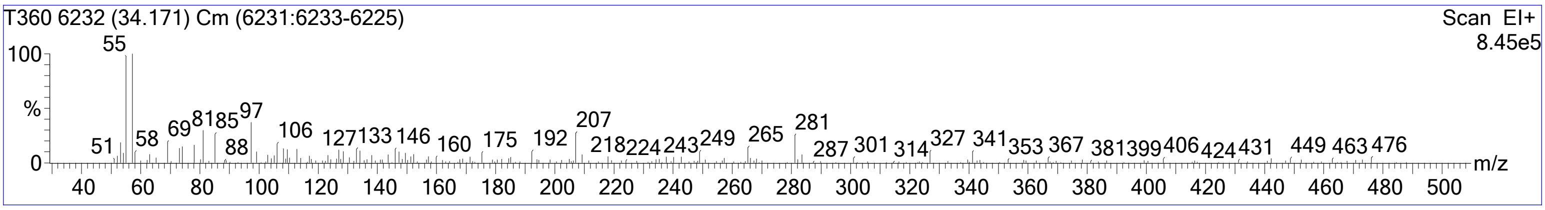

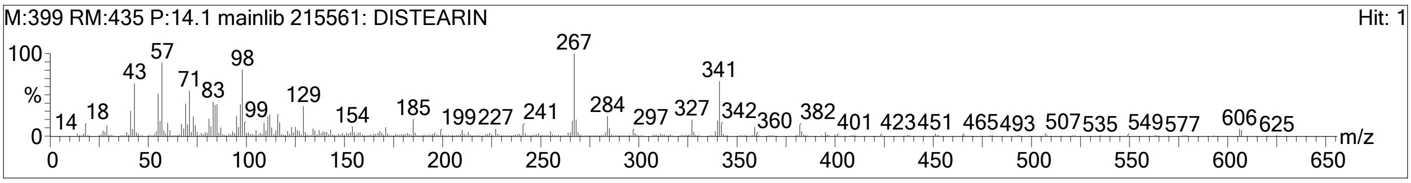

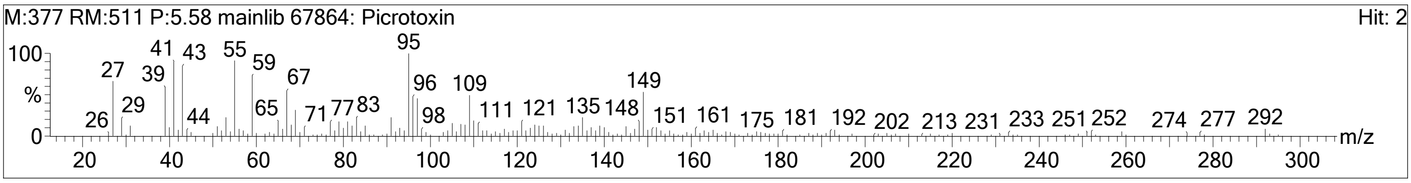

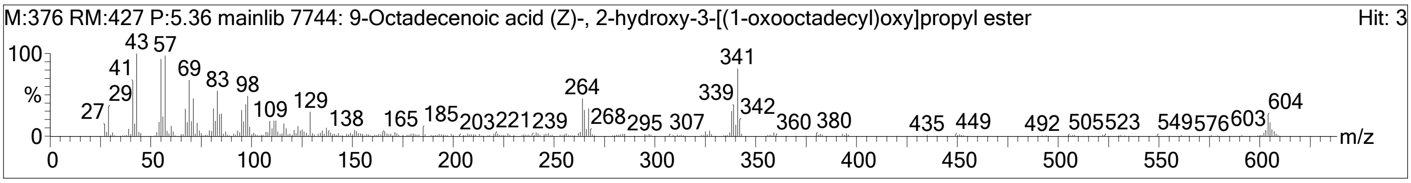

Supplement: S5 Fig — (PDF) [file pone.0219014.s011.pdf]
